# The OpenMolcas *Web*: A Community-Driven Approach to Advancing Computational Chemistry

**DOI:** 10.1021/acs.jctc.3c00182

**Published:** 2023-05-22

**Authors:** Giovanni Li Manni, Ignacio Fdez. Galván, Ali Alavi, Flavia Aleotti, Francesco Aquilante, Jochen Autschbach, Davide Avagliano, Alberto Baiardi, Jie J. Bao, Stefano Battaglia, Letitia Birnoschi, Alejandro Blanco-González, Sergey I. Bokarev, Ria Broer, Roberto Cacciari, Paul B. Calio, Rebecca K. Carlson, Rafael Carvalho Couto, Luis Cerdán, Liviu F. Chibotaru, Nicholas F. Chilton, Jonathan Richard Church, Irene Conti, Sonia Coriani, Juliana Cuéllar-Zuquin, Razan E. Daoud, Nike Dattani, Piero Decleva, Coen de Graaf, Mickaël G. Delcey, Luca De Vico, Werner Dobrautz, Sijia S. Dong, Rulin Feng, Nicolas Ferré, Michael Filatov(Gulak), Laura Gagliardi, Marco Garavelli, Leticia González, Yafu Guan, Meiyuan Guo, Matthew R. Hennefarth, Matthew R. Hermes, Chad E. Hoyer, Miquel Huix-Rotllant, Vishal Kumar Jaiswal, Andy Kaiser, Danil S. Kaliakin, Marjan Khamesian, Daniel S. King, Vladislav Kochetov, Marek Krośnicki, Arpit Arun Kumaar, Ernst D. Larsson, Susi Lehtola, Marie-Bernadette Lepetit, Hans Lischka, Pablo López Ríos, Marcus Lundberg, Dongxia Ma, Sebastian Mai, Philipp Marquetand, Isabella C. D. Merritt, Francesco Montorsi, Maximilian Mörchen, Artur Nenov, Vu Ha Anh Nguyen, Yoshio Nishimoto, Meagan S. Oakley, Massimo Olivucci, Markus Oppel, Daniele Padula, Riddhish Pandharkar, Quan Manh Phung, Felix Plasser, Gerardo Raggi, Elisa Rebolini, Markus Reiher, Ivan Rivalta, Daniel Roca-Sanjuán, Thies Romig, Arta Anushirwan Safari, Aitor Sánchez-Mansilla, Andrew M. Sand, Igor Schapiro, Thais R. Scott, Javier Segarra-Martí, Francesco Segatta, Dumitru-Claudiu Sergentu, Prachi Sharma, Ron Shepard, Yinan Shu, Jakob K. Staab, Tjerk P. Straatsma, Lasse Kragh Sørensen, Bruno Nunes Cabral Tenorio, Donald G. Truhlar, Liviu Ungur, Morgane Vacher, Valera Veryazov, Torben Arne Voß, Oskar Weser, Dihua Wu, Xuchun Yang, David Yarkony, Chen Zhou, J. Patrick Zobel, Roland Lindh

**Affiliations:** 1 Electronic Structure Theory Department, Max Planck Institute for Solid State Research, Heisenbergstraße 1, 70569 Stuttgart, Germany; 2 Department of Chemistry − BMC, Uppsala University, P.O. Box 576, SE-75123 Uppsala, Sweden; 3 Yusuf Hamied Department of Chemistry, University of Cambridge, Lensfield Road, Cambridge CB2 1EW, United Kingdom; 4 Department of Industrial Chemistry “Toso Montanari”, University of Bologna, 40136 Bologna, Italy; 5 Theory and Simulation of Materials (THEOS) and National Centre for Computational Design and Discovery of Novel Materials (MARVEL), École Polytechnique Fédérale de Lausanne (EPFL), CH-1015 Lausanne, Switzerland; 6 Department of Chemistry, University at Buffalo, State University of New York, Buffalo, New York 14260-3000, United States; 7 ETH Zurich, Laboratory for Physical Chemistry, Vladimir-Prelog-Weg 2, 8093 Zurich, Switzerland; 8 Department of Chemistry, Chemical Theory Center, and Minnesota Supercomputing Institute, University of Minnesota, Minneapolis, Minnesota 55455-0431, United States; 10 The Department of Chemistry, The University of Manchester, M13 9PL, Manchester, U.K.; 11 Chemistry Department, Bowling Green State University, Overmann Hall, Bowling Green, Ohio 43403, United States; 12 Institut für Physik, Universität Rostock, Albert-Einstein-Str. 23-24, 18059 Rostock, Germany; 13 Chemistry Department, School of Natural Sciences, Technical University of Munich, Lichtenbergstr. 4, 85748 Garching, Germany; 14 Theoretical Chemistry, Zernike Institute for Advanced Materials, University of Groningen, Nijenborgh 4, 9747AG Groningen, The Netherlands; 15 Dipartimento di Biotecnologie, Chimica e Farmacia, Università di Siena, Via A. Moro 2, 53100 Siena, Italy; 16 Department of Chemistry, Pritzker School of Molecular Engineering, James Franck Institute, Chicago Center for Theoretical Chemistry, The University of Chicago, Chicago, Illinois 60637, United States; 17 Division of Theoretical Chemistry and Biology, School of Engineering Sciences in Chemistry, Biotechnology and Health, KTH Royal Institute of Technology, SE-106 91 Stockholm, Sweden; 18 Instituto de Ciencia Molecular, Universitat de València, Catedrático José Beltrán Martínez n. 2, 46980 Paterna, Spain; 19 Instituto de Óptica (IO−CSIC), Consejo Superior de Investigaciones Científicas, 28006, Madrid, Spain; 20 Department of Chemistry, KU Leuven, Celestijnenlaan 200F, 3001 Leuven, Belgium; 21 Institute of Chemistry, The Hebrew University of Jerusalem, Jerusalem 91904, Israel; 22 Department of Chemistry, Technical University of Denmark, Kemitorvet Bldg 207, 2800 Kongens Lyngby, Denmark; 23 HPQC Labs, Waterloo, N2T 2K9 Ontario Canada; 24 HPQC College, Waterloo, N2T 2K9 Ontario Canada; 25 Istituto Officina dei Materiali IOM-CNR and Dipartimento di Scienze Chimiche e Farmaceutiche, Università degli Studi di Trieste, I-34121 Trieste, Italy; 26 Department of Physical and Inorganic Chemistry, Universitat Rovira i Virgili, Tarragona 43007, Spain; 27 ICREA, Pg. Lluís Companys 23, 08010 Barcelona, Spain; 28 Chalmers University of Technology, Department of Chemistry and Chemical Engineering, 41296 Gothenburg, Sweden; 29 Department of Chemistry and Chemical Biology, Department of Physics, and Department of Chemical Engineering, Northeastern University, Boston, Massachusetts 02115, United States; 30 Department of Chemistry, Fudan University, Shanghai 200433, China; 31 Institut de Chimie Radicalaire (UMR-7273), Aix-Marseille Univ, CNRS, ICR 13013 Marseille, France; 32 Department of Chemistry, Kyungpook National University, Daegu 702-701, South Korea; 33 Institute of Theoretical Chemistry, Faculty of Chemistry, University of Vienna, Währinger Straße 17, A-1090 Vienna, Austria; 34 State Key Laboratory of Molecular Reaction Dynamics and Center for Theoretical Computational Chemistry, Dalian Institute of Chemical Physics, Chinese Academy of Sciences, Dalian 116023, People’s Republic of China; 35 SSRL, SLAC National Accelerator Laboratory, Menlo Park, California 94025, United States; 36 Department of Chemistry, University of Washington, Seattle, Washington 98195, United States; 37 Institut für Physik, Universität Rostock, Albert-Einstein-Str. 23-24, 18059 Rostock, Germany; 38 Institute of Theoretical Physics and Astrophysics, Faculty of Mathematics, Physics and Informatics, University of Gdańsk, ul Wita Stwosza 57, 80-952, Gdańsk, Poland; 39 Division of Theoretical Chemistry, Chemical Centre, Lund University, P.O. Box 124, SE-22100, Lund, Sweden; 40 Molecular Sciences Software Institute, Blacksburg, Virginia 24061, United States; 41 Department of Chemistry, University of Helsinki, P.O. Box 55, FI-00014 University of Helsinki, Finland; 42 Condensed Matter Theory Group, Institut Néel, CNRS UPR 2940, 38042 Grenoble, France; 43 Theory Group, Institut Laue Langevin, 38042 Grenoble, France; 44 Department of Chemistry and Biochemistry, Texas Tech University, Lubbock, Texas 79409-1061, United States; 45 Department of Chemistry − Ångström Laboratory, Uppsala University, SE-75120 Uppsala, Sweden; 46 Nantes Université, CNRS, CEISAM, UMR 6230, F-44000 Nantes, France; 47 Department of Chemistry, National University of Singapore, 3 Science Drive 3, 117543 Singapore; 49 Graduate School of Science, Kyoto University, Kyoto 606-8502, Japan; 50 Department of Chemistry, Graduate School of Science, Nagoya University, Furo-cho, Chikusa-ku, Nagoya, Aichi 464-8602, Japan; 51 Institute of Transformative Bio-Molecules (WPI-ITbM), Nagoya University, Furo-cho, Chikusa-ku, Nagoya, Aichi 464-8601, Japan; 52 Department of Chemistry, Loughborough University, Loughborough, LE11 3TU, U.K.; 53 Quantum Materials and Software LTD, 128 City Road, London, EC1V 2NX, United Kingdom; 54 Scientific Computing Group, Institut Laue Langevin, 38042 Grenoble, France; 55 Department of Chemistry and Biochemistry, Butler University, Indianapolis, Indiana 46208, United States; 56 Department of Chemistry, University of California, Irvine, California 92697, United States; 57 Laboratory RA-03, RECENT AIR, A. I. Cuza University of Iaşi, RA-03 Laboratory (RECENT AIR), Iaşi 700506, Romania; 58 Chemical Sciences and Engineering Division, Argonne National Laboratory, Lemont, Illinois 60439, USA; 59 National Center for Computational Sciences, Oak Ridge National Laboratory, Oak Ridge, Tennessee 37831-6373, United States; 60 Department of Chemistry and Biochemistry, University of Alabama, Tuscaloosa, Alabama 35487-0336, United States; 61 University Library, University of Southern Denmark, DK-5230 Odense M, Denmark; 62 Department of Chemistry, Johns Hopkins University, Baltimore, Maryland 21218, United States; 63 Uppsala Center for Computational Chemistry (UC_3_), Uppsala University, PO Box 576, SE-751 23 Uppsala. Sweden

## Abstract

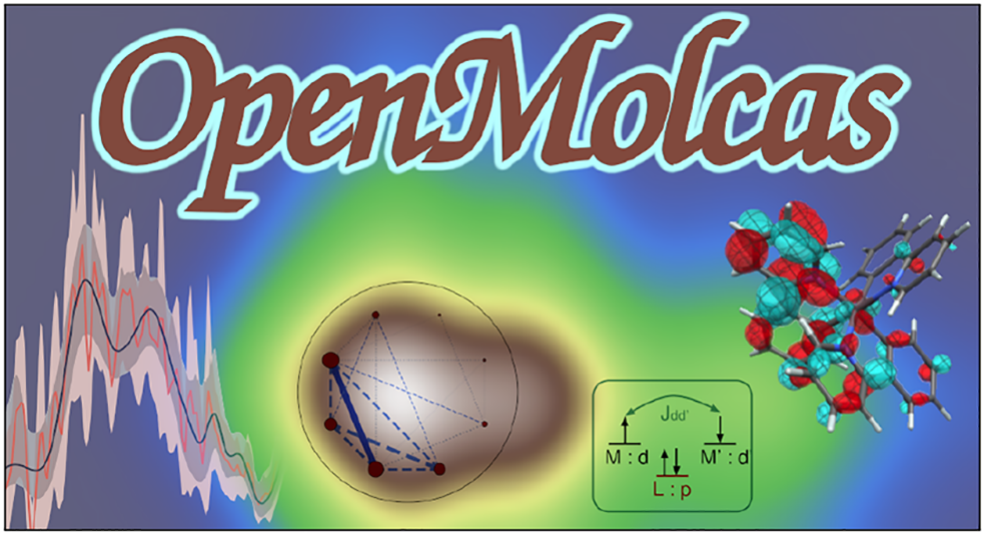

The developments of the open-source OpenMolcas chemistry software environment since spring 2020 are described, with a focus on novel functionalities accessible in the stable branch of the package or via interfaces with other packages. These developments span a wide range of topics in computational chemistry and are presented in thematic sections: electronic structure theory, electronic spectroscopy simulations, analytic gradients and molecular structure optimizations, ab initio molecular dynamics, and other new features. This report offers an overview of the chemical phenomena and processes OpenMolcas can address, while showing that OpenMolcas is an attractive platform for state-of-the-art atomistic computer simulations.

## 1 Introduction

The MOLCAS package has its origin in 1989 as a departmental development project in the Theoretical Chemistry group at Lund University, Sweden. The group around Prof. B. O. Roos developed the project, with a few invited scientists, keeping the project as a closed-source programming effort. The developments of the project in this time frame, 1989–2016, are documented in a number of publications.^1−7^ Starting with the 2018 release of the open-source program package OpenMolcas, under the Lesser General Public License (LGPL),^8^ further development has been a community effort. These efforts have already been the subject of additional publications.^9,10^

Here, a compilation of the continuing development efforts of the growing OpenMolcas community is put forward, as it progressed from early 2020 until late 2022. In this presentation, original developments and modifications to existing codes will be presented. These developments are manifested as modifications and additions to the core OpenMolcas open-source repository, or as associated open-source external utilities which are independent codes or interfaces. Collectively this creates the OpenMolcas*Web*, a manifestation of software developments in the open-source era. For convenience of the reader, these developments–which are many–have been sorted into six thematic sections to provide easier and more structured reading: “Electronic Structure Theory”, “Electronic Spectroscopy”, “Gradients and Molecular Structure Optimization”, “Vibrational and Vibrationally Corrected Electronic Spectroscopy”, “Ab Initio Molecular Dynamics”, and “Basis Sets, Ab Initio Model Potentials and Orbital Rotation”. Each of these sections consists of a multitude of independent contributions. For the sake of brevity of this introduction, each contribution is shortly described at the beginning of each section rather than here. The report ends with a summary. Sample input and output files, computational details, and further discussions are provided in the Supporting Information.

## 2 Electronic Structure Theory

OpenMolcas and the Molcas predecessor have historically been leading packages in multireference techniques to tackle strong and dynamic electron correlation effects. To continue this tradition, numerous updates involve advanced electronic structure approaches that allow large active space calculations able to address inherently multiconfigurational systems.

Examples of strongly correlated systems are offered by the class of exchange-coupled polynuclear transition metal clusters. Their low-energy states are generally multireference in character and very close in energy, resulting in fascinating properties, such as high-*T*_c_ superconductivity,^11,12^ magneto-electric coupling,^13^ and exotic magnetic orders.^14,15^ These low-energy states usually differ mainly in the spin-arrangements between the magnetic centers, rather than in changes in the charge density (charge-transfer excitations). Examples are transition-metal oxides with 3d open shells, such as cuprate superconductors, multiferroics as YMnO_3_ or RMn_2_O_5_ (R = Sm, Eu, Gd, Tb, Dy, Ho, Er, Tm, Bi) and Mott insulators such as the vanadium oxides. Magnetic excitation energy gaps range from a few meV to 100 meV and require specialized methods able to accurately account for static and dynamic correlation effects, as well as the relaxation that arises from the interaction between these two forms of correlation, often referred to as *screening effects*.

The development of novel multireference techniques in OpenMolcas beyond the established complete active space (CAS), such as restricted active space (RAS),^16^ generalized active space (GAS),^17^ selected configuration interaction (SCI),^18−32^ density matrix renormalization group (DMRG),^33−39^ and stochastic-MCSCF strategies based on full-CI quantum Monte Carlo (FCIQMC),^40−45^ has been driven by the exponential scaling of the dimension of multiconfigurational wave functions with the size of the active space. This exponential scaling usually limits the active space size to at most 18 electrons and 18 orbitals, CAS(18,18), in the absence of the above-mentioned advanced techniques.

These novel methods are available both within the OpenMolcas environment and also via interfaces to a number of satellite programs specialized in molecular electronic structure approaches, such as Block,^36^CheMPS2,^46^QCMaquis,^47^Dice,^25,26^GronOR,^48^ and NECI,^44^ offering possibilities to perform DMRG, selected-CI, nonorthogonal-CI and FCIQMC large active space calculations, and more.

In the latest OpenMolcas package, stochastic techniques have been substantially extended to include (1) spin-purification^49^ and spin-adaptation^50^ techniques, (2) the stochastic optimization of generalized active space wave functions (Stochastic-GASSCF),^51,52^ and (3) state-averaged Stochastic-MCSCF optimizations within the same and across different spin sectors. Selected-CI computations, in the form of the semistochastic heat-bath configuration interaction^26^ self-consistent field (S)HCI-SCF approach, are accessible via the recent interface to Dice.^25,26^ An interesting alternative to electronic structure characterizations, arising from the interface with GronOR, is offered by the nonorthogonal configuration interaction (NOCI) strategy. The QCMaquis module in latest OpenMolcas implements new DMRG-based methods for explicitly correlated and excited-state electronic structure calculations. Moreover, it integrates a new Python-based version of AutoCAS, which enables automating and streamlining CAS-based calculations.

Multireference configuration interaction (MRCI) methods represent a robust approach toward dynamic correlation effects, relying on a multiconfigurational, often but not limited to the CAS-type reference wave function. MRCI-based calculations in the latest OpenMolcas environment are made possible by a number of interfaces. Fully *uncontracted* MRCI calculations are now possible through the RelaxSE interface, with a specific focus on the accurate determination of magnetic excitations. Uncontracted MRCI techniques provide substantial support in accurately capturing screening effects (dynamical correlation), arising from the interaction of the magnetic centers with the bridging ligands. The OpenMolcas–RelaxSE interface complements the established OpenMolcas–COLUMBUS interface in performing MRCI computations. Additionally, the latest OpenMolcas–COLUMBUS interface provides the means for computing full nonadiabatic coupling vectors and spin–orbit interactions at the MRCI level of theory. Driven by the advances in OpenMolcas, the COLUMBUS package has also been released as an open-source project, and its interoperability with OpenMolcas has been enhanced.

The Stochastic-GASSCF^52^ that emerges from the OpenMolcas–NECI interface also allows stochastic-MRCI-like calculations, using SD bases, and with the possibility to spin-purify the targetted wave functions.

Multiconfigurational second-order perturbation theory (PT2) approaches, such as CASPT2 and RASPT2, represent another tool in OpenMolcas to tackle correlation effects involving electrons and orbitals outside the active space, i.e., external correlation. PT2 techniques have contributed greatly to the popularity of the OpenMolcas package over the years. In the latest OpenMolcas environment, new and robust quasi-degenerate variants of CASPT2 have been implemented; these combine the best features of MS-CASPT2 and XMS-CASPT2 in a single approach. These can be used for both calculating accurate relative energies and properly describing near-degenerate regions of the potential energy surface. A new scheme to eliminate the intruder state problem in CASPT2 has been implemented; it relies on an exponential regularization of the first-order amplitudes. The resulting σ^*p*^-CASPT2 approach is robust to intruder states and shows minimal dependence on the regularization parameter. An extension to the frozen natural orbital (FNO) CASPT2 has been developed, enabling its use with the more general RASSCF wave functions.

Multiconfiguration pair-density functional theory (MC-PDFT) is one of the latest techniques to be added to the OpenMolcas package; the goal of this method is to efficiently account for all correlation (both static and dynamic) by combining an MCSCF wave function with an on-top density functional. The method has been shown to often be as accurate as CASPT2, and sometimes it is more accurate. It relies solely on the one- and two-body reduced density matrices (RDM) and has a lower computational cost than CASPT2. The MC-PDFT implementation available in OpenMolcas has recently been extended to include new types of on-top functionals with improved performance for excitation energies, methodological extensions that account for spin–orbit coupling and quasi-degenerate state-interaction effects, and molecular dynamics interfaces. MC-PDFT calculations in OpenMolcas may be based on CASSCF, RASSCF, GASSCF (including SP), CASCI, DMRG or stochatic-MCSCF reference wave functions, and it is sometimes called CAS-PDFT, RAS-PDFT, GAS-PDFT, SP-PDFT, CASCI-PDFT, or DMRG-PDFT to indicate this.

Finally, within the latest OpenMolcas it is also possible to build and optimize *transcorrelated* (TC) Hamiltonians as a way to deal with dynamic correlation (mostly *cusp* correlation). This is possible both via the imaginary-time propagation extension of the TD-DMRG algorithm and via the TC-FCIQMC algorithm. In the latter, preoptimized (stochastic-)MCSCF wave functions may be used as a reference.

### 2.1 Stochastic Configuration Interaction Eigensolvers

Starting from the collaboration between Ali Alavi and Giovanni Li Manni in late 2014 that led to the first Stochastic-CASSCF^53^ in OpenMolcas, stochastic configuration interaction (CI) eigensolvers became key components of the OpenMolcas project.^9,44^ Relying on the stochastic optimization of the CI wave function,^40,41,43,44,54−61^ and the computationally inexpensive super-CI algorithm for the variational orbital optimization,^16,62−69^ larger Stochastic-CASSCF wave functions^53,70−73^ are now routinely optimized on modern parallel computer architectures.

Basis sets, point group symmetry, atomic orbital integrals (with and without resolution-of-identity Cholesky decomposition, RICD), molecular orbital transformations (AO/MO transformations and MO localizations), and active space selection are handled by OpenMolcas. From this information OpenMolcas produces a FCIDUMP(74) file, containing the one- and two-electron integrals in the basis of the active molecular orbitals. Using the FCIDUMP file, the configurational space is then generated and optimized stochastically within the NECI code. One- and two-electron RDMs are stochastically sampled after reaching stationary conditions^43,61^ and used by OpenMolcas for the super-CI orbital relaxation step. From the rotated orbitals a new FCIDUMP file is generated and used for a subsequent CI iteration. This iterative procedure is continued until the MCSCF energy has reached stationary conditions.^63−65^

The initial Stochastic-MCSCF implementation allowed only the optimization of complete active space wave functions, in the form of the Stochastic-CASSCF method, and was limited to electronic ground state wave functions expanded in Slater determinants (SDs). This choice of basis had the obvious limitation of preventing the optimization of low-spin states lying energetically above their high-spin counterparts.

Important recent work, now available in NECI and OpenMolcas, has allowed (1) performing GUGA spin-adapted^45,50^ and spin-purified^49^ Stochastic-CASSCF optimizations, (2) building and optimizing stochastic SD-based *restricted* and *generalized active space* wave functions (Stochastic-RASSCF and Stochastic-GASSCF^51,52^), and (3) performing stochastic optimization of excited state wave functions, in the form of state-specific (SS) or state-averaged (SA) stochastic-MCSCF procedures. Notably, it is now possible to perform SA-CASSCF calculations across states of different spin multiplicities. These developments allow the Stochastic-MCSCF tools to complement the corresponding conventional MCSCF procedures when larger active spaces are desired.

Many-body electron correlation effects beyond the active space, often referred to as *dynamic correlation effects*, can be accounted for by coupling Stochastic-MCSCF reference wave functions to the multiconfiguration pair-density functional theory (MC-PDFT)^75−77^ post-MCSCF procedure. The most important strength of the combined Stochastic-MCSCF/MC-PDFT approach is that MC-PDFT only requires one- and two-body RDMs, which are readily available from the preceding Stochastic-MCSCF optimization, while the more computationally demanding PT2 procedure requires higher-order density matrices, which are harder to sample stochastically.^78^ Moreover, MC-PDFT features a significantly reduced computational scaling compared to MR-PT2 approaches, as a function of the number of virtual orbitals. These differences make the Stochastic-MCSCF/MC-PDFT approach very appealing. For further details on the MC-PDFT approach, consult section 2.8.

Methods based on transcorrelated Hamiltonians (see section 2.4) are to be considered as alternative tools to tackle the dynamic correlation problem. In this respect, Stochastic-MCSCF wave functions can also be coupled to transcorrelated methods, via the OpenMolcas–NECI–CASINO interface.

**Figure 1:**
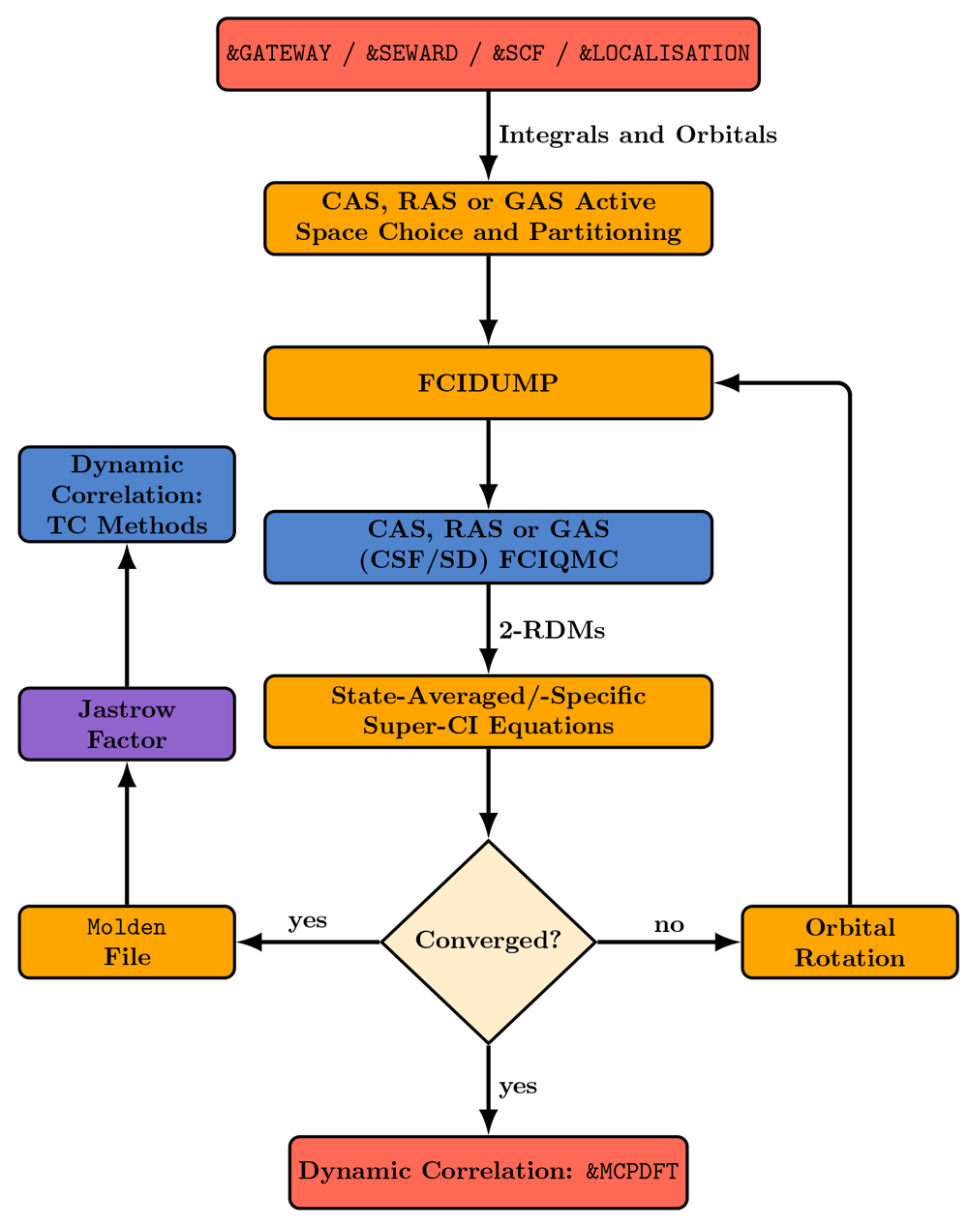
Flowchart illustrating the capabilities of the stochastic MCSCF interface. Colors are used to distinguish the different programs: NECI in blue, the RASSCF module of OpenMolcas in orange, other OpenMolcas modules in red, and CASINO in violet.

The flowchart in Figure 1 illustrates the Stochastic-MCSCF methods and their interface to post-MCSCF procedures, either within OpenMolcas or to external packages (CASINO).

An overview of the latest advances based on stochastic approaches follows.

#### GUGA Spin Adaptation in Stochastic-CASSCF†

Within a Slater determinant basis, without spin-purification methods^49^ (see below), the total spin quantum number is not guaranteed to be conserved during a simulation. This can lead to spin-contamination of the wave function, or convergence to a low-energy state whose spin multiplicity is higher than the targeted one. By directly working in a spin-adapted basis of configuration state functions (CSFs), the targeted total spin is guaranteed by construction. There are several ways to implement CI eigensolvers in spin-adapted bases.^79−86^ The graphical extension^87−90^ of the unitary group approach^91−93^ (GUGA), is one of the most popular techniques, which has already been adopted in the early days of the MOLCAS project within the RASSCF module, and in the more recent GASSCF approach.^17^ A GUGA-FCIQMC algorithm has recently been developed.^45^ Moreover, the GUGA formalism enables a new conceptual strategy for the sparsification of the CI problem: special unitary transformations of the molecular orbitals, in the form of localizations and reorderings, can produce extremely sparse and quasi-*block*-diagonal CI Hamiltonian matrices, and highly compressed eigenvectors, to the limit of dominantly single-reference wave functions.^73,94,95^ This strategy is extremely advantageous for methods that benefit from sparsity in the Hamiltonian and the corresponding eigenvectors, including GUGA-FCIQMC. Relying on GUGA-FCIQMC and the wave function compression strategy, it has been possible to study complex magnetic interactions in exchange-coupled polynuclear transition metal compounds,^72,73,94^ ferromagnetic domains in the hole-doped Hubbard model,^96,97^ and low-dimensional and cluster Heisenberg spin systems.^95,98^ The stochastic sampling of one- and two-body RDMs within the GUGA-FCIQMC code has allowed the implementation of the spin-adapted Stochastic-CASSCF method,^50^ that is available via the latest OpenMolcas–NECI interface. The spin-free one- and two-body RDMs, ρ_*ij*_ and Γ_*ij*,*kl*_, are stored in the OpenMolcas native DMAT, PSMAT, PAMAT format (see the Supporting Information of ref (50) for details).

#### Spin Purification in an SD Basis

Spin-adaptation techniques, such as the GUGA approach described above, have a high algorithmic complexity compared to SD-based CI eigensolvers, where the Slater–Condon rules allow fast excitation generation and inexpensive matrix element evaluation. In addition, the SD basis readily enables the computation of spin projection properties, such as spin polarization (magnetization) or the optimization of anisotropic Hamiltonians. Furthermore, many post-MCSCF methods and codes are developed on the basis of Slater determinants, such as the similarity-transformed FCIQMC.^99−101^ Other methods, such as internally contracted second-order perturbation theory, rely on higher-order density matrices that are available in SD-based FCIQMC but not in GUGA-FCIQMC.^78^ Thus, it is highly desirable to have a method for spin purification in an SD basis, which is now available via a first-order spin penalty strategy.^49^

In the first-order spin penalty approach, a modified Hamiltonian
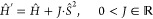
1is utilized, that can induce any system to be antiferromagnetically ordered. Since *Ĥ* and  commute, the eigenstates of  are still eigenstates of *Ĥ* and the eigenvalues of *Ĥ* can be directly calculated from the corresponding eigenvalues of  by subtracting *J* · *S*(*S* + 1). The idea of the first-order penalty applied to the CI-problem is widely known,^102,103^ and it has been shown that it works particularly well within the FCIQMC algorithm.^49^

Within spin-purified FCIQMC, the sampling of RDMs does not require any conceptual or algorithmic adaptation as compared to the conventional SD-based FCIQMC algorithm.^43,61,104^ The RDM entries of the spin-purified wave function can be fed back to OpenMolcas to calculate properties or to perform orbital relaxation within any of the Stochastic-MCSCF approaches. The choice of the spin-purification paramenter, *J*, is made within the NECI input, while in OpenMolcas the inputs are equivalent to the ones used for conventional SD-based Stochastic-MCSCF.

The first-order spin penalty has already been successfully applied to stochastic active space calculations to predict the electronic structure of chemically relevant systems featuring high-spin electronic ground states. In particular, the method has been utilized for the computation of the ^3^Σ_g_ – ^1^Δ_g_ spin gap of oxygen, where up to 16 electrons in 28 orbitals have been correlated, and for the study of the magnetic interactions on a trinuclear  cluster via a large CAS(55,38) calculation of the vertical Γ^(1/2)^ ←Γ^(3/2)^ transition.^49^

#### Stochastic-GASSCF

**Figure 2:**
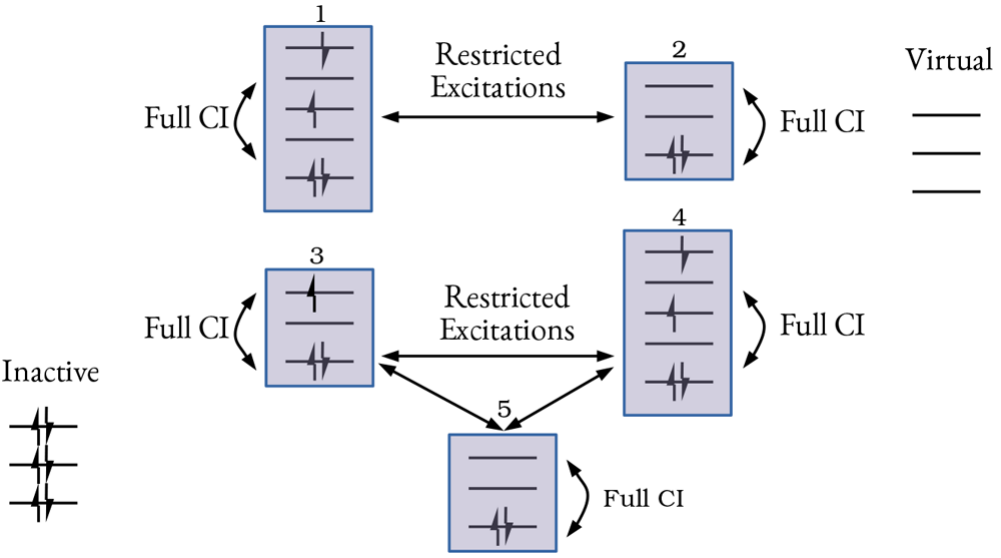
Pictorial representation of a GAS wave function with five GAS subspaces. GAS1 and GAS2 are connected to each other but disconnected from the rest. GAS3, GAS4, and GAS5 are also connected to each other but disconnected from GAS1 and GAS2.

The generalized active space (GAS) approach allows a flexible, controlled, and systematic way to build truncated CI wave functions that span a preselected portion of the corresponding CAS space. As for RAS, GAS-CI wave functions are preselected by the user. In GAS, the active orbitals are partitioned in a number of active subspaces. Within each subspace, a full-CI expansion is generated (complete set of intraspace excitations), while the number of interspace excitations is restricted.^17,51,105−107^ GAS spaces are defined *disconnected* if no interspace excitations are permitted, while they are defined *connected* if interspace excitations are allowed. Figure 2 depicts a possible GAS wave function.

The number of interspace excitations is limited by constraining the particle number per GAS space. If there are *k* GAS spaces and the particle number in the *i*-th GAS space is denoted with *x*_*i*_, the allowed particle distributions can be constrained by *local*,
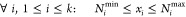
2or *cumulative* constraints,
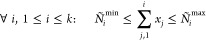
3The flexibility of GAS implies that other constrained wave functions like RAS are automatically available. Conventional GASSCF, that Ma and Li Manni implemented in 2011 and made available within the MOLCAS(7) and the OpenMolcas(9) chemistry software packages, only supports the cumulative GAS constraints.^17^ Another similar approach is the occupation restricted multiple active spaces self-consistent field (ORMAS-SCF) method, that only supports local constraints.^106^ The recent Stochastic-GAS algorithm allows both *local* and *cumulative* constraints, while allowing much larger active space sizes.

Special GASSCF wave functions, optimized by conventional techniques (Davidson) were combined with the post-MCSCF MC-PDFT approach and proved their utility for modeling transition metal chemistry; they are the separated pair^108^ and the extended separated pair^109^ approximations. One- and two-body RDMs can be stochastically sampled as for stochastic FCI or CAS wave functions.^43,53,61,104^ Those can be subsequently utilized to calculate orbital gradients, Hessians or within the super-CI theory^17,69^ to variationally relax the molecular orbitals. This gives rise to Stochastic-GASSCF and allows the calculations of other properties of interest from the RDMs.

Stochastic-GAS has been utilized in several test case applications.^51,52^ The application of Stochastic-GAS to a stack of benzene molecules, at varying intermolecular distances, illustrates the applicability of the method to fragment-based chemical systems. A very large Stochastic-GAS(96,159) calculation has been utilized for an Fe^II^–porphyrin model system, and demonstrates how the algorithm can be used to account for dynamic correlation effects. Stochastic-GASCI has also been utilized to investigate the low-energy spin ladder of an Fe_4_S_4_ cubane cluster, showing how the GAS strategy can be utilized to quantify the two competing spin-exchange and charge-transfer mechanisms stabilizing different spin-states.

#### (Spin-)State-Averaged MCSCF

In *state-averaged* (SA) MCSCF approaches, multiple electronic states are simultaneously optimized at the MCSCF level with the states sharing a common set of molecular orbitals. The orbitals are self-consistently optimized under the weight-averaged field of the electronic states considered. While not optimal for any of the individual states, these orbitals are the ones minimizing the weighted energy of the targeted states. Compared to state-specific calculations, this strategy ensures the orthogonality between the optimized roots by virtue of the common orbital set, which simplifies many post SA-MCSCF procedures.^110^ For an alternative approach based on the nonorthogonal CI strategy, see section 2.5.

Most commonly, the state-averaging procedure is used in excited state optimization in order to prevent variational collapse (in the context of “root-flipping”). Further applications arise when MCSCF states are used as references in response theory^111^ or MRCI binding curves.^112^ In the context of oligo-nuclear transition metal clusters, the state-averaging concept is commonly extended to average across different spin multiplicities (“spin-averaging”).^113−116^ The state-averaged procedure is very versatile when the states under consideration are not too different in character, and in general, under such conditions, it provides a balanced description of the electronic states investigated.

Due to historical design decisions, conventional state-averaged MCSCF in OpenMolcas used to be only possible within a single spin multiplicity. Stochastic-MCSCF has been extended to allow for state-averaged calculations across multiple spin symmetries. The spin- and state-averaging is available for SD based stochastic approaches (Stochastic-CAS, Stochastic-GAS, and spin-purified Stochastic-MCSCF) and the GUGA spin-adapted stochastic-MCSCF scheme. The same interface also accepts density matrices generated conventionally within OpenMolcas, thereby allowing for spin-averaging up to (18,18) active spaces without relying on any external software (see the *WRMA* keyword in the OpenMolcas documentation of the RASSCF module and the SI of this manuscript for more details).

### 2.2 Density Matrix Renormalization Group

The QCMaquis(47,117) interface allows a broad range of large active space calculations based on the DMRG theory.^33^QCMaquis relies on the so-called matrix product state (MPS)/matrix product operator (MPO) formulation of DMRG and leverages the generality of this framework to extend DMRG beyond ground-state calculations.^118^ In this respect, two extensions of QCMaquis are particularly relevant in the context of OpenMolcas. The first one concerns DMRG-based quantum dynamics simulations^119^ based on the so-called tangent-space time-dependent DMRG theory.^120^ The second one concerns explicitly correlated DMRG calculation based on the transcorrelated method (see section 2.4). In OpenMolcas it is also possible to combine DMRG with MCPDFT.^121,122^

#### Beyond Ground State Calculations

QCMaquis has been extended to support quantum-dynamics simulations with the time-dependent density matrix renormalization group (TD-DMRG) method.^123^ Specifically, it implements the so-called tangent-space TD-DMRG method.^120^ When applied to the nonrelativistic electronic Hamiltonian,^124^ TD-DMRG enables the simulation of nonequilibrium electronic processes for large orbital spaces. Applications of this method include the simulation of spectra via the correlation function formalism, the calculation of dynamical response properties beyond the linear-response approximation, and the design of attosecond time-resolved experiments.^124^ Although already competitive with alternative state-of-the-art algorithms, the TD-DMRG algorithm as currently implemented in QCMaquis can be largely improved. Its combination with orbital-optimization techniques, either based on quantum information theory^125^ or on self-consistent field algorithms,^126,127^ could enhance TD-DMRG efficiency by making it applicable to large, strongly correlated molecules. Work in this direction is in progress.

DMRG is inherently tailored toward ground-state calculations. QCMaquis can optimize excited states sequentially, in increasing energy order, by orthogonally constrained optimizations.^47^ However, such a procedure becomes unpractical for high-energy excited states due to its inherent sequential structure. Various more efficient excited-state DMRG variants have been developed in the QCMaquis framework in the past few years.^128^ Among them, the most promising one is DMRG[FEAST],^129^ a novel method that applies the FEAST algorithm^130^ on DMRG wave functions. DMRG[FEAST] can be straightforwardly applied to both electronic and vibrational structure calculations.^129^ DMRG[FEAST] overcomes the limitations of the excited-state DMRG variants that are based on orthogonally constrained optimizations by enabling a direct optimization of all excited states with energy lying in a given energy window.

#### Streamlining the Active Space Selection

An algorithm^131^ has been devised to automatically select active spaces based on single-orbital entropies^132^ obtained with a full-valence partially converged DMRG wave function.^133,134^ If the active space selection becomes too large for DMRG because of too many valence orbitals, the large-CAS protocol^135^ will partition the CAS into smaller subspaces. The single-orbitals entropies for each of these subspaces are then evaluated separately, and these results are combined to calculate approximate full-CAS single-orbital entropies. AutoCAS selects orbitals for active spaces by dividing strong and weak correlated orbitals based on these entropies, so that eventually a final CAS emerges for a fully converged DMRG or (depending on the resulting size) CASSCF calculation.^136^ Active spaces can be selected automatically for excited states by applying the AutoCAS selection protocol separately to each state. The generated active spaces are then unified to a consistent CAS, which suits the requirements of each state.^134^

**Figure 3:**
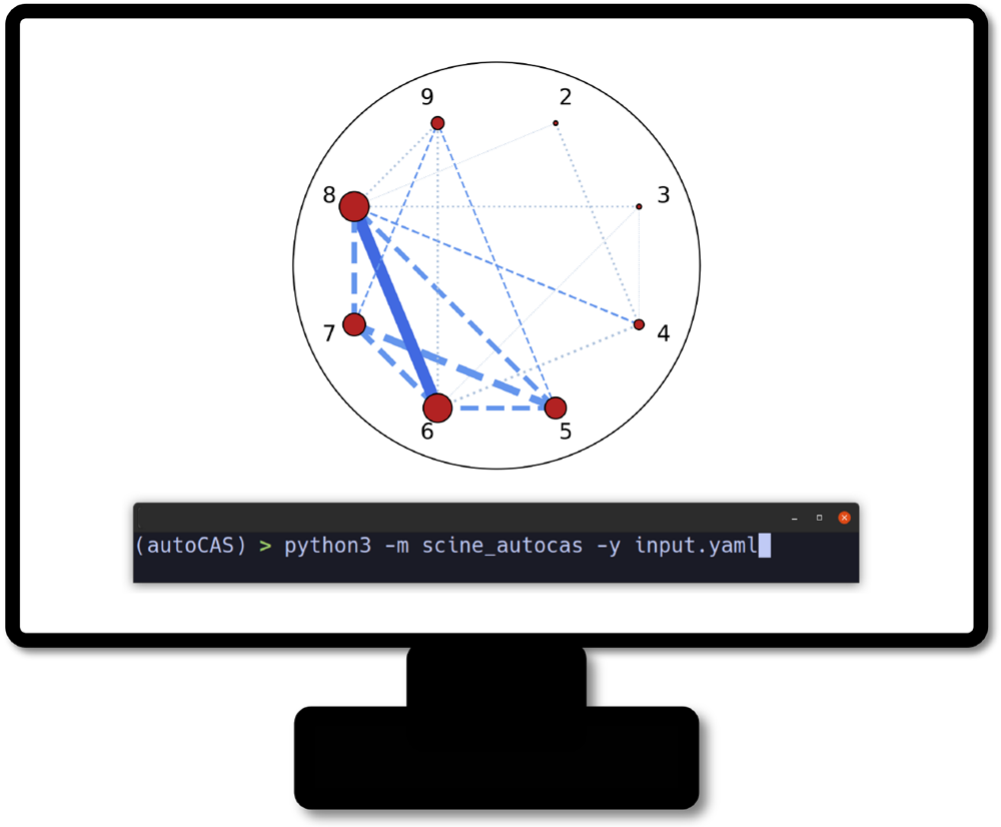
Schematic representation of the new CLI of AutoCAS. Displayed is the entanglement diagram for the NO radical, calculated based on the ANO-RCC-VDZ^139^ basis set and with a full-valence active space. For this example, AutoCAS suggests an active space that comprises orbitals 5–8.

The first AutoCAS version implementing this algorithm provides a graphical user interface (GUI), which is tailored to control OpenMolcas as its back-end for all quantum chemical calculations and QCMaquis as its DMRG solver. A Python 3 module^137,138^ has also been developed, which can control the automatic workflow either from a command line interface (CLI) or through a YAML input file (Figure 3). The new version of AutoCAS implements all features that were available in the previous version, so that automatic active space calculations can be executed on any hardware without the need of a GUI. Furthermore, the module is freely available and fully customizable to allow the creation of user-defined workflows.

### 2.3 The Heat-Bath Configuration Interaction Self-Consistent Field Method

**Figure 4:**
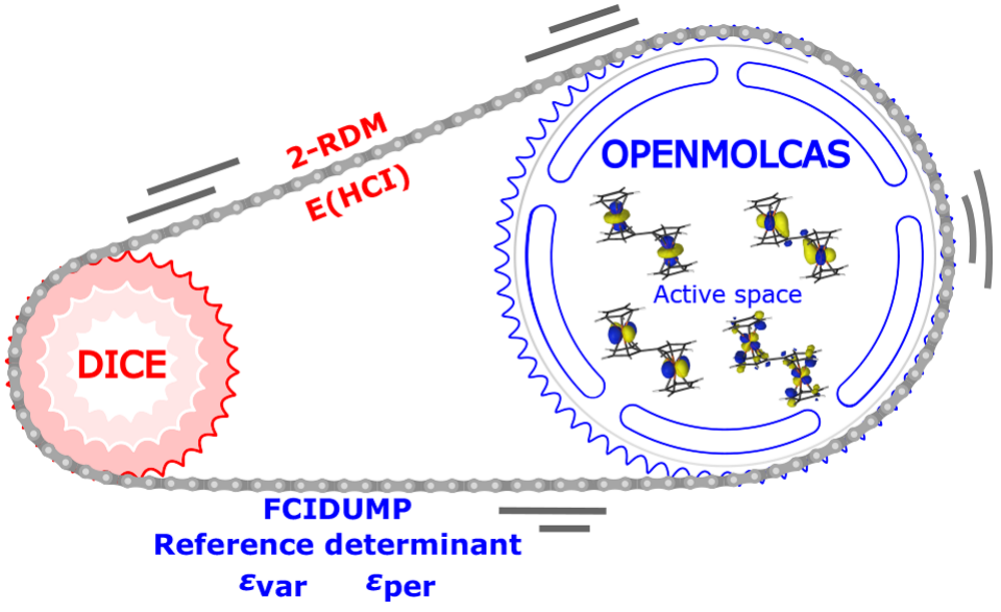
Schematic representation of an HCI-SCF calculation performed by the OpenMolcas–Dice interface. In each iteration, the RASSCF module produces an FCIDUMP file, required by Dice. The two-particle RDM calculated by Dice is then supplied to RASSCF, and the orbitals are optimized with the super-CI method. Besides standard keywords required by RASSCF, only one extra parameter is required, i.e., at least one starting (or reference) determinant. Two thresholds, ε_var._ and ε_per_, can be optionally specified. The default values of ε_var._ and ε_per_ are 10^–4^ *E*_h_ and 10^–5^ *E*_h_, respectively. For larger active spaces, one might aim for tighter thresholds.

The interface to Dice(25,26) allows the coupling of the heat-bath configuration interaction (HCI) method to the super-CI method for orbital optimization in the RASSCF module of OpenMolcas (Figure 4), thus, providing a self-consistent version of HCI, HCI-SCF. Similarly to many selected-CI methods, HCI employs a two-stage strategy: (1) a variational stage, in which only important determinants are selected iteratively; and (2) a deterministic perturbative stage, in which a second-order correction to the variational energy is included. Two corresponding thresholds are required: ε_var._ controls the number of determinants in the variational stage, and ε_per_ controls the approximation of the perturbative correction. For a detailed discussion of HCI, the reader is recommended to consult refs (25)–^27^.

**Figure 5:**
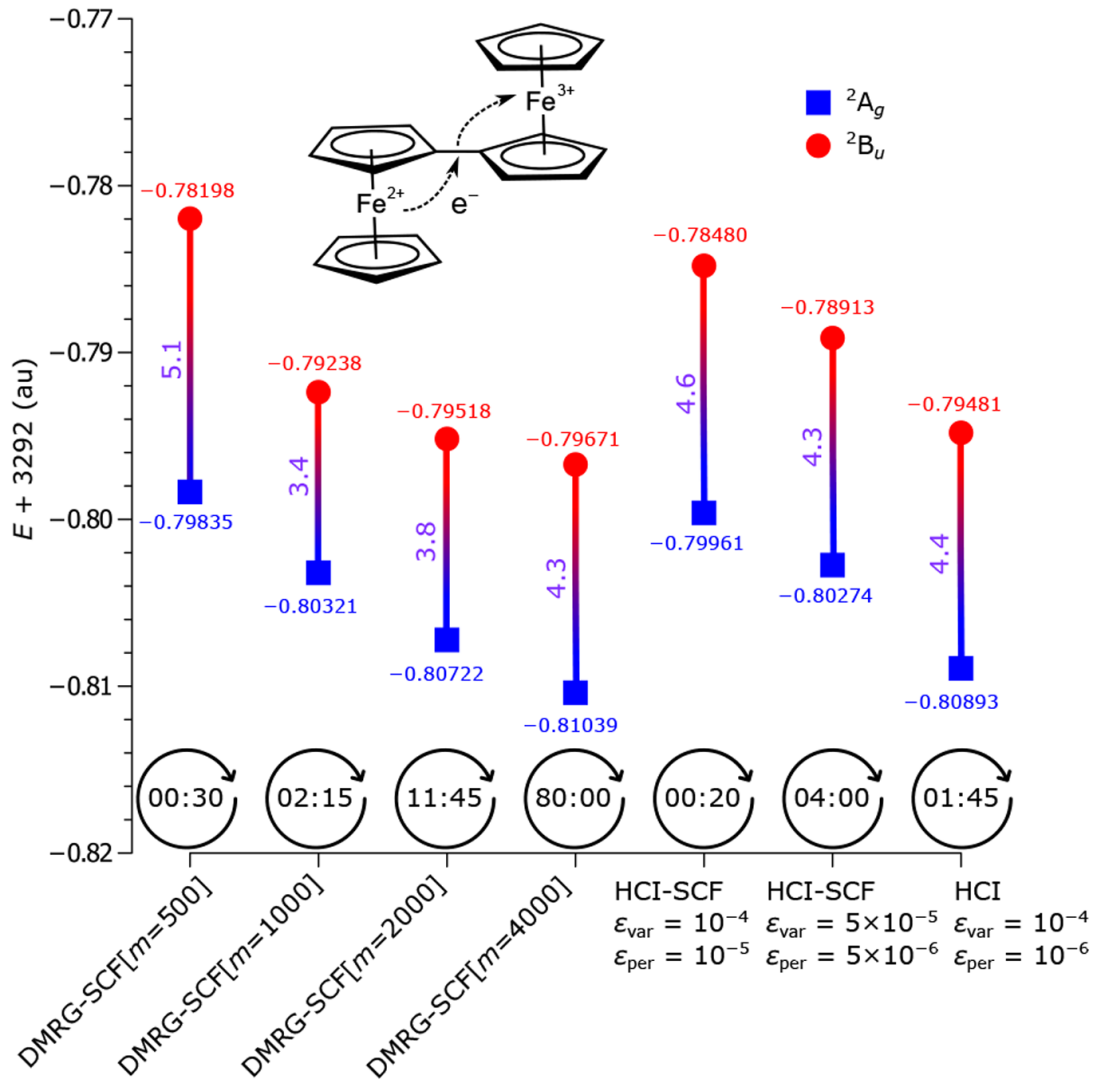
Total energies (in *E*_h_) of the ground state ^2^A_g_ and the excited state ^2^B_u_ in , calculated with DMRG-SCF(27,28) and HCI-SCF(27,28). The energy values have been shifted by adding 3292 *E*_h_ to the total energies. The electronic coupling *V*_ET_ values (in kcal mol^–1^) are in purple. The computing times (in hours) of the first SCF iteration are in black. The last HCI calculation is semistochastic: the perturbative component is calculated deterministically at ε = 10^–5^*E*_h_ and stochastically at ε = 10^–6^*E*_h_ ; the active space is taken from the cheapest HCI-SCF calculation.

To demonstrate the capabilities of the OpenMolcas–Dice interface, the intervalence electron transfer reaction between the Fe^2+^ and Fe^3+^ centers in the biferrocene cation  has been analyzed^140^ at this level of theory. In that, the electronic coupling, *V*_ET_, between the donor and acceptor centers has been calculated, which is equal to half of the energy gap between the ground state ^2^A_g_ and the excited state ^2^B_u_.^141^ Two large active spaces, CAS(27,28) and CAS(35,36), have been considered (details in the SI). The HCI-SCF and DMRG-SCF results with the CAS(27,28) active space (Figure 5) illustrate that HCI-SCF is comparable to DMRG-SCF in terms of accuracy and computational time.

### 2.4 Transcorrelated Methods

The singular nature of the Coulomb potential imposes the requirement that the solutions to the electronic Schrödinger equation exhibit cusps as two electrons or an electron and a nucleus coalesce.^142^ These features are qualitatively difficult to describe in quantum chemistry methods using basis functions depending on one electronic coordinate, causing the results to converge slowly with basis-set size. This can be avoided by introducing an explicit dependence on electron–electron and electron–nucleus distances in the wave function, for example via the Jastrow *ansatz*.^143^ In this *ansatz* the wave function, Ψ, is written as an antisymmetric part Φ, such as a CI expansion, multiplied by a Jastrow factor *e*^*J*^, where *J* is a real-space function of particle positions which contains optimizable parameters. The transcorrelated (TC) method of Boys and Handy^144,145^ then enables the use of this wave function in quantum chemical methods by absorbing the Jastrow factor into the Hamiltonian and using one’s method of choice to obtain Φ. Transcorrelation refers to a class of explicitly correlated electronic-structure methods that instead of modifying the wave function *ansatz*, as in F12-based methods, resolve the correlation factor in the Hamiltonian by similarity transformation. In fact, the TC Hamiltonian, *H̃*, is a similarity-transformed version of the original Hamiltonian, *Ĥ*,
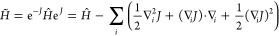
4The last term in eq 4 introduces three-electron terms into the TC Hamiltonian and renders the two-electron term non-Hermitian.

Many methods to solve the Schrödinger equation are unable to handle non-Hermitian Hamiltonians, but for projective approaches such as FCIQMC,^99−101^ imaginary-time time-dependent DMRG,^146,147^ coupled cluster,^148,149^ and quantum imaginary time evolution,^150,151^ this causes minor inconveniences at most. More recently, Liao et al. have also demonstrated a time-independent TC-DMRG algorithm capable of handling the non-Hermitian TC Hamiltonian.^152^ However, in this work, all TC-DMRG results have been obtained with the TD-DMRG method.

The presence of three-electron terms implies that  six-index matrix elements need to be calculated and stored, as opposed to the regular  scaling of four-index matrix elements, where *M* is the number of spatial orbitals in the basis set. This increase in scaling of the computational cost of the precomputation stage of the calculation and of the storage requirements can be, however, expected to be offset by the faster convergence with basis set size *M* of the TC method, which requires smaller values of *M* to reach a target accuracy thanks to its explicit description of electron–electron correlations.

It is also possible to generate a two-body formulation of the TC method by introducing a mean-field approximation over the three-body interactions.^148,149^ In this formulation, the necessary matrix elements storage scale as .

#### Transcorrelated FCIQMC

Transcorrelated FCIQMC calculations can be performed with OpenMolcas through its interface with NECI.^44^ Given a set of orbitals stored as a molden file, one can use CASINO(153) to optimize a tailored Jastrow factor,^154−156^ from which the TC-FCIQMC calculation can then be performed using NECI in combination with the TCHInt library.^157^

**Figure 6:**
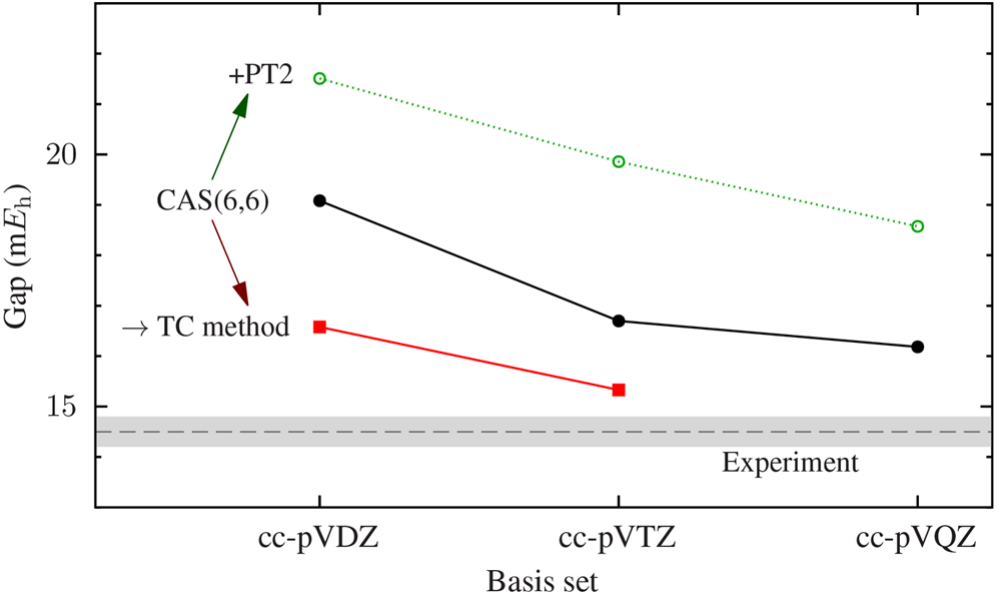
Spin gap of the CH_2_ molecule between the singlet ^1^A_1_ ground state and the triplet ^3^B_1_ excited state using the cc-pV*x*Z basis-set family. The TC-FCIQMC method using CASSCF(6,6) orbitals yields a spin gap within 1 m*E*_h_ of the experimental value, already at the cc-pVTZ basis-set level.

As a demonstration of the application of the TC-FCIQMC method, in Figure 6 the ^1^A_1_ →^3^B_1_ spin gap of the CH_2_ molecule is plotted, using CASSCF(6,6) and CASSCF(6,6)/PT2 results, TC-FCIQMC energetics, based on the CASSCF(6,6) reference molecular orbitals, and the experimental value^158^ for comparison. The dynamic correlation introduced by the TC-FCIQMC method significantly improves the quality of the CASSCF(6,6) spin gaps, yielding accurate results with small basis sets. Perturbative corrections, in the form of the CASSCF(6,6)/PT2, worsen the CASSCF(6,6) spin gap predictions.

#### Transcorrelated DMRG

**Figure 7:**
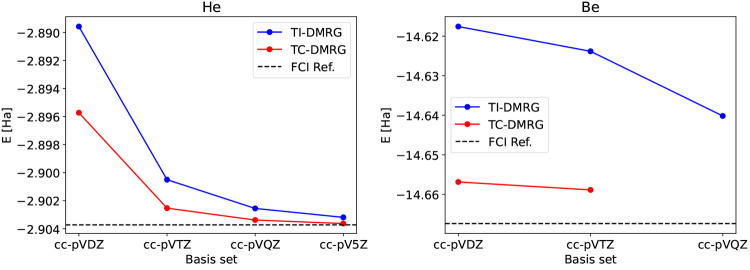
Comparison of the energies of the He (left panel) and Be (right panel) atoms obtained with conventional TI-DMRG (blue lines and dots) and with tcDMRG (red lines and dots) with correlation-consistent basis sets of varying size. The TI-DMRG and tcDMRG energies, as well as the reference energies, were taken from ref (147). The parameters for the TI-DMRG and tcDMRG calculations can be found in ref (147) as well. Note that the tcDMRG energy of Be with the cc-pVQZ is not reported in the graph due to exceeding memory requirements for the corresponding tcDMRG calculation.

Transcorrelated DMRG (referred to as tcDMRG, for consistency with previous works^146^) is available through QCMaquis. The tcDMRG method^146,147^ exploits the idea that an imaginary-time TD-DMRG (see section 2.2) can be used for ground-state optimization and straightforwardly applied to non-Hermitian operators. The ground state of the non-Hermitian, three-body Hamiltonian can be optimized with imaginary-time TD-DMRG – which defines the tcDMRG scheme.^146,147^ As with any other transcorrelated method, tcDMRG converges faster to the complete basis set limit compared to time independent (TI) DMRG, as shown in Figure 7 for the He and Be atoms.

Additionally, by reducing the extent of correlation effects in the many-body wave functions, transcorrelation enables encoding the full-CI wave function as a more compact MPS. For this reason, as shown in ref (146), tcDMRG converges faster with the bond dimension *m* compared to conventional DMRG.

### 2.5 Nonorthogonal Configuration Interaction

In multiroot calculations, when the character of the electronic states optimized at the SA-CASSCF level differs significantly, for example when considering charge transfer states, the SA procedure can lead to poor prediction of their electronic structure. In these cases, the optimal set of active orbitals for each of the electronic states differ, i.e., orbital relaxation effects vary significantly for the states investigated. Therefore, using a unique set of orbitals for all electronic states, as done in SA-CASSCF, represents an important limitation.

This problem can be circumvented by enlarging the active space, but unfortunately this comes with important, and often prohibitive computational costs, related to the exponential scaling of the size of the many-body wave function with the size of the active space.

The nonorthogonal configuration interaction (NOCI) approach offers an interesting alternative to the increase of the active space within the SA-CASSCF framework. NOCI combines a rigorous, ab initio description of the electronic state of interest without losing the intuitive interpretation of the outcomes, characteristic of simple phenomenological model descriptions. NOCI calculations are made possible by the GronOR interface.^48^GronOR is massively parallel, GPU-accelerated, and capable of performing calculations on systems with up to 200 atoms.

NOCI expresses each electronic state in its own optimal set of orbitals to rigorously include orbital relaxation effects. Consequently, electronic states are no longer orthogonal and NOCI calculations are required to obtain the eigensolutions. Apart from taking into account the orbital relaxation, one important advantage of using NOCI is the fact that the NOCI wave functions are generally very compact, typically counting less than ten terms, each being one of the nonorthogonal (diabatic) electronic states that were used as basis for the NOCI. This makes the interpretation of the results very straightforward.

The implementation of NOCI in GronOR is focused on the use of ensembles of molecules to study intermolecular processes, although the program can also be used to study intramolecular processes such as charge transfer in donor–acceptor molecules. The implementation for ensembles is labeled NOCI for fragments (NOCI-F) and starts with the generation of the fragment states. These are typically the ground state and some excited states, but can also include cationic and anionic states. Once this is done for all fragments/molecules of the ensemble, the many-electron basis functions (MEBFs) for the NOCI are constructed as antisymmetric spin-adapted products of the different fragment states. For example, combining cationic and anionic states of different fragments, one obtains the diabatic representation of a charge transfer state of the system with full orbital relaxation. In general, these MEBFs represent an optimal descriptions of the diabatic electronic states of the ensemble. The NOCI-F fragment wave functions can be generated with any wave function based approach as long as the wave function can be expressed as an expansion of Slater determinants. Typically, one uses CASSCF wave functions, but other multiconfigurational wave functions are equally valid. Note that when using antisymmetrized products of XASSCF (X = C,R,G) fragment wave functions to generate the MEBFs, the orbital sets of different MEBFs are mutually nonorthogonal, and the various fragment orbital sets within one MEBF are mutually nonorthogonal. In addition, for applications with one single *X*ASSCF wave function in each MEBF (that is, when only one fragment is considered), the method is similar to RASSI, except that NOCI does not require that the active spaces of the different MEBFs be the same.

Lifting the restriction of orthogonality between molecular orbitals increases dramatically the complexity of the calculation of the matrix elements between Slater determinants, preventing a more widespread use of NOCI for many years. However, the increasing computer power, the development of efficient algorithms and powerful parallel implementation has paved the way for renewed interest in these approaches; in particular in GronOR (1) individual matrix elements are efficiently calculated through the factorization of the transformed second-order cofactors,^159^ and all the determinant pairs contributing to the few MEBFs matrix elements are calculated independently and in parallel, (2) the atomic orbital integrals are transformed into a common set of orthogonal molecular orbitals (shared by all NOCI states), by diagonalizing the overlap matrix of the MOs of all states and removing the linear dependencies,^160^ and (3) a threshold to filter out small weighted contributions from determinant pair to their respective MEBF matrix element has been introduced, that does not affect significantly the relative energies of the different NOCI states.^48,161^

**Figure 8:**
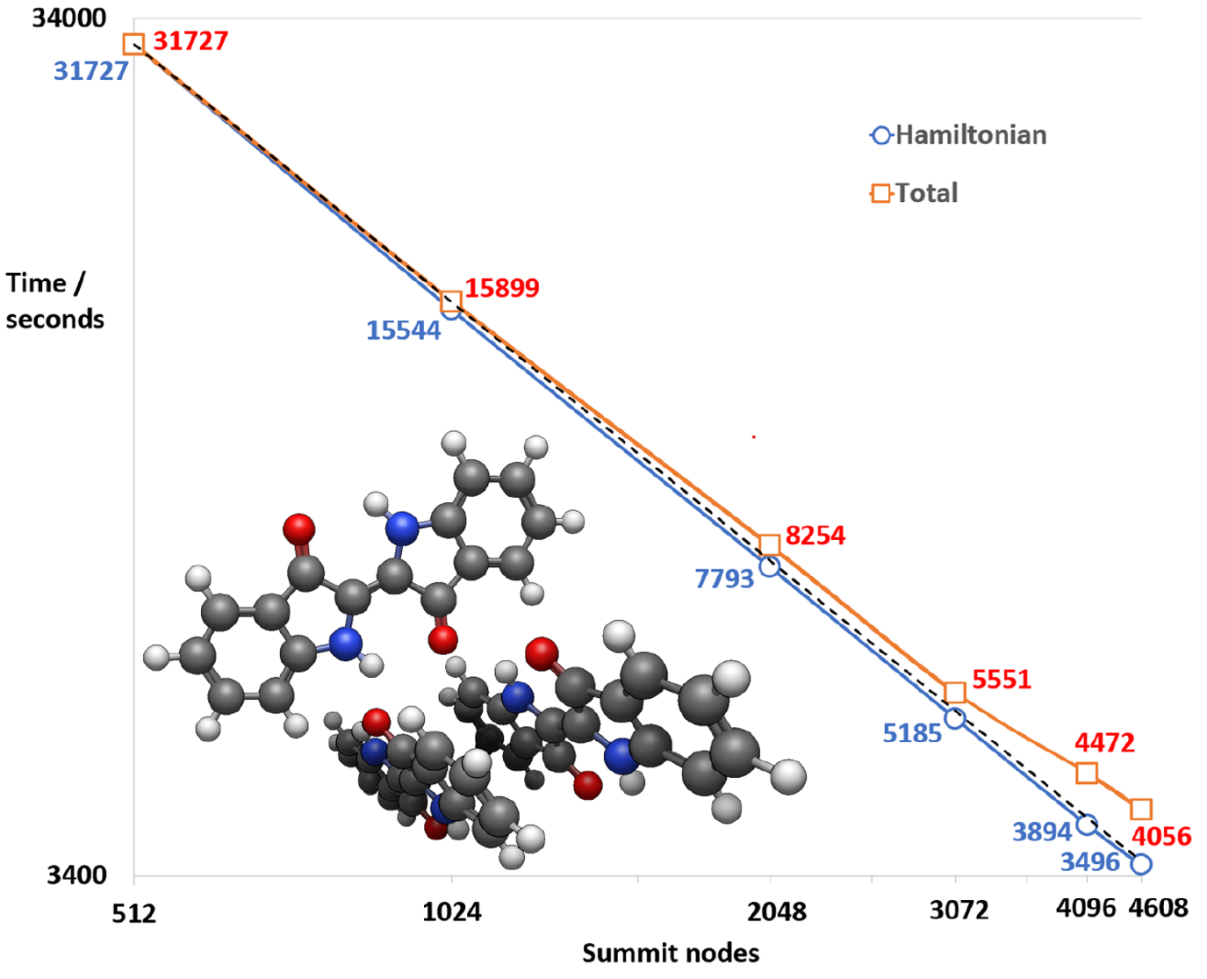
Parallel scalability on OLCF’s Summit supercomputer of a GronOR 4 × 4 Hamiltonian calculation for a trimer of indigo molecules taken from the crystal structure. Shown are for 512, 1024, 2048, 3073, 4096, and 4608 Summit nodes the wall clock times in seconds for the computation of the Hamiltonian matrix elements (blue) and the total elapsed time (red) which includes the setup time for distribution of the integrals and the construction of the MEBFs. The dotted line indicates ideal scaling.

An illustration of the parallel scalability of GronOR is given in Figure 8 for a trimer of indigo molecules. This is one of the largest systems calculated to date, with 90 atoms and 408 electrons. Total wall clock times and times for computation of the matrix elements only are given for the calculation of a 4 × 4 Hamiltonian matrix for the spin states S_0_S_0_S_0_, S_0_S_0_S_1_, S_0_S_1_S_0_, and S_1_S_0_S_0_. The computation of the matrix elements scales linearly, i.e., ideally from 512 to a full machine run on 4608 nodes on the Summit supercomputer at Oak Ridge Leadership Computing Facility (OLCF), with six ranks per node. At larger node counts, the reading from file and distribution of the 50 GB of two-electron integrals becomes a discernible fraction of the total time, as indicated by the difference between the blue and red curves. Nevertheless, the parallel scalability on 3072 nodes is 95%, and on the full machine run on 4608 nodes is still 87%. This example benchmark system demonstrates the ability of GronOR to effectively tackle molecular clusters of significant size.

GronOR uses the one- and two-electron integrals from OpenMolcas and a list of CI coefficients plus orbital occupations of the multideterminantal wave functions that are used to construct the MEBFs of the NOCI matrix. GronOR works with Slater determinants and therefore makes use of the *PRSD* keyword of the RASSCF module to write out the wave function in terms of determinants instead of configuration state functions. The list of coefficients and orbital occupations is saved in a separate file that is directly read by GronOR. The transformation of one- and two-electron integrals to a common MO basis is performed by the MOTRA module, in which the automatic orthogonalization of the orbitals has to be deactivated using the *NOORth* keyword. If the Cholesky decomposition of the integrals is used, MOTRA transforms the Cholesky vectors, and the auxiliary program rdcho reconstructs the two-electron integrals in the common MO basis. Subsequently, the TRAINT and TRAONE files are written in a format that can be read by GronOR with the auxiliary program rdtraint. Both auxiliary programs are part of the GronOR repository on GitLab.^162^

### 2.6 Multireference Configuration Interaction

The MRCI capabilities of OpenMolcas are expanded with interfaces to COLUMBUS and RelaxSE, allowing calculations of nonadiabatic and spin–orbit couplings, and the use of uncontracted variants specialized in the treatment of magnetic excitations.

#### Spin–Orbit and Nonadiabatic Interactions at the MRCI Level

COLUMBUS is a collection of programs for high-level ab initio molecular electronic structure calculations.^163,164^COLUMBUS is distinguished by its flexible and powerful uncontracted MRCI functionality.^165,166^ Using the parallel implementation, it is routinely possible to solve CI problems with more than a billion configurations.^167,168^ Aside from MRCI energies, COLUMBUS also features efficient functionalities to compute MRCI gradients and nonadiabatic couplings (NACs).^169^ Finally, there is an efficient two-component spin–orbit MRCI^170^ functionality with a more recent extension to perturbative spin–orbit couplings (SOCs).^171^

An interface between COLUMBUS and OpenMolcas is available on various levels. In its most common operation, OpenMolcas serves as the integral engine used by COLUMBUS. This mode is particularly popular because it provides the possibility to perform spin–orbit MRCI within an all-electron computation. Furthermore, using the perturbative SOC code, it is possible to compute SOCs, gradients and, since recently, also the full NACs, all at the MRCI level. This mode is popular for nonadiabatic dynamics and the parametrization of vibronic coupling models (more details below).^172^ Additional options for connecting OpenMolcas and COLUMBUS exist, for example at the level of the MO coefficients. These are discussed in more detail in ref (7).

Following the lead of OpenMolcas, COLUMBUS has recently undergone an open-source release. Considering that both packages are openly available facilitates the maintenance of a stable interface. Rather than writing interfaces for individual versions, it is possible to interface to one specific snapshot on the Git repository to provide a well-defined version.

Energies, energy gradients, nonadiabatic couplings (NACs), and SOCs are pivotal elements in the investigation of the competition between internal conversion (IC) and intersystem crossing (ISC). These electronic structure data are best calculated with correlated multireference methods, such as MRCI or MS-CASPT2, which can provide a balanced description of all regions of the potential energy surfaces.^173^ Nonadiabatic nuclear dynamics can be carried out (1) with electronic structure data obtained on-the-fly, or (2) from analytic model functions. The on-the-fly approach, which usually uses relatively modest electronic wave functions, is easily implemented in the adiabatic representation, which however is singular at conical intersections requiring particular care in the numerical propagation procedure.^174^ The analytic model function approach, which can use sophisticated electronic wave functions, including large MRCI, obtains a smooth and continuous description by using a predetermined diabatic representation of the coupled potential energy surfaces given as a diabatic potential energy matrix (DPEM), usually obtained from a complex fitting procedure. Due to the nonuniqueness of a diabatic representation,^175−177^ there are a variety of methods of diabatization. According to the type of information used, diabatizations can be grouped into several categories: derivative-based methods,^178−181^ property-based methods,^141,182^ methods based on electronic wave functions,^183,184^ and diabatization by *ansatz*.^185,186^ For the most recent developments in diabatization schemes, the reader is referred to ref (187).

Because COLUMBUS can provide analytical derivative couplings at the highest MRCI levels, the following discussion focuses on derivative-based methods. These methods directly use the derivative couplings to diabatize electronic states. The residual derivative couplings can be determined and used to assess the quality of the diabatization. Existing derivative-based methods include (1) solution of the Poisson equation,^178,179^ (2) the Shepard interpolation,^180^ (3) line integral methods.^181^ Zhu and Yarkony proposed a simultaneous Fitting-and-Diabatizing (FaD) method (subsequently extended to include NN), in which the DPEM is expressed with symmetrized functional form. Ab initio electronic structure data including energies, energy gradients, and derivative couplings are simultaneously fit and diabatized to generate a robust quasi-diabatic representation.^188^ The diabatization is performed among states with the same spin multiplicity, thus the resultant DPEM provides an analytical tool for describing internal conversion.

With the interface between COLUMBUS and OpenMolcas being available, it is also possible to compute SOCs at the same MRCI level as above, thus enabling a complete description of both IC and ISC using the same wave functions. The SOCs are initially evaluated in the adiabatic representation. As with the Coulomb Hamiltonian, they have to be transformed into the diabatic representation to gain a smooth and continuous functional form. Considering a system in which IC and ISC are both possible, for each group of states with the same spin multiplicity, a distinct diabatization is performed. Based on the diabatizations, the SOCs between states with different spin multiplicities can then be diabatized giving rise to a complete diabatic representation for both IC and ISC.^189^ And last, but not least, within the GUGA formulation of MRCI in COLUMBUS it is possible to derive spin-densities from the spin-free reduced density matrices.^190^

#### Uncontracted MRCI for Magnetic Interactions

The interface to RelaxSE(191) gives access to fully uncontracted MR-SCI, MR-SDCI, and methodological extensions specifically designed to tackle the problem of magnetic excitations, such as the difference dedicated CI (DDCI)^192,193^ approach or selected active space plus single-excitation CI (SAS+S).^194^ It is also designed to ensure *S*^2^ eigenstates. RelaxSE runs after a minimal CASSCF/RASSCF + MOTRA calculation, providing reference orbitals and associated integral files. It is available under LGPL license.^195^

**Figure 9:**
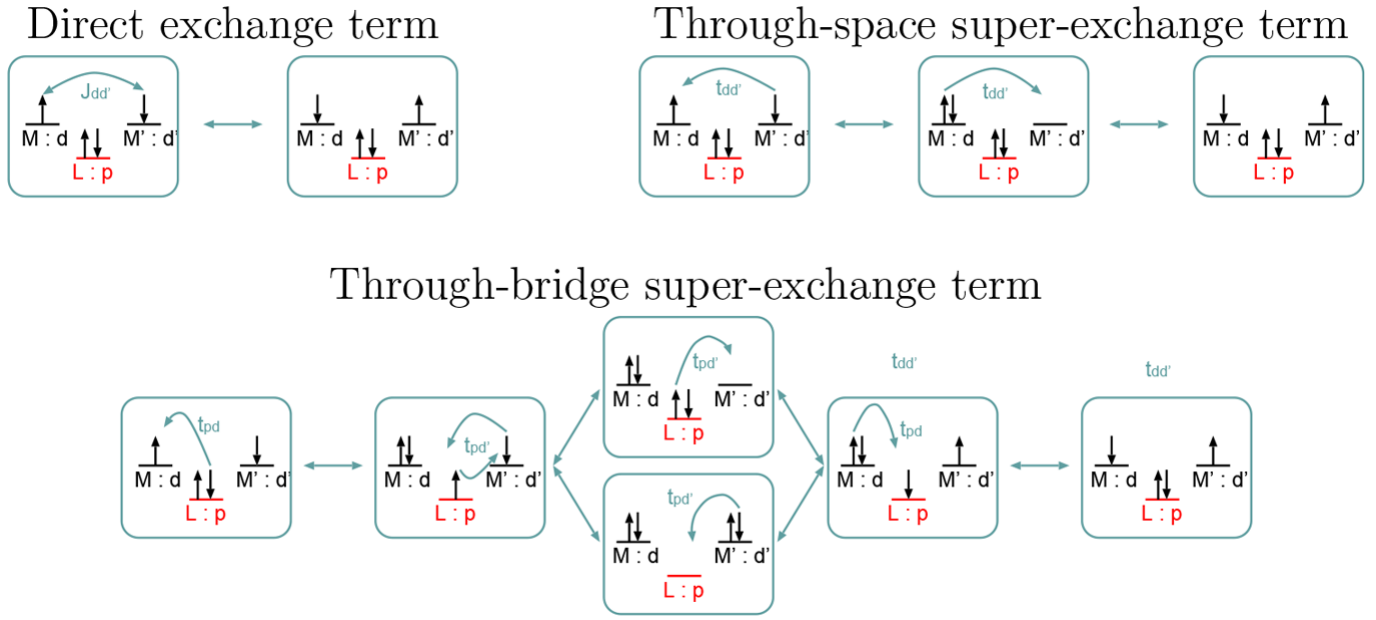
Scheme of the different terms in a ligand-bridge magnetic exchange.

RelaxSE can use either CAS reference wave functions, or a set of selected configurations within the CAS. Ligand-to-metal and/or metal-to-ligand configurations can further be added for building a more complete reference wave function. The flexibility in designing ad hoc reference wave functions within the RelaxSE framework is especially important, if one considers that *effective* spin-exchange interactions are the result of direct-exchange, through-space superexchange, and through-bridge superexchange interactions (see Figure 9).

The CAS+DDCI method has proven to be very efficient to accurately evaluate magnetic excitations when the number of open shells per magnetic center remains small (1 or 2). One can cite for instance the prediction within experimental accuracy of the magnetic exchange integrals in the cuprate superconductors parent compound La_2_CuO_4_:^196^ the first-neighbor exchange integral was predicted to be *J* = −124 meV while it was experimentally evaluated to *J* = −128(6) meV^197^ and *J* = −134(5) meV,^198^ and the second-neighbor exchange was predicted to be *J*′ = −6.5 meV while derived from Raman experiments to be |*J*′| < 9 meV.^199^ When the number of magnetic orbitals per magnetic center is larger than two, then one has to switch to the SAS+S method.^194^ Its accuracy can be pictured on the exchange integrals of the multiferroic YMnO_3_ compound, where the magnetic integrals are predicted to be *J*_1_ = −3.19 meV and *J*_2_ = −3.41 meV, while the average of the latter was fitted from inelastic neutron scattering to *J*_av_ = −3.0 meV^200^ and *J*_av_ = −2.3 meV.^201^

### 2.7 Multiconfigurational Perturbation Theory

The CASPT2 method is one of the most well-known multireference perturbation theories (MRPTs). The full CASPT2 method, also known as CASPT2-N, was first implemented in MOLCAS in 1992.^202^ Since then, it has become one of the main assets of the OpenMolcas package. The following describes some recent improvements to the method and implementation, allowing more robust treatment of near-degeneracies, removal of the “intruder state problem” and extension to the restricted active space (RASPT2) variant.

#### New Quasi-Degenerate Variants of CASPT2

Two new quasi-degenerate variants of CASPT2, namely, extended dynamically weighted CASPT2 (XDW-CASPT2)^203^ and rotated multistate CASPT2 (RMS-CASPT2),^204^ have been recently developed with the aim of maintaining the typical accuracy of multistate (MS) CASPT2^205^ for relative energies, while ensuring smooth potential energy surfaces (PES) throughout conformational space, akin to extended multistate CASPT2 (XMS-CASPT2).^206^ The key steps underlying these new variants are two: (1) the (input) CASSCF wave functions are initially rotated such that they diagonalize the state-averaged Fock operator; and (2) the Hamiltonian is partitioned for each model state separately, using state-specific Fock operators constructed with a dynamic weighting scheme and the rotated CASSCF wave functions. The dynamic weighting scheme depends on the interaction strength between the model states, denoted ξ_*αβ*_, and is quantified by either relative energies, Hamiltonian coupling elements, or a combination thereof. This results in a methodology, XDW-CASPT2, that effectively interpolates between MS-CASPT2 and XMS-CASPT2, and varies between state-specific and state-averaged regimes depending on the molecular geometry. The sharpness of the transition between these two regimes is controlled through an empirical parameter ζ, which is given as an input to the calculation. In the special case where ζ → *∞*, the dynamic weighting scheme is suppressed and the Fock operators remain purely state-specific regardless of the molecular geometry. In this limit, the approach becomes parameter-free, and is called RMS-CASPT2; as opposed to the other limit, ζ → 0, that is equivalent to XMS-CASPT2.

**Figure 10:**
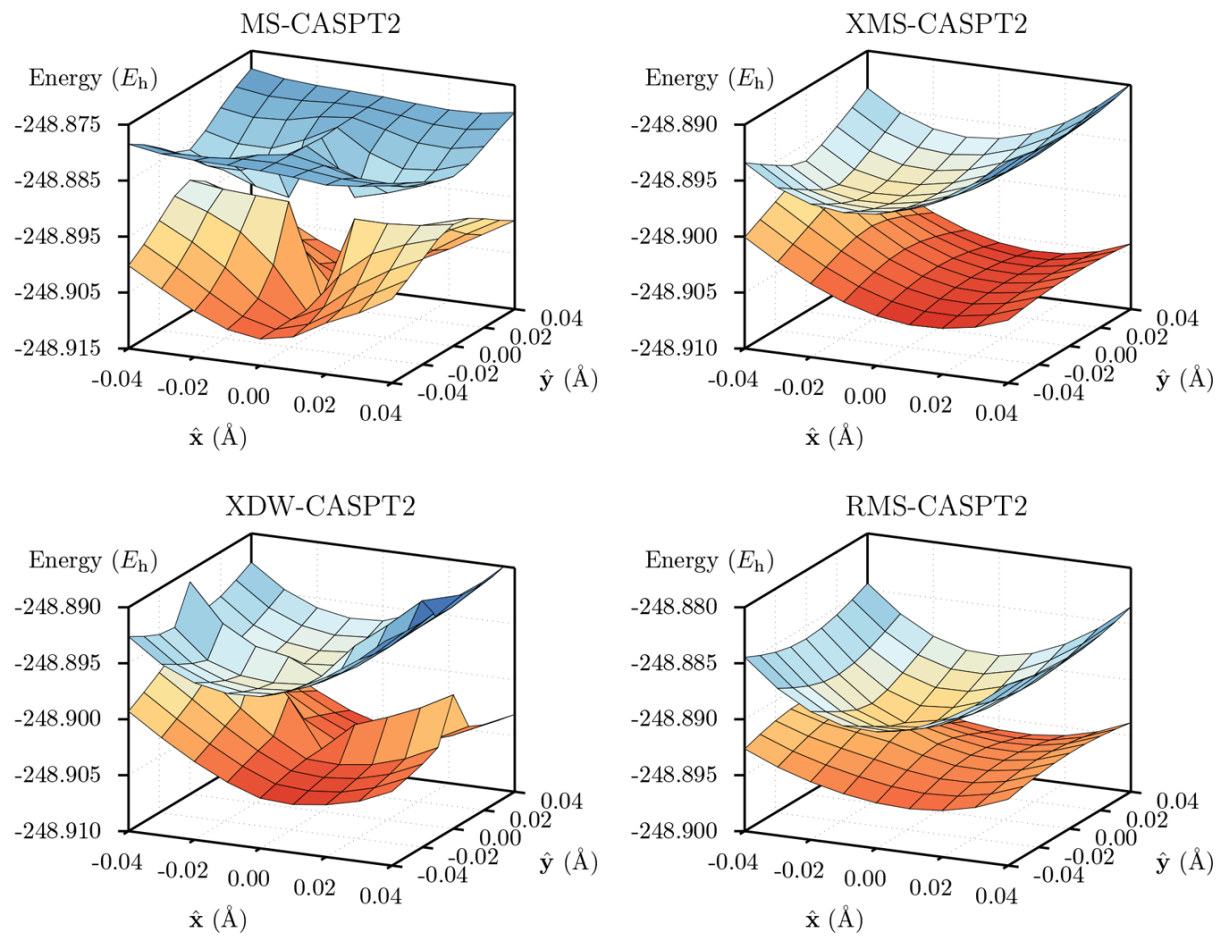
Potential energy surfaces of the branching space of PSB-3 computed using different quasidegenerate variants of CASPT2. Note that the origin of the plots corresponds to the SA-CASSCF CI geometry.

To assess the accuracy of the two new methods with respect to relative energies, a series of vertical singlet transitions were calculated in a number of small organic molecules taken from Schreiber et al.’s benchmark set,^207^ and compared to those obtained with MS-CASPT2.^203^ Both XDW- and RMS-CASPT2 perform very similar to MS-CASPT2, with mean absolute deviations of 0.02 and 0.01 eV, respectively. In contrast, XMS-CASPT2 deviates by 0.12 eV on average. The robustness of the new approach with respect to discontinuities on the PES was investigated in several systems, encompassing the avoided crossing of lithium fluoride, and the conical intersections in allene, ethene and the protonated Schiff base 3 (PSB-3) cation.^203,204^ In all cases, the potential energy surfaces obtained with RMS-CASPT2 are smooth at all molecular geometries considered, in particular at points of near-degeneracy (avoided crossings and conical intersections). The same is in general true for XDW-CASPT2 as well, even though for certain combinations of the input parameter ζ and the expression used for the interaction strength ξ_*αβ*_, the PES may show unphysical wiggles when the underlying CASSCF states change very rapidly as a function of the molecular geometry. A comparison of the branching space of the S_0_/S_1_ conical intersection of PSB-3 obtained with MS-, XMS-, RMS-, and XDW-CASPT2 is shown in Figure 10.

Overall, from the investigations carried out, it can be concluded that RMS-CASPT2 constitutes a solid choice for both the calculation of excitation energies and the exploration of potential energy surfaces, at essentially the same computational complexity of the more famous MS-CASPT2 and XMS-CASPT2 variants.

#### Regularized CASPT2

A recurring issue in second-order perturbation theory is the divergence of the energy expansion due to small zeroth-order energy denominators. This is the case also in CASPT2, where this so-called *intruder state problem* (ISP) is typically dealt with by a real or imaginary level shift that prevents the denominators to vanish.^208,209^ These shifts—in particular the imaginary shift—are effective in removing the diverging terms of the series, however, they also affect all the other ones which would otherwise require no modification. This sometimes results in a significant dependence of the results on the value of the level shift. An alternative approach, inspired by the recent work in regularized orbital-optimized MP2 by Lee and Head-Gordon,^210^ is to use σ^*p*^-regularization as a way of removing the intruder states. The resulting methodology, σ^*p*^-CASPT2, relies on an energy-dependent exponential factor, which either damps the first-order amplitudes associated with vanishing denominators (*p* = 1) or completely suppresses them (*p* = 2). It is noted in passing that the expression for the latter case has also been reported by Evangelista.^211^ These regularization schemes were recently implemented in OpenMolcas, which can be used in combination with any flavor of CASPT2 available in the package.^212^

**Figure 11:**
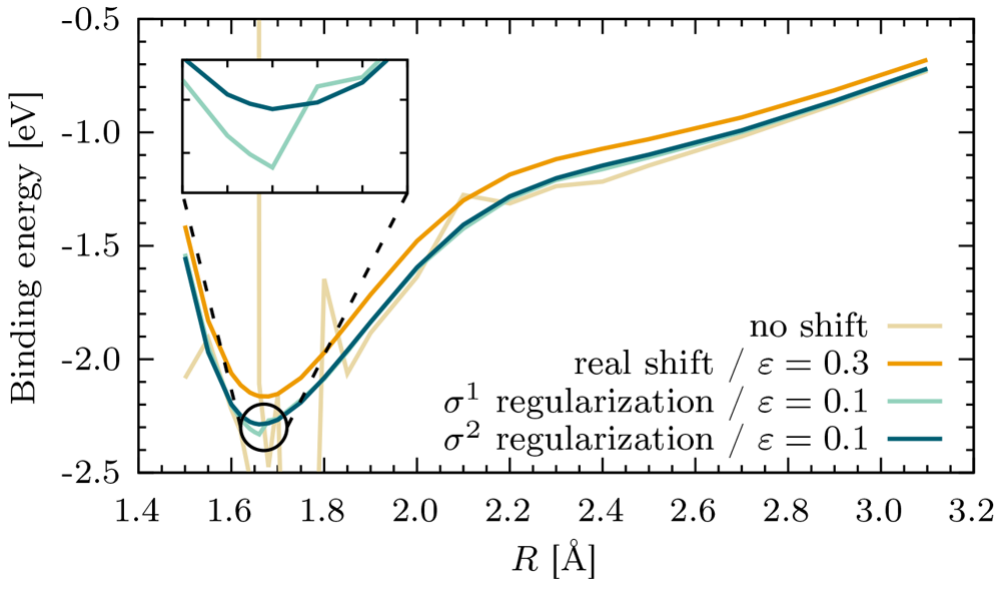
Dissociation of the chromium dimer with different shifts and regularization techniques. The real shift removes all intruders only with a large ε value, however significantly shifting the potential energy curve. On the other hand, the two regularizers require a smaller regularization value. The inset shows the discontinuity of σ^1^-CASPT2 around the equilibrium as compared to the smooth curve obtained with σ^2^-CASPT2. The imaginary shift curve is not shown as it is essentially overlapping to the σ^2^-CASPT2 one with a similar value of ε.

To assess the robustness of σ^*p*^-CASPT2 in removing intruder states and its sensitivity with respect to the regularization parameter, this was systematically investigated for more than 300 excitation energies, as well as the paradigmatic dissociation of the chromium dimer^212^ (shown in Figure 11). From the two variants implemented (*p* = 1 and *p* = 2), σ^1^-CASPT2 is the least sensitive approach to the value of σ, though its application is severely limited by the discontinuity of the regularization function at the origin. On the other hand, σ^2^-CASPT2 provides a robust choice that effectively removes the intruder states at any molecular geometry, yet showing only a weak sensitivity of the results to the regularization parameter. In particular, when compared to the level shift techniques, it was found that it slightly outperforms the imaginary shift and is clearly superior to the real shift, which should be avoided altogether.

#### Frozen Natural Orbitals Method Applied to RASPT2

The computational costs in many body perturbation theory (MBPT) treatments can be reduced via *natural orbitals* (NOs) obtained from approximate wave functions. In the frozen natural orbitals (FNO) method,^213,214^ the natural orbitals of the virtual space are built from the eigenvectors of the virtual–virtual sub-block of the following simplified second-order PT density matrix:
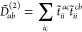
5(*i* is inactive, *a*, *b*, *c* are virtual orbital indices, ε their orbital energies) where the corresponding MP2-like amplitudes, ***t̃***, in canonical orbital basis are given by
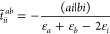
6and the integrals are conveniently computed from the MO-transformed Cholesky vectors, (*ai*|*bi*) = ∑_*j*_*L*_*ai*_^*J*^*L*_*bi*_^*J*^. The NOs span the virtual space as they are obtained by diagonalizing , while encoding in their eigenvalues (occupations) the notion of importance for the subsequent correlation treatment. Hence, based on a suitable selection criterion (see below), only those corresponding to the largest eigenvalues are used to compute the correlated wave function/energy upon rotation back to a canonical form. The matrix of eq 5 is symmetric, positive definite, and for large basis sets shows the typical clustering of its eigenvalues toward zero. This property is independent of the size of the system, as it reflects only the degree of linear dependence of the basis used to span the virtual space. The resulting natural orbitals corresponding to small eigenvalues can then be excluded (“*Frozen*”) from the PT correction to the zeroth-order energy, as their contribution to the dynamical correlation effects should be negligible. For CASSCF reference wave functions, a metric has been used to retain a consistent amount of correlation (even if different amount of NOs) along different nuclear distortions, producing smooth potential energy surfaces (PESs).^215,216^ This metric ζ(ν) defines the fraction of NOs to be retained as follows:^216^
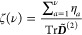
7where the first ν largest eigenvalues η and the trace of the matrix defined in eq 5 are used. For a given value of ζ (ranging between 0 and 1, where 1 is the fully correlated calculation), the number of retained virtual NOs ν in the calculation is dynamically determined, which means it can change for different geometries while still providing the same amount of correlation and thus yield smooth PESs.

Compared to the case of CASSCF reference wave functions,^215,217^ caveats to extending eq 5 for RASSCF arise when the index *i* belongs to either RAS1 or RAS3 spaces.^218^ FNO as conceived for CASSCF uses pseudocanonical active orbitals,^215,217^ obtained by diagonalization of the active–active block of the Fock matrix. These orbitals are then split into two groups on the basis of their eigenvalue. Only those corresponding to negative eigenvalues (primary-active) are retained in the definition of the density matrix of eq 5. It can be argued here in favor of using the same strategy for application of FNO to RASPT2,^218^ despite the fact that this may be expected to have more severe consequences on accuracy. In fact, it is easy to foresee that especially RAS3 orbitals will have very little contribution to eq 5, as by their nature it is very unlikely that any RAS3 orbital will qualify as primary-active. The naïve use of eq 6 may therefore lead to instabilities in the application of FNO in models such as RASSCF that contemplate more than a single active space. Such problems can be overcome through a regularization of the linear equations from which these MP2-like amplitudes originate, in order to give preference to solutions with smaller norms (*L*_2_-regularization). Using the notation Δε to indicate a generic denominator in eq 6, this type of regularization leads ultimately to the following redefinition of the denominators:
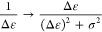
8where the parameter σ is set in input to a suitable value for the system at hand. The value of σ^2^ enters as a scaling factor in the term in the Lagrangian that is associated with the norm of the resulting amplitudes, thus renormalizing diverging amplitudes toward zero. Noticeably, in fact, the regularized expression of eq 6 behaves as Δε/σ^2^ for small values of Δε, whereas it reduces to 1/Δε for denominators large compared to σ. Finally, it is worth pointing out that this renormalized expression for the denominators is the same as the one used for the evaluation of the first-order correction to the wave function and the second-order energy with an imaginary shift,^209,212^ but in the present case it is used exclusively for the evaluation of the simplified PT density matrix through eq 5.

**Figure 12:**
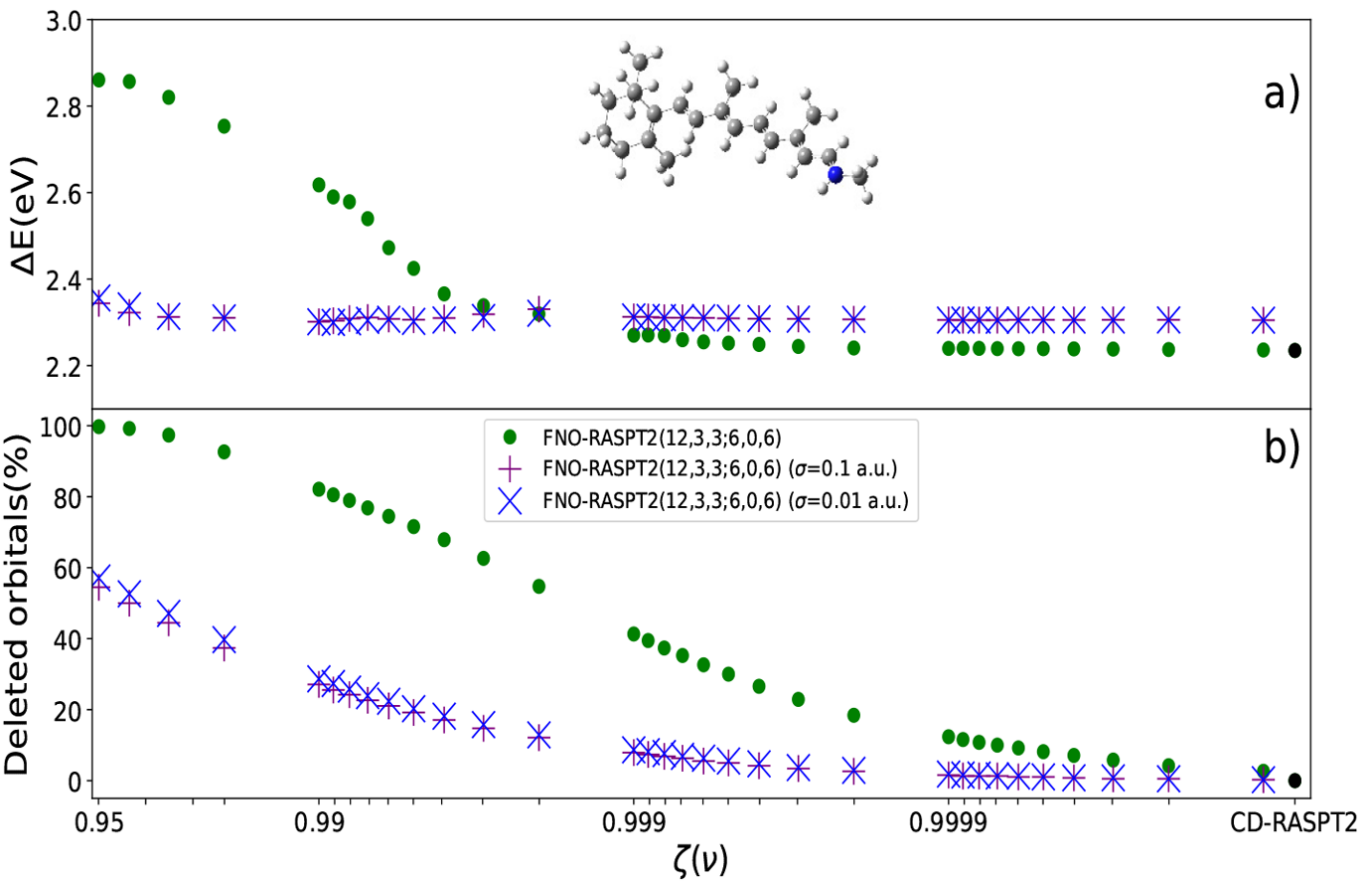
FNO-RASPT2 vs CD-RASPT2 benchmark for PSB-11 (structure provided as an inset): (a) vertical excitation energy (Δ*E*, in eV) for the S_0_ → S_1_ππ* excited state, and (b) percentage of virtual orbitals deleted in each calculation. Two different choices for regularization parameter (σ = 0.1 *E*_h_, in purple; σ = 0.01 *E*_h_, in blue) are provided to measure how they affect the calculations across the ζ = [0.95, 0.9999] range.

Figure 12 shows results of the application of the FNO protocol to RASSCF calculation on the protonated Schiff base 11 (PSB-11). In this example, that uses the ANO-L-VDZP basis set, all the 12 π valence occupied and virtual orbitals of PSB-11 are included in the active space, with the RAS1/RAS3 spaces allowed to have up to 3 holes/electrons, leading to RAS(12,3,3;6,0,6) wave functions. The standard notation introduced by Gagliardi and co-workers is used for RAS active spaces:^219^ RAS(*n*, *l*, *m*; *i*, *j*, *k*) where *n* is the number of active electrons; *l* is the maximum number of holes allowed in RAS1; *m* is the maximum number of electrons to enter in RAS3; and *i*, *j*, and *k* denote the number of orbitals in RAS1, RAS2, and RAS3 subspaces, respectively.

The vertical excitation energy S_0_ → S_1_ values (see Figure 12a) are heavily overestimated unless the regularization shown in eq 8 is employed. The reasoning behind this can be seen in Figure 12b: across the whole range of correlations ζ(ν) explored, the standard FNO-RASPT2 fails to correctly determine the amount of NOs that can be safely excluded from the calculation, removing almost all virtual orbitals when aiming to retain ∼95% of the correlation. Regularization (both at σ = 0.1 *E*_h_ and σ = 0.01 *E*_h_) fixes this issue and produces energies already at ζ = 0.95 that are almost the same as those of the full CD-RASPT2 calculation. This is in agreement with what was previously found for FNO-CASPT2.^215^

The results shown in Figure 12a suggest a slight blue-shift in energy is to be predicted when using the regularized FNO-RASPT2; this is in line with what has been observed in CASPT2 calculations, where electronic excitation can be systematically blue-shifted by increasing the value of imaginary level shift employed.^220^ Despite this caveat, ζ = 0.95 allows removing ∼60% of the virtual orbitals for a DZ basis set and already provides excitation energies that are within a tenth of an eV of the full CD-RASPT2 reference calculation. A more extensive numerical benchmark is underway and will soon be published, providing further guidelines as to how to apply this method more generally.

This regularization technique enables the use of the FNO protocol with RAS wave functions and is expected to be suitable for generalized active space (GAS)^17^ models, thereby paving the way for extending the applicability of multiconfigurational perturbation theory^221^ to excited state calculations on larger systems treated with more diffuse and accurate basis sets.

### 2.8 Multiconfiguration Pair-Density Functional Theory

Multiconfiguration pair-density functional theory (MC-PDFT)^75,77,222,223^ combines density functional theory with multiconfiguration wave function theory. Here a brief overview of MC-PDFT in OpenMolcas is offered before the individual new developments are discussed.

MC-PDFT allows a natural way to treat inherently multiconfigurational systems for which a single Slater determinant (as used in Kohn–Sham density functional theory) does not provide a good zeroth-order description due to near-degeneracy correlation effects. MC-PDFT uses a multiconfiguration wave function as a reference wave function. The reference wave function can be obtained from single-state (SS, also called state-specific) or state-averaged (SA) CASSCF,^68^ RASSCF,^16^ or GASSCF,^17^ including the separated-pair approximation,^108^ from CAS-CI,^105^ RAS-CI,^105^ or GAS-CI,^224^ or from Stochastic-CI or DMRG. The kinetic energy, the density, and the on-top pair density are computed from the multiconfigurational reference wave function. The MC-PDFT energy is calculated as the classical energy computed from the wave function and the on-top energy computed from a functional, called the on-top functional, of the density and the on-top pair density. Thus, the energy is
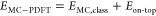
9where
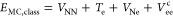
10and
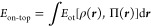
11where *E*_MC,class_ is the classical energy; *E*_on-top_ is the nonclassical energy; *V*_NN_, *T*_e_, *V*_Ne_, and *V*_ee_^c^ are respectively the nuclear–nuclear repulsion energy, electronic kinetic energy, nuclear–electron attraction energy, and the classical two-electron Coulombic energy of the multiconfiguration wave fuction; ρ(***r***) and Π(***r***) are respectively the electron density and the on-top pair density at point ***r***; and *E*_ot_[ρ(***r***), Π(***r***)] is the on-top energy density at point ***r***. The density and on-top pair density are computed as
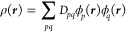
12
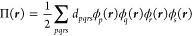
13where *p*, *q*, *r*, and *s* are indices of molecular orbitals, ϕ_*p*_(***r***) is the value (assumed real) of the *p*th molecular orbital at point ***r***, and *D*_*pq*_ and *d*_*pqrs*_ are the one- and two-electron RDMs, respectively, in the molecular-orbital basis.

The currently used on-top functionals are obtained by translating Kohn–Sham exchange–correlation functionals, which depend on α and β densities, into functionals of ρ and Π. For example, in the translated PBE functional^75,225^ (called tPBE), effective α and β densities and density gradients are computed from ρ and Π and then used in the PBE exchange-correlation functional:
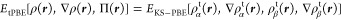
14where ∇ denotes differentiation with respect to the argument ***r***, and symbols with superscript “t” such as ρ_α_^t^(***r***) refer to effective spin densities that depend on the on-top pair density, Π(***r***), as well as the total density. The effective spin densities are not measures of true spin densities, but rather they are intermediate quantities that provide information about the multiconfigurational and open-shell characters of the electronic structure. OpenMolcas can use on-top functionals defined by using the original translation protocol,^75^ for example, tLSDA,^226,227^ tPBE,^225^ trevPBE,^228^ tBLYP,^229,230^ and tOPBE,^225,231^ and it can also use “fully translated” (ft) functionals, defined later,^76^ for example, ftLSDA, ftPBE, ftrevPBE, ftBLYP, and ftOPBE. The fully translated functionals use the gradient of the pair density, ∇Π(***r***), as well as that of the density, ∇ρ(***r***), when computing the gradients of the effective spin densities.

MC-PDFT is typically as accurate as, and sometimes more accurate than, CASPT2^232^ for vertical excitations,^233−242^ barrier heights,^243,244^ singlet–triplet gaps,^121,245−248^ spin-state orderings,^249^ and bond energies.^76,108,248,250−254^

**Figure 13:**
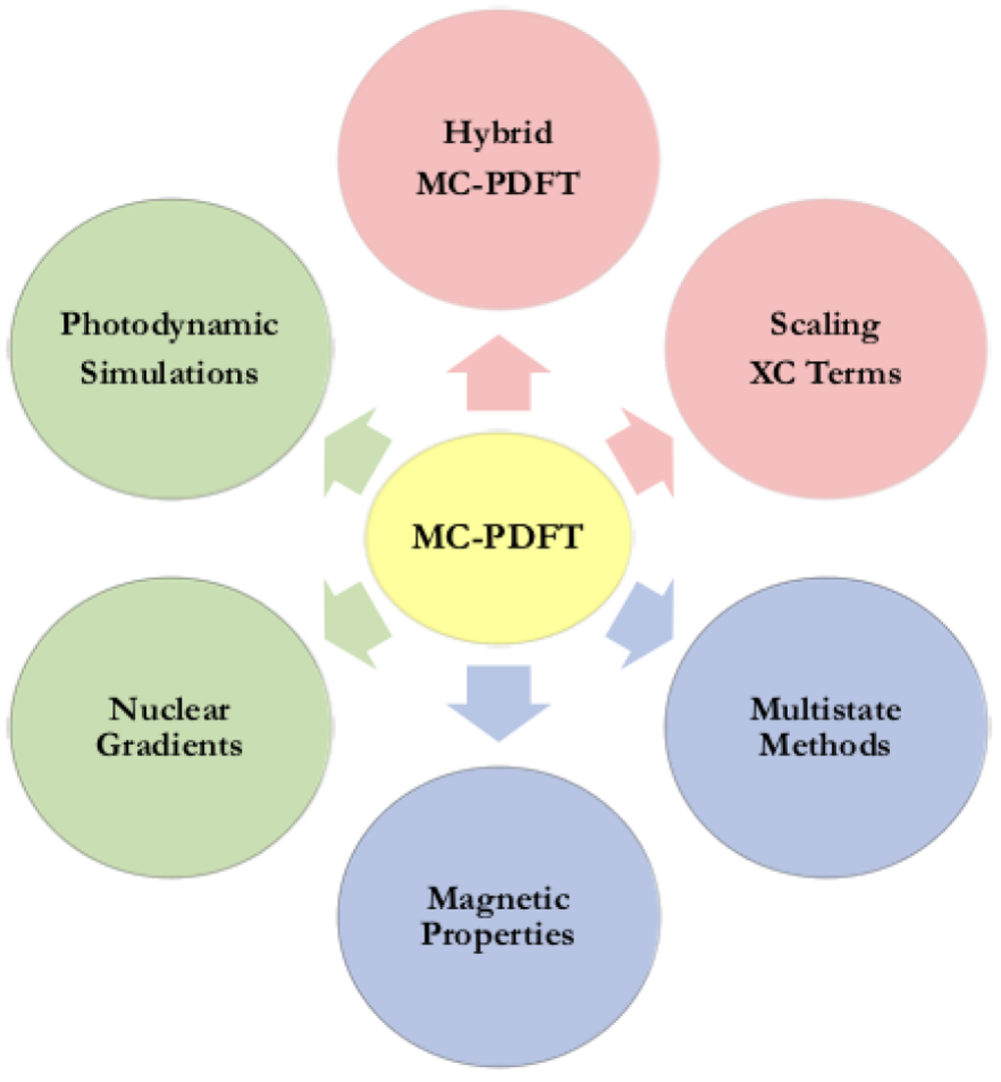
New MC-PDFT capabilities available in OpenMolcas are divided into functional developments (red), methodological developments (blue), and feature implementations (green). The new capabilities include hybrid MC-PDFT, scaling of exchange and correlation (XC) terms in density functionals, multistate methods, magnetic properties (including spin–orbit coupling), gradients needed for force calculations, and simulations involving electronically excited species as in photochemistry.

Here developments since the publication of ref (9) are emphasized. Recent theoretical developments of MC-PDFT that have been implemented in OpenMolcas are shown in Figure 13. Options for hybrid^255^ and scaled^256,257^ on-top functionals with improved accuracy in reproducing experimental excitation energies have been added. Methodological developments that allow for including spin–orbit coupling^258−260^ and/or state-interaction effects^261−263^ have been implemented. Finally, feature extensions such as analytic gradient evaluations^264−267^ and interfaces with electronically nonadiabatic molecular dynamics software^268−270^ that enable ab initio dynamics simulations of photochemical processes at lower computational cost than other electronic structure methods of equal accuracy for strongly correlated systems are reported in the appropriate sections below, see sections 4.2, 6.3, and 6.7, respectively.

In association with these new developments, the OpenMolcas DFT infrastructure has been completely rewritten, via the integration of the Libxc library^271^ as standard platform for the implementation of density functionals. Old DFT code in OpenMolcas has been deprecated, and hundreds of additional functionals are introduced and are now available for standard DFT calculations as well as MC-PDFT computations. Functionals that depend on the electron density Laplacian are also supported for KS-DFT calculations (not yet for MC-PDFT).

#### Hybrid MC-PDFT

Hybrid MC-PDFT (HMC-PDFT) introduces nonlocal exchange and correlation from the underlying multireference wave function into the MC-PDFT energy and is now available in OpenMolcas.^255^ The HMC-PDFT energy is given by
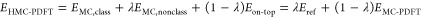
15where *E*_MC,nonclass_ and *E*_ref_ are, respectively, the nonclassical energy and the total energy computed from the reference wave function. The individual components in eq 15 are computed and reported in the course of a standard MC-PDFT calculation; thus, unlike hybrid Kohn–Sham functionals, the HMC-PDFT calculation does not cost more than the nonhybrid counterpart. This attractive feature, along with its superior performance, has allowed the method to be successfully applied for calculation of excitation energies, dipole moments, and energy differences of spin states.^255,272−277^ Benchmark tests on a diverse set of excitation energies suggest that for the tPBE on-top functional, the optimal hybridization parameter is λ = 0.25, the same as in the “PBE0” exchange–correlation functional^278,279^ of Kohn–Sham density functional theory; this hybrid on-top functional is referred to as “tPBE0”.^255,272^

The *LAMBDA* keyword is used in the MC-PDFT program to control the hybridization. The recommended tPBE0 functional, for instance, can be specified by *LAMBDA = 0.25*. Using the same keyword, *LAMBDA*, the user can also change the values of the diagonal elements of the model-space Hamiltonian in multistate PDFT calculations (discussed below); the hybrid energies on the diagonal become a linear combination of the PDFT and conventional wave function energies of the intermediate-state wave functions.

**Figure 14:**
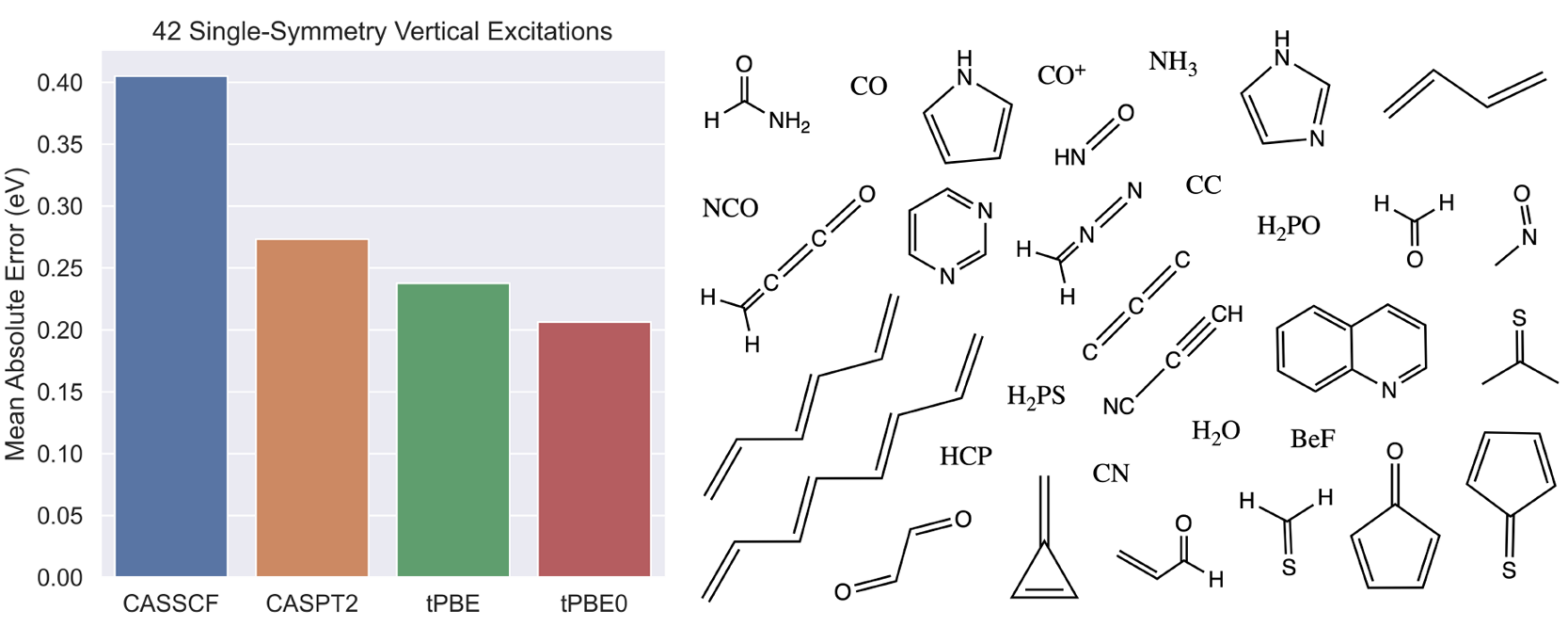
Mean absolute error of CASSCF, CASPT2, tPBE, and tPBE0 calculated in OpenMolcas for 42 single-symmetry excitations in the QUESTDB data set of benchmark vertical excitations.^280^ This test includes excitations in which the ground and excited state have the same spin quantum number and point-group irrep (in up to *D*_2h_ symmetry supported by OpenMolcas). It includes all the excitations in QUESTDB for which the active space determined in a previous study^272^ was assumed to be good enough (the criterion was that the tPBE0 excitation energy was within 0.55 eV of the benchmark value). Those active spaces were then targeted in OpenMolcas by specifying the number of doubly occupied and active orbitals of each irrep to be selected from RHF/UHF orbitals; if the calculation converged and the CASSCF value was within 1.1 eV of the benchmark, then it was included in the final set of 42 excitations.

Figure 14 shows the performance of CASSCF, tPBE, tPBE0, and CASPT2 on a set of 42 single-symmetry excitations from the QUESTDB data set^280^ of benchmark vertical excitations; these results were obtained with the aug-cc-pVTZ basis set. Active spaces were selected from RHF or UHF orbitals by stipulating the size of the active space and orbital symmetries from the “Aug(12,12)” results of a recent benchmark study that employed an automated scheme.^272,281^ It is found that for vertical excitations on small-to-medium-sized organic molecules such as those shown in Figure 14, tPBE0 improves on the performance of tPBE by 0.05 eV.^272^ Interested readers may refer to a recent benchmark study^272^ for an analysis of the complete set of 542 vertical excitations, in which the mixing parameter of λ = 0.25 used in tPBE0 is found to be optimal. In the smaller analysis presented here, we find that tPBE0 also performs better than CASPT2.

#### Scaling Exchange and Correlation

To allow for flexibility in the exchange–correlation functionals that can be used in Kohn–Sham density functional theory and the on-top functionals that can be used in MC-PDFT, the capability of scaling the exchange and correlation terms has been introduced for any Kohn–Sham-theory exchange–correlation functional or any MC-PDFT on-top functional that can be written as a sum of exchange and correlation terms. Scaling factors *c*_X_ and *c*_C_ have been introduced such that
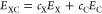
16where *c*_X_ = *c*_C_ = 1 would reproduce the original functional. The input of these scaling factors can be done either through a flexible input via Libxc functional factors, or by use of the *DFCF* keyword.

The DFT functional suffix -HLE (high local exchange) is used to denote the special choice of *c*_X_ = 1.25 and *c*_C_ = 0.5, which was originally introduced in Kohn–Sham density functional theory and linear-response TDDFT to improve the prediction of band gaps and electronic excitation energies.^282,283^ It has now been widely tested in KS-DFT, with mixed success,^284−289^ which is consistent with its proposed role as a functional suitable for selected (but not all) applications. It has also been tested in MC-PDFT, and it has been found that, when compared to tPBE, the tPBE-HLE on-top functional (i.e., tPBE with *c*_X_ = 1.25 and *c*_C_ = 0.5) significantly improves the prediction of the spin-state energies of transition metal complexes,^257^ although it appears to degrade the prediction of spin-forbidden main-group atomic excitation energies and bond dissociation energies.^256^ However, it is noted that HLE significantly improves the spin-splitting energies in Kohn–Sham density functional theory.^286^

#### Multistate Methods

In order for potential-energy-surface topological features such as conical intersections to be correctly reproduced by a quantum-chemical method, and in order to obtain consistent energies for nearly degenerate states, state energies should be obtained as eigenvalues of effective Hamiltonian matrices. For example, CASPT2^232^ should be replaced with MS-CASPT2,^205^ XMS-CASPT2,^206^ or RMS-CASPT2,^212^ especially when considering degenerate or nearly degenerate states. Since photochemistry is usually dominated by conical intersection seams and their nearby vicinities, this is essential for photochemical simulations.

For MC-PDFT, several methods have been proposed in which the last step is a diagonalization of a Hamiltonian matrix.^261−263,290,291^ Two of these methods, extended multistate PDFT (XMS-PDFT)^262^ and compressed multistate PDFT (CMS-PDFT),^263^ can be executed in OpenMolcas with simple keywords. These two methods are generically called multistate methods (MS-PDFT), and in the spirit of quasidegenerate perturbation theory,^292^ they transform a small number of SA-CASSCF eigenvectors to a new set of states called intermediate states. The space spanned by the chosen SA-CASSCF eigenvectors and hence also spanned by the intermediate states is called the model space. The diagonal and off-diagonal elements of the model-space Hamitonian matrix are calculated differently. The diagonal elements are the MC-PDFT energies of the intermediate states, and the off-diagonal elements are computed by wave function theory, as in a configuration interaction calculation. The XMS-PDFT and CMS-PDFT methods differ in their choice of intermediate states.

The XMS intermediate states diagonalize Granovsky’s choice of the zeroth-order Hamiltonian matrix that is used in XMS-CASPT2^206^ and in extended multiconfiguration quasi-degenerate perturbation theory (XMCQDPT).^292^ Because the intermediate states are obtained through a diagonalization, XMS-PDFT is the most efficient of the currently available multistate PDFT methods.

The CMS-PDFT intermediate states maximize the sum over the model-space states of the classical electron–electron Coulomb energies. Because a higher classical Coulomb energy means a more compressed electronic density, the CMS intermediate states are more physically motivated than the XMS intermediate states. CMS-PDFT is more expensive than XMS-PDFT, and it finds the intermediate states by an iterative process that can have convergence and uniqueness complications; however, CMS-PDFT shows better accuracy than XMS-PDFT in some tested cases.

**Figure 15:**
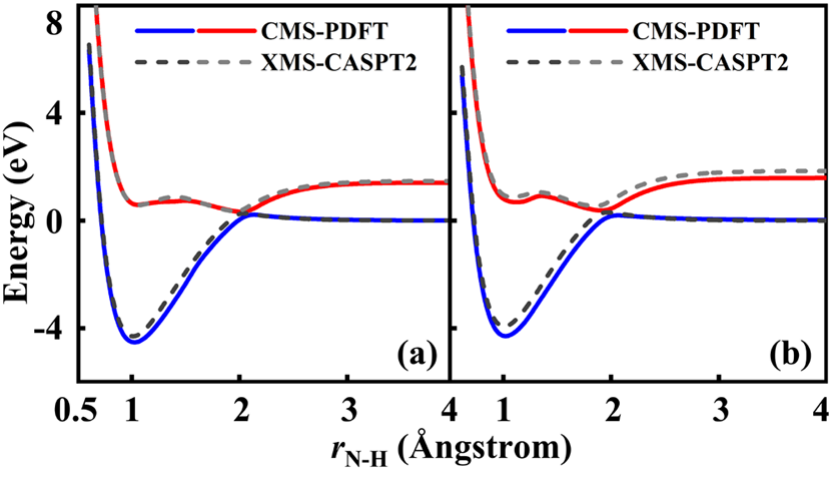
Potential energy curve of N–H dissociation in methylamine, along a dissocation path with the H–C–N–H dihedral angles of 0° in (a) and 90° in (b), respectively. This figure is made with data from the Supporting Information of ref (263).

Figure 15 shows the potential energy curves for the N–H dissociation in methylamine. The paths chosen for this figure go close to conical intersections, and therefore they show narrowly avoided intersections (locally avoided crossings), which provide a difficult test for calculations of excited-state potential energy surfaces. The figure shows that CMS-PDFT provides similar potential energy curves to those from XMS-CASPT2 for this problem.

#### Spin–Orbit Coupling and Magnetic Properties

Spin–orbit coupling is important for the calculation of accurate energies and energy differences for molecules with open-shell character on heavy atoms (atoms in the rows of the periodic table with *n* ≥ 4). It is also important for magnetic property calculations, including magnetic anisotropy, electron paramagnetic resonance spectroscopy, the Zeeman effect, and zero-field splitting (ZFS). OpenMolcas can include spin–orbit coupling by constructing a spin–orbit Hamiltonian at the MC-PDFT, XMS-PDFT, or CMS-PDFT level, and it enables computation of the magnetic properties at a lower computational cost than post-SCF multireference methods based only on wave functions.^258,259^ To calculate the spin–orbit coupling, one must provide the energy and wave function for a set of spin-free states with two or more spin multiplicities.^293,294^ In the current version of OpenMolcas, a keyword *WJOB* has been added in the MCPDFT module to facilitate this. This keyword allows one to write the required information into the JOBIPH file. For MC-PDFT, only the energy of each state is written into the JOBIPH file because an MC-PDFT calculation is based on a previous wave function; for XMS-PDFT and CMS-PDFT, both the energies and the CI coefficients of the eigenstates (obtained by diagonalizing the model-space Hamiltonian in MS-PDFT) are written into the JOBIPH file. Then the JOBIPH file is read in the RASSI module, where spin–orbit coupling matrix elements are calculated by the atomic-mean-field approximation^295^ to the Breit–Pauli Hamiltonian. The spin–orbit-inclusive states are calculated by the restricted-active-space state-interaction spin–orbit (RASSI-SO) formalism^295,296^ that allows the Hamiltonian to be diagonalized even though the wave functions of the spin-free states with different multiplicities were calculated with different orbital sets. The spin–orbit treatment in OpenMolcas is reviewed in detail in a previous article.^258^

The spin–orbit capabilities of OpenMolcas have been used with MC-PDFT for various applications: (1) The *g* tensors were computed by MC-PDFT and XMS-PDFT for 25 transition-metal complexes.^258^ A CASCI-PDFT scheme was developed and was shown to be more than a factor-of-2 more accurate than conventional PT2 calculations. (2) Zero-field splitting parameters^259^ were studied by MC-PDFT and CMS-PDFT using orbitals optimized by weighted-state-averaged CASSCF, and it was shown that PDFT is accurate and efficient.^259^ (3) The spin–orbit energy of ground-state Ce^+^ and the bond energy of CeH^+^ were computed, illustrating the calculation for a very heavy (*n* = 6) metal.^260^ (4) Singlet–triplet gaps and zero-field-splitting parameters were calculated for Cr^IV^ aryl complexes, and both MS-CASPT2 and CMS-PDFT were found to be more accurate than calculations by Kohn–Sham DFT.^274^

## 3 Electronic Spectroscopy

The availability of more sophisticated and efficient methods, like those described in section 2, enables the simulation of more complex processes with a higher accuracy. However, for performing and interpreting such simulations, new developments are often required. This section details some recent developments that allow the use of OpenMolcas for the simulation of different types of magnetic properties and electronic spectroscopy, in particular those where the approximation of a fixed molecular structure—i.e., frozen nuclei—can be made. First an improvement beyond the so-called Lines approximation in the calculation of anisotropic exchange interaction in binuclear systems, achieved by modifications to the SINGLE_ANISO module, is described. Subsequently alternative tools are presented, molcas_suite and angmom_suite—both open-source packages in PyPI—for the same type of analysis. This is followed by the description of three extensions or modifications of the RASSI code. First, the facilitation of the calculation of electron–nucleus hyperfine coupling at relativistic level and the analysis of spin-forbidden transitions. Second, for the computation of transition intensities, the performance of the exact semiclassical light–matter interaction operator has been significantly improved, making it possible to efficiently calculate two-photon scattering processes. Third, excited state properties of molecular aggregates can now be computed with multiconfigurational wave function methods with the Frenkel excitonic coupling model, a formalism that separates dimers into two interacting monomers. The computation of Dyson orbitals, required for the simulation of photoionization dynamical parameters, has been improved by modifications to the RASSI module. Interfaces to the Tiresia(297) and SCAMPI(298) codes for the electronic continuum have been developed. Moreover, the implementation of two-particle Dyson matrices enables the simulation of single-site double-core-hole ionization and of normal and resonant Auger–Meitner autoionization spectra within the one-center approximation at the RASSCF/RASPT2 levels of theory. Finally, ultrafast electron dynamics, including those triggered and stirred by ultrashort light pulses, can be studied at the time-dependent configuration interaction level, using the quantities available in static multiconfigurational electronic structure methods. All these topics are discussed in more detail below.

### 3.1 Derivation of Anisotropic Exchange Interaction from Binuclear Ab Initio Calculations

The anisotropic exchange interaction between metal ions is the basic ingredient determining the properties of polynuclear metal complexes and magnetic materials with a significant spin–orbit coupling on the metal sites. For a pair of magnetic centers characterized by the pseudospins  and  respectively, the general form of exchange interaction is given by the following Hamiltonian:
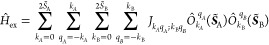
17where  are the exchange parameters and  are generalized Stevens operators for the pseudospin ***S*~**.^299^ On the ranks of these operators (*k* and *q*), the condition *k* + *q* = even is imposed due to a required invariance of  with respect to time inversion. While the isotropic (Heisenberg) exchange interaction, realized in the absence of appreciable spin–orbit coupling on the magnetic centers, contains one single exchange parameter, the anisotropic exchange interaction can involve many dozens of them depending on the size of  and  and on the strength of spin–orbit coupling on the metal sites. This precludes direct extraction of anisotropic exchange parameters from the experiment.

To simplify the description of anisotropic exchange interactions, the Lines model^300^ has been earlier implemented in the POLY_ANISO program^301^ incorporated in OpenMolcas. The details of using the Lines approach combined with ab initio calculations of individual metal centers can be found in ref (302). However, the Lines model is strictly valid only in the limits of (1) strongly axial doublets on the metal sites and (2) isotropic spins on magnetic centers. For other (intermediate) cases, the evaluation of all  parameters entering the expression for  should be done a priori. To this end, a fully ab initio methodology has been developed to extract the entire set of  parameters from CASSCF/CASPT2/RASSI-SO calculations of binuclear magnetic complexes and fragments. As a prerequisite, one-center calculations of the individual metal fragment are performed from which the ZFS and Zeeman Hamiltonians on each magnetic center are derived with the SINGLE_ANISO program. During their derivation, the pseudospins at individual magnetic sites are defined and subsequently used for the derivation of different contributions to  by applying irreducible tensor techniques^299^ to the lowest  multiplet states of the binuclear complex or fragment. The methodology is implemented in the function *PREX*, entering the current version of the SINGLE_ANISO module.

As an example, the derivation of anisotropic exchange parameters for a recently studied Dy^III^Mn^II^ complex^303^ is presented. The calculations with the SINGLE_ANISO program show that the ground Kramers doublet at the Dy^III^ site  is highly axial (*g*_*z*_ ≫ *g*_*x*_, *g*_*y*_). The CASSCF/CASPT2/RASSI-SO calculations for the Dy^III^ fragment (in which Zn substitutes Mn) have been done with OpenMolcas in a standard fashion for Ln complexes.^302^ Since the Mn^II^ ion is a high-spin one (*S*_B_ = 5/2), the corresponding *g*-tensor is a priori isotropic, with *g* factors close to 2.0, which exempts us from the ab initio investigation of this single-ion fragment. Repeating then the CASSCF/CASPT2/RASSI-SO calculations for the whole DyMn binuclear complex and applying *PREX* function to its lowest  multiplets, the anisotropic exchange parameters are derived. The parameters corresponding to *k*_*A*_ = 1 and *k*_*B*_ = 1, 3, 5, allowed by time-reversal symmetry, are shown in Table 1 (the neglected contribution for *k*_*A*_ = *k*_*B*_ = 0 gives only an unimportant energy shift of all levels). It can be seen that the first-rank contributions (*k*_*A*_ = *k*_*B*_ = 1) are by far the dominant ones. These contributions can be recast in the form of noncollinear Ising interaction.^304^ Remarkably, this form of exchange interaction is also predicted by the Lines approach, which applies in the present case (one magnetic ion is axially anisotropic and another fully isotropic). However, the Lines approach entails an unknown parameter (the Lines exchange parameter^302^) extracted from magnetic data fitting. On the contrary, the anisotropic exchange parameters obtained with the proposed ab initio approach (Table 1) already give a satisfactory description of the magnetic susceptibility of the Dy^III^Mn^II^ complex.

**Table 1:** Parameters of the Anisotropic Magnetic Exchange (in cm^–1^) Extracted from Calculation of the DyMn Binuclear System^a^

*k*_*A*_	*q*_*A*_	*k*_*B*_	*q*_*B*_	Real Part	Imaginary Part
1	0	1	0	–1.657	2.696 × 10^–18^
1	0	1	–1	2.076 × 10^–2^	–1.278 × 10^–3^
1	0	1	1	–2.076 × 10^–2^	–1.278 × 10^–3^
1	0	3	0	–5.864 × 10^–3^	–4.397 × 10^–19^
1	0	3	–2	7.061 × 10^–4^	1.746 × 10^–5^
1	0	3	2	7.061 × 10^–4^	–1.746 × 10^–5^
1	–1	1	1	–4.702 × 10^–4^	1.987 × 10^–4^
1	1	1	–1	–4.702 × 10^–4^	–1.987 × 10^–4^
1	0	3	–1	7.228 × 10^–5^	3.249 × 10^–4^
1	0	3	1	–7.228 × 10^–5^	3.249 × 10^–4^
1	–1	1	–1	–7.385 × 10^–5^	9.054 × 10^–5^
1	1	1	1	–7.385 × 10^–5^	–9.054 × 10^–5^
1	0	3	–3	7.169 × 10^–5^	–7.316 × 10^–5^
1	0	3	3	–7.169 × 10^–5^	–7.316 × 10^–5^
1	–1	3	3	1.936 × 10^–5^	–4.496 × 10^–5^

a The active space of the CASSCF method included 4f^9^ and 5d^5^ shells of the Dy^III^ and Mn^II^, respectively, amounting to 14 electrons in 12 orbitals. All roots arising from the coupling of the ground ^6^H term of Dy^III^ and ground spin *S*_B_ = 5/2 of Mn^2+^ were explicitly optimized and mixed by spin–orbit interaction in RASSI. The ANO-RCC-VTZP basis set was used for closer atoms, while smaller VDZP contractions were used for distant atoms. The first 15 parameters are shown in descending order of importance.

### 3.2 Model Hamiltonian Projection

As an alternative to the methodologies available within OpenMolcas presented in section 3.1, model Hamiltonians for one or two spin centers with arbitrary angular momenta can be projected directly from the output of a CAS/RASSCF/(CASPT2)/RASSI-SO calculation (via the rassi.h5 file) using the open-source molcas_suite and angmom_suite packages available on PyPI.

This implementation is based on the projection of a given set of ab initio states onto an arbitrary set of angular momentum eigenstates and the subsequent determination of parameters of numerous spin Hamiltonian terms using irreducible tensor operator techniques. The transformation from the ab initio eigenstates into a basis of well-defined angular momenta is carried out in two steps: (1) the spin-free ab initio states are projected via the group theoretical orthogonal projector  onto a set of LS-terms which transform under the (2*L* + 1)-dimensional irreducible representations of SO(3) and span the selected model space; then (2) the obtained terms are (de)coupled via Clebsch–Gordan vector (decomposition or) addition to yield the transformation to the final model basis. The correspondence between the ab initio and orbital angular momentum eigenstates of each spin-free *L*-term is established by diagonalization of *L*_*z*_ (and adjustment of phases due to the Condon–Shortley convention), which is analogous to the methods described in section 3.1. The advantage of using the orthogonal projector  is that systems with significant mixing of different *L*-terms can still be brought into correspondence with the canonical orbital angular momentum eigenbasis without significant approximation. Subsequently, the total ab initio Hamiltonian including spin–orbit coupling is transformed into this new angular momentum basis, and arbitrary spin Hamiltonian parameters are determined by projection with their matrix representation, exploiting the orthogonal character of their construction. This flexible implementation of model Hamiltonian projection supports numerous spin Hamiltonian terms such as (an)isotropic spin–orbit coupling, exchange interaction^305^ and the crystal field potential^306,307^ for any angular momenta present in the model basis. The only requirements are that the ab initio states contain the sufficient angular momenta to match the model space as defined by the user; this can fail, for example, in the case where strong covalency of the 5f shell of actinides means that the orbital angular momentum operator is ill-defined. The presence of the appropriate manifold of angular momentum states can be assessed by molcas_suite.

The use of this method is exemplified in the case of Cp^iPr5^TbI_3_TbCp^iPr5^,^308^ where the calculation of the full exchange spectrum arising from the exchange interaction of the two angular momenta (*L*_1_ = *L*_2_ = 3) and spins (*S*_1_ = *S*_2_ = 3) of the terbium centers with a single radical spin (R = 1/2) situated in a bridging σ-orbital is demonstrated. Isotropic spin–orbit coupling and the crystal field potential at each individual terbium site, as well as the exchange coupling between the terbium ions and the radical, are included. All required parameters are obtained via projection using molcas_suite from two ab initio calculations of fragments constituting each Tb-radical pair, which each span the |^8^F⟩ ⊕ |^6^F⟩ manifold. The parameters so-obtained can be used to construct the model Hamiltonian for the full Cp^iPr5^TbI_3_TbCp^iPr5^ molecule using the angmom_suite package, a calculation which is currently inaccessible using ab initio CASSCF methods alone. The eigenstates of the model Hamiltonian can then be used to compare to experimental spectra of the molecule, along with magnetic properties such as the temperature dependent magnetic susceptibility and electron paramagnetic resonance (EPR) *g*-tensors. Furthermore, analyzing the composition of the ab initio states (molcas_suite) as well as the eigenstates of the model Hamiltonian (angmom_suite) in terms of the angular momentum basis can aid the interpretation of various spectroscopic and magnetic properties.

### 3.3 Relativistic Hyperfine Coupling

The electron–nucleus hyperfine coupling (HFC) is known to be extremely sensitive to relativistic effects–even finite nuclear volume corrections may exceed 10% in magnitude for isotopes such as ^199^Hg.^309,310^ Furthermore, the orbital angular momentum may generate large contributions to the HFC in open-shell metal complexes, either directly or via SO coupling.^311^ A set of options to calculate HFC was implemented in the RASSI module for RASSI-SO calculations,^312^ following a similar approach as developed previously for electron *g*-factors.^294,313^ The initial HFC implementation^312^ had a major limitation, in that the hyperfine integrals were nonrelativistic, thus limiting applications to light atoms or cases where so-called contact terms hardly contribute. This limitation has recently been lifted, with the development of an exact 2-component (X2C) replacement of the HFC option.^314^ Nonrelativistic calculations are still possible, via X2C with a large value of the speed of light. The HFC option is also capable of matrix product state DMRG calculations with QCMaquis and OpenMolcas.^39,118^ Applications in ref (314) showed, among other findings, that ^199^Hg HFC is correctly obtained from RASSI-SO calculations, whereas the previously employed nonrelativistic integrals produce divergent results. The RASSI-SO HFC option (in its original implementation) was also successfully applied to studies of NMR ligand chemical shifts in open-shell actinide complexes, providing the first fully ab initio calculations of these spectroscopic parameters.^315,316^ A persistent challenge is the generation of sufficient spin polarizations in active-space calculations, even with the large active spaces accessible via DMRG.

Alternatively, a similar method for calculation of relativistic HFC parameters based on the X2C transformation has also been implemented in the HYPERION package, which interfaces with OpenMolcas.^317,318^ Similarly to ref (314), HYPERION calculates X2C hyperfine coupling parameters on the basis of CAS/RAS/DMRG wave functions with or without RASSI-SO, and has been benchmarked against selected alkali metal, transition metal, and lanthanide atoms, showing excellent agreement with experimental data from atomic spectroscopy. HYPERION includes an orbital decomposition method for assisting active space selection for calculations of HFC.

### 3.4 Wave Function Analysis for Spin–Orbit Coupled Wave Functions

Recent developments in the RASSI code, have made it possible to extract important properties and information from SO-coupled RASSI wave functions via (1) natural orbitals (NOs) and associated natural spin orbitals (NSOs) and their populations,^7,319,320^ (2) natural bond orbital (NBO) and natural localized molecular orbital (NLMO) analyses of the associated density matrices in the atomic orbital (AO) basis and accompanying utility software^321,322^ interfacing with the popular NBO toolkit,^323,324^ and (3) spin–orbit natural transition orbitals (SO-NTOs).^325^ This functionality generalizes and extends previously available functionality at the spin-free level.

The concept of natural transition orbitals (NTOs)^326−331^ has found many useful applications. NTOs give a compact description of how, and to what extent, two electronic states are connected via a one-electron transition. A recent extension of the concept to the spin–orbit coupled wave functions from OpenMolcas RASSCF + RASSI calculations has enabled a detailed understanding of the intensity of spin-forbidden transitions with the help of SO-NTOs.^325^ For example, with the SO-NTO functionality the (usually weak) intensity of a spin-forbidden transition arising from the nonvanishing transition dipole moment in the presence of SO coupling can be analyzed in terms of the contributing hole and particle NTOs and the associated singular values (amplitudes). Details of the formalism and implementation are provided in ref (325).

**Figure 16:**
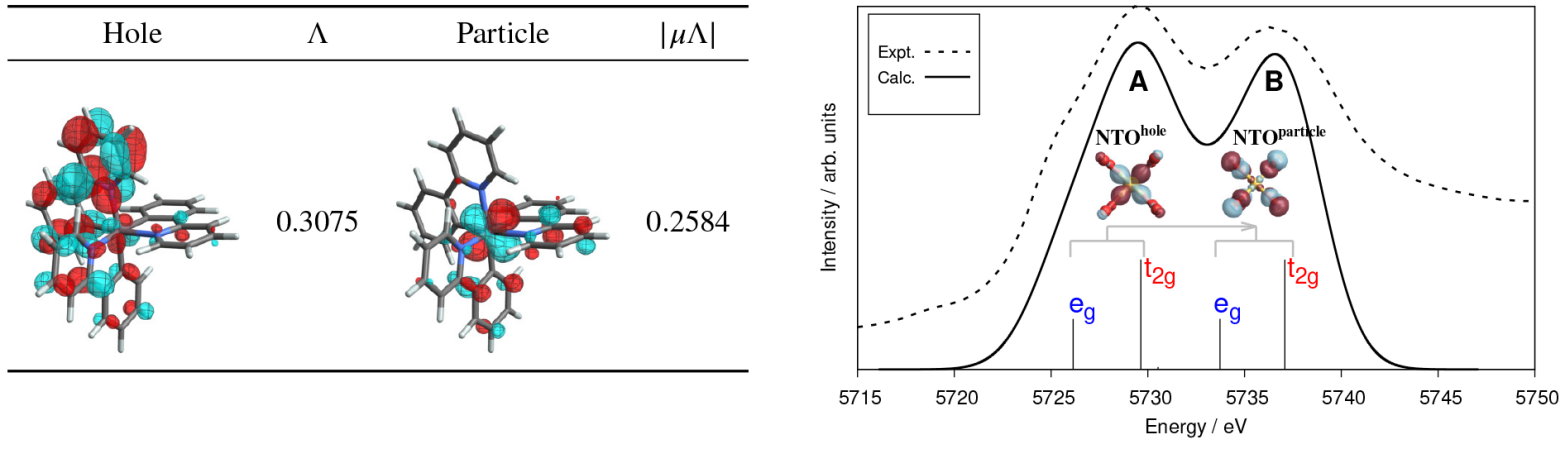
Left: A dominant NTO pair (±0.03 isosurfaces), the associated singular value Λ, and the weighted transition dipole moment NTO contribution |*μΛ*| (*e a*_0_), for the spin-forbidden T_1_–S_0_ transition of [Ir(ppy)_3_]. SO-NTO analysis reported in ref (325). Right: Calculated Ce L_3_ edge for a cluster-embedded model of solid CeO_2_ vs experimental data.^332^ The figure is reproduced from ref (333) with permission from the Royal Society of Chemistry.

Figure 16 (left panel) displays an SO-NTO pair involved in the spin-forbidden emission from the T_1_ to the S_0_ state of the complex [Ir(ppy)_3_] (ppy = 2-phenylpyridine). The availability of orbital information about the source of intensity in spin-forbidden transitions paves the way for a more rational design of phosphorescent emitters, which are important in many fields of chemistry and adjacent disciplines.

X-ray absorption near edge structure (XANES) spectroscopy constitutes an integral part of f-element (actinides, lanthanides) research, offering rich insight on the bonding properties of the metal ion.^333−336^ Calculations of XANES spectra with MOLCAS or OpenMolcas have been reported previously for complexes with light transition metals, but f-element studies have only recently started to appear.^333^ This is, in part, thanks to the developments mentioned above. Being able to perform the analysis of the wave functions directly at the SO level can be crucial for many f-element studies, although in simple cases a spin-free analysis may suffice.

The notorious Ce L_3_ edge of CeO_2_ was successfully calculated ab initio for the first time recently.^337^ Analysis showed that the double white line feature, peaks A and B in Figure 16 (right panel), arises from core transitions into the crystal-field-split 5d e_g_ and t_2g_ orbitals into Ce^III^ 4f^1^ (peak A) and Ce^IV^ 4f^0^ (peak B) subconfigurations. This was long suspected but not previously confirmed by ab initio calculations. In particular, NTO analysis of the transitions of states in peak A to peak B showed unambiguously that the two peaks are connected by single-electron ligand-to-4f subconfiguration transitions. Sample inputs for core RAS and RASSI-NBO calculations can be found in the supporting material of ref (10) and a recent ligand K-edge XANES analysis for An^IV^ hexachlorides.^338,339^

### 3.5 Single- and Two-Photon Spectra with the Exact Semi-Classical Operator

Light–matter interactions are commonly treated using the electric dipole approximation, where the perturbing field is assumed to be constant on the length scale of the system. This approximation fails, e.g., for high-energy photons that have short wavelengths. In OpenMolcas, this was originally addressed through a complete second-order multipole expansion,^340^ which was then applied to high-energy X-ray absorption and scattering processes.^341−344^ However, the multipole expansion itself does not necessarily have a smooth convergence behavior toward the exact result,^345^ and is not origin independent unless using the correct length and velocity gauges.^346,347^ In contrast, the plane-waveform of the wave vector, i.e., the exact semiclassical light–matter interaction operator, shows excellent stability also for small basis sets.^345,348−351^ In OpenMolcas, the operator has been implemented using the Gauss–Hermite quadrature, which makes it easy to implement both isotropic averages and defined directions of wave and polarization vectors.^350,351^ This implementation has also been extended to circularly polarized light, allowing the computation of rotatory strengths and tensors beyond the dipole approximation.^351^

A complication of the exact operator is the dependence on the transition energy between initial and final states which means that new integrals have to be calculated for every transition. For single-photon absorption and emission processes this is not a major problem, because the number of individual transitions is limited and total computational cost is dominated by wave function calculations. However, for scattering processes, RASSI needs to calculate transition intensities not only between the initial state and the intermediate states (photon in), but also between all intermediate and all final states (photon out). In some X-ray scattering processes, with millions of transitions, the original implementation led to intractable demands for evaluation and storage of transition densities. To overcome this bottleneck, two new schemes have been implemented in OpenMolcas: (1) storage of 1-particle transition densities in a compact MO basis and (2) a grouping scheme for energetically close-lying transitions.^352^

**Figure 17:**
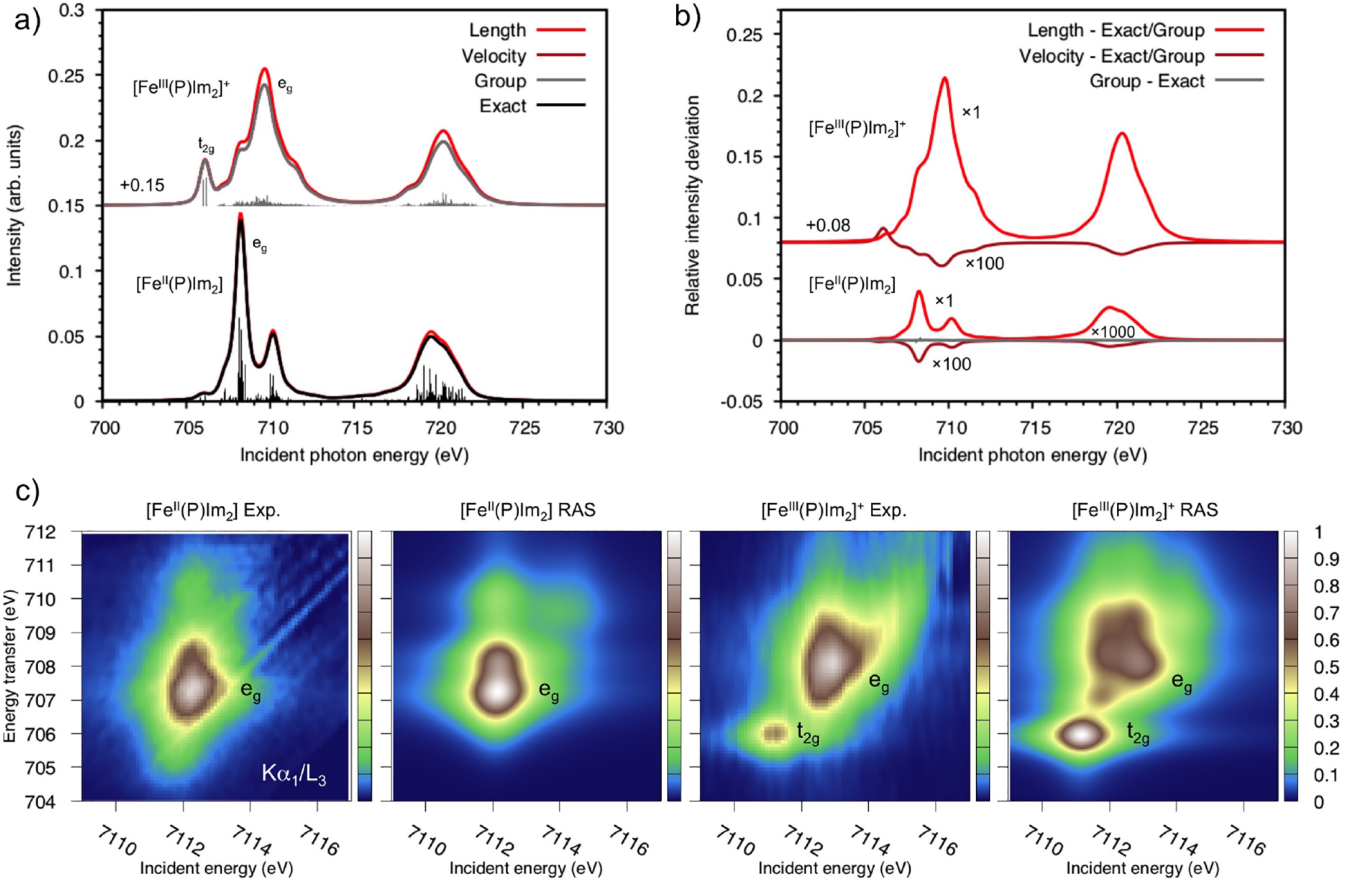
Single- and two-photon X-ray spectra of Fe^II^(P)(ImH)_2_ and Fe^III^(P)(ImH)_2_^+^ models calculated with different electric dipole and exact semiclassical light–matter interaction operators. (a) Single-photon metal L-edge X-ray absorption spectra (XAS). (b) Deviations between different operators for calculations of L-edge XAS spectra, note that the “group” differences are already scaled ×100. (c) Two-photon Kα resonant inelastic X-ray scattering (RIXS) spectra from experiment and RAS modeling using the grouping approximation. Experimental data from ref (353). Adapted from ref (352), licensed under the Creative Commons license CC BY 4.0.

The new implementation has been used to model single-photon (absorption) and two-photon (scattering) X-ray spectra of two iron–porphyrin complexes, ferrous Fe^II^(P)(ImH)_2_ and ferric Fe^III^(P)(ImH)_2_^+^ (P = porphine, ImH = imidazole), of relevance for heme enzymes such as cytochrome c and hemoglobin, see Figure 17.^353,354^ Already for the relatively straightforward calculation of metal L-edge (2p → 3d) X-ray absorption of Fe^II^(P)(ImH)_2_, the original atomic orbital basis required a disk space of 234 GB. This was reduced to 622 MB in the compact molecular orbital basis. With the grouping scheme, the timing for a metal K-edge resonant inelastic X-ray scattering (RIXS) calculation (1s → 3d absorption followed by 2p → 1s emission) of Fe^II^(P)(ImH)_2_ goes from 100 CPU days (projected) to 12 h while giving deviations of no more than 0.1%. This is 2 orders of magnitude lower than the deviations using the complete second-order multipole expansion.^352^ Together with the implementation of the core–valence separation and the improvements in the CI algorithm for calculations with a large number of states reported previously,^355^ the RASSI module in OpenMolcas can now simulate a wide range of single- and two-photon processes.

### 3.6 Evaluating Frenkel’s Excitonic Coupling Terms

Frenkel-exciton theory is often used to study excited states and electronic structures of weakly bound (molecular) aggregates.^356,357^ Briefly, for an aggregate with Hamiltonian
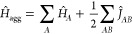
18monomer electronic structure is computed with as accurate a quantum-chemical method as available, corresponding to , followed by an approximation of the intermonomer coupling term . A simple dipole approximation^358^ can be used for  at large monomer–monomer distances, but it fails once the distance is akin to monomers’ molecular size,^359^ and Coulomb interactions have to be properly described.^360^ For instance, the most important term corresponds to the interaction of local transition densities ϱ of monomers *A* and *B*:^361^
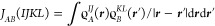
19Here, *I* and *J* are the local electronic states of monomer *A*, and *K* and *L* of monomer *B*, respectively; ***r*** and ***r***′ are electronic coordinates. Other terms include interactions of transition densities with the nuclear charge of the other monomer, and internuclear repulsion.

Recently, efficient protocols for the computation of excitonic couplings based on time-dependent density functional theory have been suggested.^362−364^ However, many chromophores (e.g., highly conjugated molecules, or molecules in states with double excitation character) may not be properly described by single-determinant-based methods. To the best of the authors’ knowledge, the only reported usage of MS-CASPT2 for energetics and couplings of DNA bases’ dimers,^365^ uses a scheme^366^ that requires excitation energies for the whole dimer, which is impractical for even medium sized chromophores.

OpenMolcas fills in the gap and allows to efficiently compute Frenkel’s excitonic couplings, using multireference methods. The implemented code takes full advantage of the SEWARD module in its Cholesky-based facets^367,368^ in order to enhance memory capabilities compared to standard integrals, along with a major speed-up. The actual evaluation of the excitonic couplings is performed by means of an adaptation of the RASSI module as provider of the necessary information from the wave functions of the sought-for electronic states of each monomer. The current implementation does not account for exchange contribution, and thus, the method is valid while the monomer wave functions do not overlap. Further details on the implementation are given in ref (369). Below are showcased the capabilities of the protocol using MS-CASPT2 and MS-RASPT2 level of theory; however, any of the multireference methods implemented inside OpenMolcas can be used.

**Figure 18:**
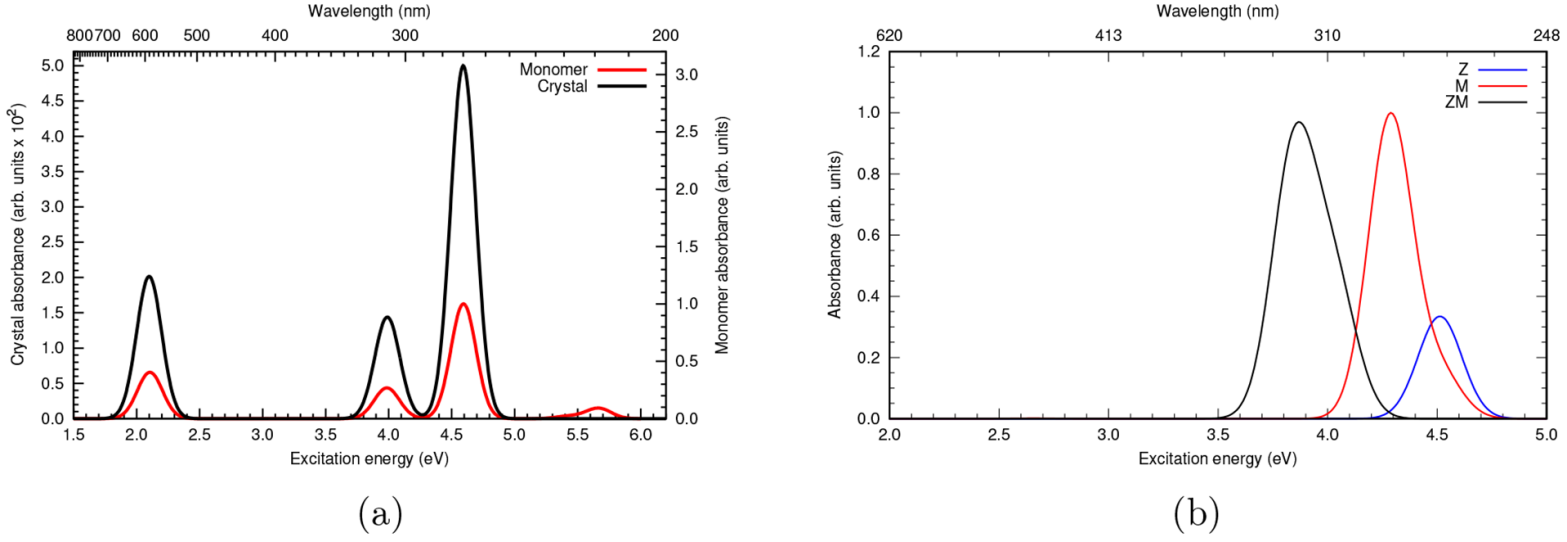
Computed excitonic spectra. (a) Excitonic absorption spectrum computed for the azulene crystal (black line) and computed absorption spectrum of an azulene monomer (red line). (b) Computed absorption spectrum of azobenzene derivatives Z (blue line) and M (red line) as separate molecules. The computed excitonic absorption spectrum of the heterodimer is given as a black line (ZM).

The first example concerns the computation of the absorption spectrum of azulene in crystal form. Azulene (a naphthalene isomer) is an aromatic molecule, differing from canonical aromatic hydrocarbons for its blue color (absorbance peak at 2.1 eV, 580 nm)^370,371^ that is unusual given the size and large dipole moment.^370^ Azulene electronic excitations are to both Rydberg and double excitation character states; hence, it is a good candidate for a multiconfigurational method. Since molecular orbital coefficients are invariant to translation, only four different spatial dispositions of the molecule were considered, later combined to evaluate all possible unique dimers to obtain the absorption spectrum of azulene crystal^372^ (Figure 18a). Although the three most absorbing (i.e., with highest oscillator strength) excitonic excited states show delocalization among monomers, as shown and discussed in the SI, crystal peaks are only very slightly red- and blue-shifted with respect to the monomer absorption (in the order of 0.05 eV). This is due to the small computed coupling values, and it implies that aggregation does not change azulene color, although absorbance is likely due to more than one molecule at a time.

The code seamlessly computes Frenkel’s excitonic couplings also for heterodimers, here exploited to investigate a heterodimer of *trans*-azobenzene and an amine-substituted derivative, which are referred to as Z and M monomer. Heterodye aggregates hold considerable research interest,^373−375^ and the here-presented heterodimer has been tested as interacting units covalently linked to DNA strand nucleobases.^376^ Azobenzene is a highly conjugated molecule, which is deemed to require a multiconfigurational treatment.^377,378^ As also experimentally noted, the heterodimer spectrum is red-shifted with respect to both monomer peaks (Figure 18b). As expected, given the short intermonomer distance with respect to the monomer sizes, such a system cannot be properly described by a dipole approximation. Indeed, the corresponding states diagram reported in the SI shows that the interaction of permanent dipoles dominates the coupling and leads to the downshift of all energy levels.

### 3.7 Ionization and Autoionization Processes

Ionization and autoionization processes are at the foundation of a number of important spectroscopic techniques to probe the properties and dynamics of molecular systems, see, e.g., ref (379) and references therein. The development of high intensity lasers and free-electron lasers with ultrashort pulses, improved synchrotron radiation sources, and more efficient electron and ion detectors have boosted the interest in such techniques and highlighted the need for reliable theoretical methods to interpret the results of the experimental measurements.

OpenMolcas allows simulating photoelectron spectra (both ultraviolet, UPS, and X-ray, XPS) at the sudden approximation level,^380,381^ enabled by the computation of Dyson orbitals.^382−385^ The existing algorithm for the computation of Dyson orbitals at the CASSCF/CASPT2 level has been revised^381^ to take advantage of full Abelian point group symmetry and correct normalization within a biorthonormal orbital basis. An interface to the Tiresia(297) B-spline code for the electronic continuum was developed, which enables the computation of accurate photoionization dynamical parameters by combining CASSCF/CASPT2 Dyson orbitals with a DFT/TD-DFT description of the electron in the continuum.^381^

**Figure 19:**
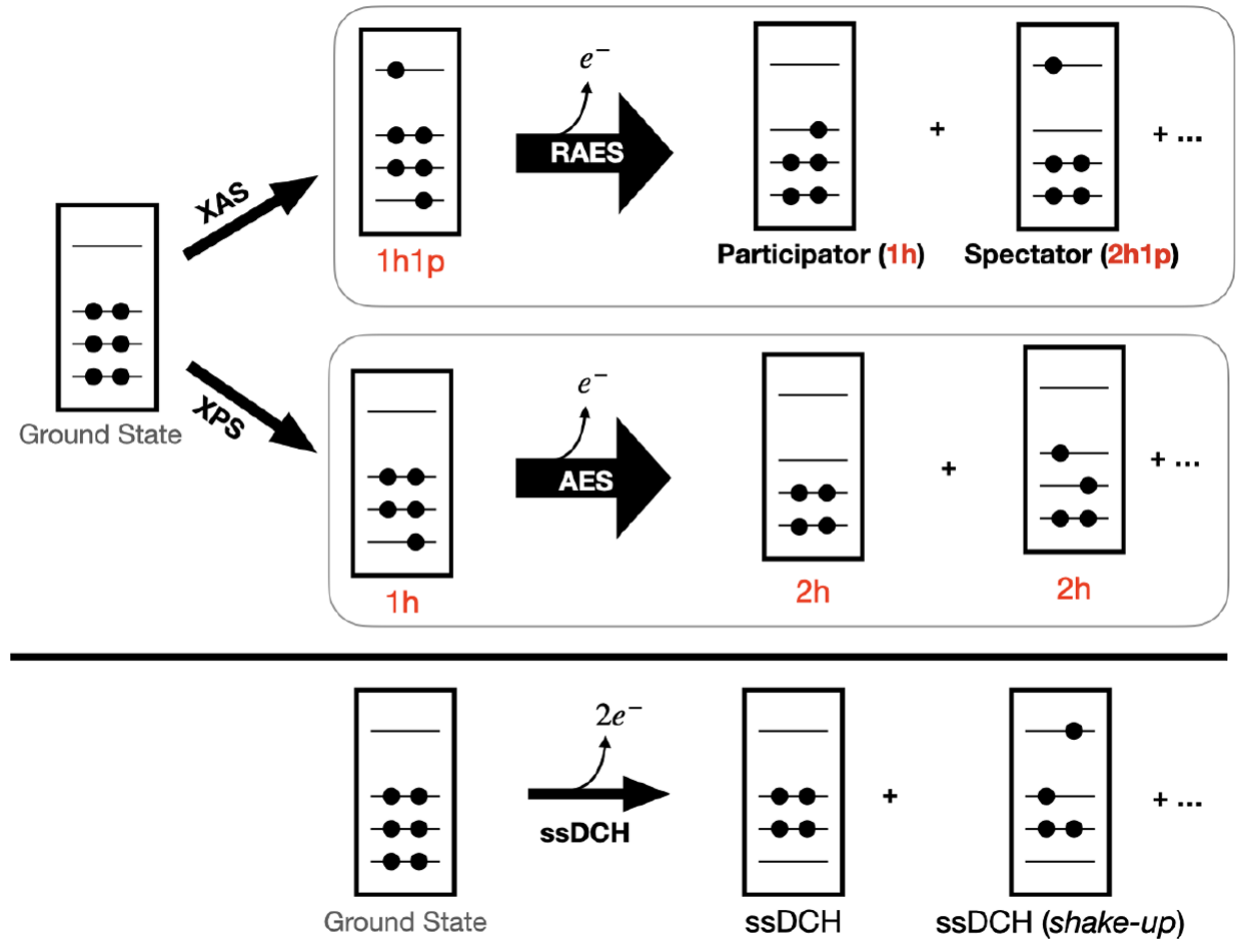
Schematic representation of the Auger–Meitner (RAES and AES) and single-site double-core-hole (ssDCH) ionization processes.

Recent developments are highlighted here on the theoretical description of single-site double-core-hole (ssDCH) ionization and Auger–Meitner electron decay (see Figure 19).

In ssDCH ionization, the multielectronic effects induced by the formation of the double core hole are greatly enhanced compared to single-core-hole states. This leads to a sharp increase in probability of events such as shakeup, making ssDCH XPS particularly suited to study relaxation and correlation effects induced by double photoionization in the inner shell. In ref (386), a multireference protocol is proposed to compute ssDCH photoelectron spectra in which the transition amplitudes are determined within the sudden approximation.

In normal Auger electron spectroscopy (AES), a core-ionized (1h) initial state decays into a manifold of doubly charged (2h) valence states of different spin multiplicity. In resonant AES (RAES), a core-excited state decays to a singly ionized state, where the outgoing electron can be either the core-excited electron, resulting in a 1h final state (participator Auger), or an inner-valence electron, resulting in a 2h1p state (spectator Auger).

The protocol^387^ to compute Auger–Meitner spectra in OpenMolcas uses the technique called *one-center approximation* (OCA).^388,389^ OCA is based on the recognition of the strongly localized nature of the initial core hole, and it amounts to the neglect of Coulomb matrix elements involving the continuum and atomic orbitals on different centers. In addition, because of the high electron kinetic energy, the continuum, expanded in partial waves centered on the core site, is approximated by the corresponding atomic one. Given the complexity and high number of final ionic states reached, OCA turns out to be adequate for an overall description of the spectral intensities in current spectra of complex molecules.^387,390,391^

**Figure 20:**
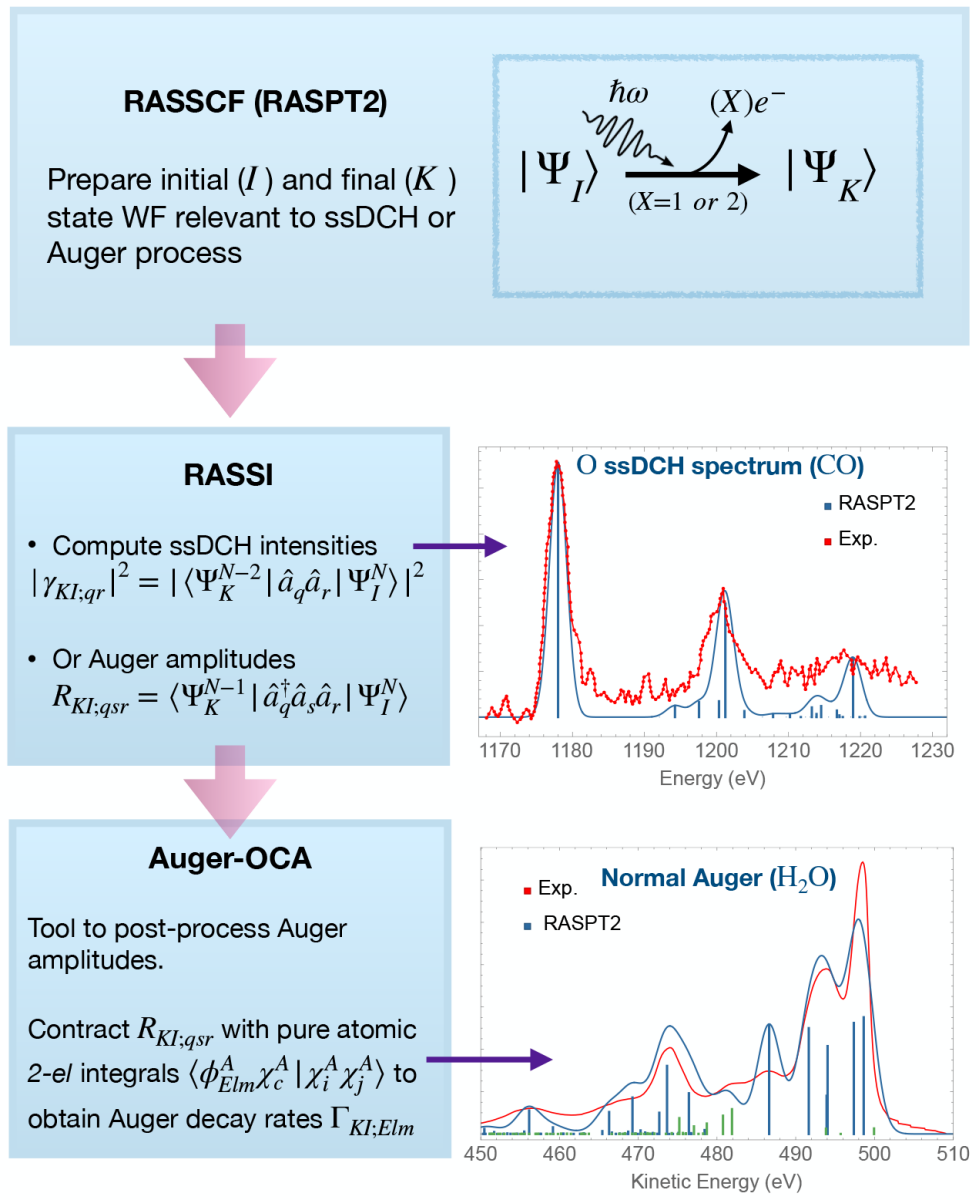
Scheme of the workflow for the calculation of the ssDCH spectral intensities in the sudden approximation and of the Auger–Meitner spectra based on the one-center approximation.

The computational steps to obtain ssDCH spectral intensities and AES/RAES decay rates in OpenMolcas are summarized in Figure 20. In both cases, the two-particle Dyson matrices corresponding to either the ssDCH intensities or to the Auger amplitudes are a key ingredient. Their computation has been implemented within the RASSI module.^386,387^

Additionally, an interface to the SCAMPI code^298^ offers an approximation level to the molecular continuum which lies in between that of OCA and Tiresia.

### 3.8 Ultrafast Electron Dynamics

**Figure 21:**
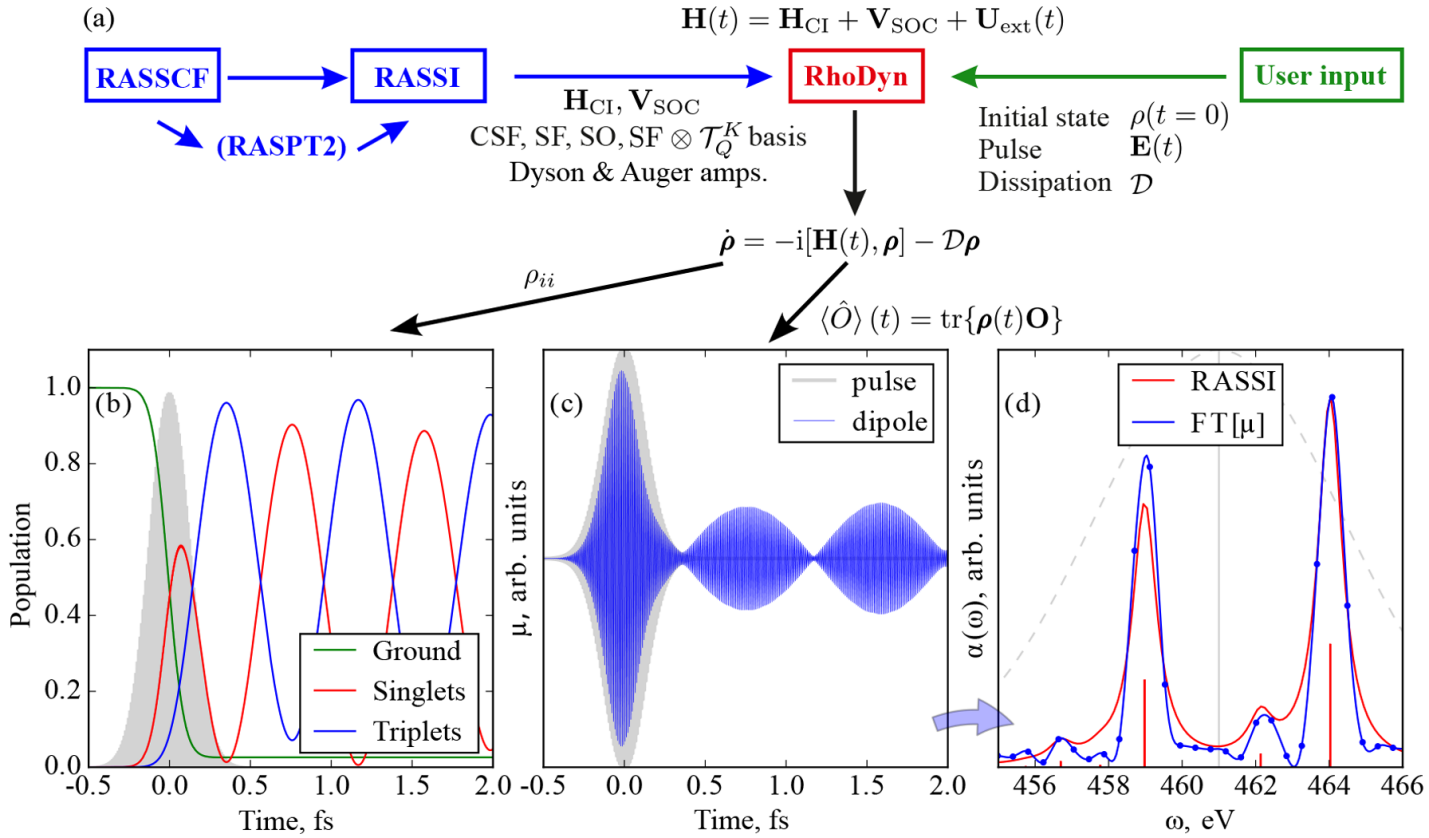
(a) Workflow and dependencies of the RhoDyn module. Here, ***H***_CI_, ***V***_SOC_, and ***U***_ext_(*t*) are the time-independent CI Hamiltonian responsible for electron correlation effects, SOC, and time-dependent external potential; for details, see ref (393). (b) Populations of ground, singlet, and triplet states of TiCl_4_ molecule after the excitation with ultrashort X-ray pulse (pulse envelope shown as a shaded gray area). (c) Response of the dipole moment for the same case. (d) Linear XAS obtained as the Fourier transform of the dipole moment (pulse in energy domain shown as gray dashed line).

Ultrafast electron dynamics can be studied within the density-matrix-based time-dependent restricted active space configuration interaction framework (ρ-TD-RASCI),^392,393^ thanks to the newly developed program module RhoDyn. Via the density-matrix formalism, RhoDyn can describe dynamics in both coherent and incoherent limits and cases in between them. Effectively, it utilizes the time-independent quantities computed in RASSCF, CASPT2, and RASSI modules to construct the Hamiltonian and recast the problem into the time domain by solving the Liouville–von Neumann equation, see Figure 21a. The user can construct the nonequilibrium initial state by specifying an initial density matrix, e.g., by populating a configuration that is not an eigenstate of the electronic Hamiltonian, or by introducing an external light field in the form of consecutive ultrashort pulses. The probabilities of ionization and autoionization (Auger decay) can be computed as described in section 3.7 and also included into consideration.

RhoDyn allows for a flexible choice of the basis for time propagation depending on the problem under study. For instance, the bases of CSFs, spin-free (SF) and spin–orbit coupled (SOC) states are possible; additionally, one can represent the spin part of the density matrix in the basis of spherical irreducible tensors (state multipoles). The use of correlated states (SF and SOC) as the basis often allows for a substantial decrease in the dimension of the problem in comparison to CSFs.^393^ In this respect, a CASPT2 correction to the Hamiltonian might be important for a better reproduction of electronic time scales, see ref (393).

The module is intended to study purely electronic dynamics when nuclear motion does not play an important role. Such an approach is advantageous to study dynamics in core-excited states since electron motion is largely isolated from nuclear effects owing to the characteristic time scales and the ultrashort lifetime of the core hole not exceeding few fs. To take the influence of the energy and phase relaxation due to vibronic interactions into account, the electronic system–vibrational bath partitioning is employed; for details, see ref (394). The user-specified dissipation superoperator  determines the details of this relaxation.

The main output of RhoDyn consists of the time-dependent reduced density matrix **ρ**(*t*); its diagonal provides occupation numbers of the basis states. For instance, Figure 21b exemplifies the evolution of spin state populations in the TiCl_4_ molecule after excitation with a short X-ray pulse. More importantly, the output **ρ**(*t*) can be used to compute the expectation value of any operator Ô. In this respect, the most prominent example is the dipole moment , see Figure 21c, as it provides access to linear (for example, X-ray absorption spectrum, XAS, in Figure 21d) and nonlinear spectra of the system. Further application examples can be found in ref (393) which describes the simulations of the linear L_2,3_ XAS, highly nonlinear high harmonic generation triggered by a strong-field infrared (IR) laser pulse, ultrafast charge migration, and spin-flip dynamics in the core-excited states^395,396^ of iron complexes triggered by an ultrashort X-ray pulse. Possible applications are not limited to these processes and may include studies of multiple ionization and other nonlinear spectra.

## 4 Gradients and Molecular Structure Optimization

OpenMolcas has recently been enhanced with two new analytic gradient options, and a molecular structure optimizer based on a machine-learning technique. This section is devoted to a brief description of these new features. The CASPT2 method was initially implemented in MOLCAS some 30 years ago. The new developments now support analytic first-order derivatives for most of the various CASPT2 versions. This extension also provides analytic nonadiabatic coupling vectors, which can be, for example, applied to minimum energy conical intersection searches. Moreover, MC-PDFT analytic gradients are now also provided for the multistate members of the family. Finally, the toolbox for exploring potential energy surfaces has been expanded with the restricted variance optimization (RVO) method. Using a surrogate model based on gradient-enhanced Kriging, the convergence of geometry optimizations is typically faster than with conventional second-order methods.

### 4.1 CASPT2 Analytic Nuclear Gradients

The development of analytical derivative theories and the optimization of molecular geometries were obvious extensions for CASPT2; however, their development was delayed by CASPT2’s complexity. The key challenge of this task is taking the partial derivative of the correlation energy in terms of wave function parameters. In particular, the formulation is made substantially more difficult using the internally contracted scheme. However, over the past 30 years, analytic derivatives have been developed in a few program packages;^397−399^ finally, the functionality is available in OpenMolcas.^400,401^

One unique characteristic of the OpenMolcas implementation is the analytic derivatives of restricted active space PT2 (RASPT2).^402^ Due to the limited numbers of holes and electrons in RAS1 and RAS3, the RASPT2 method is beneficial for large active spaces, which are impractical for CASPT2. The implementation can use either the real^208^ or imaginary^209^ level shift technique to avoid the intruder state problem. Analytic gradients in association with the σ^*p*^-CASPT2 option (see section 2.7) are not yet available.

The analytic derivatives for various multistate CASPT2 methods were also implemented,^401^ including the original MS-CASPT2,^205^ XMS-CASPT2,^206^ and the recently suggested XDW-CASPT2^203^ and RMS-CASPT2^204^ (see section 2.7). These options have been implemented in association with the resolution-of-identify option for the two-electron integrals using either external auxiliary basis sets or the so-called compact atomic CD auxiliary basis sets.^217^

**Figure 22:**
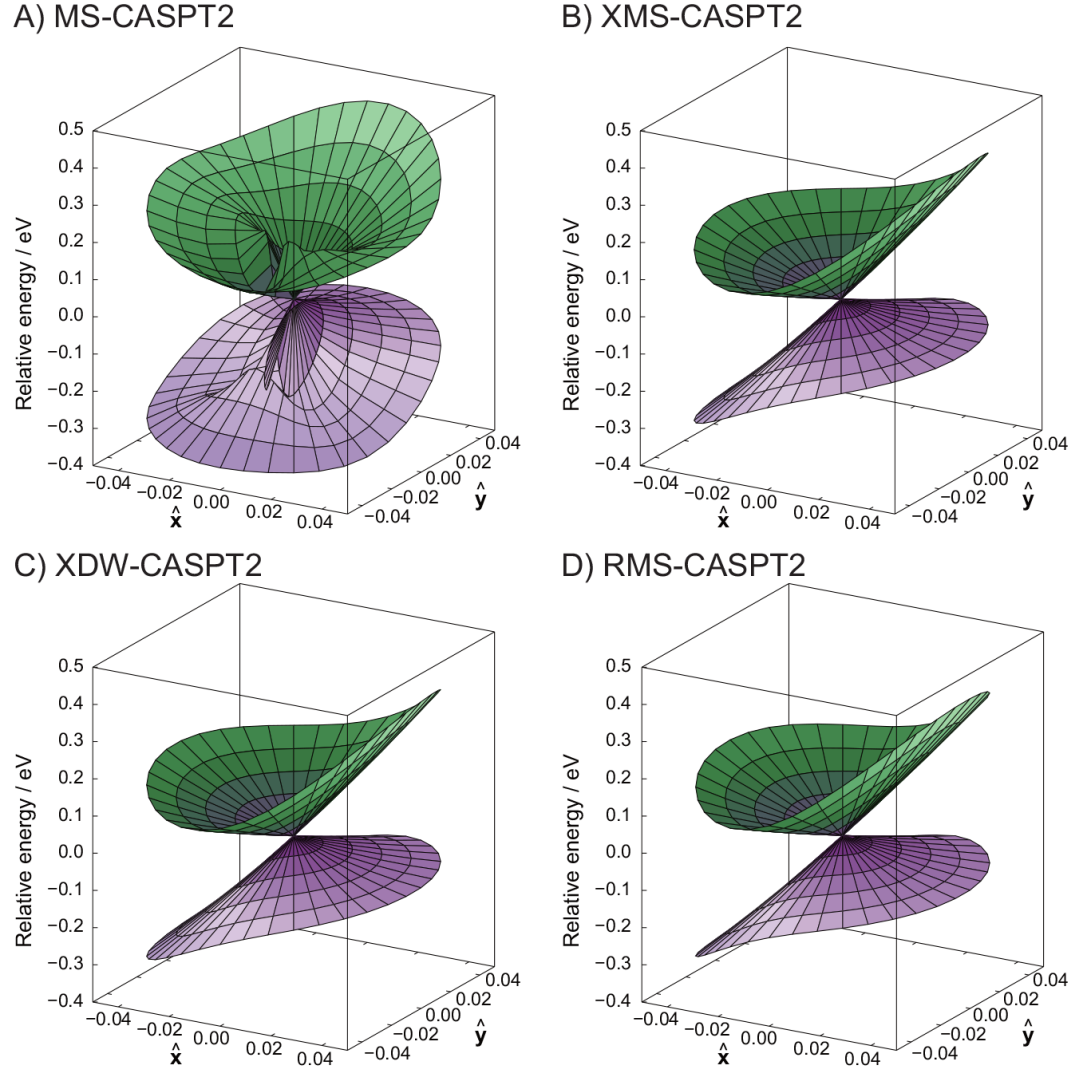
PESs around the MECI of pyramidalized ethylene optimized with (A) MS-, (B) XMS-, (C) XDW-, and (D) RMS-CASPT2 methods. Reprinted with permission from ref (401). Copyright 2022 American Chemical Society.

Here two studies are presented to demonstrate the characteristics of the newly implemented options. First, as a pilot application examining CASPT2 vs RASPT2, the excitation energies and geometrical parameters of *trans*-1,3,5,7,9,11-dodecahexaene (C_12_H_14_)^400^ were benchmarked. Using CAS(12e,12o), the number of CSFs was 2 26 512. The RASPT2 calculation was performed with a RAS(12,2,2;3,6,3) specification (see the meaning in section 2.7). Compared to the corresponding CASPT2 calculation, the error of excitation energies and geometrical parameters at the RASPT2 was 0.04 eV and 1.5 × 10^–3^ Å, respectively, with only 16% of the CSFs. In another pilot study, using the four MS-CASPT2 variants, a conical intersection (CoIn) of ethylene was located at the CASSCF level of theory and the energy variation in the branching space of the four MS-CASPT2 variants was studied.^401^ Here a smooth and slow variation is a desirable property of a well behaved MS-CASPT2 method. It is well-known that MS-CASPT2 suffers from the noninvariance character near state crossings (see Figure 22A). Clearly, MS-CASPT2 demonstrates a significant discontinuity in the vicinity of a minimum energy CoIn (MECI). The potential energy surfaces of other MS-CASPT2 variants (see Figure 22), however, are almost uniformly smooth, and these methods can be used for locating MECIs. In particular, it is emphasized that RMS-CASPT2 can be a useful substitute for MS-CASPT2.

### 4.2 MC-PDFT and MS-PDFT Analytic Nuclear Gradients

The MC-PDFT and MS-PDFT methods are briefly described in section 2.8. Here we report on the newly implemented analytic gradient options. These are implemented in OpenMolcas using the method of Lagrange multipliers.^403^ Hence, the Lagrangian of an MC-PDFT energy is expressed as,
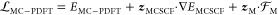
20which includes undetermined multipliers for the orbitals and CI vectors of the underlying MCSCF state or states (***z***_MCSCF_) that minimize the corresponding MCSCF electronic energy (*E*_MCSCF_). In the case of state-averaged or multistate MC-PDFT, another set of undetermined multipliers (***z***_M_) describe rotations between states within the model space. The Lagrange multipiers are determined by calculations that depend on the specific method . Therefore, MC-PDFT gradients of energies based on different MCSCF wave functions (e.g., RASSCF, GASSCF) or different MS-PDFT methods (e.g., XMS-PDFT, CMS-PDFT) each require their own implementation. On the other hand, different translated or fully translated on-top functionals do not require individual gradient reimplementations if analytic gradients for the underlying exchange–correlation functional are available.

Availability of analytic gradients for MC-PDFT calculations of different types in OpenMolcas is summarized in Table 2. Currently, OpenMolcas supports analytic gradients for MC-PDFT based on single-state^264^ and state-averaged^265^ CASSCF references, using translated or fully translated unscaled, scaled, and hybrid functionals; these gradient implementations are compatible with density fitting of the two-electron integrals (i.e., the *RICD* keyword).^266^ CMS-PDFT analytic gradients using unscaled or scaled functionals and conventional two-electron integrals are also supported.^267^ CMS-PDFT gradients with RICD and hybrid functionals are currently under development. The Lagrangian used for CMS-PDFT gradients is computed with the CI vectors of the intermediate states, so the *WJOB* keyword (see the spin–orbit coupling section in section 2.8) should not be used in CMS-PDFT gradient calculations.

**Table 2:** Availability of Analytic Gradients of MC-PDFT Energies Using Various Functional Types, Method Types, and Two-Electron Integral Types

Functional	Method	Integral keyword	Availability	Reference
Unscaled	SS/SA^a^	*NOCD*^b^	Yes	(264)
Scaled	SS/SA	*NOCD*	Yes	
Hybrid	SS/SA	*NOCD*	Yes	
Unscaled	SS/SA	*RICD*^c^	Yes	(266)
Scaled	SS/SA	*RICD*	Yes	
Hybrid	SS/SA	*RICD*	Yes	
Unscaled	CMS^d^	*NOCD*	Yes	(267)
Scaled	CMS	*NOCD*	Yes	
Hybrid	CMS	*NOCD*	No	
Unscaled	CMS	*RICD*	No	
Scaled	CMS	*RICD*	No	
Hybrid	CMS	*RICD*	No	

a state-specific or state-averaged CASSSCF-PDFT.

b conventional two-electron integrals (default).

c density-fitted two-electron integrals.

d compressed multistate PDFT.

Test calculations suggest that geometries optimized at the MC-PDFT level are similar in quality to CASPT2 optimized geometries.^264−267^ Geometry optimizations on molecules with up to 468 basis functions and a (12,12) active space have been reported.^266^ On one 3 GHz Intel Xeon Gold 6248R processor, using a coarse quadrature grid and a single thread, a single gradient calculation for this molecule was clocked at 1 h and 38 min of wall time using the default initial orbital guess and 1 h and 18 min if initialized with converged orbitals. This is more than 5 times faster than reported in ref (266), reflecting ongoing code optimization (as well as the difference between the 6248R processor and the 2.5 GHz Intel Haswell E5–2680v3 machine used previously, to which access was no longer available). These developments have facilitated practical photodynamical simulations at the MC-PDFT level, as discussed in section 6.7.

### 4.3 Molecular Structure Optimization: Restricted Variance Optimization

The location of significant points on PESs, e.g., stable structures, transition states, etc., is one of the most common tasks performed in computational chemistry. Most software packages provide tools for geometry optimization, usually based on a second-order Taylor expansion of the PES around the current structure.^404^ These conventional methods have been fine-tuned for decades, and close to optimal choices have been developed for aspects such as the selection of molecular coordinates or the update of approximate Hessian matrices. In recent years, a new class of methods have appeared that make use of the techniques popularized in the machine learning community, in particular, methods that construct on the fly a surrogate model for the PES as the optimization progresses, using for example artificial neural networks^405,406^ or Gaussian process regression (GPR).^407−409^ In OpenMolcas, one such method has been implemented, named restricted variance optimization (RVO).^410−412^ It is based on a gradient-enhanced Kriging (GEK)^413^ surrogate model, which is a GPR variant. The GEK surrogate model exactly reproduces the energies and gradients of the data points used to generate it—the previous geometries computed during an optimization—and smoothly interpolates between them. Additionally, and in contrast to conventional second-order methods, it is capable of simultaneously describing several stationary points (minima or saddle points) and converges to the true PES as the number of data points increases. The distinguishing features of the RVO method with respect to other GPR-based alternatives are the use of a Hessian model function^414^ to set the characteristic length scales of the GEK model and the restriction of the step lengths based on the uncertainty (predicted variance) of the surrogate model.

**Figure 23:**
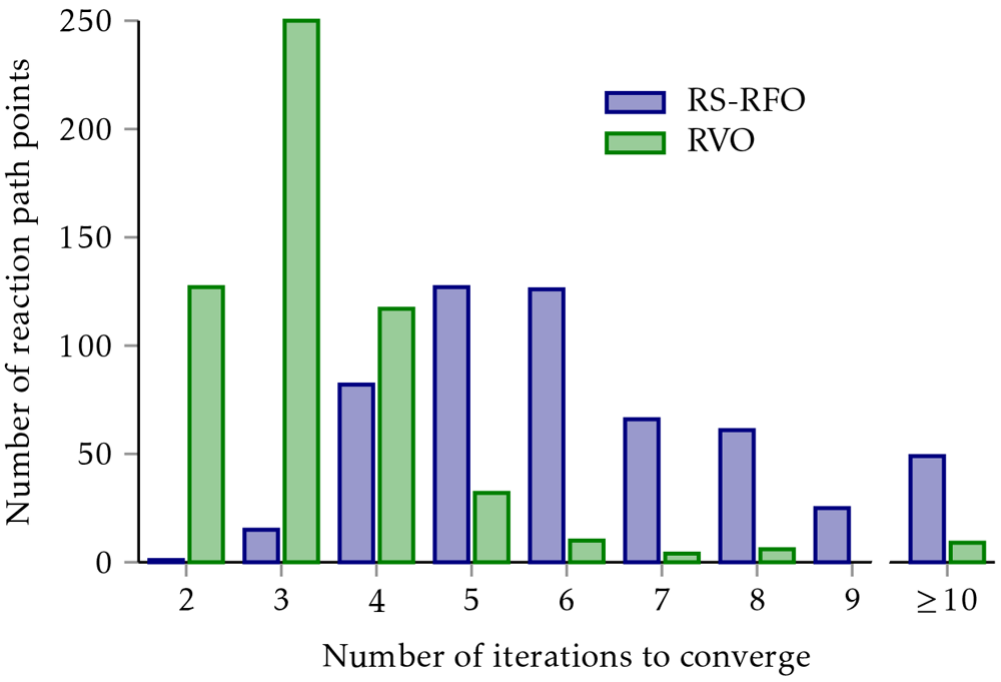
Histogram of the number of iterations needed for optimizing each reaction path point in a set of 25 reactions.^411^ The total number of iterations, obtained by summing up all the products of number of iterations times number of points, is 3730 for RS-RFO and 1993 for RVO.

The RVO method has been tested for optimizations of stable structures, transition states and reaction paths, with and without geometrical constraints, and has been shown to be a robust alternative to conventional second-order optimization methods. Even in “easy” cases, where second-order methods perform well, RVO can reduce the required number of iterations (at a negligible increase of computational cost per iteration) in around 15%. But where the method excelled was in reaction path optimizations, where it could efficiently make use of the data from previous iterations and much more easily satisfy the necessary constraints.^411^ For a set of 25 reactions, the reaction path was computed as a series of constrained optimizations, for a total of around 550 optimized structures. The overall number of iterations needed was 3730 with the conventional restricted step rational functional optimization (RS-RFO) method, and it was reduced to 1993 (a 47% decrease) with RVO. In Figure 23, a histogram of the number of optimizations that converged in a given number of iterations is presented. It is evident that most optimizations converged in 4 iterations or less with RVO, while with RS-RFO they require at least 5 or 6 iterations.

## 5 Vibrational and Vibrationally Corrected Electronic Spectroscopy

The availability of analytical gradients for some of the most advanced electronic structure methods in OpenMolcas enables improving the description of chemical systems beyond the frozen-nuclei approximation. In a first stage, a local expansion of the relevant PESs can be done based on the gradients and/or normal modes, and the effect of nuclear vibrations can be incorporated into spectroscopic simulations. The current section deals with some applications that make use of this concept, the more elaborate technique of simulating the actual time evolution of molecular structure will be discussed later in section 6.

Among the various methods to compute electronic photoabsorption spectra from first principles, the so-called nuclear ensemble approach (NEA) has gained traction in recent years, in part thanks to its conceptual simplicity and for alleviating prohibitive computational burdens. A tool is presented for predicting electronic absorption spectra in the gas phase using NEA combined with probabilistic machine learning, which opens the door to obtaining reliable spectra even with ensembles of only hundreds or even tens of sampled geometries. Moreover, exact quantum mechanical simulations for transient electronic spectroscopy within the displaced harmonic oscillator model are facilitated by an interface with the Spectron program.^415,416^ The calculation of spin dynamics from first principles with state-of-the-art methods is currently limited by the numerical evaluation of vibronic couplings. Combining the analytic CASSCF gradient facilities of OpenMolcas with the linear vibronic coupling method, a flexible interface for the fully analytic evaluation of vibronic couplings in metal complexes is introduced. In this context, a two-step approach to accelerate the evaluation of the vibronic coupling elements in the case of multiroot studies is also presented. Beyond electronic spectroscopy, purely vibrational or vibrational–rotational levels in diatomics can also be computed. Now, with the addition of LEVEL 2022, this can be done by reading analytic potentials rather than only pointwise potentials, and with an adaptive mapping procedure that greatly improves convergence. These specific applications are further explained in the following sections.

### 5.1 Nuclear Ensemble Approach for Spectral Shapes

The simulation of spectral shapes from first principles is an extremely challenging task, as it involves the simulation of excited state quantum molecular dynamics and subsequent calculation of the autocorrelation function between the ground state wave function and the time-dependent excited state one.^417−419^ While feasible (see section 6), a more affordable option is the nuclear ensemble approach (NEA).^420,421^ This time-independent method relies on several steps: (1) Obtaining the equilibrium molecular structure and possibly a local description of the PES (e.g., its associated normal modes); (2) Sampling a statistically significant ensemble of nuclear geometries around the equilibrium geometry; (3) Computing the excitation energies (Δ*E*) and oscillator strengths (*f*) for all pertinent states (roots) at each geometry; (4) Reconstructing the NEA spectrum by *phenomenologically broadening* each transition following Gaussian or Lorenztian line-shapes centered at Δ*E*, with an empirical full-width (δ) and with an area proportional to the corresponding oscillator strength *f*. It is the average of these multiple contributions what builds up the electronic spectrum.^420,421^ In this sense, the larger the number of geometries is, the more precise the spectrum *reconstruction* becomes. On top of that, using advanced multiconfigurational quantum chemistry packages such as OpenMolcas, with an efficient implementation of the CASPT2 method and atomic natural orbital (ANO) basis sets, helps to increase the accuracy of the spectra for strongly correlated problems. The NEA methodology has gained traction in recent years, as it allows to predict reliable electronic absorption and emission spectra without a prohibitive computational burden.^422−437^ With the idea of computing gas phase electronic NEA spectra, the open-source software MULTISPEC(438) was developed to carry out the steps outlined above in a semiautomated fashion.^432^ Based on OpenMolcas for the core computations of Δ*E* and *f* and for generating the Wigner ensemble of geometries (which also serve to generate initial conditions to run excited state molecular dynamics simulations, see section 6.1), it consists of a series of shell scripts that guides the user through the tasks. In its current implementation, it enables the calculation of ground state absorption spectra.^432^

Even with automated computation routines such as MULTISPEC, the total number of sampled geometries on which to perform OpenMolcas computations may be limited to a few hundreds, in the best cases, in situations requiring an expensive computational power (like in CASSCF/CASPT2) and/or dealing with complex systems (spin–orbit coupling, large number of roots, explicit solvent effects, large molecules, etc.). This scarcity of useful cases (geometries) may lead to artifacts in the reconstructed spectra if the line-width δ is not chosen properly. In particular, it should be chosen so that a trade-off between artificial vibronic features (small δ) and oversmoothing of electronic signatures (large δ) is attained. Frequently, the search for the optimal δ involves the visual inspection of the reconstructed spectra to find the compromise between under- and oversmoothing. Naturally, as it relies on a nonsatisfactory subjective perception, there is a growing interest in applying machine learning (ML) techniques (i.e., objective criteria) to adequately reconstruct the electronic NEA spectra for small data sets.^430,434,435,439,440^ Whereas these approaches lead to broadly satisfactory results, all the models reported to date still rely on the use of the phenomenological broadening underpinning the NEA formalism. To circumvent its use and, in turn, the selection of a bandwidth δ altogether, a novel approach based on the use of Gaussian mixture models (GMM), an unsupervised ML algorithm commonly used for clustering, classification, and density estimation tasks, was reported recently.^441^ The key for this approach is to mathematically transform the conventional equation for the reconstruction of NEA spectra to express it in terms of the GMM parameters that model the distribution of the pairs  for each transition. For small data sets (*N*_s_ < 400 geometries), GMM-NEA, as the methodology is dubbed, systematically outperforms alternative ML solutions in reconstructing both the full spectrum and the different transitions band shapes, especially in the band edges. A fully functional open-source implementation of GMM-NEA based on the programming language R is available,^442^ and is fully compatible with MULTISPEC.

**Figure 24:**
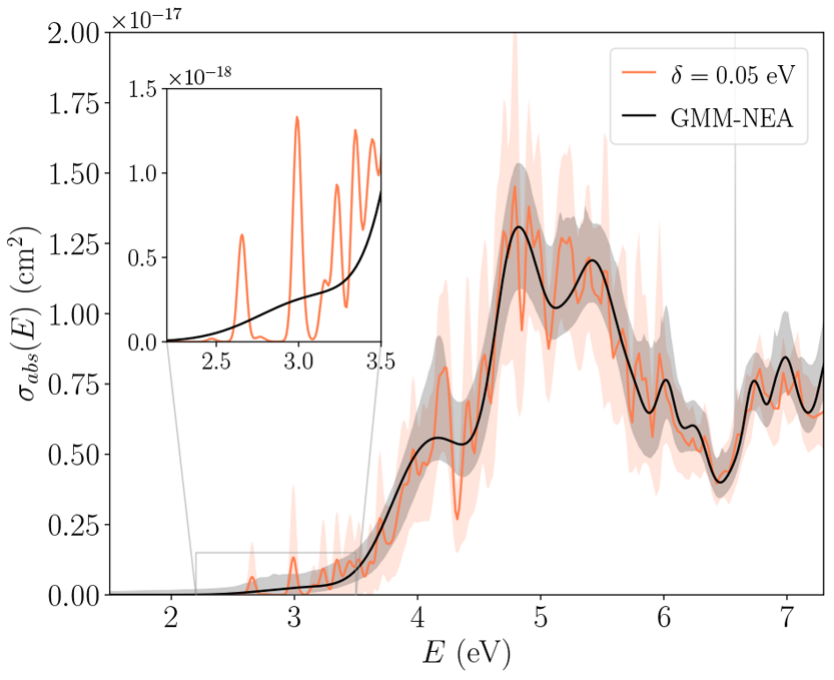
Electronic absorption cross section spectrum σ_abs_(*E*) of HgBrOOH reconstructed from 200 geometries using a unique empirical bandwidth of δ = 0.05 eV for all transition (orange line) and GMM-NEA (black line). The shaded areas represent the 95% confidence intervals. The inset details the contribution of both spectra in the region overlapping with the solar radiation (not shown).

As an example, Figure 24 shows the ground state absorption spectrum of HgBrOOH, a compound relevant in the atmospheric chemistry cycle,^443^ computed combining MULTISPEC and GMM-NEA. In particular, the values of Δ*E* and *f* were obtained for 79 transitions and 200 geometries using CASSCF/CASPT2 calculations with spin–orbit states. For comparison purposes, the originally reported computed spectrum^443^ is displayed in Figure 24. The particular choice of empirical broadening in the latter case (δ = 0.05 eV for all states) resulted in the presence of apparently strong and quite resolved bands around 2.6 and 3 eV. The absorption at these bands could play a role in the photolysis reaction of this compound, as they overlap with a region of strong solar radiation in the troposphere. In contrast, the GMM-NEA spectrum is, as expected,^441^ significantly smoother and, whereas there is indeed absorbance around 2 to 3 eV, the bands are not as resolved as previously predicted. The GMM-NEA absorption spectrum can be now used, for instance, to determine the photolysis rate (*J*) as , where ϕ(λ, *T*) is the photolysis quantum yield as a function of wavelength and temperature, σ_abs_(λ) is the absorption cross section spectrum, and  is the solar spectral actinic flux (in quanta s^–1^ cm^–2^ nm^–1^) at the altitude of interest as a function of solar zenith angle θ and wavelength. A value for *J* of 0.025 s^–1^ is obtained in such manner for HgBrOOH (see details in ref (441)). This type of computations allows to evaluate the implications of solar light chemistry in the atmospheric cycle of Hg.^443−445^

### 5.2 Exact Simulations in the Harmonic Approximation

The most accurate approach for nonlinear spectra simulation relies on a quantum mechanical description of wave packet coherences created by the light–matter interaction. When the PESs on which the nuclear wave packet evolves are represented in the approximation of the multidimensional uncoupled displaced harmonic oscillator (DHO), employing a unique set of normal modes and frequencies for all the electronic states (normally computed in the electronic ground state), analytical equations can be derived by means of second-order cumulant expansion of Gaussian fluctuations (CGF), formally exact in the case of adiabatic dynamics.^415^ These equations can be parametrized with quantum mechanical (QM) data from a single geometry: transition energies and dipole moments, normal modes and frequencies, and energy gradients.

The program iSpectron(446) was designed to parse the QM data from OpenMolcas (among other QM software) and interface it to Spectron,^415^ a platform for simulation of coherent nonlinear optical spectroscopy of single molecules and their aggregates in the DHO/CGF framework. The OpenMolcas–Spectron interface allows to compute the QM quantities with multiconfigurational wave function theory methods such as RASSCF/RASPT2. Employing such methods allows to compute many excited states (those in which the relevant nonadiabatic events occur, as well as the spectroscopically relevant higher-lying ones) and to evaluate the dipole coupling between them. Both homogeneous and inhomogeneous broadening can be taken into account. iSpectron possesses an ample body of tools for displaying and analyzing the spectra. For a detailed overview of the capabilities of iSpectron interested readers are referred to ref (446); the iSpectron code is available free of charge on GitHub.^447^

Despite its simplicity, the DHO/CGF approach can produce rather accurate spectra with a manageable computational effort. It is especially suited for rigid molecules (e.g., fused ring systems, transition metal complexes) and for events taking place on an ultrashort time scale (subps) with little geometrical changes. The availability of analytical expressions constitutes a great advantage as it allows to explore at negligible cost the effect of pulse parameters (bandwidth, duration, polarization), temperature, and environment coupling strength on the spectra. The nonadiabatic dynamics can be introduced either phenomenologically via rate-equations (which can be parametrized by fitting experimental data) or by reading in populations from (numerical) quantum dynamics (QD) simulations. A sample application of the DHO/CGF model and comparison with a molecular dynamics approach will be presented in section 6.5.

### 5.3 Analytic Linear Vibronic Couplings for Molecular Magnets

The accurate and efficient modeling of vibronic coupling in metal complexes, i.e., the mixing of electronic states beyond the Born–Oppenheimer approximation induced by nuclear motion, is crucial for the theoretical investigation of a wide range of physical processes in spectroscopy, quantum information, and magnetic memory applications. The analytic linear vibronic coupling (LVC) method implemented in the SHARC program^172,270^ is already available for nonadiabatic dynamics through its OpenMolcas interface (see section 6.3 below). However, the molecular magnetism community has been relying on vibronic coupling constants derived numerically through finite difference methods based on single-point CASSCF electronic structure calculations at distorted geometries for the modeling of the vibronically driven electronic spins dynamics.^449−452^ As simulations become more accurate, accounting for many thousands of degrees of freedom in the condensed phase, the numerical approach has practical and theoretical limitations. First, the number of single-point calculations grows linearly with the number of nuclear degrees of freedom and quickly becomes infeasible, both in terms of computational cost and file storage. Second, the large range of coupling strengths from weakly coupled environmental vibrations to strongly coupled intramolecular vibrations, poses well-known numerical challenges to finite difference schemes.

The LVC method circumvents these problems by calculating the electronic response to nuclear distortion in the form of molecular gradients and NACs of the relevant electronic states based on one single-point CASSCF calculation. At its core, the LVC method introduces vibronic coupling effects through a truncated diabatic expansion of the electronic Hamiltonian linear in the nuclear degrees of freedom, inducing a geometry-dependent unitary mixing of the equilibrium CASSCF eigenstates. Subsequently, the spin-free eigenbasis is augmented with nuclear-dependent spin–orbit coupling and the total transformation is employed to compute geometry-dependent matrix elements of any operator computed at equilibrium geometry. For a more detailed discussion of the LVC methodology, the reader should consult refs (172), (185), and (453).

**Figure 25:**
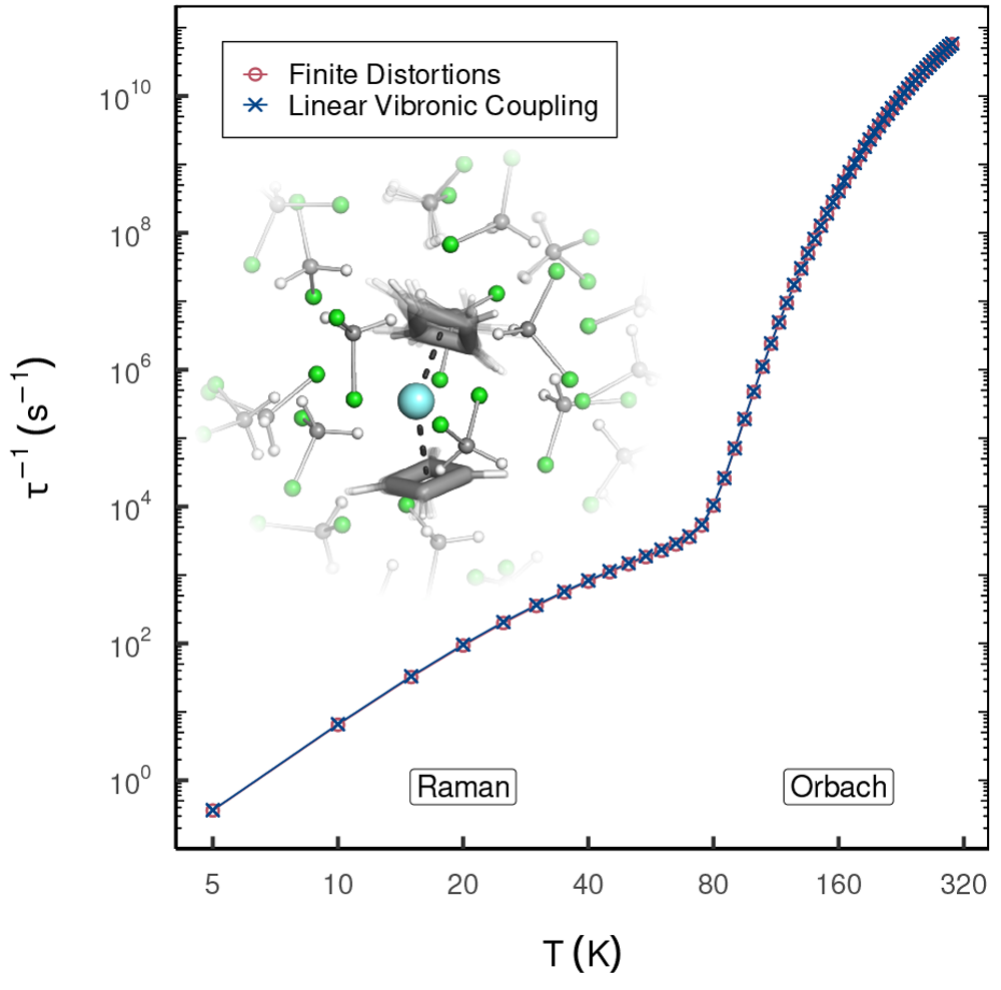
Comparison of temperature dependent magnetic relaxation rates between analytically evaluated (LVC) vibronic couplings and those derived by numerical differentiation involving single-point calculations at distorted geometries.

As an example application of the LVC-based method, ref (454) showcases a study of the magnetic relaxation in a proposed bis-cyclobutadienyl Dy^III^ single-molecule magnet (SMM) solvated in dichloromethane (DCM).^454^ Combining the OpenMolcas infrastructure for the computation of density fitting molecular gradients and NACs at the CAS/RASSCF level of theory,^455^ and the highly flexible analytical LVC methodology, fully analytic vibronic coupling constants were evaluated in the form of crystal field parameter derivatives along normal mode coordinates. This allowed to obtain magnetic relaxation rates which almost exactly match finite difference-derived couplings (Figure 25), demonstrating the general applicability of the LVC method for studying vibronic coupling in metal complexes for a wide range of cases in spectroscopy and magnetism.

The analytic LVC-based differentiation algorithm is implemented as part of the Python packages spin-phonon_suite and angmom_suite, freely available from the PyPI repository, and it is interfaced with the OpenMolcas output. The general usefulness of this implementation extends beyond its application to magnetic relaxation in lanthanide complexes. While the crystal field Hamiltonian is appropriate to describe the electronic states of the ground multiplet of lanthanide complexes, it is not the best choice in other cases. However, this method is equally applicable to other spin Hamiltonian parametrizations or to circumvent model Hamiltonians entirely and compute the bare matrix elements of the vibronic coupling derivatives. Furthermore, with the advent of CAS/RASPT2 gradients and NACs in OpenMolcas (see section 4.1), the implementation is transferable to systems featuring strong dynamical correlation. From a computational standpoint, this LVC-based method generally shows desirable computational performance compared to finite difference-derived couplings, especially when many environmental degrees of freedom are included into the calculation. For each spin multiplicity, the LVC model Hamiltonian is parametrized by  gradients and  interstate NACs. The computation of finite difference derivatives for *N*_atoms_ on the other hand requires 2(3*N*_atoms_ – 6) single-point calculations. Hence, the analytic method becomes hugely beneficial in the case of systems which feature a medium size metal complex embedded in a large electrostatic environment (i.e., as described by point charges) such as in solvated systems or true crystalline solids.

### 5.4 Two-Step Acceleration of the Analytic Evaluation of the Vibronic Couplings for Multi-Root Systems

The computation of vibronic couplings is of special importance for describing photoluminescence, molecular dynamics, and magnetic relaxation of SMMs. OpenMolcas package allows the computation of molecular gradients for certain computational methods, such as SCF, DFT, CASSCF, etc., using either numerical or analytic expressions. In particular, multiconfigurational computational methods based on CASSCF/RASSI/SINGLE_ANISO proved quite helpful in studies of SMMs over the past years, in particular for the evaluation of magnetic axiality, parameters of the crystal field *B*_*k*_^*q*^(*J*), static magnetic properties. Naturally, evaluating molecular gradients for this computational approach is of special importance for advancing computational predictions in this area, opening the gate for describing the interaction between crystal vibrations and electronic states, which is relevant for predicting magnetization relaxation times under various conditions. Since most performing SMMs are based on lanthanides, an accurate description of the low-lying energy structure involves, as a prerequisite, the mixing of a large number of spin states by spin–orbit coupling in RASSI. In this respect, the computation of vibronic couplings ∂*B*_*k*_^*q*^(*J*)/∂*Q*_α_^*A*^ at the same level of accuracy requires the evaluation of the same amount of electronic gradients for all the excited states involved in the spin–orbit coupling, as well as all the NACs between all these roots. As such, the problem scales as *N*(*N* + 1)/2 (quadratically), where *N* stands for the number of spin-free states included in the spin–orbit mixing. The current implementation in OpenMolcas allows the evaluation of molecular gradients and NACs using analytic expressions;^455,456^ however, the existing implementation was not quite optimal for multiroot state-averaged CASSCF wave functions. The code performance review pointed out a significant amount of redundant calculations, repeated for each evaluation of gradient or NAC. In the original OpenMolcas implementation, for each calculation of molecular gradient and NAC, the evaluation of molecular electron repulsion integrals (ERI), computation of Fock matrices for inactive orbitals, etc., were undertaken. Since all the states of interest are obtained in the same SA-CASSCF calculation and written based on the same molecular orbitals, all molecular ERIs and inactive Fock matrices are *identical* for all molecular gradients and NACs and, therefore, need to be evaluated only once. Herein, a two-step approach has been implemented for the analytic gradient evaluation where the ERIs, inactive Fock matrices, and other related intermediate matrices common for all required gradients and NACs, are evaluated in the first step. Subsequent evaluations of the molecular gradients for the excited states and NACs use the precomputed values. This technical trick allows for a significant speedup of subsequent MCLR steps and also helps to reduce, in part, the large input/output (I/O) operations in this process. The speedup increases with the computed molecular size and the basis set used. Table 3 shows some relative timings for the comparison of the two compounds.

**Table 3:** Relative Timings (in s) for the Evaluation of All Molecular Gradients and NACs for Two Compounds: Dy–bbpenCl (64 Atoms) and Co(acac)_2_ (35 Atoms)^a^

Basis Set	MCLR (step 1)	MCLR (step 2)	ALASKA	Total (original)	Total (two-step)	Speedup (%)
Dy-MB, 250 bf	604	163	2575	734712	633445	13.8
Dy-VDZP, 478 bf	5240	688	9405	3384522	2338250	30.9
Dy-VTZP, 850 bf	56691	4927	72861	29938122	18037329	39.8
Co-MB, 116 bf	17	8	242	14278	13800	3.3
Co-VDZP, 286 bf	263	34	1120	76274	63942	16.2
Co-VTZP, 601 bf	3751	236	6217	550590	361016	34.4

a Dy–bbpenCl was computed with CAS(9,7), 21 roots *S* = 5/2 were optimized; Co(acac)_2_ was computed with CAS(7,5), 10 roots *S* = 3/2 were considered. The MCLR and ALASKA columns refer to the calculation of a single gradient or NAC vector, while the “Total” columns refer to the total time for all *N*(*N* + 1)/2 of them (*N* = 21, 10). The expected saving is approximately *N*(*N* + 1)/2 times the “step 1” column.

A collection of scripts setting the environment and automatization of OpenMolcas calculations for various tasks, like CASSCF/RASSI/SINGLE_ANISO, CASSCF/CASPT2/RASSI/SINGLE_ANISO as well as the evaluation of the analytic or numerical vibronic couplings for any molecule is done in a parallel fashion efficiently, even on a single multicore node, with limited scratch space and memory. Among the most important features is the automated setup of all required inputs, parallel execution of calculations of different spin states, parallel per-root execution of the CASPT2, and parallel evaluation of molecular gradients and NACs. All these tasks are reusing the common ERIs or RICD integrals and other files as much as possible, without duplication or making redundant copies. The scripts are available free of charge on GitLab.^457^

### 5.5 Vibrational–Rotational Levels for Diatomics

The VibRot module, included in MOLCAS since its first versions, has allowed the calculation of roto-vibrational levels supported by a potential between a pair of atoms, along with various related properties. VibRot only allows the user to input a point-wise potential. The point-wise potential is then subjected to spline interpolation. Nowadays many extremely accurate potentials are available in analytic form,^458−467^ due to meticulous fitting to high-precision spectroscopic experiments, that represent a better alternative to the spline interpolation. One has to also be *extremely* careful with splines, particularly with spacing between the points that are provided for interpolation, because it is very easy to obtain spurious extrema or wiggles when doing spline interpolation. Therefore, whenever an analytic potential is available, it would be ideal for OpenMolcas to be able to find roto-vibrational levels directly from the analytic potential.

Another shortcoming of VibRot is that all calculations need to be converged with respect to three parameters: the minimum and maximum interatomic distances between which the Schrödinger equation is solved numerically (*R*_min_ and *R*_max_) and the discretization step (Δ*R*). Testing needs to be done to ensure convergence is achieved as *R*_min_ → 0, Δ*R* → 0 and *R*_max_ → *∞* (*R*_min_ = 0 is problematic because potentials tend toward + *∞*). An adaptive mapping procedure^468,469^ can be used to map the radial variable *R* → *Y*(*R*) so that the domain *R* ∈ [0, *∞*] becomes *Y* ∈ [0, 1]. Convergence still needs to be ensured for *Y*_min_ → 0, Δ*Y* → 0, but *Y*_max_ can be set to equal 1. Successfully implementing such an adaptive mapping procedure in a working open-source code was a long-time dream of R. J. LeRoy, as described in the final paragraph of ref (470). A well-tested version of LEVEL(471) that successfully applies this mapping procedure and overcomes the shortcomings mentioned in ref (470) is now available in OpenMolcas, along with other augmentations to the LEVEL code base, including its integration with the rest of the package.

Table 4 shows the highest vibrational quantum number, *v*_max_, found for the electronic ground state of each of three different isotopologues of BeH, the ground state of the most common isotopologue of N_2_ and the lowest-lying triplet state of the (6,6) isotopologue of Li_2_. The value of *v*_max_ = 9 for ^(6,6)^L*i*_2_(*a*) was reported in the 2014 experimental study published in ref (472), and Table 4 shows that LEVEL 2022 (now available in OpenMolcas) successfully agrees with this experimental report. Contrarily, VibRot is unable to correctly determine *v*_max_ = 9 for this potential, even with the maximum number of values (500) of *R* provided. Attempting to converge the results with this potential highlights the benefits of the adaptive mapping procedure, which eliminated the need for converging with respect to larger and larger *R*_max_ values, which was not needed with LEVEL 2022 but indeed was needed for VibRot.

**Table 4:** Vibrational Quantum Number for the Highest-Energy Vibrational Level, *v*_max_, Found with LEVEL 2022 and VibRot for Various Electronic Potential Energy Curves

	^9^BeH(X)	^9^BeD(X)	^9^BeT(X)	^14,14^N_2_(X)	^6,6^Li_2_(a)
LEVEL 2022	13	18	20	64	9
VibRot	13	17	20	64	13

Sample input and output files for all calculations presented in Table 4 can be found in the LEVEL_2022/test_suite folder of ref (471).

## 6 Ab Initio Molecular Dynamics

The OpenMolcas environment has internal modules for classical and nonadiabatic dynamics, namely, DYNAMIX and SURFACEHOP; it is also interfaced to several external programs, for example to Tinker, for options including quantum mechanics/molecular mechanics (QM/MM) simulations, and to SHARC and Quantics for nonadiabatic dynamics. In this section six new features are described, which include new tools, improvements of existing modules and interfaces, and some details in association with nonadiabatic dynamics using analytical MC-PDFT gradients.

First, several methods for generation of initial conditions have been implemented. These range in complexity from random velocities based on a Boltzmann distribution to more complex methods based on a Wigner distribution. These options have been incorporated through an easy-to-use Python script. Second, improvements to the SURFACEHOP module for surface hopping dynamics within OpenMolcas are detailed. SURFACEHOP uses the Hammes-Schiffer–Tully scheme to avoid calculation of NAC vectors by use of the wave function overlap. The approximation of this overlap matrix has been investigated and improved, resulting in more accurate and reliable simulations. Third, the section describes the newest implementation of the surface-hopping molecular dynamics SHARC code, which combined with OpenMolcas allows for very efficient–from picoseconds to nanoseconds long–simulations using parametrized potential energy surfaces and ML. Fourth, the COBRAMM platform for simulating transient electronic spectroscopy from first-principles as interfaced with OpenMolcas is presented. COBRAMM(473) is a program package for simulations within the mixed quantum–classical (trajectory-based) approximation. Some comparisons of the use of this interface vs iSPECTRON (see section 5.2) are also presented here. Fifth, the OpenMolcas–GAMESS-US/Tinker interface for state-averaged spin-restricted ensemble-reference Kohn–Sham nonadiabatic molecular dynamics is presented and an example of the approach is given as it is applied to a molecular motor. Finally, the use of the MC-PDFT method for nonadiabatic dynamics in association with the SHARC–OpenMolcas interfaces is described.

### 6.1 Initial Molecular Dynamics Conditions

Three initial condition generation methods have recently been added to OpenMolcas. These include (1) normal mode sampling (NMS) based on a classical Boltzmann distribution, (2) a Wigner distribution for the ground vibrational state, and (3) an analytical solution for a Wigner distribution with thermal dependence.

Each of these methods require knowledge of the normal mode motion of the system of interest. Therefore, the first step is to perform a Hessian calculation using OpenMolcas. The dynamixtools.py script found within the Tools/dynamixtools subdirectory can then be used with the vibrational information to generate initial conditions for running molecular dynamics. Below the two different distributions are discussed, and then some benchmark results are presented.

First, normal mode sampling is one of the newly implemented methods for initial condition generation.^474−476^ Here the phase space of each normal mode is assumed to be classical in nature, and the positions (*Q*_*i*_) and momenta (*P*_*i*_) are sampled through a random phase of the normal mode vibration at *t* = 0 shown in eqs 21 and 22, respectively:
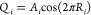
21
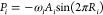
22The amplitude (*A*_*i*_) of the motion is determined from the vibrational energy available to the normal mode (*E*_*i*_) and the vibrational frequency of the mode (ω_*i*_), i.e., . In the present implementation, the energy available to each vibrational mode is independently sampled from a classical Boltzmann distribution. Once the normal mode positions and momenta are obtained, they can be transformed into Cartesian coordinates (*x*_*i*_, *p*_*i*_) using the eigenvectors of the normal mode (*L*_*i*_) and the mass matrix (*M*) as in eqs 23 and 24.
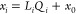
23
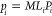
24Spurious center of mass translation and rotations often arise during the coordinate transformation step. After sampling all vibrational modes, the center of mass motion is removed, and the remaining atomic momenta and displacements are rescaled to match the total vibrational energy of the system (*∑E*_*i*_). The final velocities and positions obtained from the dynamixtools.py script can then be used directly with the OpenMolcasDYNAMIX module for molecular dynamics simulations.

Second, two sampling methods employing Wigner distributions have been implemented. Unlike NMS which assumes a classical description of the normal modes, this method produces a QM distribution.^477^ Here the momenta and positions are sampled independently, and their resulting probability (*W*_*i*_) is determined. Two methods of sampling from a Wigner distribution were considered: (1) a distribution for the ground vibrational state and (2) a distribution with thermal dependence.^475,476^ For a molecule in the ground vibrational state, the analytical solution for the probability is given by eq 25.
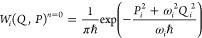
25The sampled momenta and positions are either accepted or rejected by comparing their corresponding probability to a random number cutoff. If accepted, eqs 23 and 24 are used to transform them to Cartesian coordinates. The analytical solution for a Wigner distribution with thermal dependence was also determined by Wigner and co-workers.^477^ Here the temperature dependence originates from α_*i*_(*T*) in eq 26, and the corresponding probability is given by eq 27.
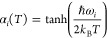
26
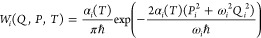
27If the probability is accepted the momenta and velocities are then transformed to Cartesian coordinates using eqs 23 and 24.

**Figure 26:**
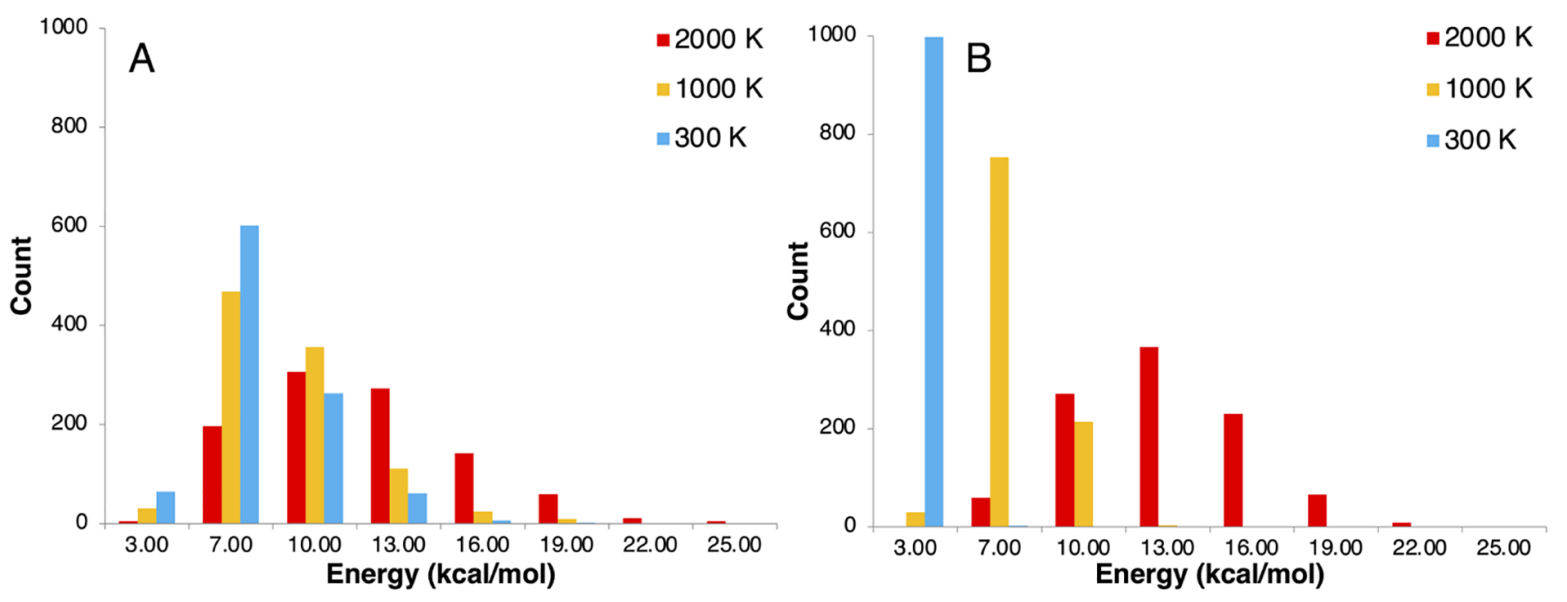
Energy distributions from 1000 initial conditions relative to the optimized S_0_ energy from using (A) a Wigner distribution with temperature dependence and (B) classical NMS.

Finally, formaldehyde (H_2_CO) in the gas phase was used to demonstrate how the newly implemented methods can produce different energy distributions. First H_2_CO was optimized in the ground state using 2-SA-(10,9)-CASSCF/6-31G*, and the normal modes were determined at the same level of theory. The ground state energy distributions from NMS and Wigner sampling with thermal dependence were determined using the energies of 1000 initial conditions relative to the optimized ground state energy (Figure 26).

Comparing the results in Figure 26 shows that at a given temperature Wigner sampling produces distributions which are both broader and centered at higher energies than those from NMS. These properties arise from maintaining the quantum distribution of the molecular system,^478^ in particular the inclusion of zero-point energy and quantizing the phase space density.^478,479^ These effects can be quite substantial for systems containing high-frequency vibrations where quantum effects are important.^479^ These results highlight that care needs to be taken when choosing an appropriate distribution for generating initial conditions.

### 6.2 Improved Nonadiabatic Coupling for Trajectory Hopping

Interfaces to OpenMolcas exist for many trajectory surface hopping (TSH) programs, allowing for a wide range of TSH simulations.^480−483^ The SURFACEHOP module within OpenMolcas can also be used to perform semiclassical nonadiabatic dynamics, specifically TSH following the “Tully fewest switches approach”.^484,485^ Running TSH dynamics directly within OpenMolcas has several benefits: increased speed thanks to reduced read/write, avoided risk of precision loss from reading printed quantities, and better future proofing with fewer potential points of failure. While SURFACEHOP is not new to OpenMolcas,^9^ recent work has improved the accuracy and reliability of TSH simulations carried out using this module.

The SURFACEHOP implementation of TSH employs the Hammes-Schiffer–Tully (HST) approximation.^486^ It relies on time-derivative couplings (TDC) and avoids the bottleneck of calculating NACs using ⟨ϕ_*i*_|*∂ϕ*_*k*_/*∂t*⟩ = ∂***R***/*∂t* ·⟨ϕ_*i*_|*∂ϕ*_*k*_/∂***R***⟩ with ϕ_*i*_ and ϕ_*k*_ electronic adiabatic states, ***R*** the nuclear coordinates, and *t* the time variable.^487^ Within HST, the required TDC can be approximated as , using only the wave function overlap *S*_*ik*_ = ⟨ϕ_*i*_(*t*)|ϕ_*k*_(*t* + Δ*t*)⟩ with Δ*t* the time step. The main change in the newest version of SURFACEHOP is how this overlap matrix *S*_*ik*_ is evaluated. Previously the scalar product of the configuration interaction vectors (CIVec) was used: , with {*C*_*i*/*k*_^*n*^} the expansion coefficients in the CSF basis. This approximation, used in several other applications,^488−491^ neglects changes in orbital composition and ordering between timesteps. It is rationalized for TSH by the limited orbital changes between short (subfs) timesteps. However, for active space based methods such as CASSCF, an issue arises due to the invariance of properties with respect to orbital ordering within the active space.

**Figure 27:**
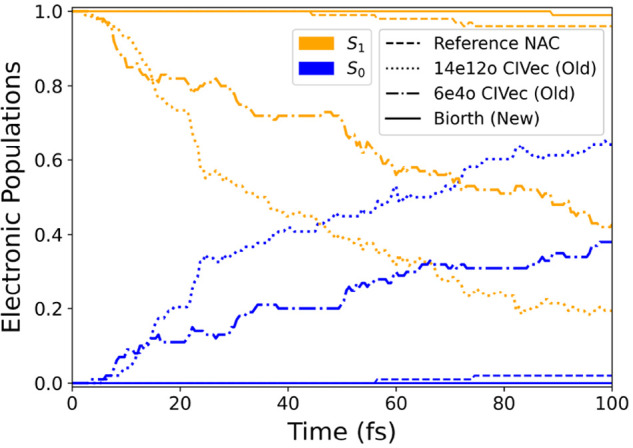
Trajectory surface hopping electronic populations upon S_1_ excitation of *trans*-azobenzene. The original CIVec scheme is shown for large and minimal active spaces, while the new Biorth and reference nonadiabatic coupling schemes are with the large active space.

When running TSH using the RASSCF module, the orbital ordering within the active space can change between timesteps, leading to unphysical values of *S*_*ik*_ using the CIVec approach. Figure 27 gives an example of an unphysical simulation which arises from this issue: the fs S_1_ → S_0_ decay seen for the S_1_ excitation of *trans*-azobenzene using CIVec (dotted line) is incompatible with both reference TSH simulation using the NAC (dashed line) and the experimental S_1_ lifetime of 13 to 16 ps.^492,493^ The influence of active space size on likelihood of reordering is shown by the improvement when reducing from 14e12o to a minimal 6e4o active space (dashed-dotted line).

In order to account for the changes in orbital mixing and order between timesteps, the new version of SURFACEHOP (Biorth) uses the transformation into a biorthonormal basis^494^ through the RASSI module. Exploiting the fact that this process is extremely efficient for CASSCF, there is almost zero additional cost to such TSH simulations.^495^ A phase correction is also added to track and fix the global phase of the wave functions, which can vary arbitrarily between timesteps. For back-comparison the old CIVec scheme is still available through use of the *NoRASSI* keyword. The new default Biorth method (solid line) clearly fixes the unphysical behavior seen for *trans*-azobenzene. Upon benchmarking of several other reactions, SURFACEHOP reliably reproduces, at lower computational cost, TSH results obtained using alternative methodologies.^496^

### 6.3 Efficient Nonadiabatic Dynamics

**Figure 28:**
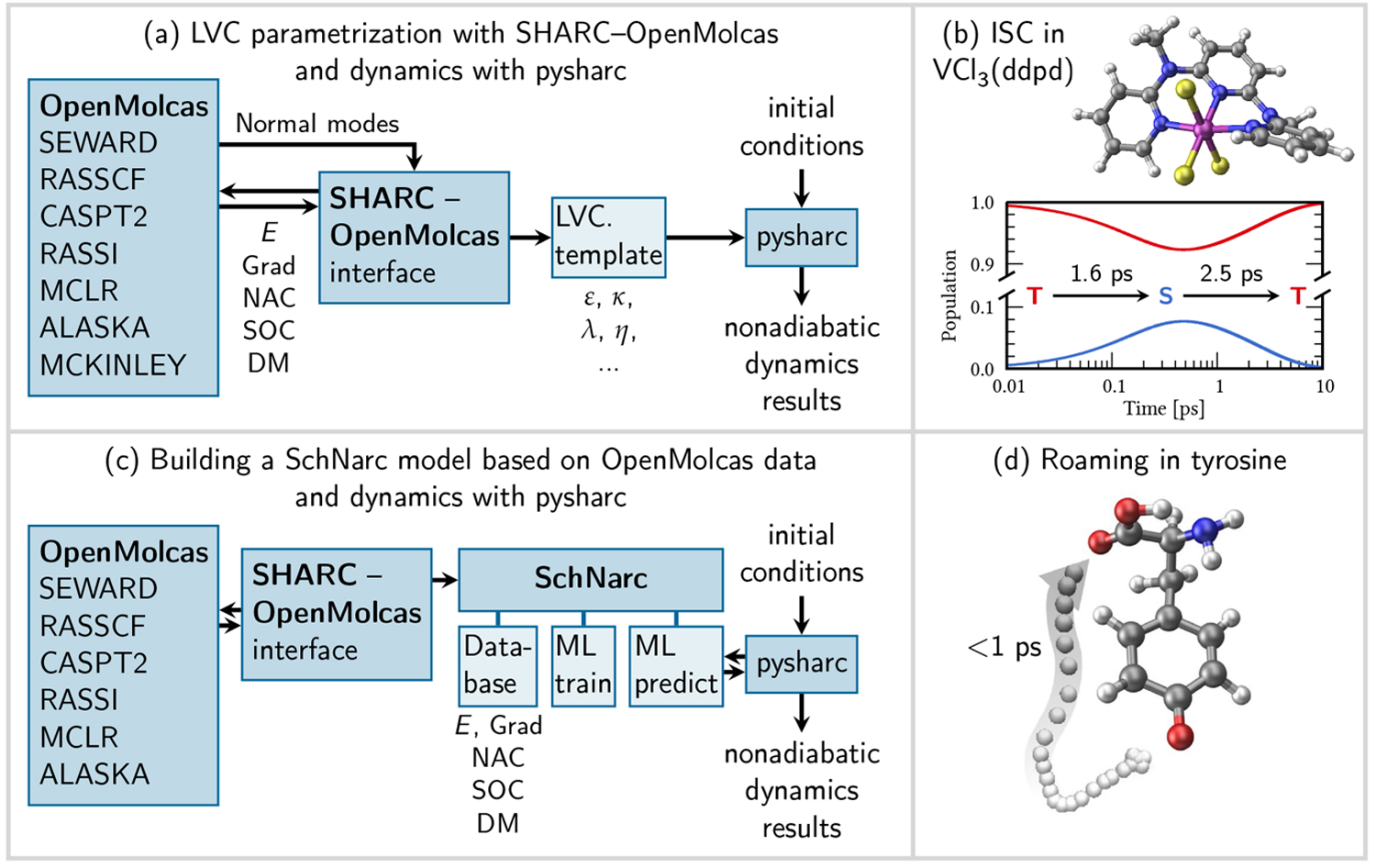
(a) Data flow for using OpenMolcas and LVC models with SHARC: A frequency calculation provides the normal modes and reference harmonic oscillator. The SHARC–OpenMolcas interface then computes energies (*E*), gradients (Grad), NAC vectors, (optionally) SOCs, and (transition) dipole moments (DM). These data are transformed into normal-mode coordinates to obtain a LVC template. Using suitable initial conditions, LVC parameters are used to simulate nonadiabatic trajectories via the pysharc driver.^172,500^ (b) Electronic population of SHARC/LVC dynamics of the VCl_3_(ddpd) complex on a picosecond time scale.^501^ (c) Data flow for using SchNarc ML models with SHARC and OpenMolcas: Desired properties (*E*, Grad, NAC, SOC, DM) are calculated with OpenMolcas and stored via the SHARC–OpenMolcas interface and the SchNarc package in a database. SchNarc uses this database to train machine learning (ML) models that predicts all required properties needed by pysharc. Together with suitable initial conditions, nonadiabatic dynamics trajectories are generated.^498^ (d) A trajectory of a roaming hydrogen atom in excited tyrosine, based on simulations on a picosecond time-scale.^502^

OpenMolcas is interfaced^9^ with the nonadiabatic molecular dynamics package SHARC (surface hopping including arbitrary couplings).^270,481^ This interface—a Python script that is part of the SHARC package—automatically generates input files for OpenMolcas and parses the output to retrieve data like energies, gradients, or couplings, as indicated in Figure 28a,c. The interface allows SHARC to perform ab initio on-the-fly dynamics simulations at a CASSCF and/or CASPT2 level of theory. Herein two new options now available in the SHARC–OpenMolcas interface and extended functionalities available in SHARC version 3.0 are reported. To alleviate the cost of expensive on-the-fly calculations, SHARC can now also work with preparameterized model potentials. First, one possibility is to use LVC models,^172,497^ as known from wavepacket dynamics simulations.^185^ The second possibility is to employ excited-state neural network potential energy surfaces and other machine learning properties via the SchNarc^498^ method—a generalization of the SchNet^499^ architecture. Below, these options and the new functionalities in SHARC 3.0 will be briefly presented. The latter includes, for example, curvature-driven and new nonadiabatic algorithms.

The LVC model uses a multidimensional harmonic oscillator for each electronic (diabatic) state, which is defined in normal mode coordinates, centered around a reference geometry (e.g., the ground state minimum). These oscillators are shifted with state-specific vertical energy shift parameters (ε) and state-specific gradient parameters (κ), and coupled by linear interaction parameters (λ), all obtained at the reference geometry. Other terms, like spin–orbit couplings (η) and (transition) dipole moments, are assumed to be constant for each diabatic state. The entire parametrization process is very efficient, requiring only one optimization plus frequency calculation followed by one excited-state single point calculation that computes all energies, gradients, nonadiabatic coupling vectors, and other terms, all available in OpenMolcas, see Figure 28a. Once the parameters are set up, SHARC can compute and diagonalize the diabatic Hamiltonian for every geometry, to obtain the adiabatic energies, gradients, and coupling elements for carrying out the nonadiabatic dynamics with the pysharc driver.^172,500^

OpenMolcas and the SHARC/LVC approach were recently exploited to investigate the nonadiabatic dynamics of the near-infrared-emissive VCl_3_(ddpd) complex (see Figure 28b).^501^ With a vanadium(III) center in a d^2^ configuration, which gives rise to a nearly triply degenerate 3_*T*_-type ground state and diverse excited states involving higher-order excitations, the use of a multiconfigurational method, such as CASSCF, is mandatory. Only by virtue of the efficient SHARC/LVC protocol and OpenMolcas was it possible to propagate up to 10 ps and discover that the initial fast (1.6 ps) triplet-to-singlet intersystem crossing is counteracted by a slower reverse intersystem crossing that takes the majority of the singlet population back into the triplet manifold.

In order to carry out machine learning potential-based dynamics simulations using the SchNarc method, one needs first to calculate energies, gradients, nonadiabatic coupling vectors and possibly (transition) dipole moments via the SHARC–OpenMolcas interface to build up a database, see Figure 28c. ML models of these properties are trained and subsequently employed in nonadiabatic dynamics simulations using the pysharc driver.^172,500^ The latter avoids slow file I/O and, in combination with the fast ML predictions, is able to push dynamics simulations up to the nanosecond time scale.^500^

One example of nonadiabatic dynamics using OpenMolcas and SchNarc is the study of excited tyrosine.^502^ Again here, multiconfigurational methods were mandatory to describe anticipated dissociative reactions. Surprisingly, by leveraging neural network models trained on CASPT2 calculations (among others), it was possible to discover unconventional roaming dynamics (Figure 28d) in excited tyrosine.^500^

SHARC version 3.0^268,270,503^ extends the SHARC interface–originally developed for TSH dynamics. Two key added capabilities are (1) curvature-driven methods,^504,505^ a type of nonadiabatic dynamics methods that do not require computation of NACs or overlap integrals of adiabatic wave functions at successive time steps (as described in section 6.2); and (2) nonadiabatic dynamics methods such as semiclassical Ehrenfest^506−509^ (SE) and coherent switching with decay of mixing^268,510^ (CSDM) that are based on self-consistent potentials (SCPs). SHARC 3.0 features several curvature-driven algorithms,^504^ including curvature-driven TSH (κTSH), curvature-driven SE (κSE), and curvature-driven CSDM (κCSDM). For the treatment of spin-conserving processes, curvature-driven algorithms only require computation of adiabatic potential energies and gradients, and for intersystem crossing they only require energies, gradients, and spin–orbit couplings. Therefore, they can be interfaced with electronic structure methods for which the wave function is not defined, for example, MC-PDFT and MS-PDFT methods. Recent applications of κCSDM to ethylene^268^ and 1,3-cyclohexadiene^511^ show the high accuracy of curvature-driven algorithms. The SCP methods in SHARC 3.0 include SE, generalized SE, CSDM, time-derivative CSDM (tCSDM),^503^ and curvature-driven CSDM (κCSDM).^504^ The CSDM, tCSDM, and κCSDM methods are recommended algorithms for nonadiabatic dynamics because of their accuracy, their robustness with respect to the choice of electronic representation, their freedom from frustrated hops (which violate self-consistency between electronic structure and nuclear motion), and the physical way in which they incorporate decoherence.^512−514^ The SHARC 3.0 package involves also the following additional features: vibrational-state-selected initial conditions; projection operators that removes artificial translational and rotational components in space-frame NACs in order to conserve center-of mass motion and total angular momentum in direct dynamics for both TSH and SCP methods;^515^ TSH with time uncertainty;^516^ TDC algorithms for self-consistent potential methods including time-derivative SE (tSE) as well as the already-mentioned tCSDM;^503^ evaluation of the TDC with norm-preserving interpolation;^517^ and a new NAC-free gradient correction scheme required for intersystem crossing processes.^518^

### 6.4 Simulations of Transient UV–vis Spectroscopy

Semiclassical and mixed quantum–classical approximations of the wave packet dynamics allow going beyond the harmonic representation of the PES and to describe events taking place on the ps time scale and large geometrical changes—such as those often found at conical intersections between the ground and excited states. A successful method to simulate wave packet dynamics is TSH,^485^ which represents the wave packet by a swarm of independent trajectories, obtained through sampling the phase space around the ground state (GS) equilibrium, subject to the Newtonian laws of motion along the PES calculated on the fly. The COBRAMM package is an interface to electronic structure codes to facilitate such simulation up to the ps time scale.^473,519,520^

In the context of a very recent global restructuring of COBRAMM, an automated workflow was implemented to perform transient electronic spectroscopy simulations within the TSH framework exploiting OpenMolcas as the QM software. The OpenMolcas/COBRAMM interface facilitates nonadiabatic dynamics simulations, in gas-phase and in an explicit environment through a QM/MM hybrid scheme, with various RASSCF/RASPT2 flavors and makes use of the recently implemented analytical gradients. The spectroscopy implementation consists of the extraction of geometries along the dynamics in user-defined intervals, the calculation of the manifold of spectroscopically relevant higher-lying states for every geometry, the generation of the individual time-slices at a given time step and, eventually, their convolution in the final spectrum. The COBRAMM package is available free of charge on GitLab.^521^

Routines for trajectory-based transient spectroscopy simulations in other spectral windows, e.g., photoelectron spectroscopy or X-ray absorption are under current development.

### 6.5 Comparison of Two Techniques for Nonadiabatic Dynamics Simulations

The ultrafast photoinduced dynamics of uridine (Urd) is used here to demonstrate the capabilities of the iSPECTRON (see section 5.2) and COBRAMM platforms, i.e., to demonstrate the difference between nonadiabatic dynamics simulations based on the DHO approximation, in combination with a second-order CGF, and on the TSH approach. Both, the DHO/CGF and TSH spectra were simulated for a water solvated Urd within a QM/MM scheme. The nucleoside conformation was taken from a classical molecular mechanics equilibration and refined at the QM(MP2)/MM level with COBRAMM. The same level of approximation was used for the GS normal modes and frequencies required by both methods. The DHO/CGF spectra were obtained at the XMS-CASPT2/SA-20-CASSCF(14,10) level at the GS equilibrium geometry, including all π-orbitals and the oxygen lone pairs in the active space. Inhomogeneous broadening can be straightforwardly accounted for by averaging the spectrum over multiple solvent arrangements. The nonadiabatic dynamics was described by a rate-equation with a ππ* → GS decay rate of 0.01 fs^–1^ corresponding to the reported 100 fs ππ* experimental lifetime. The TSH spectrum was obtained from a swarm of 50 trajectories at the XMS-CASPT2/SA-9-CASSCF(14,10) level, explicitly considering the lowest nπ* states in the dynamics. (The lowest nπ* state was found to represent a secondary channel with no intense transient features in the spectral window of interest; therefore, it is not further discussed.) The calculations of the spectroscopically relevant states were performed at the XMS-CASPT2/SA-20-CASSCF(14,10) level of theory.

**Figure 29:**
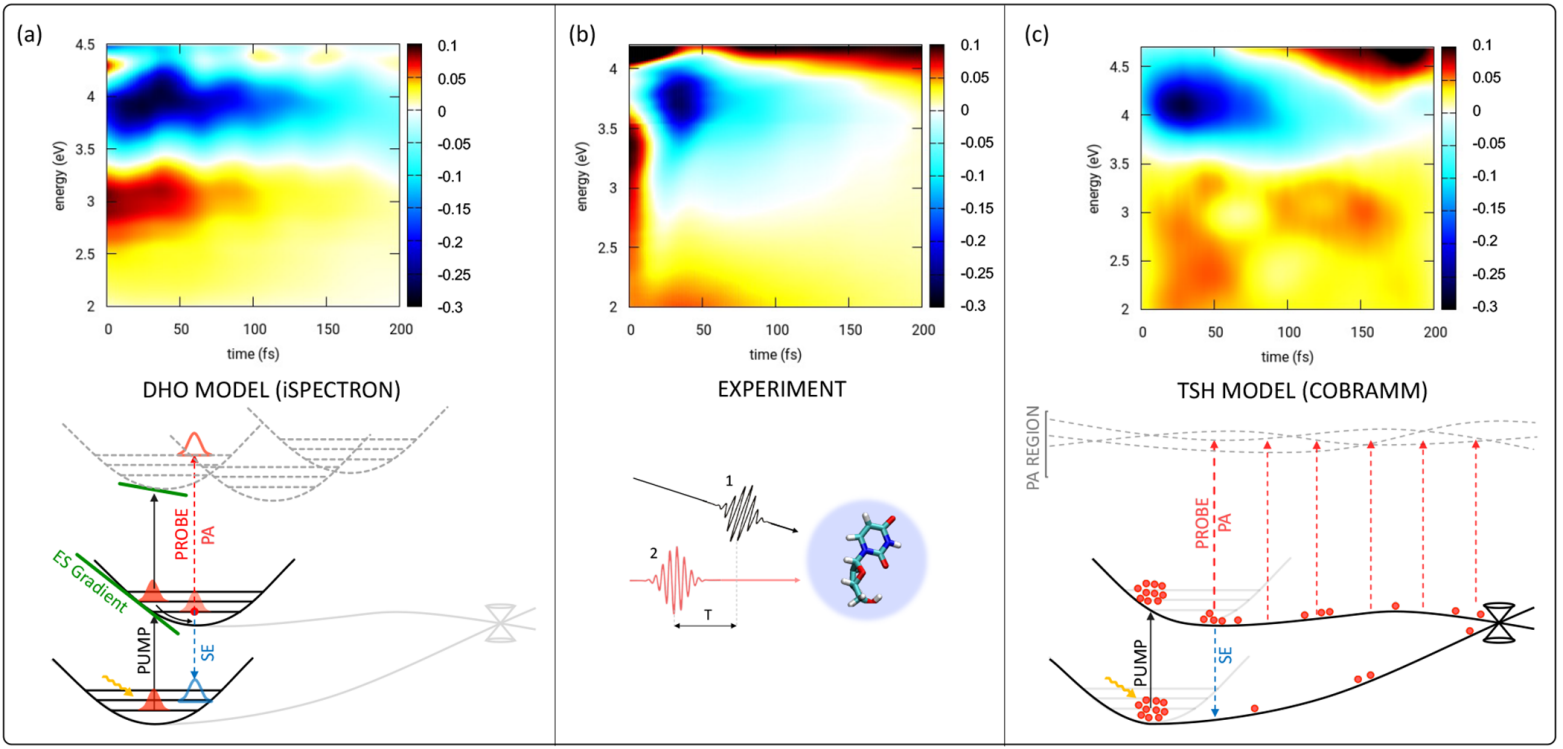
Spectra and schemes for simulated (a,c) and recorded (b) UV/vis transient absorption spectroscopy of water solvated Uridine. (a) Spectrum (top) based on the DHO model (bottom), obtained with the OpenMolcas/Spectron interface iSpectron; (b) Experimental map (top) and schematic representation of the experimental setup (bottom); (c) Spectrum (top) based on TSH model (bottom), obtained via the OpenMolcas/COBRAMM interface.

Cerullo and co-workers recorded the transient absorption spectrum of Urd in water by exciting the lowest bright band (ππ*) in the midultraviolet (UV) at 4.5 eV with a sub-30 fs pump pulse and probing in the 2 to 4.2 eV spectral window (Figure 29b).^522^ The immediate rise of an intense stimulated emission (SE) band covering the 3 to 4 eV spectral range (blue in Figure 29), as well as of a photoinduced absorption (PA) feature below 2.4 eV (PA1) can be noted. Both features decay very quickly with a 100 fs time constant, giving rise to another PA band above 4 eV (PA2). The DHO/CGF (29a) and TSH (Figure 29c) approaches reproduce qualitatively the main features of the experimental spectrum, the SE and PA1 bands which are characteristic fingerprints of the initially populated ππ* state. Both approaches reveal a further PA band at early times around 3 eV which is covered by the SE in the experiment but has been resolved for orthogonal pump and probe polarizations suppressing the SE.^522^ The TSH model captures the red-shift of the SE as well as the delayed rise of PA2 coming from the vibrationally “hot” GS formed upon decay through the ππ*/GS conical intersection. This feature is absent in the DHO/CGF spectrum as the model does not include a “hot” GS. The DHO/CGF allows to analyze quantum beating due to coherent vibrational dynamics which are strongly suppressed in the bulk.

The theoretical spectra can be subjected to global and Fourier transform analysis as their experimental counterpart, which closes the gap between experiment and simulation.

### 6.6 SI-SA-REKS Quantum–Classical Trajectories

The spin-restricted ensemble-referenced Kohn–Sham (REKS) method, its state-averaged extension (SA-REKS), and the state-interaction state-averaged REKS (SI-SA-REKS or, for short, SSR) employ ensemble-density functional theory (eDFT)^523^ to introduce nondynamic electron correlation into the description of the ground and excited electronic states of molecules and to obtain the excitation energies in a way reminiscent of multistate multiconfigurational methods of wave function-based theories.^524−526^ In fact, eDFT enables a seamless incorporation of multireference effects into the description of the molecular electronic structure, where the results of standard Kohn–Sham (KS) computations are recovered for weakly correlated (e.g., single-reference) systems. As a result, the method provides an improved description of molecules with dissociating chemical bonds and electronically excited states often featuring mixtures of biradical and charge transfer electronic characters. Because of that, the performance of SI-SA-REKS in dealing with electronically excited states has been extensively benchmarked for a model biological chromophore against different wave function-based correlated methods.^527^

The characterization of photo and thermal reactivities of even medium-size organic molecules becomes computationally expensive when multireference methods are employed in picosecond time scale simulations of the reactive dynamics.^528^ Here SSR offers a substantially more economical treatment of the electronic structure making, in principle, the systematic simulations of photochemical and photobiological processes feasible.^529,530^

The simulation of the dynamics of photochemical and photobiological processes requires an atomistic treatment of the molecular environment. This is enabled by QM/MM technologies capable to generate a model of the reacting system and to perform nonadiabatic molecular dynamics (NAMD) simulations.^531^ For this reason, some of the authors recently incorporated the SSR method in GAMESS-US(532,533) that, in turn, was interfaced with Tinker to allow the construction of QM/MM models. However, such implementation does not allow the use of the robust [Open]Molcas modules Dynamix and Surfacehop, both extensively applied to photochemical and photobiological reactions.^534,535^ On the other hand, the SSR description of the QM subsystem of QM/MM models would be particularly welcome as it makes possible to study the dynamics of this type of system faster and more accurately. Another reason for focusing on OpenMolcas is its capability, when interfaced with Tinker,^536^ to generate automatically (i.e., without human intervention) QM/MM models of photoresponsive systems.^537−539^ These are congruous QM/MM models that can give access to a rapid generation of the entire libraries of models to be used in systematic studies. Such studies would facilitate the search for novel light responsive materials such as molecular motors as well as novel photoreceptors for optogenetics. To enable SI-SA-REKS quantum–classical NAMD simulations an interface between OpenMolcas and GAMESS-US was developed (see the Supporting Information for more details on the technical details and capacity of this interface).

As an example of the flexibility of this new interface a recent application of the OpenMolcas–GAMESS-US/Tinker interface to the SSR(2,2) NAMD simulation of the E to Z photoisomerization dynamics of a specific enantiomer of (*E*)-3′-(2-methyl-2,3-dihydro-1*H*-benzo[b]cyclo-penta[d]thiophen-1-ylidene)pyrrolidin-2-one, here abbreviated as MTDP,^540^ is discussed here. The new interface played a crucial role in demonstration of the fact that the NAMD QM/MM simulations in solution yield results consistent with the experimentally observed transient absorption (TA) spectroscopy data. The MTDP could function as a single-molecule rotary motor driven by only two photochemical steps; thus avoiding the thermal helix inversion (THI) steps of classical light-driven molecular motors (i.e., the ZM and EM intermediates are not stable in such system).^541−545^ To demonstrate that a two-photon only mechanism exists, the rotary cycle was simulated at room temperature in the gas phase and in methanol, starting from the EP—*entgegen* and plus helicity—equilibrium configuration (the classic mechanism of the rotary cycle is described in the SI).

**Figure 30:**
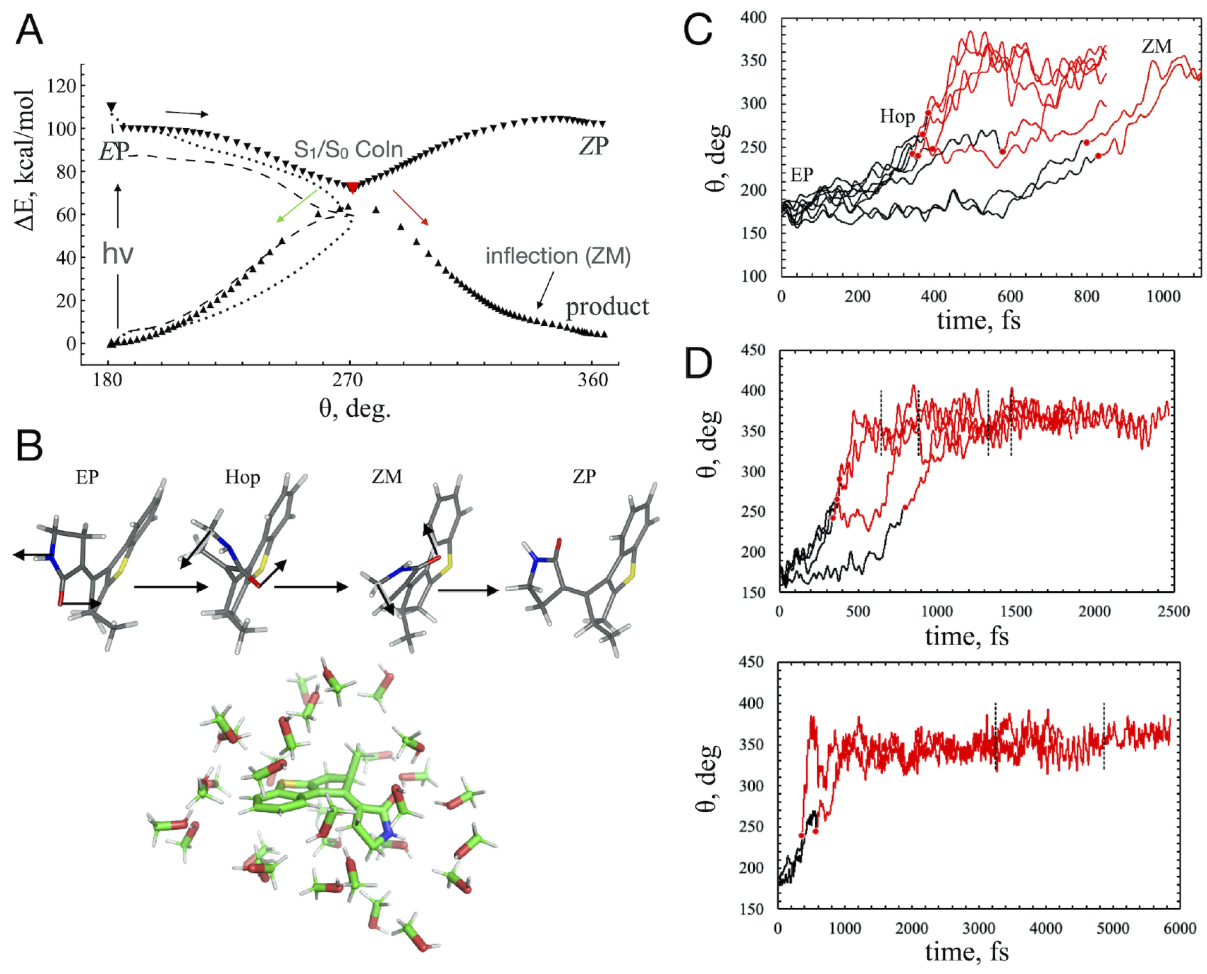
Simulations of EP → ZM → ZP half-cycle of MTDP. (A) Minimum energy path EP → ZP of the MTDP motor θ (the torsion angle defining the position of rotor with respect to stator) calculated with the SSR method (triangles). The dashed energy profiles correspond to 3-root state-average XMS-CASPT2 energies calculated with a 2 electrons in 2 π-orbitals complete active space. The dotted energy profiles show the corresponding 5-root state-average with a 10 electrons in 10 π-orbitals complete active space. (B) The top panel summarizes the conformational changes of MTDP in solvent along the QM/MM trajectories. The bottom panel shows the flexible part of the MTDP in methanol QM/MM model. (C) Simulated photoisomerization of MTDP in methanol solution based on 8 productive trajectories observed using 40 initial conditions with EP-helicity character. The propagation along the S_1_ PES (the black lines) is connected with the reactive propagation along the S_0_ PES (the red lines) by the corresponding hop points (the red circles). (D) Picosecond-scale simulation of MTDP photoisomerization in methanol solution as demonstrated through 8 reactive CCW trajectories. The top panel shows 4 trajectories in which EP to ZP transition occurs within 2.5 ps. The bottom panel demonstrated 2 trajectories in which EP to ZP transition occurs within 6 ps. The propagation along the S_1_ PES is shown with solid black lines, while the propagation along S_0_ is shown with solid red lines. The hop points are marked with red circles. The point of trajectory at which ZP conformer becomes stable is identified with vertical dashed black lines.

In this SSR(2,2) simulation of the EP → ZM → ZP half-cycle the QM subsystem (the entire MTDP solute molecule) is described at the SSR(2,2) level while the surrounding solvent is described using a parametrized MM force field. The project started with a gas-phase benchmark study comparing the SSR(2,2) energy profile of MTDP along a minimum energy path (see Figure 30A) with the corresponding XMS-CASPT2 energy profile used as a reference. As shown in Figure 30A, the system evolves toward S_1_/S_0_ CoIn (shown by the red-filled triangle), where it decays to S_0_. The torsion continues on the S_0_ PES, where the ZP configuration is reached without encountering an M helical local minimum. In fact, the ZM species occurs only as an inflection (i.e., a flatter region) on the S_0_ PES. The benchmark showed a close agreement between SSR(2,2) and XMS-CASPT2 energies along the EP → ZP path described above.

In the gas phase SSR(2,2) NAMD simulation of EP → ZM → ZP half-cycle in MTDP 50 trajectories were utilized to study the reactivity of the system. The resulting simulations predicted 280 fs latency time required for initiating the double bond torsion, a 400 fs decay time, and a 600 fs time scale of EP → ZM → ZP transition, while the quantum yield was predicted to be 87%. The photoisomerization dynamics of a methanol solution of the EP conformer of MTDP (λ_max_ = 305 nm) was experimentally investigated by femtosecond transient absorption (TA) spectroscopy upon 290 nm excitation. The measured S_1_ lifetime was elucidated to be 600 fs, which is 200 fs slower than the prediction of gas phase simulations. Moreover, the experimental quantum yield was demonstrated to be 25%, which is substantially lower, than the value predicted with gas phase simulations. Finally, through TA spectroscopy it was observed that the stable ZP diastereomer is produced after decay to S_0_ and subsequent vibrational cooling (∼30 ps) of a ZM transient species, which is substantially slower than the prediction of gas phase NAMD simulation. The above findings indicate the importance of including solvent effects.

To achieve a more accurate description of MTDP photochemistry, the newly introduced OpenMolcas–GAMESS-US/Tinker interface was used to construct the models necessary to perform QM/MM NAMD simulations of the light-driven transformation of MTDP in methanol along the EP → ZM → ZP half-cycle (Figure 30B). To do so 40 quantum-classical trajectories were used treated with the TSH algorithm. For more details regarding these simulations see the Supporting Information. The solvent simulations predicted the average S_1_ lifetime of 482 fs, while the quantum yield was predicted to be ∼20%. Here it is important to note that the simulated quantum yields and S_1_ lifetimes were obtained with relatively small statistics and are planned to be refined in the future study with larger sampling. However, the qualitative difference between the predictions of gas phase and solvent NAMD simulations (with solvent simulations being substantially closer to experimental data) is clear even with a limited statistics. In Figure 30C, the change in the torsional angle θ during EP to ZM transition in all reactive trajectories is reported. Most importantly, as shown in Figure 30D, the ZM → ZP is substantially slower in solvent simulations than in gas phase simulations.

The time scale of ZM to ZP transition places the overall EP → ZM → ZP half-cycle in the picosecond regime as opposed to the gas phase NAMD prediction that placed the entire process within less than 1 ps. Moreover, the fact that two productive trajectories did not undergo the ZM to ZP transition even within 13.5 ps is in a better agreement with ∼30 ps time scale of EP → ZM → ZP transformation seen in the experiment.

### 6.7 MC-PDFT Nonadiabatic Dynamics

Recent developments of single-state^264−266^ and multistate^267^ MC-PDFT analytic gradients have enabled the use of MC-PDFT to study electronically nonadiabatic processes. This is achieved by using the OpenMolcas interface with the nonadiabatic molecular dynamics simulation package SHARC versions 2.0^480,481,546^ and 3.0.^268,270,503^ In this section some specific details with respect to the use of the MC-PDFT approximation for nonadiabatic dynamics is presented.

Because the MC-PDFT or the MS-PDFT energy is obtained from the reference wave function without improving the reference wave function, one does not have a nonadiabatic coupling vector that corresponds to the MC-PDFT energy. Nevertheless, one can treat electronically nonadiabatic dynamics by curvature-driven methods described in section 6.3. One can also treat intersystem crossings that are not strongly affected by conical intersections, by performing single-state MC-PDFT for each spin state and approximating the nonadiabatic coupling vectors and spin–orbit couplings from the reference SA-CASSCF wave function. The latter method was applied^269^ with TSH to treat intersystem crossing dynamics between the S_1_ and T_2_ electronic states of thioformaldehyde (CH_2_S) as this transition occurs far away from conical intersections, and it has been shown that spin–orbit coupling in this system does not depend strongly on external correlation (correlation involving excitations external to a valence active space).^173^ Previous SHARC photodynamics simulations of CH_2_S using MS-CASPT2 served as excellent comparison data.^173^ The MS-CASPT2(10,6) CH_2_S simulations showed no intersystem crossing transitions within 500 fs, which is in agreement with Mai et al.’s conclusion,^173^ based on large experimental fluorescent yields, that intersystem crossing rates are small in CH_2_S. MC-PDFT(10,6) results^269^ also showed no population transitions between the two electronic states within 500 fs (see Figure 2 in ref (269)), in good agreement. Furthermore, MC-PDFT was able to simulate CH_2_S with an active space of 12 electrons in 10 orbitals—an active space that was considered too large to run MS-CASPT2 simulations^173^—and these larger-active-space calculations showed no population transfer between S_1_ and T_2_ within 500 fs.

## 7 Basis Sets, Ab Initio Model Potentials, and Orbital Rotation

Finally, three additional features are reported here. First, efficient and relatively small basis sets are important for fast multiconfigurational calculations. Hence, a new basis set for the relativistic X2C Hamiltonian, ANO-R, has been developed for the atoms from H to Rn. It has subsequently been tested in various applications. Second, embedded cluster models (a quantum part, a layer of model potentials and point charges) can be used in combination with multiconfigurational theory for electronic structure calculations of ionic solids. A simple and straightforward protocol for construction of embedding is a key function. For that purpose, a new code for the generation of ab initio model potentials (SCEPIC) has been developed. Lastly, a tool for rotating molecular orbital coefficients along with the molecular structure is provided. More details of these developments are listed below.

### 7.1 ANO-R Basis Set

Basis sets of ANO type^547^ are a special class of functions, which allow a very flexible change of the size and accuracy by selecting different contractions. This flexibility is essential for computational methods with very high scaling with respect to the basis set size. In consequence, basis sets of ANO type are the most popular among MOLCAS/OpenMolcas users in multiconfigurational calculations. The construction of the ANO basis sets also includes the option of variable density fitting based on selected electronic states of atoms and ions, which makes these basis sets “ready” for describing these electronic features.

For the last two decades, the ANO-RCC basis set^548,549^ has been widely used by the community. ANO-RCC is a relativistic basis set based on the second-order Douglas–Kroll–Hess (DKH2) Hamiltonian. The ANO-RCC basis set was developed based on a reuse of exponents of previously optimized primitive functions, and many individual decisions for each element with respect to the selection of electronic states were applied.

In contrast, the development of the new ANO-R basis set was based on the following principles: systematic construction to achieve a balanced description of all elements included as well as compactness of the basis set to ensure efficient usage in calculations. First, through systematic computation of a wide range of different sets of primitive basis functions, the minimum number of functions with a given energy difference to the complete basis set limit was selected. Then, upon selection of a common set of electron reference states and by systematic application of a natural orbital occupation number threshold, minimal sizes of density-matrix averaged^550^ ANO-contracted basis sets were built. The so-constructed ANO-R basis sets are only one possible form. Among the primitive basis functions, a set of functions and energy error was selected that yields a number of primitive functions for atoms H–Rn slightly smaller than the number of primitive functions in the ANO-RCC basis set for the same elements. However, based on our work, both smaller and larger primitive basis sets can be easily constructed. Likewise, the contracted form of the ANO-R basis sets can be adjusted for individual needs, both by choosing different occupation number thresholds and by using different electronic states in the density-averaging.^551,552^

On average, the ANO-R basis set is 10% smaller than ANO-RCC while maintaining a similar quality. However, it is important to note that the most common molecules that have to be treated with relativistic calculations, usually contain only one or two heavy elements, and all the rest is constructed from the light elements (but they also have to be described by relativistic basis set^553^). In this case, the use of ANO-R basis set provides a good performance improvement as shown in a recent benchmark study.^554^

Anions were not included into density fitting for ANO-R basis set. Thus, the description of anionic states is a hard test for the ANO-R basis set. The electron affinity (EA) of oxygen is well-known experimentally with a very high precision (1.461 112 972(87) eV^555^). Theoretical studies, with close to exact methods, predict an EA of oxygen in the range of 1.26 to 1.29 eV.^556^ Employing CASSCF calculations with an active space including 1s2s2p3s3p orbitals, followed by CASPT2, one can obtain the energies for neutral oxygen and anion. In this approach, the use of the small ANO-R-1 (3s2p1d) basis has proven to be insufficient, predicting even the sign of EA wrongly. ANO-R-2 (4s4p2d1f) estimates EA(O) as 0.82 eV, ANO-R with contraction 10s8p4d2f as 1.23 eV. The ANO-RCC basis set for the oxygen atom also contains higher angular momentum functions (g and h). Adding uncontracted g and h functions to the ANO-R basis set only slightly improves the results, reaching 1.30 eV for EA(O).

For such demanding cases, like the electron affinity of oxygen, the standard (and low-end) contraction schemes should be avoided, and a larger basis set must be used. At the same time, in many practical applications which involve oxygen atoms in a molecule, the effect of the basis set size is not that large,^554^ and reasonable results can be obtained with the ANO-R-2 basis set.

### 7.2 Ab Initio Model Potentials

Ab initio model potentials (AIMP) are a convenient way to create a layer between an ionic cluster and an electrostatic environment. Such a system can be treated by any computational techniques from DFT to multiconfigurational methods. The reader is referred to Barandiarán et al.’s^557,558^ extensive introduction to the application of AIMPs in electronic structure calculations of ionic solids. The critical limitation of this approach is the need to reconstruct the potentials for any new crystal structure.

A new code, SCEPIC, automatizes the routine of computing the AIMPs. SCEPIC is a stand-alone code, available from its home page^559^ under the Academic Free License (AFL). SCEPIC does not require any installation and it works out of the box with OpenMolcas. The input for SCEPIC contains information about crystal structure, proposed formal charges, and a set of computational details, which will be used to calculate an ion in the layer of AIMPs. The minimization procedure is used to optimize the basis set for AIMPs (both basis set-free and for potentials with a hydrogen-like basis set). The output of SCEPIC contains a ready-to-use library, which can be appended to the AIMP library in OpenMolcas.

SCEPIC also can be used to construct the input for the GATEWAY module, which contains the quantum part, AIMP layer, and a set of point charges. The latter layer is constructed algorithmically,^560^ so the total system is electroneutral and dipole and higher multiple moments are compensated.

The quality of the embedding, if AIMPs are used, can be verified by comparison of electron density and the properties computed from it, between calculations made with the same setup (functional, basis set) with periodic boundary conditions, e.g., with the CP2K code, and with OpenMolcas.^561^

The AIMP embedding has proven useful in the theoretical description of luminescent materials that are activated by local dopants. Particularly, in the case of lanthanide ion activators, the nature of recorded luminescence spectra can be very complex. If the spectra can be attributed to a single crystal site one has to deal with sets of electronic states originating from lanthanide element open shell configurations 4f^*N*^, 4f^*N*–1^5d, 4f^*N*–1^6s under the influence of a crystal matrix. AIMP embedding opens a possibility of application of multireference methods which are properly suited for solving this kind of problems. L. Seijo and Z. Barandiarán published a number of theoretical studies on local impurity states.

If there is no complete oxidation or reduction of dopant one can restrict calculations to a single active site. In this framework, spectra of CaF_2_:Pr^3+^ were studied^562^ showing that 4f^2^ A_1g_(1_*S*_0) overlaps with the 4f5d(e_g_) manifold, indicating that in this *O*_h_ site symmetry no quantum cutting occur. Moreover, the experimental 4f5d(e_g_) → 4f^2^ emission spectrum was successfully reproduced assuming simultaneous emission from two levels (^1^T_2u_ and ^1^E_u_) of the 4f5d manifold. Another feature of experimental spectra that can be studied within a single active ion cluster are impurity trapped exciton (ITE) manifolds. They appear in the calculations as electronic states where an electron occupies an orbital having significant electron density outside the first coordination shell. This electron is attracted to an impurity trapped by a hole on the 4f created in the excitation.^557,562^ Ligand-to-metal charge transfer (LMCT) processes can be studied using medium-size clusters. Barandiarán et al.^563^ studied LMCT in CaTiO_3_:Pr^3+^ and CaZrO_3_:Pr^3+^ and showed that LMCT states can be used to control balance between greenish-blue and red emission in those materials.

Cluster embedding with AIMPs is not restricted to 3D periodicity; it can also be used to model surfaces of ionic crystals and chemical reactions on surfaces. An example of such system is the diffusion of an oxygen adatom on the MgO(001) surface. This reaction has previously been proposed to be an example of a spin-crossing, where the lowest energy route would correspond to changing between singlet and triplet spin-states.^564^ Obviously, a multiconfigurational treatment is essential for the investigation of spin-crossing.

### 7.3 Rotation of Molecular Orbitals in Space

It is not uncommon to require or wish to transform the results of a particular calculation to a different reference frame. It may be needed, for example, to compare calculations done with different molecular orientations, or to compute interactions in different aggregates. Some properties like the energy are invariant to rotations and translations, and other properties like electric multipoles are trivial to transform. In the case of the molecular wave function—or the molecular orbitals (MO) in which it is based—it should also be invariant to translations and rotations; however, the MO coefficients with respect to the basis functions are not invariant to rotations. The angular components of basis functions for *l* > 0—as Cartesian or spherical harmonic functions—are always expressed in the absolute “laboratory” reference frame, such that while the MO coefficients would not change if the basis functions were rotated with the molecule, they do change when the basis functions are only translated to follow the nuclei, but not rotated. The same applies to most integrals between the basis functions: even if a property is invariant to rotations, the individual integrals from which the property can be computed could need modification. It is therefore nontrivial—but not necessarily too complicated—to transform the integrals and MO coefficients to a different molecular orientation. Explicit expressions have been given at least by Sherman and Grinter^565^ and Ivanic and Ruedenberg.^566,567^

A simple tool is provided in OpenMolcas (Tools/mort/mort.py) that simplifies these transformations, as well as changing the order of the atoms in a molecule and desymmetrizing the orbitals and integrals from a calculation done with symmetry constraints. With this, the results saved in an HDF5 file from time-consuming calculations can easily be transformed to a different frame, with minimal loss of precision, and reused for further calculation or analysis, avoiding the need for reconverging previous calculations sometimes faced by users.

## 8 Summary

The developments of the OpenMolcas program suite over the last three years have been reported throughout six thematic sections. A compact summary of each topic follows.

In the “Electronic Structure Theory” section, a number of new methods and interfaces are described, which enable novel features based on the framework of multiconfigurational SCF. In particular, improvements to the Stocastic-CI and the DMRG approaches are reported. The interfaces to Dice, RelaxSE, COLUMBUS, and GronOR are described. Moreover, three new options to the CASPT2 functionality are presented–the RMS and XDW versions of multistate CASPT2, a regularization as an alternative to level-shifting, and the frozen natural orbital option as implemented for the RASPT2 model. Finally, extensions of the MC-PDFT model are put forward. In connection with this, the LibXC infrastructure has been introduced both at the DFT and the MC-PDFT levels of approximation.

Under the theme “Electronic Spectroscopy” several extensions are reported, which facilitate the derivation of anisotropic exchange for binuclear systems beyond the Lines model, the study of model Hamiltonians for one or two spin centers with arbitrary angular momenta, the study of the hyperfine coupling at the exact two-component relativistic level, of spin-forbidden transitions, of autoionization processes using the one-center-approximation, and of Frenkel’s excitonic coupling terms. These extensions also allow the use of the semiclassical light–matter interaction operator, the simulation of photoionization processes using Dyson orbitals and of ultrafast electron dynamics.

In the “Gradients and Molecular Structure Optimization” section, the analytical nuclear gradients at the CASPT2 and MC-PDFT levels of approximation are introduced. In association with this, a molecular structure optimization algorithm based on machine-learning techniques is reported.

The following section, “Vibrational and Vibrationally Corrected Electronic Spectroscopy”, reports additional new tools in association with spectroscopy, however, here in a context beyond a fixed molecular structure. For example, the nuclear ensemble approach for vibrational corrections is available through MULTISPEC, and the displaced harmonic oscillator approximation to the nuclear wave packet approach is supported with the interface to iSpectron. Here the incorporation of tools for the study of molecular magnetism within the approximation of analytic linear vibronic coupling and a two-step approach to reduce the computational expense for vibronic coupling in the case of multiroot investigations are also described. The section is completed with a report of a new module – LEVEL – for enhanced and improved simulations of ro-vibrational spectroscopy for dinuclear systems.

The “Ab Initio Molecular Dynamics” section includes reports on tools to generate initial conditions for semiclassical molecular dynamics simulations. Additionally, improvements of nonadiabatic dynamics using the SURFACEHOP module and of the SHARC package versions 2.0 and 3.0, as interfaced to OpenMolcas, are reported. This section is concluded with the presentation of the COBRAMM interface for simulations of transient UV/vis spectroscopy, a comparison of different simulations techniques for nonadiabatic dynamics—trajectory surface hopping vs the displaced harmonic oscillator model, the newly developed OpenMolcas–GAMESS-US/Tinker interface, with an example of a nonadiabatic molecular dynamics simulation of a molecular motor, and at last some notes on the use of the MC-PDFT model in connection with simulations of nonadiabatic processes.

Finally, the “Basis Sets, Ab Initio Model Potentials and Orbital Rotation” section describes briefly new basis sets, tools to develop AIMPs and to transform molecular orbitals, consistent with the corresponding manipulations of a rigid molecular structure.

To conclude, the open-source model of software development has been very beneficial to the OpenMolcas project. The past few years have seen a surge in new developments and an expansion of the community of OpenMolcas developers. In this respect OpenMolcas and associated external programs/interfaces have developed into an open-source simulation infrastructure in the form of a *web*, where the core functionalities and file formats of OpenMolcas represents the spider in the center. Agile researchers in the electronic structure theory and molecular dynamics fields are invited to contribute to further developments of the web, either with novel implementations or improvements of existing software. If the recent rate of development is of any measure of the path forward, one should expect the project to continue to develop and the community to grow. You are all welcome to participate and enjoy.

## Supporting Information Available

Workflow and input examples for spin adaptation with GUGA-FCIQMC. Input examples for spin-purification, Stochastic-GASSCF and deterministic spin-averaged MCSCF with the NECI interface. Computational details, active spaces, discussion and input example for HCI-SCF with the Dice interface. Details and input examples for uncontracted MRCI with the RelaxSE interface. Input example for the FNO-RASPT2 method. Complete table of anisotropic magnetic exchange parameters and input examples for the Dy^III^Mn^II^ binuclear system. Input example for relativistic hyperfine coupling calculation. Input files for single- and two-photon spectra with the exact semiclassical operator. Input and output example for a LEVEL calculation. Complete computational details (including optimized geometries, extra basis functions, active space orbitals, computed energetics, and example input files), data acquisition, and its discussion for the evaluation of Frenkel’s excitonic coupling terms and the corresponding absorption spectra. Input examples and discussion for the calculation of ssDCH and Auger spectra. Input example for the RhoDyn module. Input examples for the original and two-step procedures for the analytic evaluation of vibronic coupling. Example input and output files for the SHARC–OpenMolcas interface. Descriptions of the capabilities of the OpenMolcas–GAMESS-US/Tinker interface and information about the software supporting the interface followed with a number of input examples, details of MTDP simulations in solvent. Calculated electron affinity of the oxygen atom with different ANO-R contractions. Examples of the use of SCEPIC for input generation of a bulk system and a surface (PDF, ZIP)

## Dedication

^†^ Dedicated to the fond memory of the late Prof. Josef Paldus and his invaluable contributions to GUGA.
