# Supplementary material for: The OpenMolcas Web: A Community-Driven Approach to Advancing Computational Chemistry
Source: J Chem Theory Comput. 2023 May 22;19(20):6933–91. doi: 10.1021/acs.jctc.3c00182 (PMC10601490; doi:10.1021/acs.jctc.3c00182)
Supplement: Supplementary file 1 — ct3c00182_si_001.pdf [file ct3c00182_si_001.pdf]

Supporting Information: The OpenMolcas *Web*: A Community-Driven Approach to Advancing Computational Chemistry

Giovanni Li Manni,[*,1,65] Ignacio Fdez. Galván,[*,2,65] Ali Alavi,[1,3] Flavia Aleotti,[4] Francesco Aquilante,[5] Jochen Autschbach,[6] Davide Avagliano,[4] Alberto Baiardi,[7] Jie J. Bao,[8] Stefano Battaglia,[2,9] Letitia Birnoschi,[10] Alejandro Blanco-González,[11] Sergey I. Bokarev,[12,13] Ria Broer,[14] Roberto Cacciari,[15] Paul B. Calio,[16] Rebecca K. Carlson,[8] Rafael Carvalho Couto,[17] Luis Cerdán,[18,19] Liviu F. Chibotaru,[20] Nicholas F. Chilton,[10] Jonathan Richard Church,[21] Irene Conti,[4] Sonia Coriani,[22] Juliana Cuéllar-Zuquin,[18] Razan E. Daoud,[15] Nike Dattani,[23,24] Piero Decleva,[25] Coen de Graaf,[26,27] Mickaël G. Delcey,[17] Luca De Vico,[15] Werner Dobrautz,[28] Sijia S. Dong,[8,29] Rulin Feng,[6,30] Nicolas Ferré,[31] Michael Filatov(Gulak),[32] Laura Gagliardi,[16,8] Marco Garavelli,[4] Leticia González,[33] Yafu Guan,[34] Meiyuan Guo,[35] Matthew R. Hennefarth,[16] Matthew R. Hermes,[16,8] Chad E. Hoyer,[8,36] Miquel Huix-Rotllant,[31] Vishal Kumar Jaiswal,[4] Andy Kaiser,[13] Danil S. Kaliakin,[11] Marjan Khamesian,[2] Daniel S. King,[16] Vladislav Kochetov,[37] Marek Krośnicki,[38] Arpit Arun Kumaar,[23] Ernst D. Larsson,[39] Susi Lehtola,[40,41] Marie-Bernadette Lepetit,[42,43] Hans Lischka,[44] Pablo López Ríos,[1] Marcus Lundberg,[45] Dongxia Ma,[1,8] Sebastian Mai,[33] Philipp Marquetand,[33] Isabella C. D. Merritt,[46] Francesco Montorsi,[4] Maximilian Mörchen,[7] Artur Nenov,[4] Vu Ha Anh Nguyen,[47,48] Yoshio Nishimoto,[49] Meagan S. Oakley,[8] Massimo Olivucci,[15,11] Markus Oppel,[33] Daniele Padula,[15] Riddhish Pandharkar,[16,8] Quan Manh Phung,[50,51] Felix Plasser,[52] Gerardo Raggi,[2,53] Elisa Rebolini,[54] Markus Reiher,[7] Ivan Rivalta,[4,55] Daniel Roca-Sanjuán,[18] Thies Romig,[37] Arta Anushirwan Safari,[1] Aitor Sánchez-Mansilla,[26] Andrew M. Sand,[8,56] Igor Schapiro,[21] Thais R. Scott,[8,16,57] Javier Segarra-Martí,[18] Francesco Segatta,[4] Dumitru-Claudiu Sergentu,[6,58] Prachi Sharma,[8] Ron Shepard,[59] Yinan Shu,[8] Jakob K. Staab,[10] Tjerk P. Straatsma,[60,61] Lasse Kragh Sørensen,[62] Bruno Nunes Cabral Tenorio,[22] Donald G. Truhlar,[8] Liviu Ungur,[47] Morgane Vacher,[46] Valera Veryazov,[39] Torben Arne Voß,[13]

Oskar Weser,[1] Dihua Wu,[8] Xuchun Yang,[11] David Yarkony,[63] Chen Zhou,[8] J. Patrick Zobel,[33] and Roland Lindh*,[2,64,65]

1 *Electronic Structure Theory Department, Max Planck Institute for Solid State Research, Heisenbergstraße 1, 70569 Stuttgart, Germany*
2 *Department of Chemistry – BMC, Uppsala University, P. O. Box 576, SE-75123 Uppsala, Sweden*
3 *Yusuf Hamied Department of Chemistry, University of Cambridge, Lensfield Road, Cambridge CB2 1EW, United Kingdom*
4 *Department of Industrial Chemistry "Toso Montanari", University of Bologna, 40136 Bologna, Italy*
5 *Theory and Simulation of Materials (THEOS) and National Centre for Computational Design and Discovery of Novel Materials (MARVEL), École Polytechnique Fédérale de Lausanne (EPFL), CH-1015 Lausanne, Switzerland*
6 *Department of Chemistry, University at Buffalo, State University of New York, Buffalo, NY 14260-3000, USA*
7 *ETH Zurich, Laboratory for Physical Chemistry, Vladimir-Prelog-Weg 2, 8093 Zurich, Switzerland*
8 *Department of Chemistry, Chemical Theory Center, and Minnesota Supercomputing Institute, University of Minnesota, Minneapolis, Minnesota 55455-0431, United States*
9 *Current affiliation: Department of Chemistry, University of Zurich, Winterthurerstrasse 190, Zurich 8057, Switzerland*
10 *The Department of Chemistry, The University of Manchester, M13 9PL, Manchester, UK*
11 *Chemistry Department, Bowling Green State University, Overmann Hall, Bowling Green, OH 43403, USA*
12 *Chemistry Department, School of Natural Sciences, Technical University of Munich, Lichtenbergstr. 4, 85748 Garching, Germany*
13 *Institut für Physik, Universität Rostock, Albert-Einstein-Str. 23-24, 18059 Rostock. Germany*
14 *Theoretical Chemistry, Zernike Institute for Advanced Materials, University of Groningen, Nijenborgh 4, 9747AG Groningen, The Netherlands*
15 *Dipartimento di Biotecnologie, Chimica e Farmacia, Università di Siena, Via A. Moro 2, 53100 Siena, Italy*
16 *Department of Chemistry, Pritzker School of Molecular Engineering, James Franck Institute, Chicago Center for Theoretical Chemistry, The University of Chicago, Chicago, Illinois 60637, United States*
17 *Division of Theoretical Chemistry and Biology, School of Engineering Sciences in Chemistry, Biotechnology and Health, KTH Royal Institute of Technology, SE-106 91 Stockholm, Sweden*
18 *Instituto de Ciencia Molecular, Universitat de València, Catedrático José Beltrán Martínez n.º 2, 46980 Paterna, Spain*
19 *Instituto de Óptica (IO-CSIC), Consejo Superior de Investigaciones Científicas, 28006, Madrid, Spain*
20 *Department of Chemistry, KU Leuven, Celestijnenlaan 200F, 3001 Leuven, Belgium*
21 *Institute of Chemistry, The Hebrew University of Jerusalem*
22 *Department of Chemistry, Technical University of Denmark, Kemitorvet Bldg 207, 2800 Kongens Lyngby, Denmark*

23 *HPQC Labs, Waterloo, N2T 2K9 Ontario, Canada*
24 *HPQC College, Waterloo, N2T 2K9 Ontario, Canada*
25 *Istituto Officina dei Materiali IOM-CNR and Dipartimento di Scienze Chimiche e Farmaceutiche, Università degli Studi di Trieste, I-34121 Trieste, Italy*
26 *Department of Physical and Inorganic Chemistry, Universitat Rovira i Virgili, Tarragona, Spain*
27 *ICREA, Pg. Lluís Companys 23, 08010 Barcelona, Spain*
28 *Chalmers University of Technology, Department of Chemistry and Chemical Engineering, 41296 Gothenburg, Sweden*
29 *Department of Chemistry and Chemical Biology, Department of Physics, and Department of Chemical Engineering, Northeastern University, Boston, Massachusetts 02115, United States*
30 *Department of Chemistry, Fudan University, Shanghai 200433, China*
31 *Institut de Chimie Radicalaire (UMR-7273), Aix-Marseille Univ, CNRS, ICR, 13013 Marseille, France*
32 *Department of Chemistry, Kyungpook National University, Daegu 702-701, South Korea*
33 *Institute of Theoretical Chemistry, Faculty of Chemistry, University of Vienna, Währinger Straße 17, A-1090 Vienna, Austria*
34 *State Key Laboratory of Molecular Reaction Dynamics and Center for Theoretical Computational Chemistry, Dalian Institute of Chemical Physics, Chinese Academy of Sciences, Dalian 116023, People's Republic of China*
35 *SSRL, SLAC National Accelerator Laboratory, Menlo Park, California 94025, USA*
36 *Department of Chemistry, University of Washington, Seattle, Washington 98195, United States*
37 *Institut für Physik, Universität Rostock, Albert-Einstein-Str. 23-24, 18059 Rostock, Germany*
38 *Institute of Theoretical Physics and Astrophysics, Faculty of Mathematics, Physics and Informatics, University of Gdańsk, ul Wita Stwosza 57, 80-952, Gdańsk, Poland*
39 *Division of Theoretical Chemistry, Chemical Centre, Lund University, P. O. Box 124, SE-22100, Lund, Sweden*
40 *Molecular Sciences Software Institute, Blacksburg, Virginia 24061, United States*
41 *Department of Chemistry, University of Helsinki, P.O. Box 55, FI-00014 University of Helsinki, Finland*
42 *Condensed Matter Theory Group, Institut Néel, CNRS UPR 2940, 38042 Grenoble, France*
43 *Theory Group, Institut Laue Langevin, 38042 Grenoble, France*
44 *Department of Chemistry and Biochemistry, Texas Tech University, Lubbock, TX 79409-1061, USA*
45 *Department of Chemistry – Ångström Laboratory, Uppsala University, SE-75120 Uppsala, Sweden*
46 *Nantes Université, CNRS, CEISAM, UMR 6230, F-44000 Nantes, France*
47 *Department of Chemistry, National University of Singapore, 3 Science Drive 3, 117543 Singapore*
48 *Current affiliation: School of Physics, Trinity College Dublin, The University of Dublin, College Green, Dublin 2, Ireland.*
49 *Graduate School of Science, Kyoto University, Kyoto 606-8502, Japan*

50 *Department of Chemistry, Graduate School of Science, Nagoya University, Furo-cho, Chikusa-ku, Nagoya, Aichi, 464-8602, Japan*
51 *Institute of Transformative Bio-Molecules (WPI-ITbM), Nagoya University, Furo-cho, Chikusa-ku, Nagoya, Aichi, 464-8601, Japan*
52 *Department of Chemistry, Loughborough University, Loughborough, LE11 3TU, UK*
53 *Quantum Materials and Software LTD, 128 City Road, London, EC1V 2NX, United Kingdom*
54 *Scientific Computing Group, Institut Laue Langevin, 38042 Grenoble, France*
55 *ENSL, CNRS, Laboratoire de Chimie UMR 5182, 46 allée d'Italie, 69364 Lyon, France*
56 *Department of Chemistry and Biochemistry, Butler University, Indianapolis, Indiana 46208, United States*
57 *Department of Chemistry, University of California, Irvine, California 92697, United States*
58 *Laboratory RA-03, RECENT AIR, A. I. Cuza University of Iași, RA-03 Laboratory (RECENT AIR), Iași 700506, Romania*
59 *Chemical Sciences and Engineering Division, Argonne National Laboratory, Lemont, IL 60439, USA*
60 *National Center for Computational Sciences, Oak Ridge National Laboratory, Oak Ridge, TN 37831-6373, USA*
61 *Department of Chemistry and Biochemistry, University of Alabama, Tuscaloosa, AL 35487-0336, USA*
62 *University Library, University of Southern Denmark, DK-5230 Odense M, Denmark*
63 *Department of Chemistry, Johns Hopkins University, Baltimore, Maryland 21218, USA*
64 *Uppsala Center for Computational Chemistry (UC$_3$), Uppsala University, PO Box 576, SE-751 23 Uppsala. Sweden*
65 *Contributed equally to this manuscript.*

E-mail: g.limanni@fkf.mpg.de; Ignacio.Fernandez@kemi.uu.se; roland.lindh@kemi.uu.se

Contents

S1 Stochastic Configuration Interaction Eigensolvers **S-7**
S1.1 GUGA Spin Adaptation in Stochastic-CASSCF S-7
S1.2 Spin-Purification in SD Basis S-8
S1.3 Stochastic-GASSCF S-10
S1.4 (Spin-)State-Averaged MCSCF S-12

S2 The Heat-Bath Configuration Interaction Self-Consistent Field Method **S-15**
S2.1 Computational Details S-15
S2.2 Cartesian Coordinates S-16
S2.3 Active Orbitals S-16
S2.4 Results and Discussion S-18
S2.5 HCI-SCF input example S-20

S3 Uncontracted MRCI S-20
S3.1 `RelaxSE` dependencies S-21
S3.2 Output files S-21
S3.3 Input example for CAS+DDCI calculation S-21
S3.4 Input example for SAS+S calculation S-22
S3.5 Symmetry S-23
S3.6 Restart S-23
S3.7 Parallelization S-23
S3.8 Memory S-24
S3.9 Properties calculations after a `RelaxSE` calculation S-24

S4 Frozen Natural Orbitals method applied to RASPT2 S-24

S5 Derivation of Anisotropic Exchange Interaction from Binuclear Ab Initio Calculations S-25

S6 Relativistic Hyperfine Coupling S-40

S7 Single and Two-Photon Spectra with the Exact Semi-Classical Operator S-42

S8 Vibrational–Rotational Levels for Diatomics S-47

S9 Evaluating Frenkel's Excitonic Coupling Terms S-48
S9.1 Azulene S-48
S9.1.1 Monomer geometries and energetics S-48
S9.1.2 Crystal spectrum evaluation S-56
S9.2 Z–M azobenzene derivatives heterodimer S-58
S9.2.1 Monomer geometries and energetics S-58
S9.2.2 Excitonic spectrum evaluation S-65
S9.3 Plotting absorption spectra S-66
S9.4 Example input files S-66

S10 Ionization and Autoionization Processes S-69
S10.1 ssDCH S-70
S10.2 Auger Decay Spectra S-72

S11 SA-SI-REKS Quantum–classical Trajectories S-80
S11.1 Capabilities of the Interface S-80
S11.2 A Technical Description of the Interface S-81
S11.3 Description of Rotary Cycle in Molecular Motors S-84
S11.4 Details of MTDP Simulations in Solvent S-84

S12 Two-Step Acceleration of the Analytic Evaluation of the Vibronic Couplings for Multi-Root Systems S-85

S13 MC-PDFT Nonadiabatic Dynamics S-99

S14 ANO-R Basis Set S-99
S14.1 Hartree–Fock energies for atoms with higher ANO-R-N basis sets . . . S-99

S14.2 Electron affinity of oxygen atom S-100

S15 Use of `SCEPIC` program for generation of AIMPs and for creating input templates for `OpenMolcas` calculations S-100

S16 Ab Initio Model Potentials S-101

S16.1 Quick start S-101
S16.2 Drivers for embedding script S-102
S16.3 Driver for embedding S-103
S16.4 Driver script for `SCEPIC` S-103
S16.5 Generation of input for `OpenMolcas` calculation with AIMPs S-104
S16.6 Diffusion of oxygen ad-atom on MgO(001) S-104

References S-108

S1 Stochastic Configuration Interaction Eigensolvers

S1.1 GUGA Spin Adaptation in Stochastic-CASSCF

The `OpenMolcas`–`NECI` workflow for GUGA-FCIQMC is displayed in fig. S1. Sample inputs are given in listings S1 and S2.

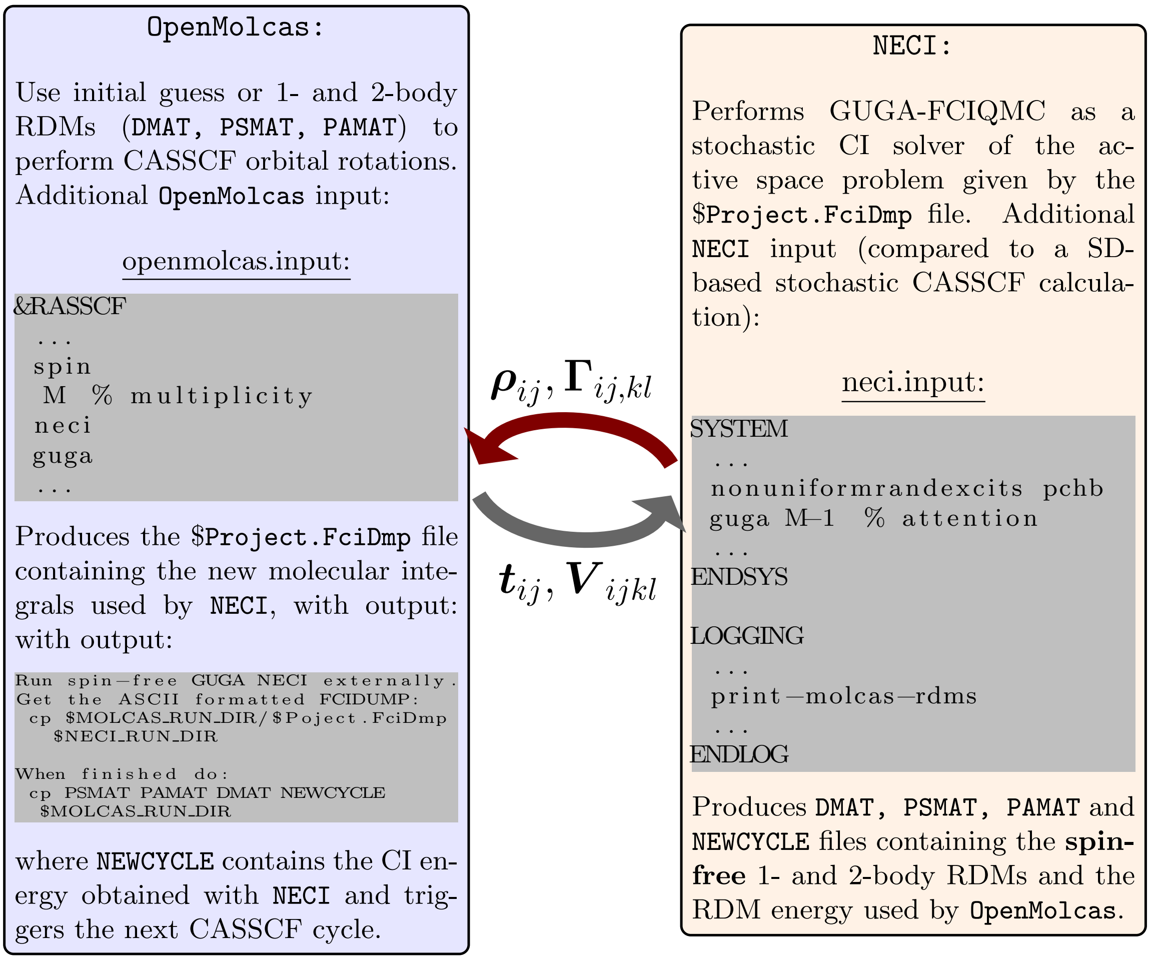

Figure S1: CASSCF/`OpenMolcas` – GUGA-FCIQMC/`NECI` Workflow.

Listing S1: Fe_2S_2 singlet CASSCF `OpenMolcas` input file. The `geometry_Fe2S2.xyz` and `fe2s2_22in26_ROHF.orbitals` files can be found in the SI of ref. S1.

```
&GATEWAY
   coord = geometry_Fe2S2.xyz
   basis = FE.ANO-RCC-VDZ, ANO-RCC-MB
   group = nosym

&SEWARD
   medium

&RASSCF
   file = $CurrDir/fe2s2_22in26_ROHF.orbitals
   Spin = 1
   nActEl = 10
```

```
    Inactive = 88
    Ras2 = 10
    guga
    NECI
        totalwalkers = 10000
        RDMlinspace = 200 3 50
        semistochastic = 1000
        definedet = 1 3 5 6 9 12 14 16 18 20
```

Listing S2: Fe_2S_2 singlet CASSCF `NECI` input file.

```
Title Iron-sulfur singlet CASSCF

System read
    electrons  10
    nonuniformrandexcits mol_guga_weighted
    nobrillouintheorem
    guga 0
endsys

calc
    methods
        method vertex fcimc
    endmethods

    definedet     1    3    5    7    9   12   14   16   18   20
    totalwalkers 100000
    semi-stochastic 5000
    pops-core 10000
    truncinitiator
    addtoinitiator  3
    allrealcoeff
    tau-values \
        start user-defined 0.01 \
        max 0.02
    tau-search \
        algorithm histogramming
    startsinglepart 100
    rdmsamplingiters 10000
endcalc

logging
    hdf5-pops
    print-molcas-rdms
    calcrdmonfly 3 5000 100
endlog
end
```

S1.2 Spin-Purification in SD Basis

Sample inputs for an open-shell singlet calculation are given in listings S3 and S4

Listing S3: Preparation of the oxygen $^1\Delta$ state in `OpenMolcas` for `NECI`.

```
&GATEWAY
     coord = O2.xyz
     basis = ANO-RCC-VDZP
```

```
    group = C1
    RICD

&SEWARD

&RASSCF
    file = $CurrDir/O2.InpOrb
    spin = 1
    /* (16, 28) is Full CI in VDZP  */
    nActEl = 16
    RAS2 = 28
    CIOnly
    NECI
        totalwalkers = 10000000
        RDMlinspace = 200000 20 1000
```

Listing S4: (16, 28) Full CI on the oxygen $^1\Delta$ state in NECI.

```
title Oxygen singlet delta state

system read
    electrons  16
    nonuniformrandexcits pchb
    nobrillouintheorem
    spin-restrict 0
    FCIDUMP-name O2.FciDmp
    freeformat

    sd-spin-purification 0.1185
endsys

calc
    time 1380

    definedet \
            1-2 3-4 \
            5-6 7-8 \
            9-10 11-12  13-14  \
            15   18

    totalwalkers 5.e7
    readpops
    walkcontgrow

    semi-stochastic 5000
    pops-core 10000

    methods
        method vertex fcimc
    endmethods

    diagshift .00
    shiftdamp .02
    stepsshift 10
    proje-changeref 1.5
    truncinitiator
    addtoinitiator  3
    allrealcoeff
```

```
    realspawncutoff .30
    jump-shift
    tau-values \
        start user-defined 0.01
    tau-search \
        algorithm conventional \
        stop-condition no-change 2000 \
        maxwalkerbloom 1
    memoryfacspawn 100.00
    memoryfacpart 50.00
    startsinglepart 10
endcalc

logging
    highlypopwrite 50
    hdf5-pops
    print-spin-resolved-RDMs
    printonerdm
    RDMlinspace 200000 20 1000
endlog
end
```

S1.3 Stochastic-GASSCF

Sample input files for a Stochastic-GASSCF calculation are provided in listings S5 and S6

Listing S5: Preparation of a GASSCF calculation on a stack of five benzene molecules with single interspace excitations in `OpenMolcas` for `NECI`.

```
&GATEWAY
    RICD
    coord = $Project.xyz
    basis = ANO-RCC-VDZP
    group = C1

&SEWARD


&RASSCF
    THRS = 1.0e-4 1.0e-1 5.0e-4
    FileOrb = $CurrDir/$Project.InpOrb
    nActEl = 30
    Symmetry = 1
    Spin = 1
    GASSCF
      5
        6
          5  7
        6
          11 13
        6
          17 19
        6
          23 25
        6
          30 30

```

```
    * Switch on NECI (external by default)
    NECI
        totalwalkers = 1000000
        RDMLinspace = 30000 20 500
        semistochastic = 20000
        definedet = 1 2 3 4 5 6    13 14 15 16 17 18    25 26 27 28 29 30    37 38
        ↪ 39 40 41 42    49 50 51 52 53 54
```

Listing S6: GASSCF on a benzene stack with single interspace excitations.

```
Title GAS on a stack of 5 benzene

System read
    electrons  30
    nobrillouintheorem
    nonuniformrandexcits GAS-CI PCHB
    GAS-spec cumulative 5
        6 5 7
        6 11 13
        6 17 19
        6 23 25
        6 30 30
        6*1 6*2 6*3 6*4 6*5
    FCIDUMP-name benzene_3.0_Ang.FciDmp
endsys

calc
    definedet 1-6   13-18   25-30   37-42    49-54

    totalwalkers 1e6

    semi-stochastic 20000
    pops-core 10000

    methods
        method vertex fcimc
    endmethods

    shiftdamp .02
    stepsshift 10

    tau-values \
        start user-defined 5e-3

    tau-search \
        algorithm conventional \
        stop-condition no-change 2000 \
        maxwalkerbloom 1

    nmcyc 10000

    proje-changeref 1.20
    truncinitiator
    addtoinitiator  3
    allrealcoeff
    realspawncutoff .30
    memoryfacspawn 10.00
    memoryfacpart 5.00
```

```
    startsinglepart 10

endcalc

logging
    highlypopwrite 50
    hdf5-pops
    print-spin-resolved-RDMs
    printonerdm
    RDMlinspace 30000 20 500
endlog
end
```

S1.4 (Spin-)State-Averaged MCSCF

A sample input for a MCSCF calculation over different spin multiplicities is given in listings S7 and S8.

Listing S7: Spin-Averaged CASSCF over doublet, quartet, sextet and octet multiplicities.

```
&GATEWAY
    coord = mn2o4.xyz
    basis = ANO-RCC-VDZP
    group = nosym
    RICD

&SEWARD

&RASSCF
    FileOrb = $Project.InpOrb
    CIROOT = 4 4 1
        * average over four roots of arbitrary multiplicity,
        * alternatively, to control the root weights w_i, use
        * CIROOT = 4 4
        *          1    2    3    4
        *          w_1  w_2  w_3  w_4
    spin = 2
        * multiplicity specified here will be ignored
    nactel = 19 0 0
    inactive = 118
    THRS = 1.0e-07, 1.0e-04, 1.0e-04
    ras2 = 16
    deleted = 0
    Symmetry = 1
    ITERation = 200 100
    prwf = 0.01
    orblisting = all
    NECI
        GUGA
        totalwalkers = 20000
        RDMlinspace  = 20000 10 100
```

Listing S8: `NECI` input for the doublet spin state.

```
# With the FCIDUMP generated within Molcas, separate NECI dynamics need to be
# run for each spin multiplicity. If one spin sector
# contains multiple states, these can be computed within
```

```
# one dynamic through the "system replica" keyword.

title Mn2O4 spin-averaging doublet
  System read
    electrons             19
    guga                  1
    nonuniformrandexcits guga-pchb
    system-replicas       2     # twice the number of doublet states
    nobrillouintheorem
    freeformat
  Endsys

  Calc
    methods
      method vertex fcimc
    endmethods
    totalWalkers    2.5e6

    definedet     1 2 3 4 5 6 7 8 9 10 11 12     13 15 17 21     24 26 28
    multiple-initial-states
         1 2 3 4 5 6 7 8 9 10 11 12     13 15 17 21 24 26 28
         1 2 3 4 5 6 7 8 9 10 11 12     13 15 17 21 24 26 28

    global-core-space
    orthogonalise-replicas
    replica-single-det-start
    semi-stochastic 10000
    pops-core       100000
    rdmsamplingiters 40000
    time  12000
    nmcyc 1000000

    tau-values \
        start user-defined 0.01
    tau-search \
        algorithm conventional \
        stop-condition no-change 2000 \
        maxwalkerbloom 1

    truncate-spawns 2.0
    diagshift 0.00
    shiftdamp 0.3
    stepsshift 10
    proje-changeref 1.2
    startsinglepart 100
    memoryfacspawn 100.0
    memoryfacpart   5.0
    truncinitiator
    addtoinitiator 3
    allrealcoeff
    realspawncutoff 0.30
  Endcalc

  Logging
    highlypopwrite           2000
    hdf5-pops
    calcrdmonfly             3 15000  100
    print-molcas-rdms
```

```
  Endlog
End
```

A sample input for a Deterministic Spin-Averaged CASSCF over one doublet and a quartet state is given in listing S9

Listing S9: Deterministic Spin-Averaged CASSCF over one doublet and a quartet state.

```

* Three separate (!) input files are required for the deterministic CASSCF over
* two multiplicities. Below three sample &RASSCF blocks are provided:

*   1. CAS-CI for the doublet:
    &RASSCF
        CIONly
        FileOrb = $Project.InpOrb
        CIROOT = 1 1 1
        spin = 2
        nactel = 19 0 0
        ras2 = 16
        inactive = 118
        WRMA
            * this keyword writes the PSMAT, PAMAT, DMAT, DSPN arrays to $WORKDIR
            * which represent the symmetric- and anti-symmetric part of the
            * 2RDM, the 1RDM and the 1 Spin-Density Matrix

*   2. CAS-CI for the quartet
    &RASSCF
        CIONly
        FileOrb = $Project.InpOrb
        CIROOT = 1 1 1
        spin = 4
        nactel = 19 0 0
        ras2 = 16
        inactive = 118
        WRMA
            * Another set of RDMs for the quartet

*   3. CASSCF which averages the RDMs
    &RASSCF
        FileOrb = $Project.InpOrb
        CIROOT = 2 2 1
            * average over two roots of arbitrary multiplicity,
        spin = 2
            * multiplicity specified here will be ignored
        nactel = 19 0 0
        inactive = 118
        ras2 = 16
        NECI
            GUGA
            totalwalkers = 20000
            RDMlinspace  = 20000 10 100
            * Specification of the entire NECI block as shown here is
            * mandatory. The program will ask you to provide the RDMs generated
            * above in consecutive numbering and a NEWCYCLE file which contains
            * the CAS-CI energies. Any renaming to PSMAT.1, PSMAT.2, ... has
            * to be manually performed by the user. Please consult the
            * GUGA-NECI interface for further guidance.
            * For each iteration of the SCF, the steps (1) and (2) have to be
```

```
            * repeated for the new orbitals written after every orbital
            * rotation to the file "IterOrb" in the WorkDir.
```

S2 The Heat-Bath Configuration Interaction Self-Consistent Field Method

Application of the HCI-SCF Method: $[(C_5H_5)Fe(C_5H_4)]_2^+$.

S2.1 Computational Details

- Two active spaces were employed, CAS(27,28) and CAS(35,36). The active natural orbitals are shown in figs. S2 and S3.
- Cartesian coordinates were obtained at the B3LYP/def2-TZVP level of theory (listing S10). All B3LYP calculations were done with the `Turbomole` package.[S2]
- The $C_{2\mathrm{h}}$ point group symmetry was used in all calculations.
- cc-pVTZ basis set for Fe, and cc-pVDZ basis set for C and H were employed.
- The Cholesky decomposition technique for the two-electron integrals was used, with a threshold of $10^{-6}\,E_\mathrm{h}$.
- DMRG-SCF calculations were done with the `OpenMolcas`–`CheMPS2` interface. Fiedler orbital ordering, perturbative noise (noise prefactor of 0.05), and residual norm threshold 10^{-4} for the Davidson algorithm were employed. Four values of m were chosen, namely 500, 1000, 2000, and 4000.
- HCI-SCF calculations were done with the `OpenMolcas`–`Dice` interface. We performed three sets of calculations with $\varepsilon_\mathrm{var} = 10^{-4}\,E_\mathrm{h}$, $5 \cdot 10^{-5}\,E_\mathrm{h}$, , $10^{-4}\,E_\mathrm{h}$ and $\varepsilon_\mathrm{per} = 10^{-5}\,E_\mathrm{h}$, $5 \cdot 10^{-6}\,E_\mathrm{h}$, $10^{-6}\,E_\mathrm{h}$, respectively. The last calculation is semi-stochastic, in which the perturbative component is calculated deterministically at $\varepsilon = 10^{-5}\,E_\mathrm{h}$ and stochastically at $\varepsilon = 10^{-6}\,E_\mathrm{h}$. Since `Dice` does not calculate the 2-RDM with semi-stochastic HCI, the active orbitals were not optimized (activated by the keyword *`CIONly`*). The active orbitals in this last calculation were taken from the HCI-SCF calculations with $\varepsilon_\mathrm{var} = 10^{-4}\,E_\mathrm{h}$ and $\varepsilon_\mathrm{per} = 10^{-5}\,E_\mathrm{h}$.
- All calculations were done with an Intel(R) Xeon(R) CPU E5-2650 @ 2.00 GHz (8 cores, 16 threads) and 62 GB of memory. `CheMPS2` was built with a shared-memory OMP parallelization, whereas `Dice` was built with MPI parallelization. The number of `OpenMolcas` processes is 4 (*`MOLCAS_NPROCS=4`*), the number of `CheMPS2` (or `Dice`) processes is 12 (*`OMP_NUM_THREADS=12`* and *`MOLCAS_DICE=12`*)
- The electronic coupling V_ET was calculated using the generalized Mulliken–Hush method:[S3]

$$V_\mathrm{ET} = \frac{(E_2 - E_1)\,|\boldsymbol{\mu}_{12}|}{\sqrt{(\boldsymbol{\mu}_{11} - \boldsymbol{\mu}_{22})^2 + 4\,|\boldsymbol{\mu}_{12}|^2}} \tag{S1}$$

with E_2 the energy of the excited state $^2\mathrm{B_u}$, E_1 the energy of the ground state $^2\mathrm{A_g}$, $\boldsymbol{\mu}_{22}$ the electric dipole moment of $^2\mathrm{B_u}$, $\boldsymbol{\mu}_{11}$ the electric dipole moment of $^2\mathrm{A_g}$, and

$\boldsymbol{\mu}_{12}$ the transition dipole moment. Since $[(C_5H_5)Fe(C_5H_4)]_2^+$ has an inversion center, eq. (S1) becomes:

$$V_{\mathrm{ET}} = \frac{E_2 - E_1}{2} \tag{S2}$$

S2.2 Cartesian Coordinates

Listing S10: B3LYP/def2-TZVP structure (in Å) of $[(C_5H_5)Fe(C_5H_4)]_2^+$.

```
40

C   1.9982613   2.1397439   0.7101555
C   1.0606557   1.1702929   1.1519365
C   0.5036756   0.5158557   0.0000000
C   1.0606557   1.1702929  -1.1519365
C   1.9982613   2.1397439  -0.7101555
H   2.5875873   2.7816599   1.3448739
H   0.8282172   0.9447440   2.1799754
H   0.8282172   0.9447440  -2.1799754
H   2.5875873   2.7816599  -1.3448739
Fe  0.0992538   2.6229777   0.0000000
C  -1.9749164   3.0878516   0.0000000
C  -1.3541382   3.6402869  -1.1501836
C  -0.3477934   4.5468736  -0.7107298
C  -0.3477934   4.5468736   0.7107298
C  -1.3541382   3.6402869   1.1501836
H  -2.7591408   2.3470637   0.0000000
H  -1.5980451   3.4116585  -2.1751106
H   0.3040348   5.1258255  -1.3449579
H   0.3040348   5.1258255   1.3449579
H  -1.5980451   3.4116585   2.1751106
H   1.5980451  -3.4116585  -2.1751106
H  -0.8282172  -0.9447440  -2.1799754
H  -2.5875873  -2.7816599  -1.3448739
C   1.3541382  -3.6402869  -1.1501836
C  -1.0606557  -1.1702929  -1.1519365
C  -1.9982613  -2.1397439  -0.7101555
H   2.7591408  -2.3470637   0.0000000
C   1.9749164  -3.0878516   0.0000000
C   0.3477934  -4.5468736  -0.7107298
H  -0.3040348  -5.1258255  -1.3449579
Fe -0.0992538  -2.6229777   0.0000000
C  -0.5036756  -0.5158557   0.0000000
C  -1.9982613  -2.1397439   0.7101555
C   1.3541382  -3.6402869   1.1501836
C   0.3477934  -4.5468736   0.7107298
H  -2.5875873  -2.7816599   1.3448739
C  -1.0606557  -1.1702929   1.1519365
H   1.5980451  -3.4116585   2.1751106
H  -0.3040348  -5.1258255   1.3449579
H  -0.8282172  -0.9447440   2.1799754
```

S2.3 Active Orbitals

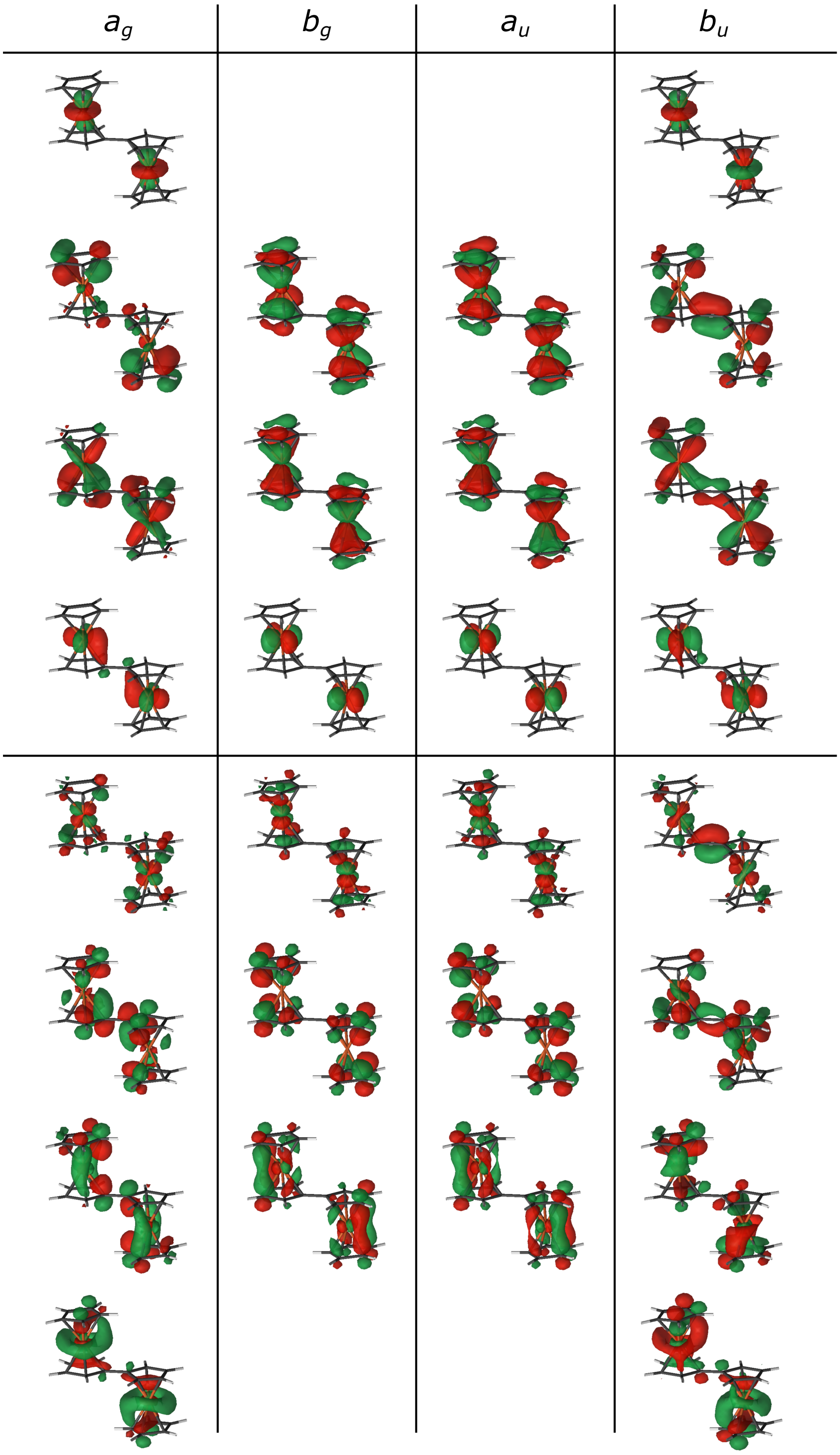

Figure S2: CAS(27,28) active natural orbitals of $[(C_5H_5)Fe(C_5H_4)]_2^+$.

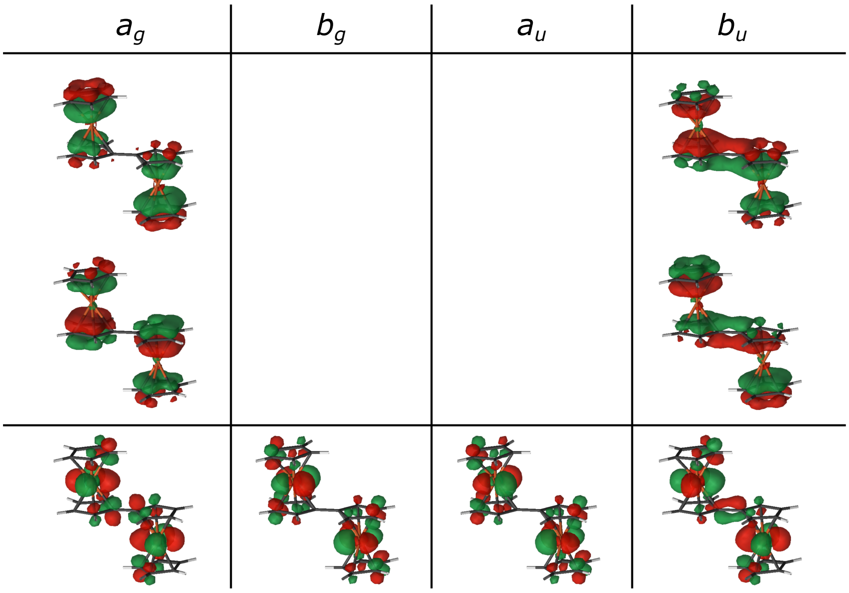

Figure S3: Extra active orbitals in the CAS(35,36) calculations of $[(C_5H_5)Fe(C_5H_4)]_2^+$.

S2.4 Results and Discussion

In tables S1 and S2, we present the total energies (in E_h) of the ground state $^2\mathrm{A_g}$ and the excited state $^2\mathrm{B_u}$ of $[(C_5H_5)Fe(C_5H_4)]_2^+$, calculated with DMRG-SCF and HCI-SCF. It can be seen that the total energies are far from converged in all cases. In contrast, the electronic coupling V_ET converges relatively fast with respect to the number of renormalized states m and the thresholds ε. For the smaller active space CAS(27,28), HCI-SCF, DMRG-SCF[$m = 4000$], and semi-stochastic HCI predict a V_ET value of $\sim$4.4 kcal mol^{-1}. For the larger active space CAS(35,36), even at an m-value of 6000, the electronic coupling has not yet converged. HCI-SCF and semi-stochastic HCI, on the other hand, give very similar results (4.4 kcal mol^{-1}). Since there is still a gap of 1.2 kcal mol^{-1} between the best (S)HCI and DMRG results, the converged relative energy is expected to be somewhere between 4.5 kcal mol^{-1} and 5.7 kcal mol^{-1}.

Table S1: Total energies (in E_h) of the ground state $^2\mathrm{A_g}$ and the excited state $^2\mathrm{B_u}$ of $[(\mathrm{C_5H_5})\mathrm{Fe}(\mathrm{C_5H_4})]_2^+$, calculated with DMRG-SCF(27,28) and HCI-SCF(27,28).

	ε_var	ε_per	$E(^2\mathrm{A_g})$	$E(^2\mathrm{B_u})$	V_ET [a]
	(E_h)		(E_h, −3292)		(kcal mol^{-1})
HCI-SCF	10^{-4}	10^{-5}	−0.799 611	−0.784 802	4.6
HCI-SCF	$5 \cdot 10^{-5}$	$5 \cdot 10^{-6}$	−0.802 740	−0.789 133	4.3
HCI[b]	10^{-4}	10^{-6}	−0.808 930	−0.794 811	4.4
DMRG-SCF[$m = 500$]			−0.798 354	−0.781 977	5.1
DMRG-SCF[$m = 1000$]			−0.803 205	−0.792 376	3.4
DMRG-SCF[$m = 2000$]			−0.807 224	−0.795 179	3.8
DMRG-SCF[$m = 4000$]			−0.810 455	−0.796 727	4.3

[a] The electronic coupling V_ET is half of the energy gap between $^2\mathrm{A_g}$ and $^2\mathrm{B_u}$.
[b] Semi-stochastic HCI calculation, the perturbative component is calculated deterministically at $\varepsilon = 10^{-5}\,E_\mathrm{h}$ and stochastically at $\varepsilon = 10^{-6}\,E_\mathrm{h}$. The active space is taken from the cheapest HCI-SCF calculation.

Table S2: Total energies (in E_h) of the ground state $^2\mathrm{A_g}$ and the excited state $^2\mathrm{B_u}$ of $[(\mathrm{C_5H_5})\mathrm{Fe}(\mathrm{C_5H_4})]_2^+$, calculated with DMRG-SCF(35,36) and HCI-SCF(35,36).

	ε_var	ε_per	$E(^2\mathrm{A_g})$	$E(^2\mathrm{B_u})$	V_ET [a]
	(E_h)		(E_h, −3292)		(kcal mol^{-1})
HCI-SCF[b]	10^{-4}	10^{-5}	−0.888 687	−0.874 637	4.4
HCI[c]	10^{-4}	10^{-6}	−0.908 208	−0.893 885	4.5
DMRG-SCF[$m = 500$]			−0.884 200	−0.870 386	4.3
DMRG-SCF[$m = 1000$]			−0.906 087	−0.879 654	8.3
DMRG-SCF[$m = 2000$]			−0.912 357	−0.890 646	6.8
DMRG-CI[$m = 4000$][d]			−0.919 855	−0.900 708	6.0
DMRG-CI[$m = 6000$][d]			−0.922 990	−0.904 974	5.7

[a] The electronic coupling V_ET is half of the energy gap between $^2\mathrm{A_g}$ and $^2\mathrm{B_u}$.
[b] HCI-SCF calculations with $\varepsilon_\mathrm{var} = 5 \cdot 10^{-6}\,E_\mathrm{h}$ require more than 62 GB of memory.
[c] Semi-stochastic HCI calculation, the perturbative component is calculated deterministically at $\varepsilon = 10^{-5}\,E_\mathrm{h}$ and stochastically at $\varepsilon = 10^{-6}\,E_\mathrm{h}$. The active space is taken from the cheapest HCI-SCF calculations.
[d] DMRG-CI calculations only, the active space is taken from the DMRG-SCF[$m = 2000$] calculations.

S2.5 HCI-SCF input example

In listing S11, we present an input example to perform an HCI-CAS calculation. This input is to calculate the ^2A_g state of $[(C_5H_5)Fe(C_5H_4)]_2^+$. The keyword ***DICE*** must be used to activate the `OpenMolcas–Dice` interface. *DIOCcupations* is used to specify a starting (or reference) determinant. The keyword requires first the number of determinants, followed by determinants. Each determinant is inserted as a string of aliases of occupations/couplings of the active (RAS2) orbitals with the aliases *2* = full, ***u*** = up, ***d*** = down, *0* = empty. Dice can run in parallel with the number of Dice processes set by ***MOLCAS_DICE***.

Listing S11: HCI-CAS(27,28) calculation of the ^2A_g state of $[(C_5H_5)Fe(C_5H_4)]_2^+$.

```
>> export MOLCAS_DICE=12

&RASSCF
 SPIN     = 1
 SYMMetry = 2
 NACTel   = 27 0 0
 INACtive = 14  8  8  13
 RAS2     =  8  6  6   8
 THRS     = 1.0e-06 1.0e-03 1.0e-03
 LUMOrb
 DICE
 EPSIlon  = 1.0e-4 1.0e-5
 DIOCcupations
  1
 2 2 2 u 0 0 0 0  2 2 2 0 0 0  2 2 2 0 0 0  2 2 2 2 0 0 0 0
```

In listing S12, we show the same calculation but with semi-stochastic HCI.

Listing S12: Semi-stochastic HCI(27,28) calculation of the ^2A_g state of $[(C_5H_5)Fe(C_5H_4)]_2^+$.

```
>> export MOLCAS_DICE=12

&RASSCF
 SPIN     = 1
 SYMMetry = 2
 NACTel   = 27 0 0
 INACtive = 14  8  8  13
 RAS2     =  8  6  6   8
 THRS     = 1.0e-06 1.0e-03 1.0e-03
 LUMOrb
 DICE
 EPSilon  = 1.0e-4 1.0e-6
 DIOCcupations
  1
 2 2 2 u 0 0 0 0  2 2 2 0 0 0  2 2 2 0 0 0  2 2 2 2 0 0 0 0
 STOChastic
 CIONly
```

S3 Uncontracted MRCI

Use of the `RelaxSE` code for MRCI calculations.

This section describes typical inputs for CAS+DDCI and SAS+S types of calculations. It also details extra information on parallelization, symmetry, restart, and the property associated code.

S3.1 `RelaxSE` dependencies

The `RelaxSE` code requires

- the `TraOne` and `TraInt` files issued from the `MOTRA` module in `OpenMolcas`, computed in the conventional way,
- an `INPUT` file providing the input namelists,
- a `prefix.ref0` input file giving the configurations selected within the CAS when required.

S3.2 Output files

The `RelaxSE` code provides the following files

- the output file as `prefix.out`,
- the wave functions at the last Davidson iteration (for restart or properties calculations) in `prefix.restart`,

and if required by the presence of ***propinp*** namelist in the `INPUT` file

- the `prefix.bdet` file containing the list of determinants,
- the `prefix.sass` file containing extra information.

S3.3 Input example for CAS+DDCI calculation

This calculation has been published in ref. S4.

Listing S13: `INPUT` file.

```
&sassinp
  prefix="clusters1",
  method = "SAS+S"
  restart = .false.
&end

&infomolcasinp
  nirrep=1,
  ntot=235,
  iIrTb(1,1) = 1,
  iChTb(1,1) = 1
&end

&orbinp
  norb_gel  = 2,
  norb_occ  = 52,
  norb_ligo = 4,
  norb_act  = 4,
  norb_ligv = 0,
```

```
    norb_virt = 175,
    norb_del  = 11,
  &end

  &vecinp
   stot=3,
   sz=2,
   vec_irrep=1,
   nvec=2,
   nref0=1,
   nelact = 4
  &end

  &davidinp
   SizeheffDavidson = 10,
   tol_conv = 1.d-8,
   ITER0 = 1
  &end

  &propinp
   Nprop=1
  &end
```

Listing S14: `clusters1.sass.ref0` file.

```
59u 60u 61u 62d
```

S3.4 Input example for SAS+S calculation

This calculation has been published in ref. S5.

Listing S15: `INPUT` file.

```
  &sassinp
    prefix="Joo2",
    method = "SAS+S"
    restart = .false.
  &end

  &infomolcasinp
    nirrep=1,
    ntot=248,
    iIrTb(1,1) = 1,
    iChTb(1,1) = 1
  &end


  &orbinp
    norb_gel  = 0,
    norb_occ  = 50,
    norb_ligo = 6,
    norb_act  = 6,
    norb_ligv = 0,
    norb_virt = 186,
    norb_del  = 0,
  &end

```

```
&vecinp
 stot=5,
 sz=4,
 vec_irrep=1,
 nvec=2,
 nref0=1,
 nelact = 6
&end

&davidinp
SizeheffDavidson = 10,
tol_conv = 1.d-8,
ITER0 = 1
&end

&propinp
 Nprop=1
&end
```

Listing S16: `Joo2.sass.ref0` file.

```
57u 58u 59u 60u 61u 62d
```

S3.5 Symmetry

In the present version of the the `RelaxSE` code, the symmetry is not implemented. As a consequence the symmetry information in the *`infomolcasinp`* namelist should be left as in the previous examples.

S3.6 Restart

If the calculation is not finished while stopped for any reason, of if one desire more accuracy, a restart from the last Davidson's iteration can be done. The prefix.restart file will then be required in addition to the `INPUT` and `prefix.ref0` files. To specify to the code to restart from the prefix.restart file one should add the following keywords:

- in the *`sassinp`* namelist: *`restart=.true.`*,
- in the *`davidinp`* namelist: *`iter0=number_of_the_last_iteration`* in the previous run, the *`tol_conv`* keyword can also be specified to increase the accuracy of the convergence for the Davidson's diagonalization procedure.

S3.7 Parallelization

The `RelaxSE` code is implemented in a MPI+OpenMP scheme. The number of MPI processes should remain small (4–6 is an optimum) while the number of OpenMP threads per MPI processes has been used up to 40 with a good scaling (see ref. S6) for more precision. It is also recommended to adjust the optional keyword *`SIZEBATCH`* (default 40) to a multiple of the number of OpenMP threads, as close as possible to a divisor of the number of virtual+ligv orbitals.

S3.8 Memory

The `RelaxSE` use of memory is often a problem for large calculations. For up to $250 \cdot 10^6$ determinants, it should work on a computer with 200 GB of memory per node. When reaching 10^9 determinants one should have more than 1 TB of memory per node available.

A low memory version of the code is under progress.

S3.9 Properties calculations after a `RelaxSE` calculation

A property code is provided in the `RelaxSE` realise. To be used it requires the same `INPUT` as `RelaxSE`, the `prefix.restart`, `prefix.bdet` and `prefix.sass` file. The last ones are generated by `RelaxSE` only is the ***propinp*** namelist is present in the `INPUT` file. If forgotten in the first run a restart calculation can be done to generate them.

Presently only few properties are coded,

- the projection of the computed states wave functions into the CAS space
- the calculation of the one-electron density matrices,
- the printing of the computed states wave functions in human readable format (to be used with care).

Other properties implementations are in progress.

S4 Frozen Natural Orbitals method applied to RASPT2

An input example for one of the FNO-RASPT2 calculations reported on the PSB-11 system ($\zeta = 99\,\%$, $\sigma = 0.1\,E_\mathrm{h}$) is given in listing S17

Listing S17: `OpenMolcas` input for a FNO-RASPT2 calculation.

```
&gateway
    Coord=$HomeDir/coord.xyz
    Basis=ANO-L-VDZP
    Group=nosymm
    RICD

 &seward

>>>COPY $HomeDir/cas.RasOrb INPORB

  &rasscf
    spin
      1
    symm
      1
    nactel
      12 3 3
    ras1
      6
    ras2
      0
    ras3
```

```
    6
  inactive
    76
  ciroot
    3 3 1
  lumorb


&caspt2
  imag=0.2
  ipea=0.0
  maxiter=200
  multistate=3 1 2 3
  nomult
  FNOCaspt2=-0.01
  RegFNOparameter=0.1
```

S5 Derivation of Anisotropic Exchange Interaction from Binuclear Ab Initio Calculations

Table S3 collects all parameters of the anisotropic magnetic exchange extracted from calculation of the binuclear system.

Table S3: Parameters of the anisotropic magnetic exchange (in cm^{-1}) extracted from calculation of the DyMn binuclear system. The active space of the CASSCF method included $4f^9$ and $5d^5$ shells of the Dy^{III} and Mn^{II}, respectively, amounting to 14 electrons in 12 orbitals. All roots arising from the coupling of the ground ^6H term of Dy^{III} and ground spin $S_B = 5/2$ of Mn^{2+} were explicitly optimized and mixed by spin–orbit interaction in RASSI. The ANO-RCC-VTZP basis set was used for closer atoms, while smaller VDZP contractions were used for distant atoms. This is the full version of table 1

k_A	q_A	k_B	q_B	Real Part	Imaginary Part
1	−1	1	−1	$-7.3853\cdot10^{-5}$	$9.0536\cdot10^{-5}$
1	−1	1	0	$-3.4434\cdot10^{-5}$	$2.7705\cdot10^{-6}$
1	−1	1	1	$-4.7023\cdot10^{-4}$	$1.9874\cdot10^{-4}$
1	−1	3	−3	$2.2467\cdot10^{-8}$	$-1.3965\cdot10^{-8}$
1	−1	3	−2	$9.2186\cdot10^{-8}$	$1.8182\cdot10^{-7}$
1	−1	3	−1	$-3.7601\cdot10^{-7}$	$2.8688\cdot10^{-7}$
1	−1	3	0	$-7.3376\cdot10^{-7}$	$-1.0045\cdot10^{-6}$
1	−1	3	1	$2.2679\cdot10^{-6}$	$1.9140\cdot10^{-6}$
1	−1	3	2	$-4.2336\cdot10^{-7}$	$-8.4868\cdot10^{-7}$
1	−1	3	3	$1.9361\cdot10^{-5}$	$-4.4965\cdot10^{-5}$
1	−1	5	−5	$-3.4628\cdot10^{-11}$	$4.2841\cdot10^{-11}$
1	−1	5	−4	$-1.4420\cdot10^{-10}$	$9.1725\cdot10^{-11}$
1	−1	5	−3	$2.4222\cdot10^{-9}$	$-6.7578\cdot10^{-10}$
1	−1	5	−2	$3.2058\cdot10^{-9}$	$-1.9943\cdot10^{-9}$
1	−1	5	−1	$2.1029\cdot10^{-10}$	$1.1379\cdot10^{-8}$
1	−1	5	0	$-1.6505\cdot10^{-8}$	$8.9115\cdot10^{-9}$
1	−1	5	1	$-2.5302\cdot10^{-8}$	$-8.2347\cdot10^{-8}$

1	−1	5	2	$1.7726{\cdot}10^{-7}$	$-5.9765{\cdot}10^{-9}$
1	−1	5	3	$2.2294{\cdot}10^{-7}$	$1.0088{\cdot}10^{-6}$
1	−1	5	4	$-1.0777{\cdot}10^{-6}$	$-3.3309{\cdot}10^{-7}$
1	−1	5	5	$-2.2523{\cdot}10^{-6}$	$-4.2296{\cdot}10^{-6}$
1	0	1	−1	$2.0764{\cdot}10^{-2}$	$-1.2782{\cdot}10^{-3}$
1	0	1	0	-1.6573	$2.6958{\cdot}10^{-18}$
1	0	1	1	$-2.0764{\cdot}10^{-2}$	$-1.2782{\cdot}10^{-3}$
1	0	3	−3	$7.1690{\cdot}10^{-5}$	$-7.3159{\cdot}10^{-5}$
1	0	3	−2	$7.0613{\cdot}10^{-4}$	$1.7459{\cdot}10^{-5}$
1	0	3	−1	$7.2281{\cdot}10^{-5}$	$3.2494{\cdot}10^{-4}$
1	0	3	0	$-5.8643{\cdot}10^{-3}$	$-4.3972{\cdot}10^{-19}$
1	0	3	1	$-7.2281{\cdot}10^{-5}$	$3.2494{\cdot}10^{-4}$
1	0	3	2	$7.0613{\cdot}10^{-4}$	$-1.7459{\cdot}10^{-5}$
1	0	3	3	$-7.1690{\cdot}10^{-5}$	$-7.3159{\cdot}10^{-5}$
1	0	5	−5	$9.0205{\cdot}10^{-8}$	$-5.9258{\cdot}10^{-9}$
1	0	5	−4	$8.1000{\cdot}10^{-7}$	$5.3169{\cdot}10^{-7}$
1	0	5	−3	$-1.1102{\cdot}10^{-6}$	$7.4078{\cdot}10^{-7}$
1	0	5	−2	$-7.3518{\cdot}10^{-6}$	$-5.8876{\cdot}10^{-7}$
1	0	5	−1	$-3.2085{\cdot}10^{-6}$	$-7.3596{\cdot}10^{-6}$
1	0	5	0	$1.5686{\cdot}10^{-5}$	$-4.7561{\cdot}10^{-21}$
1	0	5	1	$3.2085{\cdot}10^{-6}$	$-7.3596{\cdot}10^{-6}$
1	0	5	2	$-7.3518{\cdot}10^{-6}$	$5.8876{\cdot}10^{-7}$
1	0	5	3	$1.1102{\cdot}10^{-6}$	$7.4078{\cdot}10^{-7}$
1	0	5	4	$8.1000{\cdot}10^{-7}$	$-5.3169{\cdot}10^{-7}$
1	0	5	5	$-9.0205{\cdot}10^{-8}$	$-5.9258{\cdot}10^{-9}$
1	1	1	−1	$-4.7023{\cdot}10^{-4}$	$-1.9874{\cdot}10^{-4}$
1	1	1	0	$3.4434{\cdot}10^{-5}$	$2.7705{\cdot}10^{-6}$
1	1	1	1	$-7.3853{\cdot}10^{-5}$	$-9.0536{\cdot}10^{-5}$
1	1	3	−3	$1.9361{\cdot}10^{-5}$	$4.4965{\cdot}10^{-5}$
1	1	3	−2	$4.2336{\cdot}10^{-7}$	$-8.4868{\cdot}10^{-7}$
1	1	3	−1	$2.2679{\cdot}10^{-6}$	$-1.9140{\cdot}10^{-6}$
1	1	3	0	$7.3376{\cdot}10^{-7}$	$-1.0045{\cdot}10^{-6}$
1	1	3	1	$-3.7601{\cdot}10^{-7}$	$-2.8688{\cdot}10^{-7}$
1	1	3	2	$-9.2186{\cdot}10^{-8}$	$1.8182{\cdot}10^{-7}$
1	1	3	3	$2.2467{\cdot}10^{-8}$	$1.3965{\cdot}10^{-8}$
1	1	5	−5	$-2.2523{\cdot}10^{-6}$	$4.2296{\cdot}10^{-6}$
1	1	5	−4	$1.0777{\cdot}10^{-6}$	$-3.3309{\cdot}10^{-7}$
1	1	5	−3	$2.2294{\cdot}10^{-7}$	$-1.0088{\cdot}10^{-6}$
1	1	5	−2	$-1.7726{\cdot}10^{-7}$	$-5.9765{\cdot}10^{-9}$
1	1	5	−1	$-2.5302{\cdot}10^{-8}$	$8.2347{\cdot}10^{-8}$
1	1	5	0	$1.6505{\cdot}10^{-8}$	$8.9115{\cdot}10^{-9}$
1	1	5	1	$2.1029{\cdot}10^{-10}$	$-1.1379{\cdot}10^{-8}$
1	1	5	2	$-3.2058{\cdot}10^{-9}$	$-1.9943{\cdot}10^{-9}$
1	1	5	3	$2.4222{\cdot}10^{-9}$	$6.7578{\cdot}10^{-10}$
1	1	5	4	$1.4420{\cdot}10^{-10}$	$9.1725{\cdot}10^{-11}$
1	1	5	5	$-3.4628{\cdot}10^{-11}$	$-4.2841{\cdot}10^{-11}$

Input examples for the derivation of anisotropic exchange interaction are

given in listings S18 and S19. From the calculation with listing S18, the file `DyMn_vtzp_vdzp_cas_14in12_396r_aniso_cas.aniso` is obtained, which is used in listing S19 as *DATA*. The file `Dy_in_DyMn_vtzp_vdzp_cas_9in7_aniso_cas.old.aniso` is the data file for the single Dy site, where the Mn ion was computationally replaced by diamagnetic Zn, closed shell. The file `Mn_in_DyMn_vtzp_vdzp_cas_5in5_aniso_cas.old.aniso` is the data file for the single Mn site, where the Dy ion was computationally replaced by diamagnetic Lu, closed shell. The datafile for Mn can be in principle avoided in this particular case, but here it shown as an example for a general case.

Listing S18: Input file for the complete CASSCF/RASSI/SINGLE_ANISO calculation for the entire binuclear DyMn compound.

```
&SEWARD
Title
DyMn full calculation
ANGM
2.21452658174807357384 15.28782765643874124177 15.91146562345711475748
AMFI

DOAN
RICD
SDIP

CDTH
1.0d-6

VERBOSE

Basis Set
DY.ANO-RCC-vtzp
DY1 1.17187700 8.08997000 8.41998500 Angstrom
End of Basis Set

Basis Set
MN.ANO-RCC-vdzp
MN2 1.50694300 4.68651000 6.93724100 Angstrom
End of Basis Set

Basis Set
O.ANO-RCC-vdzp
O3 1.00627200 12.53340600 9.80793300 Angstrom
End of Basis Set

Basis Set
O.ANO-RCC-vtzp
O4 1.22522100 9.93113900 9.56746000 Angstrom
End of Basis Set

Basis Set
O.ANO-RCC-vtzp
O5 1.75652500 5.89205800 8.74773800 Angstrom
End of Basis Set

Basis Set
O.ANO-RCC-vtzp
O6 1.60135100 9.51194200 6.50182000 Angstrom
```

```
End of Basis Set

Basis Set
O.ANO-RCC-vtzp
O7 1.21159500 6.91777600 6.40048900 Angstrom
End of Basis Set

Basis Set
O.ANO-RCC-vdzp
O8 1.00477500 2.53138800 6.26891000 Angstrom
End of Basis Set

Basis Set
O.ANO-RCC-vtzp
O9 -0.98583600 6.97042900 8.84604400 Angstrom
End of Basis Set

Basis Set
O.ANO-RCC-vtzp
O10 -0.99509200 8.80720300 7.66485800 Angstrom
End of Basis Set

Basis Set
O.ANO-RCC-vdzp
O11 -0.63201900 4.60105000 7.59982400 Angstrom
End of Basis Set

Basis Set
O.ANO-RCC-vdzp
O12 3.44899700 4.65167800 6.18421500 Angstrom
End of Basis Set

Basis Set
O.ANO-RCC-vtzp
O13 3.54852900 8.26649900 8.32426500 Angstrom
End of Basis Set

Basis Set
N.ANO-RCC-vdzp
N14 0.08557600 0.93155100 3.24107900 Angstrom
End of Basis Set

Basis Set
N.ANO-RCC-vdzp
N15 0.14174200 -1.32847200 4.86540000 Angstrom
End of Basis Set

Basis Set
N.ANO-RCC-vtzp
N16 1.58665300 7.46455500 10.75772200 Angstrom
End of Basis Set

Basis Set
N.ANO-RCC-vdzp
N17 1.78989100 6.09153100 11.06927700 Angstrom
End of Basis Set

Basis Set
```

```
N.ANO-RCC-vdzp
N18 2.44356500 1.92993000 11.24925200 Angstrom
End of Basis Set

Basis Set
N.ANO-RCC-vdzp
N19 2.05104400 3.30700500 8.85360600 Angstrom
End of Basis Set

Basis Set
N.ANO-RCC-vdzp
N20 0.89677300 4.62332600 4.66576300 Angstrom
End of Basis Set

Basis Set
N.ANO-RCC-vdzp
N21 0.65267100 3.34143200 4.19843000 Angstrom
End of Basis Set

Basis Set
C.ANO-RCC-vdzp
C22 1.00291400 13.94895800 9.84725500 Angstrom
End of Basis Set

Basis Set
C.ANO-RCC-vdzp
C23 1.19756900 11.84486800 10.96189600 Angstrom
End of Basis Set

Basis Set
C.ANO-RCC-vdzp
C24 1.26631200 12.38354800 12.21265300 Angstrom
End of Basis Set

Basis Set
C.ANO-RCC-vdzp
C25 1.44510700 11.54515200 13.35754200 Angstrom
End of Basis Set

Basis Set
C.ANO-RCC-vdzp
C26 1.59162000 10.23693100 13.20781400 Angstrom
End of Basis Set

Basis Set
C.ANO-RCC-vdzp
C27 1.52604900 9.63952400 11.89504800 Angstrom
End of Basis Set

Basis Set
C.ANO-RCC-vdzp
C28 1.30542000 10.41716600 10.75620900 Angstrom
End of Basis Set

Basis Set
C.ANO-RCC-vdzp
C29 1.68129500 8.21789600 11.85572600 Angstrom
End of Basis Set
```

```

Basis Set
C.ANO-RCC-vdzp
C30 1.85851900 5.40299300 9.95614800 Angstrom
End of Basis Set

Basis Set
C.ANO-RCC-vdzp
C31 2.06444300 3.94288900 10.03479300 Angstrom
End of Basis Set

Basis Set
C.ANO-RCC-vdzp
C32 2.27018200 3.25637700 11.20993000 Angstrom
End of Basis Set

Basis Set
C.ANO-RCC-vdzp
C33 2.41049900 1.29809600 10.08319000 Angstrom
End of Basis Set

Basis Set
C.ANO-RCC-vdzp
C34 2.23950300 1.98663300 8.87931700 Angstrom
End of Basis Set

Basis Set
C.ANO-RCC-vdzp
C35 1.82739100 10.91736800 6.56382800 Angstrom
End of Basis Set

Basis Set
C.ANO-RCC-vdzp
C36 1.43882700 8.94086100 5.20871500 Angstrom
End of Basis Set

Basis Set
C.ANO-RCC-vdzp
C37 1.50073700 9.71242800 4.06231400 Angstrom
End of Basis Set

Basis Set
C.ANO-RCC-vdzp
C38 1.34448300 9.07654300 2.85995400 Angstrom
End of Basis Set

Basis Set
C.ANO-RCC-vdzp
C39 1.09956000 7.72579500 2.79643300 Angstrom
End of Basis Set

Basis Set
C.ANO-RCC-vdzp
C40 1.06369800 6.95017800 3.97610700 Angstrom
End of Basis Set

Basis Set
C.ANO-RCC-vdzp
```

```
C41 1.23211900 7.59213700 5.20266600 Angstrom
End of Basis Set

Basis Set
C.ANO-RCC-vdzp
C42 0.84336900 5.55892700 3.76890800 Angstrom
End of Basis Set

Basis Set
C.ANO-RCC-vdzp
C43 0.71880100 2.34710200 5.07562400 Angstrom
End of Basis Set

Basis Set
C.ANO-RCC-vdzp
C44 -0.18226100 -0.29364100 2.74198600 Angstrom
End of Basis Set

Basis Set
C.ANO-RCC-vdzp
C45 0.42256600 -0.11745600 5.32063300 Angstrom
End of Basis Set

Basis Set
C.ANO-RCC-vtzp
C46 -1.60812500 7.81489900 8.11706600 Angstrom
End of Basis Set

Basis Set
C.ANO-RCC-vdzp
C47 -2.99249800 7.65694100 7.79341200 Angstrom
End of Basis Set

Basis Set
C.ANO-RCC-vdzp
C48 4.68801500 4.84203800 6.91016900 Angstrom
End of Basis Set

Basis Set
C.ANO-RCC-vdzp
C49 0.40420400 0.98825400 4.54325800 Angstrom
End of Basis Set

Basis Set
C.ANO-RCC-vdzp
C50 -0.12635600 -1.40947700 3.55112200 Angstrom
End of Basis Set

Basis Set
H.ANO-RCC-vdzp
H51 1.07519900 14.29322600 8.95493700 Angstrom
End of Basis Set

Basis Set
H.ANO-RCC-vdzp
H52 1.74686200 14.25677400 10.37054600 Angstrom
End of Basis Set

```

```
Basis Set
H.ANO-RCC-vdzp
H53 0.18414300 14.25677400 10.24350500 Angstrom
End of Basis Set

Basis Set
H.ANO-RCC-vdzp
H54 1.19678700 13.30497300 12.32154600 Angstrom
End of Basis Set

Basis Set
H.ANO-RCC-vdzp
H55 1.45809500 11.91777200 14.20902500 Angstrom
End of Basis Set

Basis Set
H.ANO-RCC-vdzp
H56 1.73720100 9.69825200 13.95191600 Angstrom
End of Basis Set

Basis Set
H.ANO-RCC-vdzp
H57 1.86188100 7.78249800 12.65881200 Angstrom
End of Basis Set

Basis Set
H.ANO-RCC-vdzp
H58 2.29238800 3.73227800 12.00696600 Angstrom
End of Basis Set

Basis Set
H.ANO-RCC-vdzp
H59 2.50241500 0.37262000 10.06655300 Angstrom
End of Basis Set

Basis Set
H.ANO-RCC-vdzp
H60 2.25823600 1.51478200 8.07925600 Angstrom
End of Basis Set

Basis Set
H.ANO-RCC-vdzp
H61 2.55208100 11.15228100 5.97701600 Angstrom
End of Basis Set

Basis Set
H.ANO-RCC-vdzp
H62 2.05249800 11.16645700 7.46370800 Angstrom
End of Basis Set

Basis Set
H.ANO-RCC-vdzp
H63 1.03257800 11.38314300 6.29159600 Angstrom
End of Basis Set

Basis Set
H.ANO-RCC-vdzp
H64 1.64378000 10.62980200 4.10617400 Angstrom
```

```
End of Basis Set

Basis Set
H.ANO-RCC-vdzp
H65 1.40658000 9.56864500 2.07199200 Angstrom
End of Basis Set

Basis Set
H.ANO-RCC-vdzp
H66 0.95606900 7.32077300 1.97066100 Angstrom
End of Basis Set

Basis Set
H.ANO-RCC-vdzp
H67 0.64061600 5.29363800 2.90078900 Angstrom
End of Basis Set

Basis Set
H.ANO-RCC-vdzp
H68 -0.40505400 -0.38679600 1.84361900 Angstrom
End of Basis Set

Basis Set
H.ANO-RCC-vdzp
H69 0.64397800 -0.02227600 6.21900100 Angstrom
End of Basis Set

Basis Set
H.ANO-RCC-vdzp
H70 -3.07516000 7.26812000 6.92075500 Angstrom
End of Basis Set

Basis Set
H.ANO-RCC-vdzp
H71 -3.42323000 8.51558800 7.80248600 Angstrom
End of Basis Set

Basis Set
H.ANO-RCC-vdzp
H72 -3.40923400 7.08383500 8.44072000 Angstrom
End of Basis Set

Basis Set
H.ANO-RCC-vdzp
H73 5.40356200 4.40258900 6.44283600 Angstrom
End of Basis Set

Basis Set
H.ANO-RCC-vdzp
H74 4.60412400 4.46536800 7.79038700 Angstrom
End of Basis Set

Basis Set
H.ANO-RCC-vdzp
H75 4.87867600 5.77966400 6.97973900 Angstrom
End of Basis Set

Basis Set
```

```
H.ANO-RCC-vdzp
H76 -0.28178900 -2.24584700 3.17604600 Angstrom
End of Basis Set

Basis Set
H.ANO-RCC-vdzp
H77 -1.13276100 4.43499100 6.93134200 Angstrom
End of Basis Set

Basis Set
H.ANO-RCC-vdzp
H78 -0.85063100 5.35236600 7.93709000 Angstrom
End of Basis Set

Basis Set
H.ANO-RCC-vdzp
H79 3.63573900 4.41474000 5.38566700 Angstrom
End of Basis Set

Basis Set
H.ANO-RCC-vtzp
H80 4.09218800 7.94045600 8.89444100 Angstrom
End of Basis Set

Basis Set
H.ANO-RCC-vtzp
H81 3.98627600 8.66949600 7.71325500 Angstrom
End of Basis Set

End of Input

&RASSCF
  SYMM
    1
  SPIN
    11
  NACTEL
    14 0 0
  FROZ
    0
  INAC
    213
  RAS1
    0
  RAS2
    12
  RAS3
    0
  CIROOT
    11 11 1
  CIMX
    25
  ORBL
  ALL
  ORBA
  FULL
End of Input

```

```
>>> COPY $WorkDir/$Project.RasOrb
↪ $FileDir/DyMn_vtzp_vdzp_cas_14in12_rasscf_ms11_11r.RasOrb
>>> COPY $WorkDir/$Project.RunFile
↪ $FileDir/DyMn_vtzp_vdzp_cas_14in12_rasscf_ms11_11r.RunFile
>>> COPY $WorkDir/$Project.rasscf.h5
↪ $FileDir/DyMn_vtzp_vdzp_cas_14in12_rasscf_ms11_11r.rasscf.h5
>>> COPY $WorkDir/$Project.JobIph
↪ $FileDir/DyMn_vtzp_vdzp_cas_14in12_rasscf_ms11_11r.JobIph

>>COPY $Project.RasOrb INPORB
&RASSCF
  LUMO
  SYMM
    1
  SPIN
    9
  NACTEL
    14 0 0
  FROZ
    0
  INAC
    213
  RAS1
    0
  RAS2
    12
  RAS3
    0
  CIROOT
    11 11 1
  CIMX
    25
  ORBL
  ALL
  ORBA
  FULL
End of Input

>>> COPY $WorkDir/$Project.RasOrb
↪ $FileDir/DyMn_vtzp_vdzp_cas_14in12_rasscf_ms9_11r.RasOrb
>>> COPY $WorkDir/$Project.RunFile
↪ $FileDir/DyMn_vtzp_vdzp_cas_14in12_rasscf_ms9_11r.RunFile
>>> COPY $WorkDir/$Project.rasscf.h5
↪ $FileDir/DyMn_vtzp_vdzp_cas_14in12_rasscf_ms9_11r.rasscf.h5
>>> COPY $WorkDir/$Project.JobIph
↪ $FileDir/DyMn_vtzp_vdzp_cas_14in12_rasscf_ms9_11r.JobIph

&RASSCF
  LUMO
  SYMM
    1
  SPIN
    7
  NACTEL
    14 0 0
  FROZ
    0
  INAC
```

```
    213
  RAS1
    0
  RAS2
    12
  RAS3
    0
  CIROOT
    11 11 1
  CIMX
    25
  ORBL
  ALL
  ORBA
  FULL
End of Input

>>> COPY $WorkDir/$Project.RasOrb
↪ $FileDir/DyMn_vtzp_vdzp_cas_14in12_rasscf_ms7_11r.RasOrb
>>> COPY $WorkDir/$Project.RunFile
↪ $FileDir/DyMn_vtzp_vdzp_cas_14in12_rasscf_ms7_11r.RunFile
>>> COPY $WorkDir/$Project.rasscf.h5
↪ $FileDir/DyMn_vtzp_vdzp_cas_14in12_rasscf_ms7_11r.rasscf.h5
>>> COPY $WorkDir/$Project.JobIph
↪ $FileDir/DyMn_vtzp_vdzp_cas_14in12_rasscf_ms7_11r.JobIph


&RASSCF
  LUMO
  SYMM
    1
  SPIN
    5
  NACTEL
    14 0 0
  FROZ
    0
  INAC
    213
  RAS1
    0
  RAS2
    12
  RAS3
    0
  CIROOT
    11 11 1
  CIMX
    25
  ORBL
  ALL
  ORBA
  FULL
End of Input

>>> COPY $WorkDir/$Project.RasOrb
↪ $FileDir/DyMn_vtzp_vdzp_cas_14in12_rasscf_ms5_11r.RasOrb
>>> COPY $WorkDir/$Project.RunFile
```

```
$FileDir/DyMn_vtzp_vdzp_cas_14in12_rasscf_ms5_11r.RunFile
>>> COPY $WorkDir/$Project.rasscf.h5 $FileDir/DyMn_vtzp_vdzp_cas_14in12_rasscf_ms5_11r.rasscf.h5
>>> COPY $WorkDir/$Project.JobIph $FileDir/DyMn_vtzp_vdzp_cas_14in12_rasscf_ms5_11r.JobIph


&RASSCF
  LUMO
  SYMM
    1
  SPIN
    3
  NACTEL
    14 0 0
  FROZ
    0
  INAC
    213
  RAS1
    0
  RAS2
    12
  RAS3
    0
  CIROOT
    11 11 1
  CIMX
    25
  ORBL
  ALL
  ORBA
  FULL
End of Input

>>> COPY $WorkDir/$Project.RasOrb $FileDir/DyMn_vtzp_vdzp_cas_14in12_rasscf_ms3_11r.RasOrb
>>> COPY $WorkDir/$Project.RunFile $FileDir/DyMn_vtzp_vdzp_cas_14in12_rasscf_ms3_11r.RunFile
>>> COPY $WorkDir/$Project.rasscf.h5 $FileDir/DyMn_vtzp_vdzp_cas_14in12_rasscf_ms3_11r.rasscf.h5
>>> COPY $WorkDir/$Project.JobIph $FileDir/DyMn_vtzp_vdzp_cas_14in12_rasscf_ms3_11r.JobIph


&RASSCF
  LUMO
  SYMM
    1
  SPIN
    1
  NACTEL
    14 0 0
  FROZ
    0
  INAC
    213
  RAS1
```

```
    0
  RAS2
    12
  RAS3
    0
  CIROOT
    11  11  1
  CIMX
    25
  ORBL
  ALL
  ORBA
  FULL
End of Input

>>> COPY $WorkDir/$Project.RasOrb
↪ $FileDir/DyMn_vtzp_vdzp_cas_14in12_rasscf_ms1_11r.RasOrb
>>> COPY $WorkDir/$Project.RunFile
↪ $FileDir/DyMn_vtzp_vdzp_cas_14in12_rasscf_ms1_11r.RunFile
>>> COPY $WorkDir/$Project.rasscf.h5
↪ $FileDir/DyMn_vtzp_vdzp_cas_14in12_rasscf_ms1_11r.rasscf.h5
>>> COPY $WorkDir/$Project.JobIph
↪ $FileDir/DyMn_vtzp_vdzp_cas_14in12_rasscf_ms1_11r.JobIph




>>> COPY $FileDir/DyMn_vtzp_vdzp_cas_14in12_rasscf_ms11_11r.JobIph $WorkDir/JOB001
>>> COPY $FileDir/DyMn_vtzp_vdzp_cas_14in12_rasscf_ms9_11r.JobIph $WorkDir/JOB002
>>> COPY $FileDir/DyMn_vtzp_vdzp_cas_14in12_rasscf_ms7_11r.JobIph $WorkDir/JOB003
>>> COPY $FileDir/DyMn_vtzp_vdzp_cas_14in12_rasscf_ms5_11r.JobIph $WorkDir/JOB004
>>> COPY $FileDir/DyMn_vtzp_vdzp_cas_14in12_rasscf_ms3_11r.JobIph $WorkDir/JOB005
>>> COPY $FileDir/DyMn_vtzp_vdzp_cas_14in12_rasscf_ms1_11r.JobIph $WorkDir/JOB006

&RASSI
  OMEG
  J-VA
  MEES
  Prop
    6
  AngMom  1
  AngMom  2
  AngMom  3
  MLTPL1  1
  MLTPL1  2
  MLTPL1  3
  SpinOrbit
  NR  OF  JOBIPHS
    6  11  11  11   11  11  11
    1  2  3  4  5  6  7  8  9  10  11
    1  2  3  4  5  6  7  8  9  10  11
    1  2  3  4  5  6  7  8  9  10  11
    1  2  3  4  5  6  7  8  9  10  11
    1  2  3  4  5  6  7  8  9  10  11
    1  2  3  4  5  6  7  8  9  10  11
  EJOB
End of Input
>>> COPY $WorkDir/$Project.rassi.h5
```

```
↪ $FileDir/DyMn_vtzp_vdzp_cas_14in12_396r_rassi_cas.h5
>>> COPY $WorkDir/$Project.RunFile
↪ $FileDir/DyMn_vtzp_vdzp_cas_14in12_396r_rassi_cas.RunFile

&SINGLE_ANISO
MLTP
7
2 2 2 2 2 2 2
PLOT
TINT
0 300 301
XFIE
0.50
HINT
0 7.0 71
TMAG
5  2.0 3.0 5.0 8.0 10.0
MAVE
1 10
UBAR
End Of Input
>>> COPY $WorkDir/$Project.aniso
↪ $FileDir/DyMn_vtzp_vdzp_cas_14in12_396r_aniso_cas.aniso
>>> COPY $WorkDir/$Project.MH.dat
↪ $FileDir/DyMn_vtzp_vdzp_cas_14in12_396r_aniso_cas_MH.dat
>>> COPY $WorkDir/$Project.MH.plt
↪ $FileDir/DyMn_vtzp_vdzp_cas_14in12_396r_aniso_cas_MH.plt
>>> COPY $WorkDir/$Project.MH.png
↪ $FileDir/DyMn_vtzp_vdzp_cas_14in12_396r_aniso_cas_MH.png
>>> COPY $WorkDir/$Project.XT_no_field.dat
↪ $FileDir/DyMn_vtzp_vdzp_cas_14in12_396r_aniso_cas_XT_no_field.dat
>>> COPY $WorkDir/$Project.XT_no_field.plt
↪ $FileDir/DyMn_vtzp_vdzp_cas_14in12_396r_aniso_cas_XT_no_field.plt
>>> COPY $WorkDir/$Project.XT_no_field.png
↪ $FileDir/DyMn_vtzp_vdzp_cas_14in12_396r_aniso_cas_XT_no_field.png
>>> COPY $WorkDir/$Project.XT_with_field_dM_over_dH.dat
↪ $FileDir/DyMn_vtzp_vdzp_cas_14in12_396r_aniso_cas_XT_with_field_dM_over_dH.dat
>>> COPY $WorkDir/$Project.XT_with_field_dM_over_dH.plt
↪ $FileDir/DyMn_vtzp_vdzp_cas_14in12_396r_aniso_cas_XT_with_field_dM_over_dH.plt
>>> COPY $WorkDir/$Project.XT_with_field_dM_over_dH.png
↪ $FileDir/DyMn_vtzp_vdzp_cas_14in12_396r_aniso_cas_XT_with_field_dM_over_dH.png
>>> COPY $WorkDir/$Project.XT_with_field_M_over_H.dat
↪ $FileDir/DyMn_vtzp_vdzp_cas_14in12_396r_aniso_cas_XT_with_field_M_over_H.dat
>>> COPY $WorkDir/$Project.XT_with_field_M_over_H.plt
↪ $FileDir/DyMn_vtzp_vdzp_cas_14in12_396r_aniso_cas_XT_with_field_M_over_H.plt
>>> COPY $WorkDir/$Project.XT_with_field_M_over_H.png
↪ $FileDir/DyMn_vtzp_vdzp_cas_14in12_396r_aniso_cas_XT_with_field_M_over_H.png
>>> COPY $WorkDir/$Project.old.aniso
↪ $FileDir/DyMn_vtzp_vdzp_cas_14in12_396r_aniso_cas.old.aniso
```

Listing S19: Input file showing the function *PREX*.

```
>>COPY $FileDir/DyMn_vtzp_vdzp_cas_14in12_396r_aniso_cas.aniso        $WorkDir/
>>COPY $FileDir/Dy_in_DyMn_vtzp_vdzp_cas_9in7_aniso_cas.old.aniso     $WorkDir/
>>COPY $FileDir/Mn_in_DyMn_vtzp_vdzp_cas_5in5_aniso_cas.old.aniso     $WorkDir/

&SINGLE_ANISO
DATA
```

```
DyMn_vtzp_vdzp_cas_14in12_396r_aniso_cas.aniso
PREX
2 Dy_in_DyMn_vtzp_vdzp_cas_9in7_aniso_cas.old.aniso
6 Mn_in_DyMn_vtzp_vdzp_cas_5in5_aniso_cas.old.aniso
TINT
0 300 31
MLTP
6
2 2 2  2 2 2
End Of Input
```

S6 Relativistic Hyperfine Coupling

In listing S20 – a part of the verification test suite, `test/extra/396.input` – an input sample to perform a hyperfine coupling calculation using the `GEN1INT` library for the relevant one-electron integrals is given. To activate the hyperfine coupling features, `OpenMolcas` must be compiled with the `GEN1INT` option (e.g., `cmake -D GEN1INT=ON`). In the `OpenMolcas` `SEWARD` module, the ***FLDG=0*** keyword indicates that hyperfine coupling constants will be calculated for each nucleus, ***AMFI*** requests the computation of the atomic mean-field integrals for the inclusion of spin–orbit coupling effects in the `RASSI` module, ***MXTC*** indicates the calculation of X2C transformed hyperfine magnetic integrals to be used in the hyperfine calculations, note that ***MXTC*** must be used together with the ***RX2C*** keyword, and finally ***clight*** specifies the speed of light. For SCF or DFT calculations, the ***HFC*** keyword activates the hyperfine coupling tensor matrix calculation on each atom, and the ***HFC*** keyword must be used in conjunction with UHF.

With the `RASSI` module, the following keywords are required: ***SPIN*** requests the spin–orbit interaction matrix elements and ***EPRA*** computes the hyperfine tensor matrix and principal magnetic axes values for the ground spin–orbit state. For the hyperfine matrix elements, calculating both spin-dependent (ASD) and the paramagnetic spin–orbital (PSOP) parts is recommended. ASD and PSOS are calculated using the ***PROP*** keyword. Note that there are 6 ASD and 3 PSOP components for each atom. The following keywords are optional: ***AFCC*** gives separate Fermi contact contributions, ***ASDC*** the spin-dipolar contributions, ***FCSD*** the total spin-dependent contributions (AFCC+ASDC), ***APSO*** computes the paramagnetic spin–orbital contributions independently. Note that keywords ***AFCC***, ***ASDC***, and ***FCSD*** require the ASD part of the hyperfine coupling matrix, while ***APSO*** requires the PSOP part. Finally, ***ATSA*** activates the pseudospin approach to calculate the hyperfine constants for non-doublet states. If the ***ATSA*** keyword is not present, but a state has more than two components, the code will exit with an error. If the ground state is a Kramers doublet, the pseudospin approach will result in the same hyperfine coupling as the standard approach.

Listing S20: `OpenMolcas` input for an HFC calculation.

```
&SEWARD
FINIte
cholesky
BSSHOW
Basis set
Si.cc-pVTZ-DK..15s9p2d1f.5s4p2d1f.
Si     1.641895   -0.007260    0.000000   angstrom
End of basis
```

```
Basis set
S.cc-pVTZ-DK..15s9p2d1f.5s4p2d1f.
S      -0.486365   -0.002825    0.000000   angstrom
end of basis
Basis set
H.cc-pVTZ-DK..5s2p1d.3s2p1d.
H      -0.758580   1.333849    0.000000    angstrom
end of basis
RX2C
clight
137.036
Angmom
  0.0 0.0 0.0
FLDG
  0
AMFI
MXTC
End of input

&SCF
UHF
HFC
THREsholds
1.0d-10 1.0d-8 1.5d-4 0.2d-4
charge=0
End of input

&RASSCF
Title= The CASSCF of NpF6 with an active space of (1,8)
Spin
2
Symmetry
1
nActEl
1 0 0
Inactive
15
Ras2
8
CIRoots
8 8 1
Iter
100 100
OrbAppear
Compact
LumOrb
levshift
1.0
End of input

&RASSI
NrofJobIphs
 1 8
 1 2 3 4 5 6 7 8
SPIN
EPRA
AFCC
ASDC
```

```
FCSD
APSO
ATSA
PROPerties
  27
'ASD    1' 1
'ASD    1' 2
'ASD    1' 3
'ASD    1' 4
'ASD    1' 5
'ASD    1' 6
'ASD    2' 1
'ASD    2' 2
'ASD    2' 3
'ASD    2' 4
'ASD    2' 5
'ASD    2' 6
'ASD    3' 1
'ASD    3' 2
'ASD    3' 3
'ASD    3' 4
'ASD    3' 5
'ASD    3' 6
'PSOP   1' 1
'PSOP   1' 2
'PSOP   1' 3
'PSOP   2' 1
'PSOP   2' 2
'PSOP   2' 3
'PSOP   3' 1
'PSOP   3' 2
'PSOP   3' 3
end of input
```

S7 Single and Two-Photon Spectra with the Exact Semi-Classical Operator

Input files for performing photon-in photon-out calculations using the exact semi-classical light–matter operator. Notice that the inputs files are for a RASPT2 calculations, but this methodology works with RASSCF too.

Listing S21: `tpu.xyz`: $Fe^{II}(P)(ImH)_2$ geometry.

```
55
FeII(P)(ImH)2 B3LYP/6-32G(d) geometry
FE        0.00000000   0.00000000   0.00000000
N        -1.41871810   1.42829924  -0.05008112
C        -2.77860804   1.23671423  -0.04241476
C        -3.47045523   2.50649453  -0.08145791
H        -4.54733899   2.62348940  -0.08542147
C        -2.51334757   3.47244019  -0.11097290
H        -2.63967034   4.54784164  -0.14500326
C        -1.23755992   2.79042795  -0.09118545
C         0.00000000   3.42986011  -0.10875308
H         0.00000000   4.51616347  -0.14255035
```

```
C        3.41304412   0.00000000   0.00000000
H        4.49975735   0.00000000   0.00000000
N        0.00000000   0.07346165   2.01412485
C        0.00000000   1.16913437   2.75418232
H        0.00000000   2.18010867   2.37849152
N        0.00000000   0.85276984   4.07481867
H        0.00000000   1.51393411   4.83664673
C        0.00000000  -0.52475535   4.18191792
H        0.00000000  -1.02921946   5.13541076
C        0.00000000  -0.99044340   2.89607911
H        0.00000000  -2.00856863   2.54191494
N       -1.41871810  -1.42829924   0.05008112
N        1.41871810  -1.42829924   0.05008112
N        1.41871810   1.42829924  -0.05008112
C       -2.77860804  -1.23671423   0.04241476
C        2.77860804  -1.23671423   0.04241476
C        2.77860804   1.23671423  -0.04241476
C       -3.47045523  -2.50649453   0.08145791
C        3.47045523  -2.50649453   0.08145791
C        3.47045523   2.50649453  -0.08145791
H       -4.54733899  -2.62348940   0.08542147
H        4.54733899  -2.62348940   0.08542147
H        4.54733899   2.62348940  -0.08542147
C       -2.51334757  -3.47244019   0.11097290
C        2.51334757  -3.47244019   0.11097290
C        2.51334757   3.47244019  -0.11097290
H       -2.63967034  -4.54784164   0.14500326
H        2.63967034  -4.54784164   0.14500326
H        2.63967034   4.54784164  -0.14500326
C       -1.23755992  -2.79042795   0.09118545
C        1.23755992  -2.79042795   0.09118545
C        1.23755992   2.79042795  -0.09118545
C        0.00000000  -3.42986011   0.10875308
H        0.00000000  -4.51616347   0.14255035
C       -3.41304412   0.00000000   0.00000000
H       -4.49975735   0.00000000   0.00000000
N        0.00000000  -0.07346165  -2.01412485
C        0.00000000  -1.16913437  -2.75418232
H        0.00000000  -2.18010867  -2.37849152
N        0.00000000  -0.85276984  -4.07481867
H        0.00000000  -1.51393411  -4.83664673
C        0.00000000   0.52475535  -4.18191792
H        0.00000000   1.02921946  -5.13541076
C        0.00000000   0.99044340  -2.89607911
H        0.00000000   2.00856863  -2.54191494
```

Listing S22: `fe2-ce.input`: Input for computing the ground state and 1s and 2p core-excitations. It calls for a pre-computed reference orbital file (`ref.RasOrb`).

```
*-----------------------------------------------------------------------
* fe2-ce.input - Compute the ground state and 1s and 2p core-excitations
*.               and save the JobMix files.
*-----------------------------------------------------------------------
&GATEWAY
TITLE = "RIXS map of FeII(P)(ImH)2 using the exact operator"
COORD = tpu.xyz
Basis = ANO-RCC-VDZP
RICD
```

```
&SEWARD
*-----------------------------------------------------
** Ground state
*-----------------------------------------------------
>>> COPY $CurrDir/ref.RasOrb INPORB
&RASSCF
LUMORB
SYMMETRY
 1
Spin
 1
Charge
 0
RASSCF
 1 2
SupSym
 1 ; 1 45
 0 ;
 1 ; 1 25
 1 ; 2 39 40
CIROot
 1 1 1
>>> COPY $Project.RasOrb $CurrDir/fe2-gs.RasOrb
*------
&CASPT2
MULTistate
 All
Imaginary
 0.3
MAXIter
 200
>>> COPY $Project.JobMix $CurrDir/$Project.gs1.JobMix
*-----------------------------------------------------
** 1s core-excitation
*-----------------------------------------------------
>>> foreach SYMMETRY in ( 1, 2 )
>>> COPY $CurrDir/fe2-gs.RasOrb INPORB
&RASSCF
LUMORB
SYMMETRY
 $SYMMETRY
Spin
 1
Charge
 0
RASSCF
 1 2
SUPSYM
 1 ; 1 45
 0 ;
 1 ; 1 25
 1 ; 2 39 40
HEXS
 1
 3
CIROot
 60 60 1
*-------
```

```
&CASPT2
MULTistate
 All
Imaginary
 0.3
MAXIter
 200
>>> COPY $Project.JobMix $CurrDir/$Project.1s.s$SYMMETRY.$SPIN.JobMix
>>> end do
*----------------------------------------------------
** 2p core-excitation
*----------------------------------------------------
>>> foreach SYMMETRY in ( 3, 4 )
>>> foreach SPIN in ( 1, 3 )
>>> COPY $CurrDir/fe2-gs.RasOrb INPORB
&RASSCF
LUMORB
SYMMETRY
 3
Spin
 1
Charge
 0
RASSCF
 1 2
SUPSYM
 1 ; 1 45
 0 ;
 1 ; 1 25
 1 ; 2 39 40
CIROot
 120 120 1
*---------
&CASPT2
MULTistate
 All
Imaginary
 0.3
MAXIter
 200
>>> COPY $Project.JobMix $CurrDir/$Project.2p.s$SYMMETRY.$SPIN.JobMix
>>> end do
>>> end do
*-------------------------------------------------------------------
```

Listing S23: `fe2-rixs-exact.input`: Input for computing the RIXS maps using the full exact operator. It calls for previously computed ground and core-excited states `JobMix` files as described above.

```
*--------------------------------------------------------------------------------
* fe2-rixs-exact.input - Compute the RIXS map with the full exact operator.
*--------------------------------------------------------------------------------
&GATEWAY
TITLE = "RIXS map of FeII(P)(ImH)2 using the exact operator"
COORD = tpu.xyz
Basis = ANO-RCC-VDZP
RICD
angmom
```

```
 0.00 0.00 0.00
omqi
 0.00 0.00 0.00
*--------------------------------------------------
&SEWARD
multipole
 3
center
 3
 1 0.0 0.0 0.0
 2 0.0 0.0 0.0
 3 0.0 0.0 0.0
*------------------------------------------------------
>>> COPY $CurrDir/fe2-ce.gs.JobMix       JOB001
>>> COPY $CurrDir/fe2-ce.2p.s3.1.JobMix  JOB002
>>> COPY $CurrDir/fe2-ce.2p.s4.1.JobMix  JOB003
>>> COPY $CurrDir/fe2-ce.2p.s3.3.JobMix  JOB004
>>> COPY $CurrDir/fe2-ce.2p.s4.3.JobMix  JOB005
>>> COPY $CurrDir/fe2-ce.1s.s1.JobMix    JOB006
>>> COPY $CurrDir/fe2-ce.1s.s2.JobMix    JOB007
*------------------------------------------------------
&RASSI  &END
Ejob
NROF jobiphs
7 All
SUBS
481
TINT
QIALL
qipr
1e-8
SPINorbit
*---------------------------------------------------
```

Listing S24: `fe2-rixs-exact-group.input`: Input for computing the RIXS maps using the exact operator with the grouping scheme.

```
*--------------------------------------------------------------------------------
* fe2-rixs-exact-group.input -
* Compute the RIXS map with the exact operator and grouping scheme.
*--------------------------------------------------------------------------------
&GATEWAY
TITLE = "RIXS map of FeII(P)(ImH)2 using the exact operator"
COORD = tpu.xyz
Basis = ANO-RCC-VDZP
RICD
angmom
 0.00 0.00 0.00
omqi
 0.00 0.00 0.00
*--------------------------------------------------
&SEWARD
multipole
 3
center
 3
 1 0.0 0.0 0.0
 2 0.0 0.0 0.0
```

```
 3 0.0 0.0 0.0
*------------------------------------------------------
>>> COPY $CurrDir/fe2-ce.gs.JobMix       JOB001
>>> COPY $CurrDir/fe2-ce.2p.s3.1.JobMix  JOB002
>>> COPY $CurrDir/fe2-ce.2p.s4.1.JobMix  JOB003
>>> COPY $CurrDir/fe2-ce.2p.s3.3.JobMix  JOB004
>>> COPY $CurrDir/fe2-ce.2p.s4.3.JobMix  JOB005
>>> COPY $CurrDir/fe2-ce.1s.s1.JobMix    JOB006
>>> COPY $CurrDir/fe2-ce.1s.s2.JobMix    JOB007
*------------------------------------------------------
&RASSI  &END
Ejob
NROF jobiphs
7 All
SUBS
481
TINT
TIGr
0.001
QIALL
qipr
1e-8
SPINorbit
*---------------------------------------------------
```

S8 Vibrational–Rotational Levels for Diatomics

An input example for a LEVEL calculation is given in listing S25, and an excerpt of the output in listing S26.

Listing S25: Input example for a LEVEL calculation.

```
&LEVEL

 IAN1   =  3                                 # Atomic number for atom 1
 IMN1   =  6                                 # Mass   number for atom 1
 IAN2   =  3                                 # Atomic number for atom 2
 IMN2   =  6                                 # Mass   number for atom 2
 CHARGE =  0                                 # Charge of the molecule
 VLIM   =  0.0d0                             # Energy at dissociation (cm-1)
 RH     =  0.0005                            # ODE step size
 RMIN   =  0.125                             # ODE lower limit
 PPAR   =  5                                 # p
 QPAR   =  3                                 # q
 MMLR   =  6 8 10                            # Long-range inverse powers
 DSCM   =  333.768                           # Depth  at equilibrium (cm-1)
 REQ    =  4.17001                           # Length at equilibrium (Angstrom)
 RREF   =  8.0d0                             # Reference distance    (Angstrom)
 CMM    =  6.719e6  1.12635e8 2.78694e9      # Long-range constants (C6,C8,C10)
 PARM   = -5.16e-1 -9.59e-2 1.17e-1 -2.28e-2 # Beta 0, Beta 1, Beta 2, Beta 3
 LCDC   = 0
```

Listing S26: Output excerpt for a LEVEL calculation.

```
 SUMMARY (ALL ENERGIES IN CM-1):

```

```
 Find  10 Potential-1 vibrational levels with  J=  0
     v     E(v)         v     E(v)         v     E(v)         v     E(v)
   --------------   --------------   --------------   --------------
     0   -299.3817      3   -133.8550      6    -34.6437      9     -0.8188
     1   -236.6922      4    -93.4264      7    -16.4357
     2   -181.5854      5    -60.3267      8     -5.4964

 An  n= 6  N-D theory extrapolation from last 2 levels implies   vD =  10.128
```

S9 Evaluating Frenkel's Excitonic Coupling Terms

Computational details and results for the applications discussed in section 3.6.

S9.1 Azulene

S9.1.1 Monomer geometries and energetics

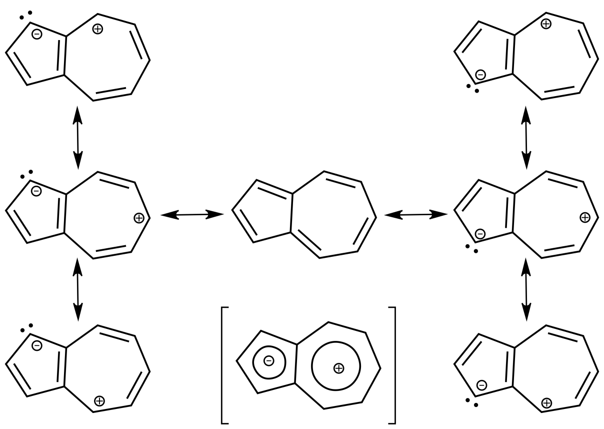

Figure S4: **Azulene molecule.** A few of the azulene resonance structures are shown here, together with the corresponding resonance hybrid (bottom middle). Only those formulas with charge separation imply an aromatic compound, hence they are the most important ones. Aromaticity induces a dipole from the 7-membered to the 5-membered ring.

Azulene is an aromatic molecule, constituted by five-membered and seven-membered fused rings, with structure as shown in fig. S4. Azulene electronic excitation spectrum is characterized by both $\pi \rightarrow \pi^*$ and $\pi \rightarrow$ Rydberg transitions. Thus, an extra basis set, specifically tailored for the azulene molecule, was created using the module `GENANO`, starting from a general Rydberg basis set.[S7] The obtained basis set (which we called AZURYDBERG, listing S27) provides one basis function for each s, p_x, p_y, p_z orbitals, and is applied to a ghost atom (X) located on the molecule centre of mass.

The geometry of azulene was optimized in vacuo at the SA-CASSCF level of theory,[S8] using the triple zeta basis ANO-RCC-VTZP,[S9] plus AZURYDBERG. State averaging was done over 8 states to preserve the Rydberg virtual orbitals inside the active space, although the optimization was, obviously, performed on the ground state. The active space comprised 10 electrons and 14 RAS2 orbitals (table S4), of which there were 5 π, 5 π^*, and 4 Rydberg virtual (s-type, $p_{x,y,z}$-type). We used a level shift of $0.1\,E_\mathrm{h}$ to help wave function optimization convergence.

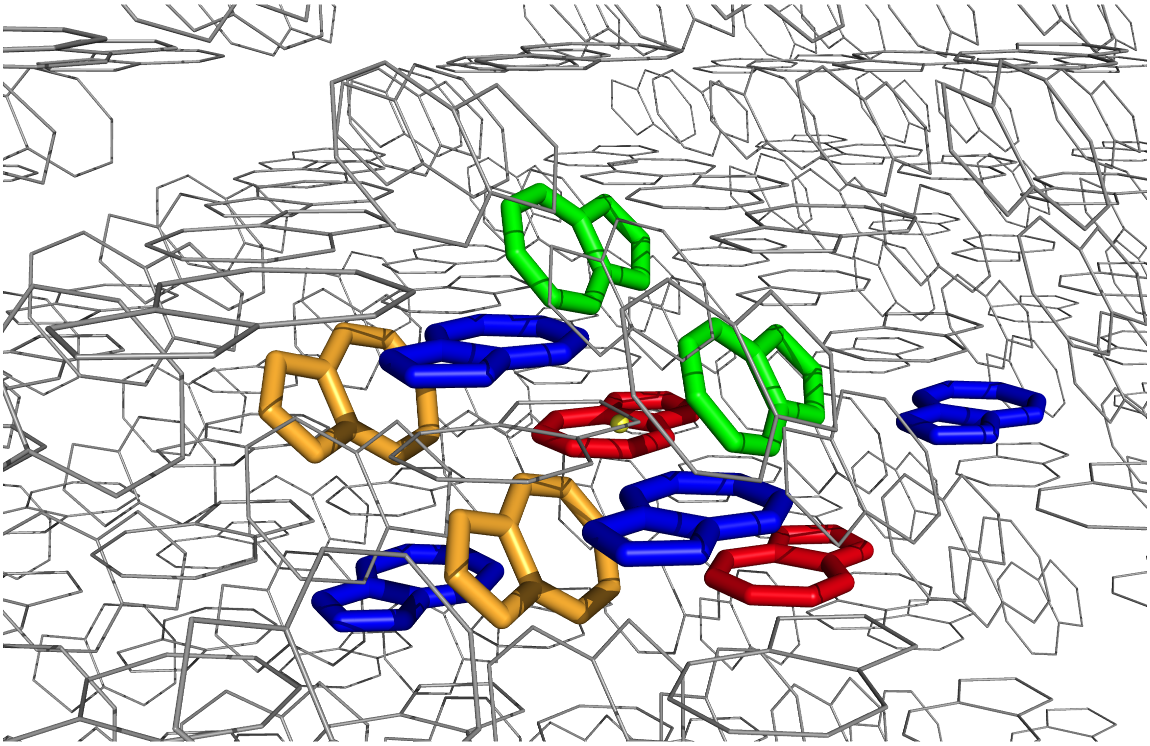

Figure S5: **Azulene crystal representation.** Among the 500 molecules azulene $5\times5\times5$ supercell (grey lines, only a fraction is shown here), we highlighted four different types, which we refer to as: flat (red sticks), inverted flat (blue sticks), tilted (green sticks), and inverted titled (orange sticks). Hydrogen atoms are hidden for the sake of readability. We defined all possible combinations of unique dimers as those found within 25 Å from the flat azulene monomer in the middle of the figure, highlighted by a yellow dot. These sets of dimers were later used to compute excitonic couplings, to be extended to the rest of the crystal.

The optimized geometry (listing S28) corresponded to the reference "flat" geometry highlighted in fig. S5. Azulene monomers inside the crystal structure[S10] can have four different orientations, which we refer to as "flat", "inverted flat", "tilted", and "inverted tilted", as shown in fig. S5. Thus, the flat optimized geometry was rigidly projected onto the other three different orientations (listings S29 to S31).

Single point energy evaluations were performed for the four monomer orientations, at the same level of theory as the previous geometry optimization (8roots-SA-CASSCF, active space with 10 electrons in 14 orbitals). Energies were subsequently re-evaluated at the MS-CASPT2 level of theory,[S11] with default IPEA shift value $0.25\,E_\mathrm{h}$,[S12] and an imaginary level shift of $0.1\,E_\mathrm{h}$.[S13] Computed energetics for the azulene monomer are reported in table S5. All calculation used Cholesky decomposition for speed up,[S14–S16] and no symmetry constraint.

Listing S27: Rydberg extra basis functions specifically tailored for azulene.

```
/X.AZURYDBERG..8s8p8d.1s1p1d.
Rydberg orbitals for Azulene
GHOST (8s,8p,8d) -> [1s,1p,1d]
     0.0   0
* s-type functions
    8    1
    0.02462393256690
    0.01125334281667
    0.00585838058488
    0.00334597391176
    0.00204842249316
    0.00132364236431
    0.00089309575944
    0.00062431294198
      0.3776806518
     -1.1846097118
      0.1486302928
     -0.6983559229
      0.8163346267
     -0.6988936683
      0.3755558467
     -0.0930378890
* p-type functions
    8    1
    0.04233528041629
    0.01925420565070
    0.00998821043479
    0.00568936060666
    0.00347567961959
    0.00224205895181
    0.00151063985318
    0.00105475270898
      0.1524651841
     -0.0598750355
     -0.8289270600
     -0.0490334535
     -0.3174558759
      0.2469509609
     -0.1398163117
      0.0370466341
* d-type functions
    8    1
```

```
    0.06054020300665
    0.02744569273592
    0.01420439913628
    0.00807659325172
    0.00492718647974
    0.00317481115512
    0.00213712310185
    0.00149101554039
      0.0557392505
     -0.0066265130
     -0.3854752598
     -0.3837364797
     -0.3078424907
      0.0203538284
     -0.0471536110
      0.0125574710
```

Table S4: Active space orbitals for azulene. Electronic occupation for each state is given under each orbital (S_0–S_7 from top to bottom). Density contour value = 0.0121.

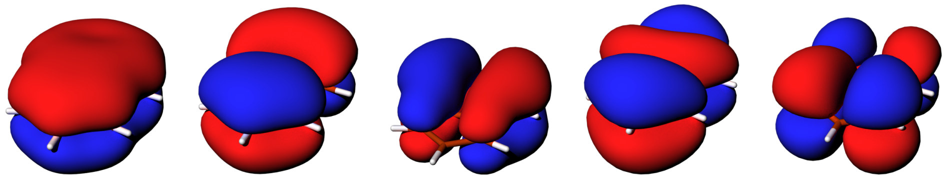

S_0	1.958	1.939	1.934	1.827	1.820
S_1	1.944	1.913	1.874	1.786	1.108
S_2	1.954	1.926	1.901	1.866	1.005
S_3	1.938	1.917	1.837	1.533	1.368
S_4	1.954	1.926	1.900	1.867	1.007
S_5	1.931	1.896	1.691	1.390	1.556
S_6	1.954	1.926	1.902	1.863	1.004
S_7	1.947	1.923	1.851	1.790	1.102

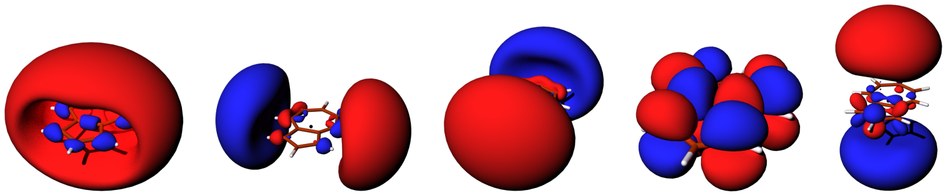

S_0	0.000	0.000	0.000	0.047	0.000
S_1	0.000	0.000	0.000	0.059	0.001
S_2	0.989	0.005	0.006	0.032	0.000
S_3	0.000	0.000	0.000	0.055	0.002
S_4	0.006	0.993	0.001	0.031	0.000
S_5	0.000	0.000	0.000	0.061	0.173
S_6	0.009	0.001	0.990	0.032	0.000
S_7	0.000	0.000	0.000	0.038	0.858

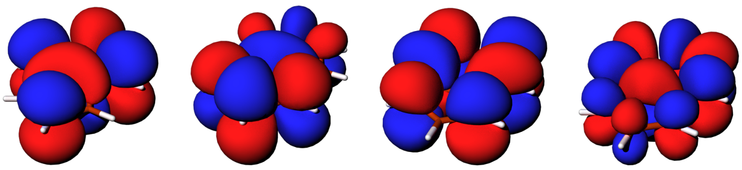

S_0	0.183	0.063	0.184	0.045
S_1	0.908	0.094	0.247	0.065
S_2	0.125	0.060	0.095	0.037
S_3	0.585	0.098	0.595	0.072
S_4	0.123	0.059	0.094	0.038
S_5	0.631	0.084	0.521	0.065
S_6	0.127	0.058	0.096	0.038
S_7	0.227	0.066	0.152	0.046

Listing S28: Atom coordinates for the optimized flat geometry (in Å). X is a label for the center of mass of the molecule.

```
C1  15.0157279  1.1938718  9.1716026
C2  14.7261655  0.2530861 10.1694956
C3  14.0281222 -0.8266259  9.6117327
C4  13.2166484 -1.4309588  7.3449705
C5  13.0143820 -1.2561629  5.9744097
C6  13.4083353 -0.1933988  5.1607788
C7  14.1038444  0.9708566  5.4892623
C8  14.5905183  1.3781646  6.7329156
C9  14.5039863  0.7267595  7.9545605
C10 13.8599947 -0.5905520  8.2414124
H1  15.5391949  2.1168250  9.3072465
H2  14.9970753  0.3454082 11.2013624
H3  13.6779839 -1.6905151 10.1364765
H4  12.8205927 -2.3388077  7.7634050
H5  12.4835158 -2.0469373  5.4789478
H6  13.1353647 -0.2853835  4.1256077
H7  14.2896664  1.6449384  4.6745772
H8  15.1012124  2.3242710  6.7475135
X   14.0398182  0.0227078  7.5707620
```

Listing S29: Atom coordinates for the optimized inverted flat geometry (in Å). X is a label for the center of mass of the molecule.

```
C1  -15.015728 -1.1938718  -9.1716026
C2  -14.726166 -0.2530861 -10.1694960
C3  -14.028122  0.8266259  -9.6117327
C4  -13.216648  1.4309588  -7.3449705
C5  -13.014382  1.2561629  -5.9744097
C6  -13.408335  0.1933988  -5.1607788
C7  -14.103844 -0.9708566  -5.4892623
C8  -14.590518 -1.3781646  -6.7329156
C9  -14.503986 -0.7267595  -7.9545605
C10 -13.859995  0.5905520  -8.2414124
H1  -15.539195 -2.1168250  -9.3072465
H2  -14.997075 -0.3454082 -11.2013620
H3  -13.677984  1.6905151 -10.1364770
H4  -12.820593  2.3388077  -7.7634050
H5  -12.483516  2.0469373  -5.4789478
H6  -13.135365  0.2853835  -4.1256077
H7  -14.289666 -1.6449384  -4.6745772
H8  -15.101212 -2.3242710  -6.7475135
X   -14.039818 -0.0227078  -7.5707620
```

Listing S30: Atom coordinates for the optimized tilted geometry (in Å). X is a label for the center of mass of the molecule.

```
C1  -3.6573802 -11.1260710 13.1852123
C2  -3.9411660 -10.1823646 14.1820064
C3  -4.6333022  -9.0994265 13.6231375
C4  -5.4407573  -8.4926124 11.3556033
C5  -5.6422589  -8.6667935  9.9848515
C6  -5.2539342  -9.7324276  9.1722693
C7  -4.5670538 -10.9013218  9.5024309
C8  -4.0843632 -11.3109369 10.7468785
C9  -4.1666637 -10.6574668 11.9677127
```

```
C10 -4.8029223  -9.3360862 12.2531023
H1  -3.1391512 -12.0518305 13.3218420
H2  -3.6701514 -10.2747875 15.2138366
H3  -4.9780748  -8.2327633 14.1468590
H4  -5.8319284  -7.5822292 11.7731255
H5  -6.1674074  -7.8729032  9.4882806
H6  -5.5247077  -9.6391174  8.1366398
H7  -4.3850446 -11.5773077  8.6884630
H8  -3.5803263 -12.2605860 10.7627638
X   -4.6199504  -9.9475698 11.5816821
```

Listing S31: Atom coordinates for the optimized inverted tilted geometry (in Å). X is a label for the center of mass of the molecule.

```
C1  3.6573802 11.1260710 -13.1852123
C2  3.9411660 10.1823646 -14.1820064
C3  4.6333022  9.0994265 -13.6231375
C4  5.4407573  8.4926124 -11.3556033
C5  5.6422589  8.6667935  -9.9848515
C6  5.2539342  9.7324276  -9.1722693
C7  4.5670538 10.9013218  -9.5024309
C8  4.0843632 11.3109369 -10.7468785
C9  4.1666637 10.6574668 -11.9677127
C10 4.8029223  9.3360862 -12.2531023
H1  3.1391512 12.0518305 -13.3218420
H2  3.6701514 10.2747875 -15.2138366
H3  4.9780748  8.2327633 -14.1468590
H4  5.8319284  7.5822292 -11.7731255
H5  6.1674074  7.8729032  -9.4882806
H6  5.5247077  9.6391174  -8.1366398
H7  4.3850446 11.5773077  -8.6884630
H8  3.5803263 12.2605860 -10.7627638
X   4.6199504  9.9475698 -11.5816821
```

Table S5: Computed energetics for the azulene monomer. Oscillator strengths for the $S_0 \rightarrow S_4$ and $S_0 \rightarrow S_6$ transitions are not reported since below the 10^{-5} threshold.

Azulene	SA-CASSCF	CASPT2	Ref. weight	MS-CASPT2	Excitation energy			f_{Osc}	Einstein coefficient	TDM	
state	(E_h, −383)	(E_h, −384)		(E_h, −384)	Main character	(eV)	(nm)		(s^{-1})	(e Å)	(D)
S_0	−0.695 061	−1.156 753	0.68	−1.156 755	GS	—	—	—	—	—	—
S_1	−0.621 314	−1.077 262	0.68	−1.079 035	$\pi \rightarrow \pi^*(b_1)$	2.12	586	0.0036	693 050	0.1389	0.667
S_2	−0.539 929	−0.972 257	0.68	−1.009 650	$\pi \rightarrow \pi^*(a_1)$	4.00	310	0.0024	1 647 486	0.0822	0.395
S_3	−0.530 194	−1.009 652	0.67	−0.987 229	$\pi \rightarrow \pi^*(b_1)$	4.61	269	0.0088	8 164 571	0.1480	0.711
S_4	−0.526 297	−0.956 504	0.68	−0.972 332	$\pi \rightarrow \mathrm{Ryd}_{s}(a_2)$	5.02	247	—	—	0.0001	0.001
S_5	−0.524 235	−0.986 066	0.67	−0.956 504	$\pi \rightarrow \mathrm{Ryd}_{p_y}(b_2)$	5.45	228	0.0002	249 222	0.0201	0.097
S_6	−0.523 664	−0.954 636	0.68	−0.954 561	$\pi \rightarrow \mathrm{Ryd}_{p_x}(a_2)$	5.50	225	—	—	0.0003	0.002
S_7	−0.520 226	−0.950 650	0.68	−0.947 715	$\pi \rightarrow \mathrm{Ryd}_{p_z}(b_1)$	5.69	218	0.0008	1 139 945	0.0404	0.194

S9.1.2 Crystal spectrum evaluation

Since wave function orbital components are invariant to spatial rigid translation of the underlying atoms, we used the same four unique monomer MS-CASPT2 wave functions (i.e., the corresponding `JobMix` file) for all possible dimer evaluations. Out of the 500 azulene molecules contained in the $5 \times 5 \times 5$ supercell, we chose one azulene monomer as reference. We located all symmetry unique dimers within 25 Å from the reference molecule (fig. S5), since we deemed that dimers further away would not have significant coupling terms. For these dimers, we computed excitonic couplings using the input shown in listing S34.

As a practical approximation when building the crystal excitonic Hamiltonian, we considered each monomer electronic excitation separately. This is justified by considering, for example, the flat–flat dimer that, among those we computed, has the largest computed excitonic couplings, in absolute value. As shown in table S6, excitonic states 2 and 3 are mostly constituted by a in-phase (state 2) and out-of-phase (state 3) combination of monomer excitations to the first excited state ($\langle 1\,2|$ and $\langle 2\,1|$). Since the in-phase one is the lowest excitation, these two states represent a J-aggregate, and the corresponding absorbance peak should be at least slightly red-shifted with respect to the monomer one. Similarly, excitonic states 6 and 7 are constituted by $\langle 1\,3|$ and $\langle 3\,1|$ (7 is in-phase, thus they are in a H-aggregate situation, blue-shift of the absorbance peak), and 8 and 9 are constituted by $\langle 1\,4|$ and $\langle 4\,1|$ (8 is in-phase, that is J-aggregate, red-shift of the absorbance peak). Contrarily, excitonic states 4, 5, 10, 11, 12, 13, 14, and 15 present a mixture of constituting states, also for those states with a clear predominance of a single monomer state (e.g., excitonic state 12). Taking into consideration the computed oscillator strengths for the excitonic excitation reported in table S7, only excitations to the excitonic states 2, 7 and 8 are worth further investigation. Therefore, the choice of using only the corresponding underlying monomer excitations, and not including couplings between different excitations.

Therefore, we built a 500 dimensional matrix, the diagonal elements of which were the monomer $S_0 \rightarrow S_1$ excitation energy, and the off-diagonal elements were the computed Frenkel $\langle (2)B\,(1)A|v|(1)B\,(2)A\rangle$ (i.e., monomer B is excited from state 1 to state 2, at the same time as monomer A is relaxed from state 2 to state 1) couplings between the various dimers. We employed periodic boundary conditions to introduce coupling between monomers at the cell unit border. Similarly, we built matrices using the monomer $S_0 \rightarrow S_2$ excitation energy, along with $\langle (3)B\,(1)A|v|(1)B\,(3)A\rangle$ couplings, and $S_0 \rightarrow S_3$ excitation energy, along with $\langle (4)B\,(1)A|v|(1)\,B(4)A\rangle$ couplings. After diagonalization of each matrix, the eigenvalues corresponded to the excitation energies, and applying the eigenvectors to the monomer transition dipole moments we obtained the corresponding oscillator strengths, used to plot the spectrum of fig. 18a.

Section S9.3 details how we used excitation energies and oscillator strengths data to plot the simulated spectra of azulene monomer and crystal.

Table S6: Eigenvectors of the Frenkel Hamiltonian relative to the flat–flat azulene dimer. Original values were in scientific notation, but are reported as float with only two decimals for ease of comparison. Yellow highlighted values are larger than 0.5 in absolute value, green highlighted values are comprised between 0.5 and 0.1, in absolute value. An excited excitonic state that was perfectly described by a combination of two equivalent monomer excited states (that is, as if in a dipole–dipole approximation) would have values $\pm 1/\sqrt{2}$ for the combining states, and zeroes otherwise. An excitonic state that was mostly constituted by a single monomer excited state (that is, no exciton delocalization) would have values close to unity for such constituting state, and zeroes otherwise. For example, the excitonic ground state is completely constituted by the monomer ground states.

	Excitonic state														
$\langle B\,A\vert$	1	2	3	4	5	6	7	8	9	10	11	12	13	14	15
$\langle 1\,1\vert$	1.00	0.00	0.00	0.00	0.00	0.00	0.00	0.00	0.00	0.00	0.00	0.00	0.00	0.00	0.00
$\langle 1\,2\vert$	0.00	0.68	−0.73	0.03	−0.01	0.00	0.00	0.00	0.00	0.00	0.00	0.00	0.00	0.00	0.00
$\langle 1\,3\vert$	0.00	0.00	0.00	0.00	−0.01	−0.67	0.75	0.00	0.00	0.00	0.00	0.00	0.00	0.00	0.00
$\langle 1\,4\vert$	0.00	0.00	0.00	0.11	−0.02	0.00	−0.01	0.72	−0.69	0.00	−0.02	0.00	0.00	0.00	0.00
$\langle 1\,5\vert$	0.00	0.02	−0.02	−0.65	0.13	0.00	0.01	0.06	−0.06	0.01	0.72	0.12	0.01	0.14	−0.01
$\langle 1\,6\vert$	0.00	−0.02	0.02	0.63	−0.12	0.00	−0.01	−0.04	0.04	0.01	0.64	0.18	0.01	−0.38	0.01
$\langle 1\,7\vert$	0.00	0.00	0.00	−0.04	0.01	0.00	0.00	0.00	0.00	0.00	−0.21	0.98	0.05	0.04	0.00
$\langle 1\,8\vert$	0.00	−0.01	0.01	0.36	−0.07	0.00	0.00	−0.02	0.02	0.00	0.16	0.01	0.00	0.91	−0.04
$\langle 2\,1\vert$	0.00	0.73	0.68	0.01	0.03	0.00	0.00	0.00	0.00	0.00	0.00	0.00	0.00	0.00	0.00
$\langle 3\,1\vert$	0.00	0.00	0.00	0.01	−0.02	0.75	0.67	0.00	0.00	0.00	0.00	0.00	0.00	0.00	0.00
$\langle 4\,1\vert$	0.00	0.00	0.00	0.02	0.11	0.00	0.01	0.69	0.72	−0.02	0.00	0.00	0.00	0.00	0.00
$\langle 5\,1\vert$	0.00	−0.02	−0.01	0.12	0.63	0.00	0.01	−0.06	−0.07	−0.74	0.01	−0.01	0.10	−0.01	−0.14
$\langle 6\,1\vert$	0.00	−0.02	−0.02	0.12	0.63	0.00	0.01	−0.04	−0.04	0.59	−0.01	0.02	−0.31	−0.01	−0.37
$\langle 7\,1\vert$	0.00	0.00	0.00	0.03	0.14	0.00	0.00	−0.01	−0.01	0.28	0.00	−0.05	0.94	−0.01	−0.11
$\langle 8\,1\vert$	0.00	−0.01	−0.01	0.07	0.37	0.00	0.01	−0.02	−0.03	0.16	0.00	0.00	0.00	0.03	0.91

Table S7: Computed flat–flat azulene dimer spectral data, relative to only the excitations from the excitonic ground state.

from	to	exc. energy (eV)	osc. str.
1	2	2.11	0.0071
1	3	2.11	0.0000
1	4	3.69	0.0000
1	5	3.70	0.0001
1	6	4.00	0.0000
1	7	4.01	0.0047
1	8	4.62	0.0177
1	9	4.62	0.0000
1	10	5.16	0.0001
1	11	5.17	0.0002
1	12	5.36	0.0000
1	13	5.37	0.0000
1	14	5.67	0.0009
1	15	5.67	0.0006

S9.2 Z–M azobenzene derivatives heterodimer

S9.2.1 Monomer geometries and energetics

Geometries for the Z (fig. S6a) and M (fig. S6b) *trans*-azobenzene models were optimized in vacuo at the DFT level of theory using the B3LYP functional[S17–S20] and def2-TZVP basis set.[S21,S22] Both molecules presented a nearly perfectly planar geometry (fig. S6, panels c and d). Listings S32 and S33 report the optimized coordinates.

Single point energy evaluations were performed for both monomers at the four roots state-averaged RASSCF level of theory,[S8] using the double zeta ANO-RCC-VDZP basis set.[S9] The active orbitals comprised the whole conjugated π system, plus the two central nitrogen atoms' lone pairs. Monomer Z comprised 5 orbitals in RAS1 (table S8), 7 in RAS2 (table S9), and 4 in RAS3 (table S10), with 3 holes/excitations allowed in RAS1 and RAS3, and a total of 18 active electrons. Monomer M comprised 6 orbitals in RAS1 (table S11), 7 in RAS2 (table S12), and 4 in RAS3 (table S13), with 3 holes and excitations allowed in RAS1 and RAS3, and a total of 20 active electrons. We used a level shift value of $0.1\,E_\mathrm{h}$ to help wave function convergence. Subsequently, energies were re-evaluated at the MS-RASPT2 level,[S23,S24] with default IPEA shift value $0.25\,E_\mathrm{h}$,[S12] and an imaginary level shift of $0.1\,E_\mathrm{h}$.[S13] Complete energetics data for Z and M azobenzene derivatives are reported in table S14, respectively.

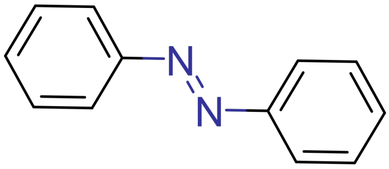

(a)

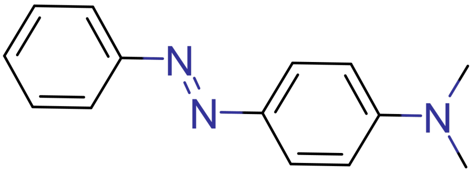

(b)

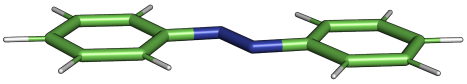

(c)

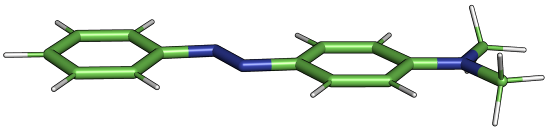

(d)

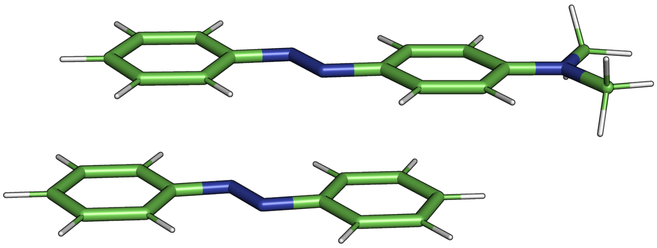

(e)

Figure S6: **Structures of *trans*-azobenzene models.** 2D chemical structures of Z (a) and M (b) compounds, while (c) and (d) are 3D representations of the corresponding final optimized geometries. ZM dimer model is shown in (e) where Z and M are in antiparallel orientation and separated by a distance of 3.5 Å.

Listing S32: Coordinates (in Å) of Z final optimized geometry. The coordinates of the centre of mass are $(5.898, -0.350, -1.271)$.

```
C      5.89790438    -0.40458092     3.25861401
C      5.89789351     0.86664170     2.69687206
C      5.89791048     1.01261834     1.31596003
C      5.89785772    -1.53073770     2.43471036
C      5.89788167    -1.39274702     1.05718685
C      5.89789107    -0.11241877     0.49031776
N      5.89789467     0.15269137    -0.90112465
N      5.89789536    -0.85211074    -1.64086575
C      5.89789381    -0.58703052    -3.03231030
C      5.89787763     0.69327808    -3.59922482
C      5.89785058     0.83121533    -4.97675526
C      5.89790632    -1.71209810    -3.85791172
C      5.89790731    -1.56617704    -5.23882809
C      5.89789921    -0.29497443    -5.80061572
H      5.89790728     1.74240821     3.33314577
H      5.89793014     1.99098590     0.85336050
H      5.89786037    -2.52002729     2.87531099
H      5.89787024    -2.25586886     0.40709428
H      5.89785977     1.55642495    -2.94916460
H      5.89787929     1.82048877    -5.41739498
H      5.89787168    -0.17780331    -6.87697021
H      5.89792363    -2.69044626    -3.39527172
H      5.89791186    -2.44196968    -5.87506823
H      5.89788201    -0.52180199     4.33496345
```

Listing S33: Coordinates (in Å) of M final optimized geometry. The coordinates of the centre of mass are $(9.1922, -0.49978, 0.03962)$.

```
C      9.16908905    -0.64135276    -4.47233796
C      9.13228658     0.51481030    -3.69264092
C      9.14583343     0.43250918    -2.31039729
C      9.21830312    -1.88723733    -3.85774088
C      9.23053558    -1.97715663    -2.47201689
C      9.19599677    -0.82181523    -1.68873581
N      9.21038293    -1.02779639    -0.28864668
N      9.21221652     0.01585924     0.40478472
C      9.21924663    -0.16350371     1.79417600
C      9.23639225     0.99853690     2.56958559
C      9.24268980     0.94596169     3.94950316
C      9.20747095    -1.40147673     2.45337661
C      9.21175945    -1.46848733     3.82881943
C      9.22959523    -0.29438819     4.62313133
H      9.09202143     1.48565937    -4.17139596
H      9.11708573     1.32115177    -1.69632107
H      9.24636804    -2.78843665    -4.45740342
H      9.26730280    -2.93624160    -1.97163698
H      9.24484717     1.95370770     2.06004376
H      9.25591933     1.87015957     4.50698743
N      9.23305729    -0.36293613     5.99375562
H      9.19370851    -2.30763180     1.86392392
H      9.20111860    -2.44045913     4.29926610
C      9.20556873    -1.64837593     6.66305485
C      9.25558534     0.85058773     6.78644498
H     10.07688706    -2.25861216     6.40823609
H      8.30929363    -2.22047592     6.40624120
H      9.20792090    -1.49295118     7.73869511
```

```
H     10.14925763      1.45069763      6.58552188
H      9.25859114      0.58767217      7.84109055
H      8.37781336      1.47756579      6.59718525
H      9.15817501     -0.56720423     -5.55251971
```

Table S8: RAS1 active space orbitals for *trans*-azobenzene Z monomer. Electronic occupation for each state is given under each orbital. Density contour value = 0.05.

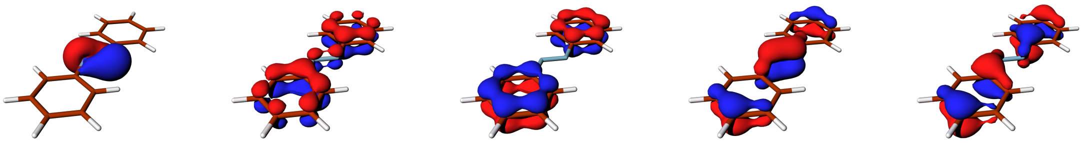

S_0	1.998	1.965	1.965	1.929	1.912
S_1	1.944	1.968	1.966	1.949	1.919
S_2	1.998	1.941	1.927	1.904	1.791
S_3	1.998	1.940	1.927	1.904	1.788

Table S9: RAS2 active space orbitals for *trans*-azobenzene Z monomer. Electronic occupation for each state is given under each orbital. Density contour value = 0.05.

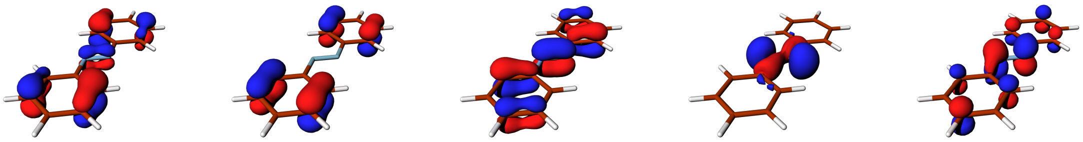

S_0	1.900	1.901	1.854	1.997	0.153
S_1	1.901	1.901	1.884	1.055	1.051
S_2	1.620	1.747	1.506	1.997	0.580
S_3	1.745	1.520	1.611	1.997	0.577

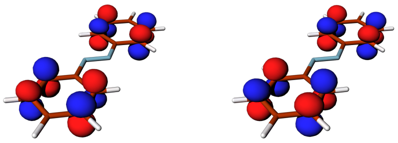

S_0	0.100	0.100
S_1	0.108	0.101
S_2	0.330	0.242
S_3	0.244	0.328

Table S10: RAS3 active space orbitals for *trans*-azobenzene Z monomer. Electronic occupation for each state is given under each orbital. Density contour value = 0.05.

	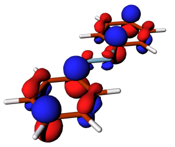	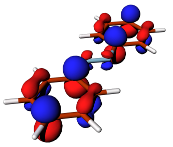	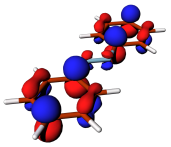	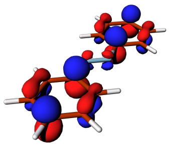
S_0	0.090	0.072	0.033	0.032
S_1	0.099	0.091	0.034	0.034
S_2	0.217	0.091	0.059	0.050
S_3	0.220	0.091	0.059	0.050

Table S11: RAS1 active space orbitals for *trans*-azobenzene M monomer. Electronic occupation for each state is given under each orbital. Density contour value = 0.05.

	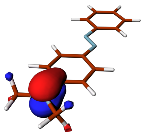	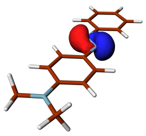	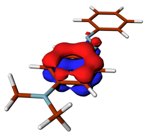	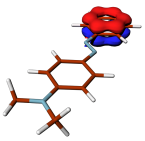	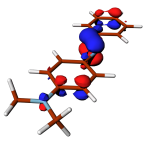
S_0	1.997	1.998	1.967	1.965	1.932
S_1	1.998	1.944	1.969	1.967	1.950
S_2	1.994	1.998	1.934	1.965	1.907
S_3	1.997	1.998	1.967	1.934	1.904

	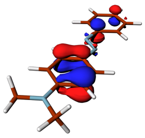
S_0	1.915
S_1	1.922
S_2	1.857
S_3	1.860

Table S12: RAS2 active space orbitals for *trans*-azobenzene M monomer. Electronic occupation for each state is given under each orbital. Density contour value = 0.05.

	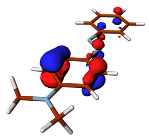	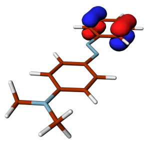	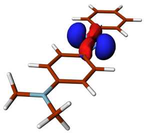	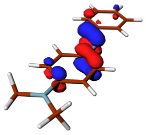	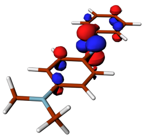
S_0	1.909	1.909	1.997	1.853	0.159
S_1	1.909	1.909	1.055	1.884	1.049
S_2	1.581	1.581	1.997	1.328	0.460
S_3	1.901	1.901	1.997	1.499	0.669

	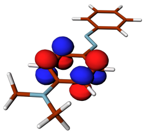	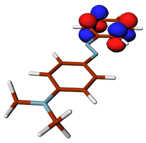
S_0	0.093	0.100
S_1	0.097	0.101
S_2	0.678	0.108
S_3	0.107	0.474

Table S13: RAS3 active space orbitals for *trans*-azobenzene M monomer. Electronic occupation for each state is given under each orbital. Density contour value = 0.05.

	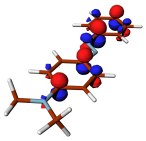	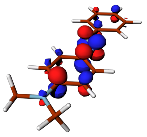	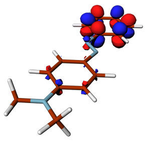	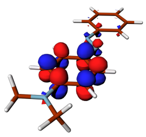
S_0	0.087	0.069	0.033	0.031
S_1	0.098	0.090	0.034	0.032
S_2	0.126	0.088	0.032	0.063
S_3	0.130	0.085	0.063	0.031

Table S14: Complete energetics data and properties for azobenzene Z and M derivatives.

Z monomer	SA-CASSCF	RASPT2	Ref. weight	MS-RASPT2	Excitation energy			f_{Osc}	Einstein coefficient	TDM	
state	(E_h, −569)	(E_h, −571)		(E_h, −571)	Main character	(eV)	(nm)		(s^{-1})	(e Å)	(D)
S_0	−0.683 515	−0.432 985	0.63	−0.432 985	GS	—	—	—	—	—	—
S_1	−0.563 434	−0.337 809	0.62	−0.337 809	$n \rightarrow \pi^*$	2.59	479	0.000 00	—	—	—
S_2	−0.506 697	−0.267 140	0.62	−0.267 140	$\pi \rightarrow \pi^*$	4.51	275	0.012 04	10 642 888	0.17	0.84
S_3	−0.506 165	−0.266 114	0.62	−0.266 113	$\pi \rightarrow \pi^{*2}$	4.54	273	0.000 00	—	—	—

M monomer	SA-CASSCF	RASPT2	Ref. weight	MS-RASPT2	Excitation energy			f_{Osc}	Einstein coefficient	TDM	
state	(E_h, −702)	(E_h, −704)		(E_h, −704)	Main character	(eV)	(nm)		(s^{-1})	(e Å)	(D)
S_0	−0.859 348	−1.089 153	0.57	−1.089 223	GS	—	—	—	—	—	—
S_1	−0.738 453	−0.991 638	0.56	−0.991 638	$n \rightarrow \pi^*$	2.66	467	0.000 05	14 488	0.01	0.07
S_2	−0.685 583	−0.931 741	0.56	−0.931 700	$\pi \rightarrow \pi^*$	4.29	289	0.035 50	28 301 885	0.31	1.48
S_3	−0.682 774	−0.923 560	0.56	−0.923 530	$\pi \rightarrow \pi^{*2}$	4.51	275	0.005 38	4 742 679	0.12	0.56

S9.2.2 Excitonic spectrum evaluation

A ZM heterodimer model (fig. S6e) was constructed as close as possible to what described in ref. S25: Z and M were parallel, with the respective center of masses at a distance of 3.5 Å. Excitonic coupling was computed using the input reported as listing S35.

Obtained Frenkel's Hamiltonian eigenvectors, and corresponding excitonic excitation energies are reported in tables S15 and S16. As opposed to the data presented in table S6, ZM excitonic states 2, 3, 4, and 5 are dominated by a single state. In particular, excitonic state 2 is dominated by the second excited state of the Z monomer, while states 3, 4, and 5 are dominated by the second, third, and fourth excited states of the M monomer, respectively. Finally, excitonic states 6 and 7 are due to a mixing of M monomer third and fourth excited states, which are rather close in energy.

The data was collated to plot the spectrum, following section S9.3, and to draw the state diagram shown in fig. S7. It is notable how all excitonic excited states are red-shifted with respect to the originating monomer states. A dipole–dipole approximation based treatment of the system would not be capable of describing this peculiar situation, since it would only couple states of different monomers to induce splitting.

Table S15: Eigenvectors of the Frenkel Hamiltonian relative to ZM heterodimer. Original values were in scientific notation, but are reported as float with only two decimals for ease of comparison. Yellow highlighted values are larger than 0.5 in absolute value, green highlighted values are comprised between 0.5 and 0.1, in absolute value. A stands for the states of the Z monomer, B stands for the states of the M monomer.

	Excitonic state						
$\langle B\,A\vert$	1	2	3	4	5	6	7
$\langle 1\,1\vert$	1.00	0.01	0.00	−0.01	0.02	0.02	0.00
$\langle 1\,2\vert$	−0.01	0.99	−0.13	0.00	0.00	0.00	0.00
$\langle 1\,3\vert$	−0.02	0.00	0.00	−0.02	−0.01	0.91	0.42
$\langle 1\,4\vert$	0.00	0.00	0.00	0.01	0.02	−0.42	0.91
$\langle 2\,1\vert$	0.00	0.13	0.99	0.00	0.00	0.00	0.00
$\langle 3\,1\vert$	0.01	0.00	0.00	1.00	0.00	0.02	0.00
$\langle 4\,1\vert$	−0.02	0.00	0.00	0.00	1.00	0.02	−0.01

Table S16: ZM dimer spectral data, relative to only the excitations from the excitonic ground state.

from	to	exc. energy (eV)	osc. str.
1	2	2.49	0.0000
1	3	2.56	0.0000
1	4	3.85	0.0313
1	5	4.01	0.0049
1	6	4.04	0.0087
1	7	4.07	0.0021

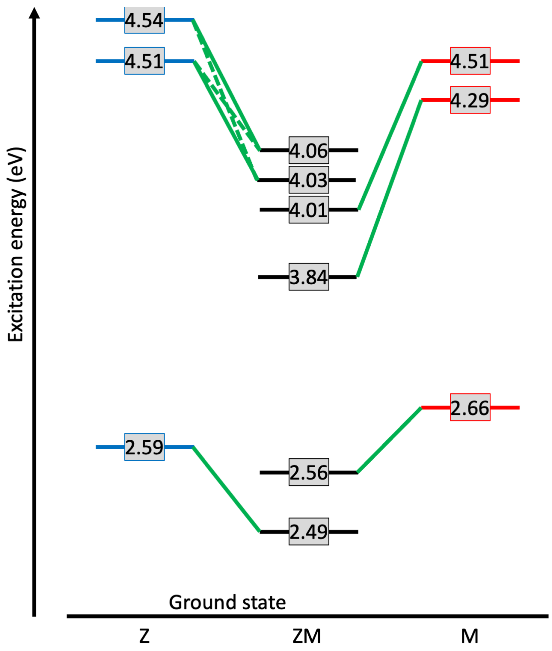

Figure S7: **State diagram for ZM heterodimer.** Z monomer excited states (blue boxes on the left) and M monomer excited states (red boxes on the right) are combined into the ZM heterodimer excitonic states (black boxes in the middle). Excitation energies are on a qualitative scale. All heterodimer excitonic excited states are red-shifted with respect to the generating monomer states.

S9.3 Plotting absorption spectra

Starting from a set of excitation energies and their corresponding oscillator strengths f, we plotted a simulated absorption spectrum as here detailed. We generated a Gaussian-like distribution for each excitation, with the line shape function defined as:

$$g(E) = \frac{f_n}{\sigma\sqrt{2\pi}} \exp\left(-\frac{(E - \Delta E_{\mathrm{S}_n - \mathrm{S}_0})^2}{2\sigma^2}\right) \tag{S3}$$

Each function is centered at the excitation value ($\Delta E_{\mathrm{S}_n - \mathrm{S}_0}$) and weighted by the corresponding f_n, with n running over all excitations of the considered molecule/system. The parameter σ is a phenomenological bandwidth, which we chose to be 0.1 eV. The final spectrum results from the sum of Gaussian-like distributions.

In fig. 18a, the azulene monomer spectrum was plotted using the data of table S5, while the one relative to the crystal used what obtained through the procedure described at the end of section S9.1.2. Both spectra were normalized such as the highest peak of the monomer spectrum corresponded to an intensity of 1.

In fig. 18b, the absorbance spectrum for Z and M monomers was plotted using the data of table S14, and for ZM heterodimer the data from table S16. All spectra were normalized such that the M monomer highest peak intensity corresponded to 1.

S9.4 Example input files

We present here the input files required to compute Frenkel's excitonic couplings for a homodimer of azulene (listing S34) and the heterodimer of Z and M *trans*-azobenzene (listing S35). In both cases, energetics calculations for each monomer have already been

performed. Further technical details on the use of the `RUNFILE`, as well as when to use the keywords ***ricd*** and ***fake ricd*** are given in ref. S26.

Listing S34: Example of input for a dimer of azulene molecules. The input implies that one has already computed the energetics for both monomers, and has available the corresponding geometries and `JobMix` files.

```
> COPY $InputDir/flat_opt_248.xyz monoA.xyz
> COPY $InputDir/tilt_opt_231.xyz  monoB.xyz

***** INTEGRALS OF A+B PART 1 ******

 &GATEWAY
BASDIR = $InputDir
coord=monoA.xyz
coord=monoB.xyz
bsse=1
Basis=
ANO-RCC-VTZP, X.AZURYDBERG
NoMove
Group=Nosym
ricd

 &SEWARD

>>> COPY $Project.RunFile  AUXRFIL1

***** SYSTEM A ******

 &GATEWAY
BASDIR = $InputDir
coord=monoA.xyz
Basis=
ANO-RCC-VTZP, X.AZURYDBERG
NoMove
Group=Nosym

 &SEWARD
fake ricd

> COPY $InputDir/flat_opt-pt2_mix.JobMix JOB001

 &RASSI
EJOB
Nr  of  Job = 1 all
TRD1
TRDI
MONA

***** INTEGRALS OF A+B PART 2 ******

 &GATEWAY
BASDIR = $InputDir
coord=monoA.xyz
coord=monoB.xyz
bsse=2
Basis=
ANO-RCC-VTZP, X.AZURYDBERG
```

```
NoMove
Group=Nosym

 &SEWARD
fake ricd

>>> COPY $Project.RunFile AUXRFIL2

***** SYSTEM B ******

 &GATEWAY
BASDIR = $InputDir
coord=monoB.xyz
Basis=
ANO-RCC-VTZP, X.AZURYDBERG
NoMove
Group=Nosym

 &SEWARD
fake ricd

> COPY $InputDir/tilt_opt-pt2_mix.JobMix JOB001

 &RASSI
EJOB
Nr  of  Job = 1 all
TRD1
TRDI
MONB
EXCItonics
```

Listing S35: Example of input for a heterodimer of Z and M *trans*-azobenzene molecules. The input implies that one has already computed the energetics for both monomers, and has available the corresponding geometries and `JobMix` files.

```
> COPY $InputDir/monoZ.xyz monoA.xyz
> COPY $InputDir/monoM.xyz monoB.xyz

***** INTEGRALS OF A+B PART 1 ******

 &GATEWAY
coord=monoA.xyz
coord=monoB.xyz
bsse=1
Basis=ANO-RCC-VDZP
NoMove
Group=Nosym
ricd

 &SEWARD

>>> COPY $Project.RunFile  AUXRFIL1

***** SYSTEM A ******

 &GATEWAY
coord=monoA.xyz
Basis=ANO-RCC-VDZP
```

```
NoMove
Group=Nosym

 &SEWARD
fake ricd

> COPY $InputDir/CPL.monoZ.JobMix JOB001

 &RASSI
EJOB
Nr  of  Job = 1 all
TRD1
TRDI
MONA

***** INTEGRALS OF A+B PART 2 ******

 &GATEWAY
coord=monoA.xyz
coord=monoB.xyz
bsse=2
Basis=ANO-RCC-VDZP
NoMove
Group=Nosym

 &SEWARD
fake ricd

>>> COPY $Project.RunFile AUXRFIL2

***** SYSTEM B ******

 &GATEWAY
coord=monoB.xyz
Basis=ANO-RCC-VDZP
NoMove
Group=Nosym

 &SEWARD
fake ricd

> COPY $InputDir/CPL.MonoM.JobMix JOB001

 &RASSI
EJOB
Nr  of  Job = 1 all
TRD1
TRDI
MONB
EXCItonics
```

S10 Ionization and Autoionization Processes

This section reports examples of calculations of single-site double-core-hole (ssDCH) and Auger decay spectra. The implementation details can be found in refs. S27, S28, respec-

tively.

S10.1 ssDCH

For the calculation of double-core-hole spectral intensities, target double-core-hole state wave functions are computed using the ***DEXS*** keyword on the `RASSCF` input. Below we give an input example for the calculation of the ssDCH spectrum of CO at the carbon K^{-2} edge for 30 final ssDCH states. Notice that our approach is invoked with the keyword ***DCHS*** in the `RASSI` input section, followed by an integer that specifies the orbital number of the double core hole (normally this should be equal to 1, that is, the first active orbital).

Listing S36: Input example of a ssDCH spectrum calculation.

```
 &GATEWAY
 expert
 SYMMetry
 xy y
 Basis set
 O.AUG-CC-PVQZ
 Spherical all
 O                  0.0000000000        0.0000000000       -1.0658055343 / Bohr
 End of basis

 Basis set
 C.AUG-CC-PVQZ..
 Spherical all
 C                  0.0000000000        0.0000000000        1.0658055343 / Bohr
 End of Basis
 Verbose
 BSShow

&SEWARD
 Cholesky

&SCF

&RASSCF &END
 EXPERT
 LUMORB
 Symmetry = 1
 Spin = 1
 nActEl = 10 2 0
 ALTER
 1
 1 2 3
 SUPSym
 1
 1 2
 0; 0; 0
 Inactive = 2 0 0 0
 Ras1 = 1 0 0 0
 Ras2 = 4 3 0 3
 Ras3 = 0 0 0 0
 LEVShft = 0.7
 CIROot = 1 1 1
 Iter=200 100
End of input
```

```
>> COPY $Project.JobIph $Project.JobIph_GS

>> COPY $Project.RasOrb INPORB
&RASSCF &END
 EXPERT
 LUMORB
 Symmetry = 1
 Spin = 1
 nActEl = 8 2 0
 SUPSym
 1
 1 3
 0; 0; 0
 DEXS
 1
 1
 Inactive = 2 0 0 0
 Ras1 = 1 0 0 0
 Ras2 = 4 3 0 3
 Ras3 = 0 0 0 0
 LEVShft = 1.2
 OrbListing = All
 OrbAppear = Full
 Ciroot = 30 30 1
>> COPY $Project.JobIph $Project.JobIph_A1x

>> COPY $Project.JobIph_GS JOBIPH
&CASPT2 &END
MAXITER
60
MULTistate = all
IMAG
0.25
End of input
>> COPY $Project.JobMix JOB001

>> COPY $Project.JobIph_A1x JOBIPH
&CASPT2 &END
MAXITER
60
MULTistate = all
IMAG
0.25
End of input
>> COPY $Project.JobMix JOB002

&RASSI
Nrof JobIphs = 2 all
Ejob
DCHS = 1
End of input
```

The output of the RASSI section shows the binding energies and spectral intensities, which are used to plot the ssDCH spectrum of fig. S8.

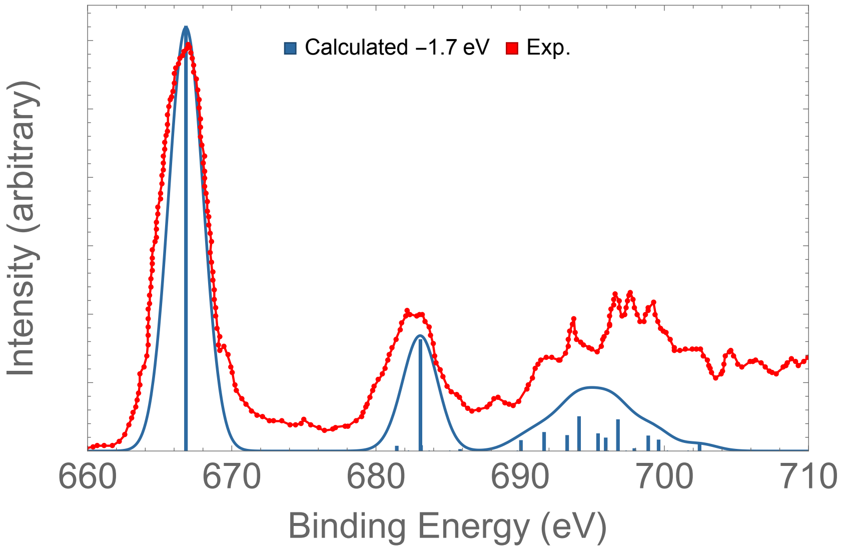

Figure S8: ssDCH stick spectrum of CO at the carbon K^{-2} edge, convoluted with Gaussian functions using half-width-at-half-maximum (hwhm) of 0.45 eV. The binding energies have been shifted by −1.3 eV to better compare to the experiment. The experimental points were extracted from ref. S29.

S10.2 Auger Decay Spectra

Auger spectra are obtained within the one-center approximation[S28] (OCA), which is currently only available for K-edge calculations of second and third row elements (C, N, O, F, Ne, Na, Mg, Al, Si, P, S, Cl, Ar). Auger matrix elements are computed with the ***TDYS*** keyword in the `RASSI` input section, followed by an integer (n) specifying the number of scattering centers. The next n lines contain strings with the scattering center type. An example for the computation of Auger matrix elements for the carbon K-edge is: ***TDYS= 1; C 1s***. Notice that ***TDYS*** requires the ***DYSON*** keyword and support to HDF5 files.

The next step is to run the `AugerOca` program, which can be found in the `Tools` folder. `AugerOca` requires Python 3 and support to `numpy` and `h5py` packages. The `AugerOca` Python 3 package (as well as `numpy` or `h5py`) can be installed via `pip`.

```
$pip install auger-oca
```

After installing the required packages, the python script `auger_main.py` can be copied and executed from any directory. For basic instructions and options, see the help menu.

Listing S37: Help menu of `auger_main.py`.

```
$python3 auger_main.py -h

usage: auger_main.py [-h] [-i INP] [-d DIRECTORY] [--oca] [--spec] [--raes] [--aes] [--t]
↪ [--s]

This program will process the 2p-Dyson density files (r2TM_) and provide:
Auger decay rates via one-center approach (OCA).
[Remember to make sure you have the $Project.rassi.h5 file in your directory.]

 usage example (RAES):

 $python3 $Tools/AugerOca/auger_main.py --raes -i r2TM_K2V_002_001

 usage example (AES for a triplet final state):
```

```

 $python3 $Tools/AugerOca/auger_main.py --aes --t -i r2TM_K2V_002_001

 (recommended) usage based on a directory with a collection of r2TM_ files.
 This directory can be the scratch directory for the RASSI calculation.

 $python3 $MOLCAS/Tools/AugerOca/auger_main.py --raes --spec -d $WorkDir

options:
  -h, --help            show this help message and exit
  -i INP, --input INP   path of the density file (r2TM_)
  -d DIRECTORY, --directory DIRECTORY
                        path of a directory with a number of density files (r2TM_)
  --oca                 Perform Auger OCA (default: True)
  --spec                save auger.spectrum.out (default: False)
  --raes                Consider resonant Auger [RAES] (default: True if OCA)
  --aes                 Consider non-resonant Auger [AES] (default: False)
  --t                   For AES, consider triplet final states (default: False)
  --s                   For AES, consider singlet final states (default: False; True if AES)
```

Below we give one input example to compute the resonant Auger spectrum (RAES) of the N_2 molecule. Here, the `AugerOca` post processing step is incorporated in the input of the calculation with a *>>SHELL* command.

For practical reasons, for molecules with equivalent scattering centers we recommend to localize the core orbitals, preferably using a Cholesky localization procedure (maintaining symmetry), or any other localization procedure such as Boys or Pipek–Mezey (lifting symmetry point group). See ref. S28 for a detailed description of the RAES spectrum calculation of N_2.

Listing S38: Input example of a resonant Auger spectrum calculation. Notice that the input file shown is incomplete. It must be completed following the ** REPEAT* instructions.

```
*--------------------------------------------------------------------------------
* Molecule: N2
* Basis: AUG-CC-PVQZ
* Symmetry: C2v with localized core
* Features tested: N2 resonant Auger spectrum using auger_oca package
* Comments: auger_oca package, if not available, can be installed with your prompt:
* pip install auger_oca
* N 1s orbitals are localized with the Cholesky method
*--------------------------------------------------------------------------------

 &GATEWAY
 expert
 SYMMetry
 xy y
 Basis set
 N.aug-cc-pvqz..
 Spherical all
 n1                0.0000000000     0.0000000000    0.548800 / Angstrom
 End of basis

 Basis set
 N.aug-cc-pvqz..
 Spherical all
 n2                0.0000000000     0.0000000000   -0.548800 / Angstrom
 End of basis
 Verbose
 BSShow

```

```
 &SEWARD &END
 Cholesky
 End of input

 &SCF &END
 End of input

 &LOCALISATION
 NORBitals
 2 0 0 0
 Cholesky

&RASSCF
 EXPERT
 LUMORB
 Symmetry
 1
 Spin
 1
 nActEl
 14 1 0
 SUPSym
 2
 1 2
 1 1
 0
 0
 0
 Inactive
 0 0 0 0
 Ras1
 2 0 0 0
 Ras2
 4 2 0 2
 Ras3
 0 0 0 0
 CIRO
 1 1 1
 Iter=200 100
 End of input
>>> COPY $Project.JobIph $Project.JobIph_gs
>>> COPY $Project.RasOrb $Project.RasOrb_gs

>> COPY $Project.RasOrb_gs INPORB
 &RASSCF &END
 EXPERT
 LUMORB
 Symmetry
 4
 Spin
 1
 nActEl
 14 1 0
 SUPSym
 2
  1 1
  1 2
 0
```

```
 0
 0
 HEXS
 1
 1
 Inactive
 0 0 0 0
 Ras1
 2 0 0 0
 Ras2
 4 2 0 2
 Ras3
 0 0 0 0
 CIRO
 6 6 1
 LEVShft
 0.8
 Iter=200 100
 End of input
>> COPY $Project.JobIph $Project.JobIph_CORE

>> COPY $Project.RasOrb_gs INPORB
 &RASSCF &END
 EXPERT
 LUMORB
 Symmetry
 1
 Spin
 2
 nActEl
 13 1 0
 SUPSym
 2
  1 1
  1 2
 0
 0
 0
 Inactive
 0 0 0 0
 Ras1
 2 0 0 0
 Ras2
 4 2 0 2
 Ras3
 0 0 0 0
 CIRO
 30 30 1
 Iter=200 100
 End of input
>> COPY $Project.JobIph $Project.JobIph_A1

>> COPY $Project.RasOrb_gs INPORB
 &RASSCF &END
 EXPERT
 LUMORB
 Symmetry
 2
```

```
 Spin
 2
 nActEl
 13 1 0
 SUPSym
 2
  1 1
  1 2
 0
 0
 0
 Inactive
 0 0 0 0
 Ras1
 2 0 0 0
 Ras2
 4 2 0 2
 Ras3
 0 0 0 0
 CIRO
 30 30 1
 Iter=200 100
 End of input
>> COPY $Project.JobIph $Project.JobIph_B1

>> COPY $Project.RasOrb_gs INPORB
 &RASSCF &END
 EXPERT
 LUMORB
 Symmetry
 4
 Spin
 2
 nActEl
 13 1 0
 SUPSym
 2
  1 1
  1 2
 0
 0
 0
 Inactive
 0 0 0 0
 Ras1
 2 0 0 0
 Ras2
 4 2 0 2
 Ras3
 0 0 0 0
 CIRO
 30 30 1
 Iter=200 100
 End of input
>> COPY $Project.JobIph $Project.JobIph_B2

>> COPY $Project.RasOrb_gs INPORB
 &RASSCF &END
```

```
 EXPERT
 LUMORB
 Symmetry
 3
 Spin
 2
 nActEl
 13 1 0
 SUPSym
 2
  1 1
  1 2
 0
 0
 0
 Inactive
 0 0 0 0
 Ras1
 2 0 0 0
 Ras2
 4 2 0 2
 Ras3
 0 0 0 0
 CIRO
 30 30 1
 Iter=200 100
 End of input
>> COPY $Project.JobIph $Project.JobIph_A2


>> COPY $Project.JobIph_CORE JOBIPH
&CASPT2 &END
MAXITER
160
MULTistate = 1 1
IMAG
0.25
End of input
>> COPY $Project.JobMix $Project.JobMix_CORE
>> COPY $Project.JobMix_CORE JOB001

>> COPY $Project.JobIph_A1 JOBIPH
&CASPT2 &END
MAXITER
160
MULTistate = all
IMAG
0.25
End of input
>> COPY $Project.JobMix $Project.JobMix_A1x
>> COPY $Project.JobMix_A1x JOB002

>> COPY $Project.JobIph_B1 JOBIPH
&CASPT2 &END
MAXITER
160
MULTistate = all
IMAG
```

```
0.25
End of input
>> COPY $Project.JobMix $Project.JobMix_B1x
>> COPY $Project.JobMix_B1x JOB003

>> COPY $Project.JobIph_B2 JOBIPH
&CASPT2 &END
MAXITER
160
MULTistate = all
IMAG
0.25
End of input
>> COPY $Project.JobMix $Project.JobMix_B2x
>> COPY $Project.JobMix_B2x JOB004

>> COPY $Project.JobIph_A2 JOBIPH
&CASPT2 &END
MAXITER
160
MULTistate = all
IMAG
0.25
End of input
>> COPY $Project.JobMix $Project.JobMix_A2x
>> COPY $Project.JobMix_A2x JOB005

&RASSI
 Nrof JobIphs =  5 all
 Ejob
 TDYS
 2
 n1 1s
 n2 1s
 DYSON
 End of input

>> SHELL $MOLCAS/Tools/AugerOca/auger_main.py -d $WorkDir --raes --spec
>> COPY auger.spectrum.out $CurrDir/$Project.auger.spectrum.out
```

After the `Happy Landing`, a file `auger.spectrum.out` containing the Auger spectrum is created. The data is used to plot the spectrum in fig. S9.

Listing S39: First five lines of the `auger.spectrum.out` file.

```
 385.144525272 4.703085550629902e-05 4.806396806165732e-05
 382.040239436 1.4554513122060067e-06 1.4858744308002272e-06
 375.405751766 5.773528972561873e-05 5.9010026834452716e-05
 374.625644331 3.840425866689331e-05 3.924869159969684e-05
 374.402025037 6.288330829760877e-06 6.42586976974955e-06
 ...
```

Notice that the first column of the `auger.spectrum.out` file gives the kinetic energy, followed by the Auger intensity on each nitrogen atom. As the nitrogen atoms are equivalent in this example, the intensities on the same row are expected to be similar.

We recommend using the built-in basis sets available within the `OpenMolcas` package, especially the Dunning and ANO families of basis sets. Using tailor-made basis sets or basis sets imported from external repositories, such as `Basis Set Exchange` (BSE), is

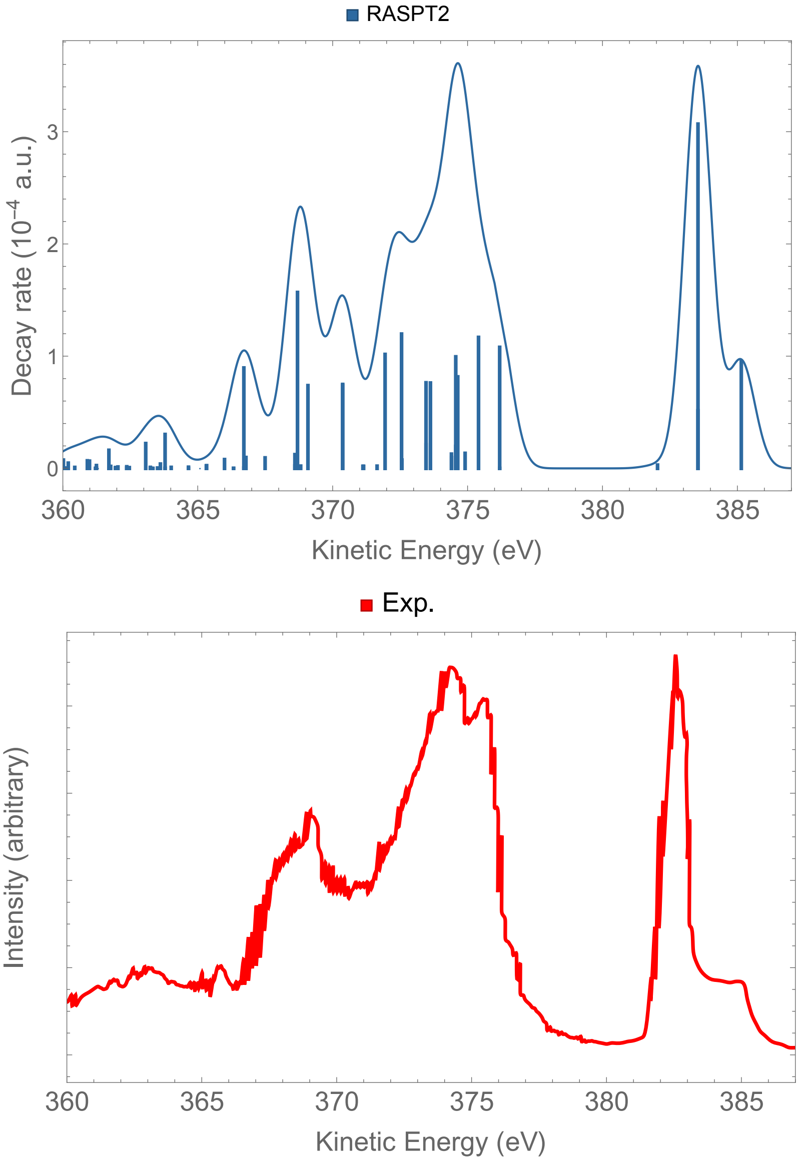

Figure S9: RAES spectrum of N_2 convoluted with Gaussian functions using hwhm of 0.5 eV. The experimental points were extracted from ref. S30.

possible but at your own risk.

Additional input examples for normal Auger of water (singlet and triplet channels), RAES of N_2 without localization step, and RAES of CO at the carbon edge are found in the files `AES_H2O_singlet.input`, `AES_H2O_triplet.input`, `RAES_N2_c2v.input`, and `RAES_CO-C_edge.input` in the `SI_files` folder called `Auger`.

Examples of inputs for the CO and the H_2O molecules, and their respective outputs, can also be found in the folder `Tools/AugerOca/auger_examples/`.

S11 SA-SI-REKS Quantum–classical Trajectories

In this section additional information is provided with respect to the `OpenMolcas`–`GAMESS-US/Tinker` interface. In particular, the capabilities of the interface, the technical description of the interface, the description of rotary cycle in molecular motors, and results from MTDP simulations in solvent are described in detail.

S11.1 Capabilities of the Interface

Here is reported the construction and application of a novel interface created to link `OpenMolcas` and the `GAMESS-US/Tinker` software packages. This interface enables SSR based simulations at the QM/MM level. More specifically, the interface exploits the availability of the SSR energy and analytical gradient in the latest version of `GAMESS-US`. Thus, by combining the molecular dynamics and surface hopping (`Dynamix` and `Surfacehop` modules, respectively) functionality of `OpenMolcas` with QM/MM energy and gradient calculations from `GAMESS-US/Tinker`, one enables the NAMD simulations using SSR level for the quantum mechanical treatment of photoresponsive molecular systems. The interface and the examples of calculations can be downloaded from GitLab repository.[S31]

The use of ensemble mixtures of electronic configurations leads to the occurrence of fractional occupations of frontier (molecular) orbitals, which are variationally optimized together with the molecular orbital coefficients. In the current version of `GAMESS-US`, the SSR method is implemented for systems with two fractionally occupied orbitals containing in total two active electrons. This is, in principle, sufficient to describe the ground and the first open-shell singlet excited states implicated in the π-bond torsion. The S_0 and S_1 states of such a system are represented in SSR formalism in terms of different electronic configurations. More specifically, the perfectly spin-paired singlet (PPS) and the open-shell singlet (OSS) configurations are included. In the SA-REKS(2,2) method, a weighted sum (an ensemble) of these configurations is self-consistently optimized. The energies of the individual states are then computed using the variationally optimized orbitals and fractional occupation numbers. The PPS energy is assumed to approximate the energy of the ground electronic state and the OSS energy approximates the excited electronic state energy. The SSR(2,2) method improves the description of the ground and excited states from SA-REKS(2,2) by including (possible) interaction between the PPS and OSS configuration. An extension of the SSR(2,2) method, the SSR(3,2) method is also available in GAMESS-US, where the configuration space is augmented by the doubly excited singlet (DES) configuration. For the SSR(2,2) and SSR(3,2) methods, `GAMESS-US` also provides the analytic energy gradient. In conclusion, the SSR(2,2) and the SSR(3,2) offer a performance that, potentially, parallel those of XMS-CASPT2/CASSCF(2,2) calculations of singlet states but provides the energies of only 2 electronic states and therefore

2 potential energy surfaces (PES). The necessary support of electrostatic potential fitted (ESPF) QM/MM calculations of the ESPF method has been already distributed within the `OpenMolcas/Tinker` interface and systematically used for the construction of QM/MM models.[S32,S33] Recently an improved version of ESPF[S34] was implemented in `GAMESS-US/Tinker` and is introduced in a local `GAMESS-US` build, while being anticipated to be included in the future official `GAMESS-US` releases. At the moment a patch enabling this non-standard version of `GAMESS-US` is available through the interface GitLab repository.

S11.2 A Technical Description of the Interface

Below is a brief description of the interface. The `OpenMolcas` modules are used as the main driver for NAMD, while the `GAMESS-US/Tinker` is employed to calculate the SSR energies, CI coefficients and gradients. The handling of temporal files and parsing throughout the entire interface is implemented in shell scripts activated by the extended `MOLCAS` input language (EMIL) commands. The EMIL commands are specified in the `[Open]Molcas` input file. Each cycle of NAMD (equivalent to one time-step of MD) within the interface occurs in four stages (see the graphic representation in fig. S10):

Stage 1. Each NAMD cycle (i.e., the cycle connecting the current geometry and velocities to the next geometry and velocities) starts with `OpenMolcas` initializing shell scripts that update the electronic state driving the propagation, the geometry of the flexible part of the model at the current time-step (N), and the molecular orbitals produced in the previous time-step ($N-1$) in `GAMESS/Tinker` input file that is used as a guess for the calculation of REKS energy and gradient at time-step N. The time difference between $N-1$ and N is typically 1 fs.

Stage 2. `GAMESS-US/Tinker` calculations with REKS-based QM/MM method are performed, which produces the potential energies of each electronic state in the geometry corresponding to time-step N, respective CI coefficients, energy gradient of the electronic state necessary for driving the propagation, and molecular orbitals. The shell scripts parse these data to the temporal files within the scratch folder.

Stage 3. The surface hopping module, `Surfacehop`, reads from the scratch folder the data necessary for Tully's fewest switches surface hop algorithm, namely the electronic state driving the propagation, potential energies of each electronic state, and CI coefficients as produced by `GAMESS-US/Tinker`. After the execution of Tully's fewest switches surface hopping algorithm, the `Surfacehop` module issues the decision on whether NAMD continues on the same PES or whether the system undergoes a hop between the two PESs, which leads to an update of the electronic state selected for NAMD propagation. If the hop is allowed, the EMIL command initiates another `GAMESS-US/Tinker` calculation to obtain the energy and gradient for the new NAMD state.

Stage 4. Molecular dynamics module, `Dynamix`, reads from the scratch folder the geometry of time-step N, the potential energies of each electronic state, and the energy gradient of the electronic state driving the propagation. Based on the REKS forces the Verlet algorithm generates the geometry of the time-step $N+1$.

Detailed Description of the Interface Workflow. The initiation of the interface between `OpenMolcas` and `GAMESS-US/Tinker` requires: the `OpenMolcas` input file (`$Project.input`) specifying input parameters for Dynamix and Surfacehop modules of `OpenMolcas`; `RunFile` containing the information about the number of atoms and

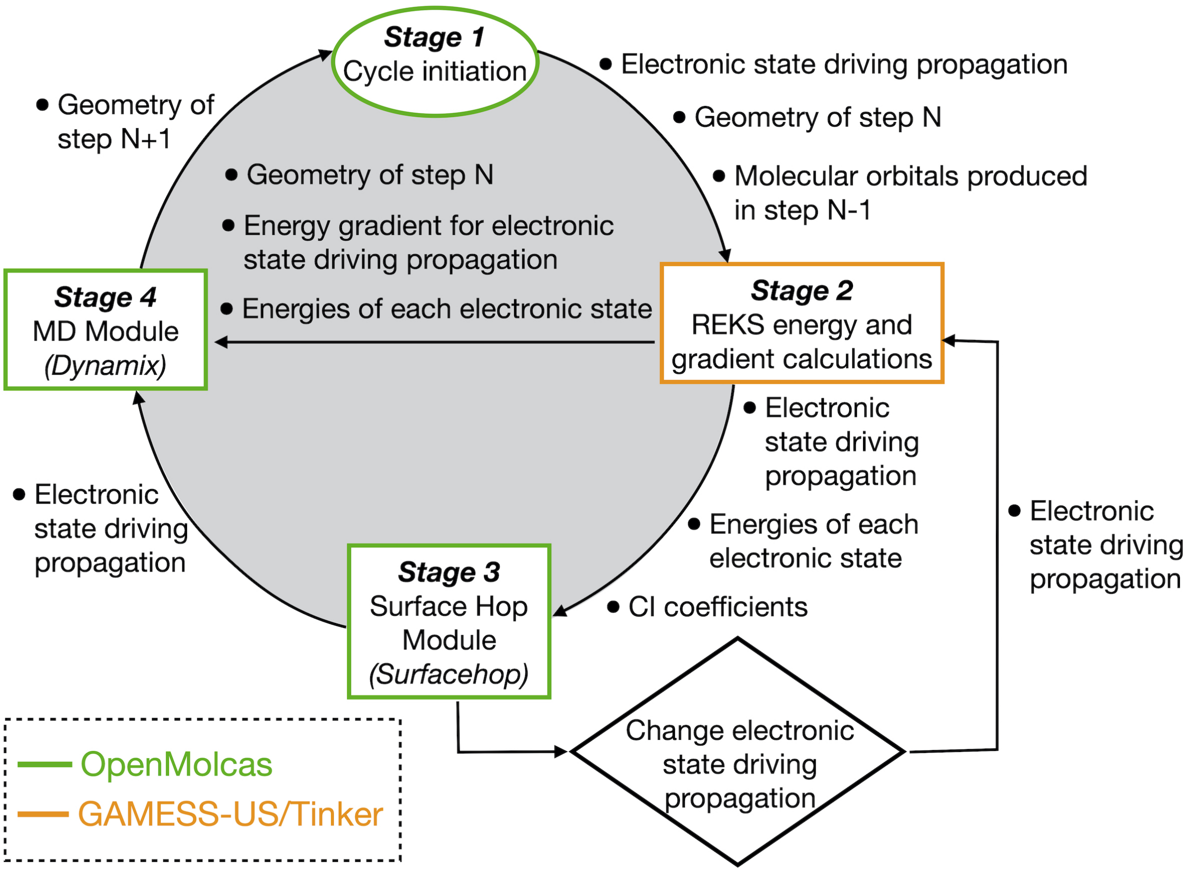

Figure S10: Schematic representation of the workflow in the `OpenMolcas`–`GAMESS-US`/`Tinker` interface.

atom types in the flexible part of the system; initial velocities file; JobIph file that allows for allocation of memory for CIBigArray of OpenMolcas; the input file specifying number of electronic states and CI coefficients ($Project.in2); as well as the GAMESS-US input file ($Project.G.inp) containing the geometry of the entire model and parameters relevant to QM calculations with SSR(2,2) and MM calculations with Tinker. The handling of temporal files and parsing throughout the entire interface is done with shell scripts that are activated with EMIL commands. The EMIL commands are specified in the OpenMolcas input file. All shell scripts are stored in the folder named GUTS. During the first cycle (first time step) in *Stage 1* the interface saves the number of electronic states, number of CI coefficients, the root number of current electronic state of NAMD propagation, and the geometry of the flexible part of the molecule in the scratch folder (OpenMolcas WorkDir). This task is performed by shell scripts slice_in2.sh and slice_gamess.sh, which extract this information from $Project.in2 and $Project.G.inp files. In each subsequent cycle (each time step) shell scripts update_gamess_geo.sh and update_gamess_vec.sh insert current geometry of the flexible part of the molecule, the root number of current electronic state of NAMD propagation, and the molecular orbitals produced during previous cycle (which are used as the initial guess in current cycle) into $Project.G.tmp.inp file corresponding to the current time step.

In *Stage 2* the EMIL command shell rungms-dev initiates the GAMESS-US/Tinker calculations with SSR(2.2)-based QM/MM method, where GAMESS-US/Tinker produces potential energies corresponding to each electronic state, CI coefficients, energy gradient of current NAMD state, and molecular orbitals. To allow for parsing of produced data with OpenMolcas modules the EMIL commands execute the shell scripts that parse the corresponding data of GAMESS-US/Tinker output file to the temporal files within scratch folder, which is done in every cycle. These shell scripts are get_gradient.sh, get_pot_energy.sh, get_S0_and_S1_energy.sh, and get_S0_and_S1_coeff.sh. The temporal files corresponding to this data are GRADIENT.txt, POT_ENERGY.txt, ROOTS_E.txt, and ROOTS_C.txt.

In *Stage 3* the Surfacehop module of OpenMolcas reads from the scratch folder the data necessary for Tully fewest switches hop algorithm, namely the current state of NAMD, potential energies, and CI coefficients produced by GAMESS-US/Tinker. This is enabled through modifications of OpenMolcas source code, where Surfacehop module is instructed to read data from temporal files created with the shell scripts. After execution of Tully fewest switches surface hopping algorithm, the Surfacehop module issues the decision on whether the NAMD continues on the same PES or whether the system undergoes the surface hopping between two PES with subsequent update of NAMD state in the scratch folder. If surface hop is detected, the EMIL command initiates the GAMESS-US/Tinker calculations to obtain the energy gradient of new NAMD state. To achieve that we made a modification to a source code of Surfacehop module which upon surface hop creates temporal file HOP.txt and prints new root into the temporal file RLXROOT.txt. Using IF (-FILE HOP.txt) statement in OpenMolcas input and slice_gamess_hop.sh shell script, we update the NAMD state in $Project.G.inp file.

In final stage *Stage 4* the Dynamix module of OpenMolcas reads from the scratch folder the current geometry, the potential energies of each electronic state, and the energy gradient of current NAMD state, which were produced by GAMESS-US/Tinker. This is achieved with modifications to OpenMolcas code. Based on the forces calculated with SSR(2,2) the Verlet algorithm propagates the geometry of the system to the next time step and creates

temporal file `GEOMETRY.txt` in scratch folder, while shell script `update_gamess_geo.sh` writes this new geometry into `Project.G.tmp.inp`. Upon completion of *Stage 4* the cycle repeats for the next time step.

S11.3 Description of Rotary Cycle in Molecular Motors

According to classic mechanism, absorption of the first photon results in breaking of the π-bond of the axle, which releases the strain energy and activates a counterclockwise (CCW) torsional motion of the rotor with respect to the stator. At nearly orthogonal configurations, the decay to the ground electronic state (S_0) then occurs in the vicinity of a S_1/S_0 conical intersection (CoIn) leading to restoration of the π-bond and relaxation to a stable intermediate with a Z configuration and an opposite M (minus) helicity (ZM). Then, the ZM intermediate is thermally converted to the final ZP product via a THI step making the system ready for a subsequent light-triggered Z to E half-rotation. After absorption of the second photon, a similar two-step CCW mechanism is activated, ultimately leading to the formation of the EM intermediate and reconstitution of the original EP reactant and completing the cycle.

S11.4 Details of MTDP Simulations in Solvent

The QM/MM model and corresponding initial conditions were prepared using the methodology that was proven to be efficient in the simulations of molecular switches/motors and chromophores in protein and was discussed in detail in previous publications.[S35–S40] Initially the trajectories were executed for 850 fs at the SSR(2,2) level of theory. In four cases the propagation time was increased to 1100 fs since the hopping only occurred at times >790 fs. After the hop from S_1 to S_0 each trajectory was propagated for an additional 200 fs using the same level of theory. At this point, the subset of "reactive" trajectories (i.e., the trajectories leading to the ZM photoproduct as opposed to the ones leading back to EP) were further propagated on the S_0 PES using the faster KS DFT with BHHLYP functional allowing to describe the ZM to ZP transition occurring over picosecond time scale. The use of a less computationally expensive KS DFT method instead of SSR justified by the fact that, for the single-reference systems, both SSR and KS DFT yield essentially identical energies and gradients.[S41,S42] After the ZP was reached through a change in helicity, the trajectory was continued for 1 ps to ensure ZP stability. The total simulated times resulting from these calculations ranged from 1850 fs to 13 500 fs. Out of 40 simulated trajectories, 32 underwent surface hopping with the average S_1 life-time of 482 fs. Of 32 trajectories that underwent surface hopping only 8 were reactive with surface hopping occurring at 396, 829, 799, 578, 343, 358, 385, and 370 fs, respectively. Six productive trajectories displayed a ZM to ZP transition that occurred at 1469, 4858, 648, 3249, 883 and 1324 fs, respectively. Two out of eight trajectories did not undergo ZM to ZP transition after the 13.5 ps of simulation.

S12 Two-Step Acceleration of the Analytic Evaluation of the Vibronic Couplings for Multi-Root Systems

Input examples of the original and two-step procedure for the analytic evaluation of the vibronic couplings for multi-root systems are given in listings S40 and S41

Listing S40: `OpenMolcas` input tailored towards the original method.

```
&SEWARD
Title
Dy_bbpenCl_2020
ANGM
8.22323771761526982802 .23989506234286763540 8.02784003632896520089
AMFI

DOAN
RICD
SDIP

CDTH
1.0d-6

VERBOSE

Basis Set
DY.ANO-RCC-mb
DY1 4.351550 0.126947 4.248150 Angstrom
End of Basis Set

Basis Set
CL.ANO-RCC-mb
CL2 4.351550 -2.554927 4.248150 Angstrom
End of Basis Set

Basis Set
O.ANO-RCC-mb
O3 5.196621 0.609911 2.312693 Angstrom
End of Basis Set

Basis Set
N.ANO-RCC-mb
N4 5.763193 2.248941 4.695055 Angstrom
End of Basis Set

Basis Set
N.ANO-RCC-mb
N5 6.569970 -0.319559 5.486911 Angstrom
End of Basis Set

Basis Set
C.ANO-RCC-mb
C6 7.206167 -1.492994 5.580370 Angstrom
End of Basis Set

Basis Set
```

```
H.ANO-RCC-mb
H7 6.805824 -2.252377 5.174247 Angstrom
End of Basis Set

Basis Set
C.ANO-RCC-mb
C8 8.409806 -1.666518 6.234585 Angstrom
End of Basis Set

Basis Set
H.ANO-RCC-mb
H9 8.841479 -2.513522 6.246480 Angstrom
End of Basis Set

Basis Set
C.ANO-RCC-mb
C10 8.970285 -0.570396 6.870108 Angstrom
End of Basis Set

Basis Set
H.ANO-RCC-mb
H11 9.786636 -0.652863 7.349299 Angstrom
End of Basis Set

Basis Set
C.ANO-RCC-mb
C12 8.323645 0.625374 6.793641 Angstrom
End of Basis Set

Basis Set
H.ANO-RCC-mb
H13 8.702230 1.391629 7.211659 Angstrom
End of Basis Set

Basis Set
C.ANO-RCC-mb
C14 7.115655 0.742202 6.113937 Angstrom
End of Basis Set

Basis Set
C.ANO-RCC-mb
C15 6.391557 2.058236 6.052764 Angstrom
End of Basis Set

Basis Set
H.ANO-RCC-mb
H16 7.027753 2.795284 6.229487 Angstrom
End of Basis Set

Basis Set
H.ANO-RCC-mb
H17 5.689216 2.082289 6.749461 Angstrom
End of Basis Set

Basis Set
C.ANO-RCC-mb
C18 4.923344 3.480790 4.768124 Angstrom
End of Basis Set
```

```
Basis Set
H.ANO-RCC-mb
H19 5.497748 4.274533 4.618589 Angstrom
End of Basis Set

Basis Set
H.ANO-RCC-mb
H20 4.535185 3.554666 5.677228 Angstrom
End of Basis Set

Basis Set
C.ANO-RCC-mb
C21 6.871968 2.401848 3.719680 Angstrom
End of Basis Set

Basis Set
H.ANO-RCC-mb
H22 7.432447 1.585769 3.758763 Angstrom
End of Basis Set

Basis Set
H.ANO-RCC-mb
H23 7.436799 3.161230 4.008554 Angstrom
End of Basis Set

Basis Set
C.ANO-RCC-mb
C24 6.471625 2.628632 2.295700 Angstrom
End of Basis Set

Basis Set
C.ANO-RCC-mb
C25 6.951166 3.716164 1.559921 Angstrom
End of Basis Set

Basis Set
H.ANO-RCC-mb
H26 7.490758 4.369027 1.993232 Angstrom
End of Basis Set

Basis Set
C.ANO-RCC-mb
C27 6.659612 3.862199 0.224302 Angstrom
End of Basis Set

Basis Set
H.ANO-RCC-mb
H28 7.007736 4.600965 -0.259987 Angstrom
End of Basis Set

Basis Set
C.ANO-RCC-mb
C29 5.865889 2.941319 -0.407822 Angstrom
End of Basis Set

Basis Set
H.ANO-RCC-mb
```

```
H30 5.649182 3.056429 -1.325423 Angstrom
End of Basis Set

Basis Set
C.ANO-RCC-mb
C31 5.375905 1.843478 0.282077 Angstrom
End of Basis Set

Basis Set
H.ANO-RCC-mb
H32 4.835442 1.209514 -0.175024 Angstrom
End of Basis Set

Basis Set
C.ANO-RCC-mb
C33 5.662237 1.651056 1.627891 Angstrom
End of Basis Set

Basis Set
O.ANO-RCC-mb
O34 3.506479 0.609911 6.183607 Angstrom
End of Basis Set

Basis Set
N.ANO-RCC-mb
N35 2.939907 2.248941 3.801245 Angstrom
End of Basis Set

Basis Set
N.ANO-RCC-mb
N36 2.133130 -0.319559 3.009389 Angstrom
End of Basis Set

Basis Set
C.ANO-RCC-mb
C37 1.496933 -1.492994 2.915930 Angstrom
End of Basis Set

Basis Set
H.ANO-RCC-mb
H38 1.897276 -2.252377 3.322053 Angstrom
End of Basis Set

Basis Set
C.ANO-RCC-mb
C39 0.293294 -1.666518 2.261715 Angstrom
End of Basis Set

Basis Set
H.ANO-RCC-mb
H40 -0.138379 -2.513522 2.249820 Angstrom
End of Basis Set

Basis Set
C.ANO-RCC-mb
C41 -0.267185 -0.570396 1.626192 Angstrom
End of Basis Set

```

```
Basis Set
H.ANO-RCC-mb
H42 -1.083536 -0.652863 1.147001 Angstrom
End of Basis Set

Basis Set
C.ANO-RCC-mb
C43 0.379455 0.625374 1.702659 Angstrom
End of Basis Set

Basis Set
H.ANO-RCC-mb
H44 0.000870 1.391629 1.284641 Angstrom
End of Basis Set

Basis Set
C.ANO-RCC-mb
C45 1.587445 0.742202 2.382363 Angstrom
End of Basis Set

Basis Set
C.ANO-RCC-mb
C46 2.311543 2.058236 2.443536 Angstrom
End of Basis Set

Basis Set
H.ANO-RCC-mb
H47 1.675347 2.795284 2.266813 Angstrom
End of Basis Set

Basis Set
H.ANO-RCC-mb
H48 3.013884 2.082289 1.746839 Angstrom
End of Basis Set

Basis Set
C.ANO-RCC-mb
C49 3.779756 3.480790 3.728176 Angstrom
End of Basis Set

Basis Set
H.ANO-RCC-mb
H50 3.205352 4.274533 3.877711 Angstrom
End of Basis Set

Basis Set
H.ANO-RCC-mb
H51 4.167915 3.554666 2.819072 Angstrom
End of Basis Set

Basis Set
C.ANO-RCC-mb
C52 1.831132 2.401848 4.776620 Angstrom
End of Basis Set

Basis Set
H.ANO-RCC-mb
H53 1.270653 1.585769 4.737537 Angstrom
```

```
End of Basis Set

Basis Set
H.ANO-RCC-mb
H54 1.266301 3.161230 4.487746 Angstrom
End of Basis Set

Basis Set
C.ANO-RCC-mb
C55 2.231475 2.628632 6.200600 Angstrom
End of Basis Set

Basis Set
C.ANO-RCC-mb
C56 1.751934 3.716164 6.936379 Angstrom
End of Basis Set

Basis Set
H.ANO-RCC-mb
H57 1.212342 4.369027 6.503068 Angstrom
End of Basis Set

Basis Set
C.ANO-RCC-mb
C58 2.043488 3.862199 8.271998 Angstrom
End of Basis Set

Basis Set
H.ANO-RCC-mb
H59 1.695364 4.600965 8.756287 Angstrom
End of Basis Set

Basis Set
C.ANO-RCC-mb
C60 2.837211 2.941319 8.904122 Angstrom
End of Basis Set

Basis Set
H.ANO-RCC-mb
H61 3.053918 3.056429 9.821723 Angstrom
End of Basis Set

Basis Set
C.ANO-RCC-mb
C62 3.327195 1.843478 8.214223 Angstrom
End of Basis Set

Basis Set
H.ANO-RCC-mb
H63 3.867658 1.209514 8.671324 Angstrom
End of Basis Set

Basis Set
C.ANO-RCC-mb
C64 3.040863 1.651056 6.868409 Angstrom
End of Basis Set

End of Input
```

```
>>COPY $FileDir/Dy_bbpenCl_2020_mb_mb_cas_9in7_ms6.RasOrb  $WorkDir/INPORB
&RASSCF
LUMO
SPIN
6
NACTEL
9 0 0
FROZ
0
INAC
157
RAS1
0
RAS2
7
RAS3
0
CIROOT
21 21 1
ITERations
200 20
CIMX
20
ORBL
ALL
ORBA
FULL
End Of Input


&MCLR
ITER
200
EXPD
21
PRINT
3
THREshold
1.0d-10
NAC
1 5
End Of Input


&ALASKA
SHOW
VERBOSE
CUTOFF
1.0d-14
PNEW
NAC
1 5
End Of Input
```

Listing S41: OpenMolcas input tailored towards the new two-step approach.

```
&SEWARD
Title
Dy_bbpenCl_2020
ANGM
8.2232377176152698280 2 .23989506234286763540 8.0278400363289652008 9
AMFI

DOAN
RICD
SDIP

CDTH
1.0d-6

VERBOSE

Basis Set
DY.ANO-RCC-mb
DY1 4.351550 0.126947 4.248150 Angstrom
End of Basis Set

Basis Set
CL.ANO-RCC-mb
CL2 4.351550 -2.554927 4.248150 Angstrom
End of Basis Set

Basis Set
O.ANO-RCC-mb
O3 5.196621 0.609911 2.312693 Angstrom
End of Basis Set

Basis Set
N.ANO-RCC-mb
N4 5.763193 2.248941 4.695055 Angstrom
End of Basis Set

Basis Set
N.ANO-RCC-mb
N5 6.569970 -0.319559 5.486911 Angstrom
End of Basis Set

Basis Set
C.ANO-RCC-mb
C6 7.206167 -1.492994 5.580370 Angstrom
End of Basis Set

Basis Set
H.ANO-RCC-mb
H7 6.805824 -2.252377 5.174247 Angstrom
End of Basis Set

Basis Set
C.ANO-RCC-mb
C8 8.409806 -1.666518 6.234585 Angstrom
End of Basis Set

Basis Set
```

```
H.ANO-RCC-mb
H9 8.841479 -2.513522 6.246480 Angstrom
End of Basis Set

Basis Set
C.ANO-RCC-mb
C10 8.970285 -0.570396 6.870108 Angstrom
End of Basis Set

Basis Set
H.ANO-RCC-mb
H11 9.786636 -0.652863 7.349299 Angstrom
End of Basis Set

Basis Set
C.ANO-RCC-mb
C12 8.323645 0.625374 6.793641 Angstrom
End of Basis Set

Basis Set
H.ANO-RCC-mb
H13 8.702230 1.391629 7.211659 Angstrom
End of Basis Set

Basis Set
C.ANO-RCC-mb
C14 7.115655 0.742202 6.113937 Angstrom
End of Basis Set

Basis Set
C.ANO-RCC-mb
C15 6.391557 2.058236 6.052764 Angstrom
End of Basis Set

Basis Set
H.ANO-RCC-mb
H16 7.027753 2.795284 6.229487 Angstrom
End of Basis Set

Basis Set
H.ANO-RCC-mb
H17 5.689216 2.082289 6.749461 Angstrom
End of Basis Set

Basis Set
C.ANO-RCC-mb
C18 4.923344 3.480790 4.768124 Angstrom
End of Basis Set

Basis Set
H.ANO-RCC-mb
H19 5.497748 4.274533 4.618589 Angstrom
End of Basis Set

Basis Set
H.ANO-RCC-mb
H20 4.535185 3.554666 5.677228 Angstrom
End of Basis Set
```

```

Basis Set
C.ANO-RCC-mb
C21 6.871968 2.401848 3.719680 Angstrom
End of Basis Set

Basis Set
H.ANO-RCC-mb
H22 7.432447 1.585769 3.758763 Angstrom
End of Basis Set

Basis Set
H.ANO-RCC-mb
H23 7.436799 3.161230 4.008554 Angstrom
End of Basis Set

Basis Set
C.ANO-RCC-mb
C24 6.471625 2.628632 2.295700 Angstrom
End of Basis Set

Basis Set
C.ANO-RCC-mb
C25 6.951166 3.716164 1.559921 Angstrom
End of Basis Set

Basis Set
H.ANO-RCC-mb
H26 7.490758 4.369027 1.993232 Angstrom
End of Basis Set

Basis Set
C.ANO-RCC-mb
C27 6.659612 3.862199 0.224302 Angstrom
End of Basis Set

Basis Set
H.ANO-RCC-mb
H28 7.007736 4.600965 -0.259987 Angstrom
End of Basis Set

Basis Set
C.ANO-RCC-mb
C29 5.865889 2.941319 -0.407822 Angstrom
End of Basis Set

Basis Set
H.ANO-RCC-mb
H30 5.649182 3.056429 -1.325423 Angstrom
End of Basis Set

Basis Set
C.ANO-RCC-mb
C31 5.375905 1.843478 0.282077 Angstrom
End of Basis Set

Basis Set
H.ANO-RCC-mb
```

```
H32 4.835442 1.209514 -0.175024 Angstrom
End of Basis Set

Basis Set
C.ANO-RCC-mb
C33 5.662237 1.651056 1.627891 Angstrom
End of Basis Set

Basis Set
O.ANO-RCC-mb
O34 3.506479 0.609911 6.183607 Angstrom
End of Basis Set

Basis Set
N.ANO-RCC-mb
N35 2.939907 2.248941 3.801245 Angstrom
End of Basis Set

Basis Set
N.ANO-RCC-mb
N36 2.133130 -0.319559 3.009389 Angstrom
End of Basis Set

Basis Set
C.ANO-RCC-mb
C37 1.496933 -1.492994 2.915930 Angstrom
End of Basis Set

Basis Set
H.ANO-RCC-mb
H38 1.897276 -2.252377 3.322053 Angstrom
End of Basis Set

Basis Set
C.ANO-RCC-mb
C39 0.293294 -1.666518 2.261715 Angstrom
End of Basis Set

Basis Set
H.ANO-RCC-mb
H40 -0.138379 -2.513522 2.249820 Angstrom
End of Basis Set

Basis Set
C.ANO-RCC-mb
C41 -0.267185 -0.570396 1.626192 Angstrom
End of Basis Set

Basis Set
H.ANO-RCC-mb
H42 -1.083536 -0.652863 1.147001 Angstrom
End of Basis Set

Basis Set
C.ANO-RCC-mb
C43 0.379455 0.625374 1.702659 Angstrom
End of Basis Set

```

```
Basis Set
H.ANO-RCC-mb
H44 0.000870 1.391629 1.284641 Angstrom
End of Basis Set

Basis Set
C.ANO-RCC-mb
C45 1.587445 0.742202 2.382363 Angstrom
End of Basis Set

Basis Set
C.ANO-RCC-mb
C46 2.311543 2.058236 2.443536 Angstrom
End of Basis Set

Basis Set
H.ANO-RCC-mb
H47 1.675347 2.795284 2.266813 Angstrom
End of Basis Set

Basis Set
H.ANO-RCC-mb
H48 3.013884 2.082289 1.746839 Angstrom
End of Basis Set

Basis Set
C.ANO-RCC-mb
C49 3.779756 3.480790 3.728176 Angstrom
End of Basis Set

Basis Set
H.ANO-RCC-mb
H50 3.205352 4.274533 3.877711 Angstrom
End of Basis Set

Basis Set
H.ANO-RCC-mb
H51 4.167915 3.554666 2.819072 Angstrom
End of Basis Set

Basis Set
C.ANO-RCC-mb
C52 1.831132 2.401848 4.776620 Angstrom
End of Basis Set

Basis Set
H.ANO-RCC-mb
H53 1.270653 1.585769 4.737537 Angstrom
End of Basis Set

Basis Set
H.ANO-RCC-mb
H54 1.266301 3.161230 4.487746 Angstrom
End of Basis Set

Basis Set
C.ANO-RCC-mb
C55 2.231475 2.628632 6.200600 Angstrom
```

```
End of Basis Set

Basis Set
C.ANO-RCC-mb
C56 1.751934 3.716164 6.936379 Angstrom
End of Basis Set

Basis Set
H.ANO-RCC-mb
H57 1.212342 4.369027 6.503068 Angstrom
End of Basis Set

Basis Set
C.ANO-RCC-mb
C58 2.043488 3.862199 8.271998 Angstrom
End of Basis Set

Basis Set
H.ANO-RCC-mb
H59 1.695364 4.600965 8.756287 Angstrom
End of Basis Set

Basis Set
C.ANO-RCC-mb
C60 2.837211 2.941319 8.904122 Angstrom
End of Basis Set

Basis Set
H.ANO-RCC-mb
H61 3.053918 3.056429 9.821723 Angstrom
End of Basis Set

Basis Set
C.ANO-RCC-mb
C62 3.327195 1.843478 8.214223 Angstrom
End of Basis Set

Basis Set
H.ANO-RCC-mb
H63 3.867658 1.209514 8.671324 Angstrom
End of Basis Set

Basis Set
C.ANO-RCC-mb
C64 3.040863 1.651056 6.868409 Angstrom
End of Basis Set

End of Input




>>COPY $FileDir/Dy_bbpenCl_2020_mb_mb_cas_9in7_ms6.RasOrb  $WorkDir/INPORB
&RASSCF
LUMORB
SPIN
6
NACTEL
```

```
9 0 0
FROZ
0
INAC
157
RAS1
0
RAS2
7
RAS3
0
CIROOT
21 21 1
ITERations
200 20
CIMX
20
ORBL
ALL
ORBA
FULL
End Of Input



&MCLR
ITER
200
EXPD
21
PRINT
3
THREshold
1.0d-10
SALA
1
TWOStep
FIRST
End Of Input


&MCLR
ITER
200
EXPD
21
PRINT
3
THREshold
1.0d-10
NAC
1 5
TWOStep
SECOND
End Of Input


&ALASKA
```

```
SHOW
VERBOSE
CUTOFF
1.0d-14
PNEW
NAC
1 5
End Of Input
```

S13 MC-PDFT Nonadiabatic Dynamics

The `SHARC–OpenMolcas` interface for MC-PDFT is available in the `MCPDFT` directory with example input and output files. In addition to replacing the interface script, the user must define the method as ***MCPDFT*** and provide the functional (e.g., ***t:PBE***) and grid size (***coarse***, ***sg1fine***, ***fine***, or ***ultrafine***) in the `MOLCAS.template` file. This interface script can also run simulations with CMS-PDFT by defining the method as ***CMS-PDFT***, although this has not been well tested yet.

S14 ANO-R Basis Set

Tools for basis set generation.

S14.1 Hartree–Fock energies for atoms with higher ANO-R-N basis sets

The convergence of the results with an increase of the basis set is essential test for consistency of the basis set. Many different extrapolation formulae exist,[S43–S45] all based on various empirical evaluations of basis set convergence. Regarding ANO-type of the basis sets, as it was pointed in the literature,[S46,S47] the standard contraction levels (e.g., ANO-L-VDZP, ANO-L-VTZP, ANO-L-VQZP) are not necessarily follow the extrapolation pattern.

Instead, one may exploit the fact that for ANO-type basis sets, any combination of contracted functions for a given element can be used.[S48,S49] To clarify, for C, there are 9 s, 7 p, 3 d and 2 f contracted basis functions in the ANO-R basis set. Thus, the largest ANO-R basis set for Ç is 9s7p4d2f, much larger than ANO-R-2 (4s4p2d1f), which was chosen as the largest standard contraction level for C based on the observations in the original ANO-R paper.[S50] By removing one shell of functions at a time from the full basis set, basis sets which allow for a smooth basis set extrapolation is obtained. Here, we generated four such basis sets for each element. For C, this means 9s7p3d2f, 8s6p2d1f, 7s5p1d and 6s4p.

Figure S11 shows the convergence of the Hartree–Fock energies for atoms with an increase of the basis set from ANO-R-1 to ANO-R-8. The higher than R-3 contraction schemes were made by increasing by “layers” of s-, p-, d- etc. orbitals.

Atomic states used in the calculations in this project taken from ref. S51. The biggest challenge for such calculations is to ensure that the converged results corresponds to the same electron configuration. The latter can be achieved by restarting the calculations from the the previous calculation with smaller basis set. For contractions higher than 3

the changes in the total energies are very small, and for the majority of the elements they are less than 10^{-6} E_h.

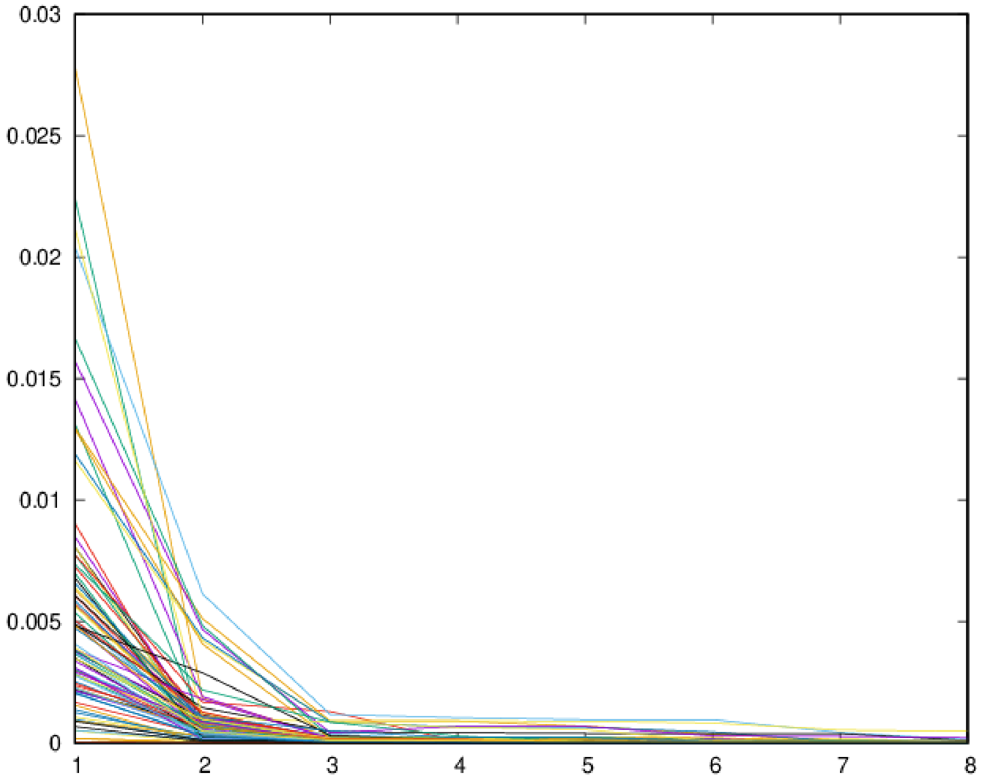

Figure S11: Convergence of Hartree–Fock atomic energies (from H to Rn) with an increase of the basis set quality. The x axis corresponds to ANO-R-N (N=1,2,3,...8), the y axis shows the error in E_h.

S14.2 Electron affinity of oxygen atom

Electron affinity for oxygen atom are computed as a difference between CASPT2 energies for the ground (triplet) state of oxygen and anion O^-. Active space used in the calculations include 1s2s2p3s3p orbitals. The further increase of the active space (to 14 orbitals) leads to a minor change of the results with the order of 0.02 eV.

S15 Use of `SCEPIC` program for generation of AIMPs and for creating input templates for `OpenMolcas` calculations

This section describes two possible use of `SCEPIC` program: Generation of ab initio model potentials (AIMP) for a new crystal and generation of input for `OpenMolcas` with the use of AIMPs.

Abbreviations used in the section include the quantum part (QP), potential part (PP), and point charges (CP). The QP is set by the coordinate file in XYZ format.

Table S17: Electron affinity of oxygen, in eV, computed with different ANO-R basis sets

N =	3	4	5	6	7	8	9	10
Ns($N-2$)p1d	−7.89	−0.60	0.31	0.67	0.93	0.99	0.99	0.99
Ns($N-1$)p1d	−0.77	0.27	0.66	0.93	0.99	0.99	0.99	
NsNp1d	0.13	0.62	0.92	0.99	0.99	0.99		
Ns($N-2$)p2d	−7.82	−0.47	0.44	0.80	1.04	1.10	1.10	1.10
NsNp2d	0.24	0.75	1.04	1.10	1.10	1.10		
Ns($N-2$)p3d1f	−7.68	−0.34	0.55	0.90	1.14	1.19	1.19	1.19
Ns($N-2$)p4d2f	−7.65	−0.29	0.59	0.94	1.18	1.23	1.23	1.23
Ns($N-2$)p4d2f1g	−7.59	−0.22	0.64	0.98	1.22	1.27	1.27	1.27
Ns($N-2$)p4d3f2g1h	−7.56	−0.19	0.67	1.02	1.25	1.30	1.30	1.30

S16 Ab Initio Model Potentials

Generation of AIMPs for a new crystal.

S16.1 Quick start

Ab initio model potentials should be generated for any new crystal structure. This section gives a step-by-step tutorial for this purpose. One option is to create input files for `SCEPIC` code directly, but for simple cases, it is possible to generate these inputs by `scepic_inp.plx` code, which can be downloaded from `SCEPIC` homepage (`https://www.molcas.org/SCEPIC`).

One needs to create an XYZ file for your periodic structure, with an extended `MOLCAS` format. An example of such a format is generated by running `scepic_inp.plx` code with a flag `-h`. The file should contain all atoms in the unit cell but in addition to that,

- it should contain a translation section at the end in the form of four lines, e.g.:

```
#Origin   0 0 0
#Vector1  2.0 0 0
#Vector2  0 2.0 0
#Vector3  0 0 2.0
```

- Atoms for which AIMPs should be constructed had additional parameters: charge and orbitals. The orbitals are given by letters (no spaces)

```
Mg 0 0 0 +2 sp
```

Note that Cartesian coordinates in the file should be inside the unit cell, which is defined by the vectors, so the partial coordinates are in the range $[0, 1]$.

In case one atom is located at different Wyckoff positions, the element name must be labeled, by adding an underscore sign and any label, e.g.,

```
Mg_a 0 0 0 +2 sp
```

After creating the coordinate file, execute `./scepic_inp.plx filename.xyz`. The script will create input files for `SCEPIC` (small scripts, written in Julia language). These scripts can be modified for more efficient use of `SCEPIC` program.

S16.2 Drivers for embedding script

There are two alternative ways to set up the size of the CP: by radius (*Charges_Radius*) or by extension of the unit cells (*Charges_Size*). These keywords are exclusive. The *Charges_Radius* keyword requires one number. It can be complemented by keyword *Charges_Center*, which defines the center of the sphere. If *Charges_Size* is defined instead, it requires one, three, or six numbers, defining the translation of the unit cell (uniform in all directions, symmetrical, or arbitrary). The setup of the AIMP region is similar to the setup of CP. Two alternative keywords can be used: *AIMP_Radius* or *AIMP_Size*.

The complete example of `driver.jl` script:

```
using embedding_factory

# define the coordinates of the system
#
# Unit cell file (it must contain in the comment line LATTICE followed by 9 numbers
uc_file = "./MgO_bulk.xyz"
# Quantum part (ordinary xyz file)
qm_file = "./MgO_clust.xyz"
#definition of elements and charges
charge_dict = Dict("Mg" => 2, "O" => -2)

# region of charges
Charges_Radius=20
Charges_Center=[0.0,0.0,0.0]
#Charges_Size=[4, 4, 4]

# region of AIMPs
AIMP_Radius=5
#AIMP_Size=[2, 2, 2]


#Multipoles to eliminate. Max=10, Default=4
MaxMultipole=3

#template for Spherical based cluster
input = Dict("elmoment" => MaxMultipole,
             "unit_cell_file" => uc_file,
             "qm_file" => qm_file,
             "cluster_radius" => Charges_Radius,
             "aimp_radius" => AIMP_Radius,
             "reference" => Charges_Center,
             "charge_dict" => charge_dict)

#template for Unit cell based cluster
input_UC = Dict("elmoment" => MaxMultipole,
             "unit_cell_file" => uc_file,
             "qm_file" => qm_file,
             "cluster_size" => Charges_Size,
             "aimp_radius" => AIMP_Size,
             "reference" => Charges_Center,
             "charge_dict" => charge_dict)
embedding(input)
#embedding(input_UC)
```

S16.3 Driver for embedding

For each atom in the system, one needs to prepare `xfield` file and `aimpfield` file. In order to do that we have to run individual calculations for each atom. The lattice coordinate file is the same as in the previous example, but the quantum part contains only one single atom. In the case of Mg, since it is located at $(0, 0, 0)$, the only modification we need is to change the coordinate file, keeping only one atom in it. To avoid confusion, you can copy the coordinate file, edit it and change the name of the file in the driver script.

In the driver file, we have to set ***Charges_Radius*** and ***AIMP_Radius*** to be large enough. ***AIMP_Radius*** must be at least 6 Å, and for the Charges – the electrostatic potential should converge.

Run `julia driver.jl` command. Rename `project.xfield` to `Mg.xfield`. Rename `project.inp` to `Mg.aimpfield` and remove from the top of the file the first 4 lines (the basis set for the real atom).

Next, we need to repeat the procedure for O atom. The coordinate file is:

```
1
comment
O 2.128242 0 0
```

Since O is not in the center of the coordinates, we have to change the center of the sphere in the driver script:

```
qm_file = "./O_clust.xyz"
Charges_Radius=40
Charges_Center=[2.1282420,0.0,0.0]
AIMP_Radius=10
```

Run `julia driver-emb.jl` and as before rename `project.xfield` to `O.xfield` and `project.inp` to `O.aimpfield` (remove 4 first lines).

S16.4 Driver script for SCEPIC

At the beginning of the script, there is a definition for each ion, which include the following parameters: element name, position (should match the corresponding selection in embedding input, formal atomic charge, exponents for valence shells (s- exponents are compulsory, others are optional), relativistic hamiltonian, nucleus, indication of Restricted or Unrestricted Hartree–Fock hamiltonian, DFT functional, and links to the files, generated by embedding program.

The exponents can be found in the basis library file. For UHF hamiltonian, the Multiplicity must be set.

Example of the driver file for MgO

```
using scepic_module

Mg_s = [169973.87,24699.066,7175.4851,2478.7920,917.73259,351.75140,
        137.51557,54.430286,21.722569,8.7194484,3.5147166,1.4212132,
        .57607650,.23395250,.09515600,.03875100,.01550040]
Mg_p = [557.13078,135.48388,47.212958,18.000955,7.1146607,2.8630240,
        1.1640185,.47630470,.19573560,.08067830,.03332660,.01333060]
ion1 = Dict("element" => "Mg",
            "position" => [ 0.00000000, 0.00000000, 0.00000000],
            "charge" => +2,
            "s_basis" =>Mg_s,
```

```
            "p_basis" =>Mg_p,
            "hamiltonian" => "rx2c",
            "nucleus" => "finite",
            "SCF" => "RHF",
            "ksdft" => "PBE",
            "AIMPlabel" => "Mg.ECP.author.0s.0s.0e-update",
            "AIMPembedding" => "./Mg.aimpfield",
            "xfield" => "./Mg.xfield")

O_s = [105374.95,15679.240,3534.5447,987.36516,315.97875,111.65428,
       42.699451,17.395596,7.4383090,3.2228620,1.2538770,.49515500,
       .19166500,.06708300]
O_p = [200.00000,46.533367,14.621809,5.3130640,2.1025250,.85022300,
       .33759700,.12889200,.04511200]
ion2 = Dict("element" => "O",
            "position" => [ 2.12824200, 0.00000000, 0.00000000],
            "charge" => -2,
            "s_basis" =>O_s,
            "p_basis" =>O_p,
            "hamiltonian" => "rx2c",
            "nucleus" => "finite",
            "SCF" => "RHF",
            "ksdft" => "PBE",
            "AIMPlabel" => "O.ECP.author.0s.0s.0e-update",
            "AIMPembedding" => "./O.aimpfield",
            "xfield" => "./O.xfield")

ion_dict =[ion1, ion2]
input = Dict("maxiter" => 20)

scepic(ion_dict)#, input)
```

Executing this script will generate ECP file to be appended to AIMP file in `basis_library` directory of `OpenMolcas`.

S16.5 Generation of input for `OpenMolcas` calculation with AIMPs

`SCEPIC` (or, more precisely, the embedding part of `SCEPIC` code) can be used for the generation of a template for `OpenMolcas` input. One should start from a selection of the quantum part of the cluster. The cluster can have a charge, which later will be compensated by electrostatic field. The positively charges clusters are preferred.

At this step it is important that all atoms in the QC part are located in the exact positions as in the ideal crystal. After the generation of the inputs, the atoms in the QC part can be move, e.g., accordingly to optimized geometry.

S16.6 Diffusion of oxygen ad-atom on MgO(001)

In this section, the usage of AIMPs for surface reactions will be discussed, exemplified by the diffusion of an oxygen ad-atom on the MgO(001) surface. This reaction has previously been proposed to be an example of a spin-crossing, where the lowest energy route would correspond to changing between singlet and triplet spin-states.[S52] Spin-crossing reactions are typical examples of where multiconfigurational approaches are important, for which reason we reexamined this reaction using an AIMP embedding.

To generate the geometries for the reaction, the following steps were followed. First, the geometries were optimized by periodic DFT. Thereafter, an embedding environment was generated for a pristine MgO(001) surface; in the production calculations, the periodic DFT geometries were pasted into this embedding. Since AIMPs are, in principle at least, sensitive to the position in which it was optimized, two sets of AIMPs were used, one to represent bulk ions and one to represent surface ions. This can be achieved in `SCEPIC` by first generating surface embedding environments, which require that ***surface => true*** in the ***embedding*** code to generate an appropriate embedding environment (the code assumes that the surface normal is parallel to the third unit-cell vector) and then optimizing a set of bulk AIMPs, which are then frozen in the optimization of surface AIMPs, using the keyword ***external_AIMPs***. Note that in this case, no coordinates in the QM region can be larger than 1.0 in the internal coordinates of the unit-cell. Once these steps have been followed, typical AIMP calculations can be made in `OpenMolcas`.

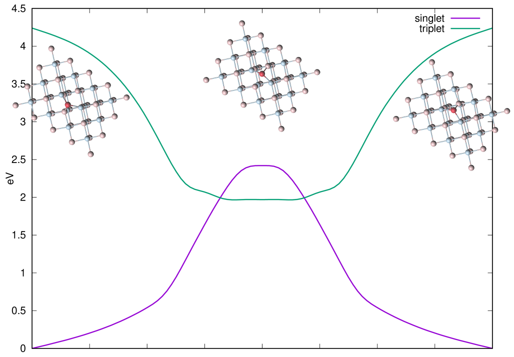

Figure S12: Diffusion add-atom oxygen on the surface of MgO

A preliminary set of results for this diffusion reaction is presented in fig. S12. These were obtained by performing DMRGSCF (`QCMaquis`) calculations, correlating the 2s2p3s3p orbitals of the three oxygens involved in the diffusion step. Preliminary calculations were made with a fairly small basis set, x2c-SVPALL with the timing for integrals being around 24 h for an Mg_8O_{20}-cluster (plus the adsorbed oxygen) and the DMRGSCF iterations similarly being around 24 h. An example input is presented below. On the border AIMPs, orthogonality functions have been placed.[S53]

```
&GATEWAY
  Basis set
    Mg.X2C-SVPAll
    Mg1             0.0431225000   2.2225390000   2.2235268571  Angstrom
    Mg2             2.2226725000   0.0432160000   2.2237518571  Angstrom
    Mg3            -2.2551525000  -0.0009020000   2.1418498571  Angstrom
    Mg4            -0.0009145000  -2.2551750000   2.1418698571  Angstrom
    Mg5             2.1787395000   4.3178470000   2.1031268571  Angstrom
    Mg6             4.3176005000   2.1783730000   2.0683868571  Angstrom
    Mg7             0.0012615000   0.0012900000  -0.0335731429  Angstrom
    Mg8             2.1418855000   2.1425770000   0.0090408571  Angstrom
  End of Basis
  Basis set
    O.X2C-SVPAll
    O1              0.4747975000   0.4748860000   3.2619938571  Angstrom
    O2              2.1325085000  -2.1395600000   2.1531948571  Angstrom
    O3             -2.1397605000   2.1323460000   2.1530178571  Angstrom
    O4              0.0034335000   4.3110760000   2.1450798571  Angstrom
```

```
    O5               4.3112325000   0.0035180000   2.1380178571  Angstrom
    O6              -2.1804605000  -2.1804810000   2.1358688571  Angstrom
    O7               4.3126935000   4.3121300000   2.1151308571  Angstrom
    O8              -4.3070655000   0.0002130000   2.1187428571  Angstrom
    O9               0.0002465000  -4.3070260000   2.1187428571  Angstrom
    O10              2.1476645000   6.4336420000   2.1186868571  Angstrom
    O11              6.4331045000   2.1473620000   2.1111888571  Angstrom
    O12             -0.2125775000  -0.2125660000   2.0809038571  Angstrom
    O13              2.2283395000   2.2274170000   2.0849338571  Angstrom
    O14              0.0095665000   2.1229320000   0.0476488571  Angstrom
    O15              2.1229595000   0.0102300000   0.0473448571  Angstrom
    O16             -0.0029405000  -2.1389070000   0.0064948571  Angstrom
    O17             -2.1389785000  -0.0030590000   0.0064398571  Angstrom
    O18              2.1428335000   4.2991510000  -0.0140371429  Angstrom
    O19              4.2991175000   2.1427510000  -0.0204941429  Angstrom
    O20              0.0026455000   0.0026820000  -2.1020091429  Angstrom
    O21              2.1357225000   2.1358840000  -2.0948771429  Angstrom
  End of Basis
  Basis set
    * Surface border AIMP
    Mg.ECP.Larsson.14s7p.1s1p.0e-PBE-MgO(001) / AIMPLIB
    pseudocharge
    Mg9              2.1392500000       8.5570000000        2.1392500000  Angstrom
    ...
    Mg22             0.0000000000      -6.4177500000        2.1392500000  Angstrom
  End of Basis
  Basis set
    * Bulk border AIMP
    Mg.ECP.Larsson.14s7p.1s1p.0e-PBE-MgO(bulk) / AIMPLIB
    pseudocharge
    Mg23             2.1392500000       6.4177500000        0.0000000000  Angstrom
    ...
    Mg40             0.0000000000       0.0000000000       -4.2785000000  Angstrom
  End of Basis
  Basis set
    * Surface AIMP
    Mg.ECP.Larsson.0s.0s.0e-PBE-MgO(001) / AIMPLIB
    pseudocharge
    A1              -6.4177500000      -4.2785000000        2.1392500000  Angstrom
    ...
    A78             14.9747500000      12.8355000000        2.1392500000  Angstrom
  End of Basis
  Basis set
    * Surface AIMP
    O.ECP.Larsson.0s.0s.0e-PBE-MgO(001) / AIMPLIB
    pseudocharge
    A79             -4.2785000000      -4.2785000000        2.1392500000  Angstrom
    ...
    A164            14.9747500000      14.9747500000        2.1392500000  Angstrom
  End of Basis
  Basis set
    * Bulk AIMP
    Mg.ECP.Larsson.0s.0s.0e-PBE-MgO(001) / AIMPLIB
    pseudocharge
    A165            -4.2785000000       2.1392500000       -2.1392500000  Angstrom
    ...
    A830            14.9747500000      14.9747500000      -12.8355000000  Angstrom
  End of Basis
```

```
  Basis set
    * Bulk AIMP
    O.ECP.Larsson.0s.0s.0e-PBE-MgO(001) / AIMPLIB
    pseudocharge
    A831            -2.1392500000         2.1392500000        -2.1392500000   Angstrom
    ...
    A1508           14.9747500000        14.9747500000       -10.6962500000   Angstrom
  End of Basis
  xfield = $CurrDir/$Project.xfield

&SEWARD

&DMRGSCF
  ActiveSpaceOptimizer = QCMaquis
  Fiedler = on
  DMRGSettings
    nsweeps = 16
    max_bond_dimension = 1000
    donotdelete = 1
  EndDMRGSettings
  OOptimizationSettings
    fileorb = $CurrDir/$Project.dmrgscf.h5
      spin = 1
     nactel = 22
  EndOOptimizationSettings
```

References

(S1) Dobrautz, W.; Weser, O.; Bogdanov, N. A.; Alavi, A.; Li Manni, G. Spin-Pure Stochastic-CASSCF via GUGA-FCIQMC Applied to Iron–Sulfur Clusters. *J. Chem. Theory Comput.* **2021**, *17*, 5684–5703, DOI: 10.1021/acs.jctc.1c00589.

(S2) Balasubramani, S. G.; Chen, G. P.; Coriani, S.; Diedenhofen, M.; Frank, M. S.; Franzke, Y. J.; Furche, F.; Grotjahn, R.; Harding, M. E.; Hättig, C.; Hellweg, A.; Helmich-Paris, B.; Holzer, C.; Huniar, U.; Kaupp, M.; Khah, A. M.; Khani, S. K.; Müller, T.; Mack, F.; Nguyen, B. D.; Parker, S. M.; Perlt, E.; Rappoport, D.; Reiter, K.; Roy, S.; Rückert, M.; Schmitz, G.; Sierka, M.; Tapavicza, E.; Tew, D. P.; van Wüllen, C.; Voora, V. K.; Weigend, F.; Wodyński, A.; Yu, J. M. TURBOMOLE: Modular program suite for *ab initio* quantum-chemical and condensed-matter simulations. *J. Chem. Phys.* **2020**, *152*, 184107, DOI: 10.1063/5.0004635.

(S3) Cave, R. J.; Newton, M. D. Generalization of the Mulliken-Hush treatment for the calculation of electron transfer matrix elements. *Chem. Phys. Lett.* **1996**, *249*, 15–19, DOI: 10.1016/0009-2614(95)01310-5.

(S4) Lévêque, J.; Rebolini, E.; Saúl, A.; Lepetit, M.-B. Theoretical study of the magnetic properties of $BaNiF_4$. *Eur. Phys. J. B* **2021**, *94*, 214, DOI: 10.1140/epjb/s10051-021-00225-5.

(S5) Lévêque, J.; Rebolini, E.; Lepetit, M.-B.; Saúl, A. Theoretical study of the magnetic properties of the $CoCu_2O_3$ compound. *Phys. Rev. B* **2022**, *106*, DOI: 10.1103/physrevb.106.224402.

(S6) Gellé, A.; Varignon, J.; Lepetit, M.-B. Accurate evaluation of magnetic coupling between atoms with numerous open shells: An ab initio method. *Europhys. Lett.* **2009**, *88*, 37003, DOI: 10.1209/0295-5075/88/37003.

(S7) Kaufmann, K.; Baumeister, W.; Jungen, M. Universal Gaussian basis sets for an optimum representation of Rydberg and continuum wavefunctions. *J. Phys. B: At., Mol. Opt. Phys.* **1989**, *22*, 2223–2240, DOI: 10.1088/0953-4075/22/14/007.

(S8) Roos, B. O.; Taylor, P. R.; Siegbahn, P. E. M. A complete active space SCF method (CASSCF) using a density matrix formulated super-CI approach. *Chem. Phys.* **1980**, *48*, 157–173, DOI: 10.1016/0301-0104(80)80045-0.

(S9) Roos, B. O.; Lindh, R.; Malmqvist, P.-Å.; Veryazov, V.; Widmark, P.-O. Main Group Atoms and Dimers Studied with a New Relativistic ANO Basis Set. *J. Phys. Chem. A* **2004**, *108*, 2851–2858, DOI: 10.1021/jp031064+.

(S10) Dittrich, B.; Fabbiani, F. P. A.; Henn, J.; Schmidt, M. U.; Macchi, P.; Meindl, K.; Spackman, M. A. Azulene revisited: solid-state structure, invariom modeling and lattice-energy minimization of a classical example of disorder. *Acta Crystallogr., Sect. B: Struct. Sci., Cryst. Eng. Mater.* **2018**, *74*, 416–426, DOI: 10.1107/s2052520618010120.

(S11) Finley, J.; Malmqvist, P.-Å.; Roos, B. O.; Serrano-Andrés, L. The multi-state CASPT2 method. *Chem. Phys. Lett.* **1998**, *288*, 299–306, DOI: 10.1016/s0009-2614(98)00252-8.

(S12) Ghigo, G.; Roos, B. O.; Malmqvist, P.-Å. A modified definition of the zeroth-order Hamiltonian in multiconfigurational perturbation theory (CASPT2). *Chem. Phys. Lett.* **2004**, *396*, 142–149, DOI: 10.1016/j.cplett.2004.08.032.

(S13) Forsberg, N.; Malmqvist, P.-Å. Multiconfiguration perturbation theory with imaginary level shift. *Chem. Phys. Lett.* **1997**, *274*, 196–204, DOI: 10.1016/s0009-2614(97)00669-6.

(S14) Aquilante, F.; Pedersen, T. B.; Lindh, R. Low-cost evaluation of the exchange Fock matrix from Cholesky and density fitting representations of the electron repulsion integrals. *J. Chem. Phys.* **2007**, *126*, 194106, DOI: 10.1063/1.2736701.

(S15) Aquilante, F.; Pedersen, T. B.; Lindh, R.; Roos, B. O.; de Merás, A. S.; Koch, H. Accurate *ab initio* density fitting for multiconfigurational self-consistent field methods. *J. Chem. Phys.* **2008**, *129*, 024113, DOI: 10.1063/1.2953696.

(S16) Aquilante, F.; Malmqvist, P.-Å.; Pedersen, T. B.; Ghosh, A.; Roos, B. O. Cholesky Decomposition-Based Multiconfiguration Second-Order Perturbation Theory (CD-CASPT2): Application to the Spin-State Energetics of Co^{III}(diiminato)(NPh). *J. Chem. Theory Comput.* **2008**, *4*, 694–702, DOI: 10.1021/ct700263h.

(S17) Vosko, S. H.; Wilk, L.; Nusair, M. Accurate spin-dependent electron liquid correlation energies for local spin density calculations: a critical analysis. *Can. J. Phys.* **1980**, *58*, 1200–1211, DOI: 10.1139/p80-159.

(S18) Lee, C.; Yang, W.; Parr, R. G. Development of the Colle–Salvetti correlation-energy formula into a functional of the electron density. *Phys. Rev. B* **1988**, *37*, 785–789, DOI: 10.1103/physrevb.37.785.

(S19) Becke, A. D. A new mixing of Hartree–Fock and local density-functional theories. *J. Chem. Phys.* **1993**, *98*, 1372–1377, DOI: 10.1063/1.464304.

(S20) Becke, A. D. Density-functional thermochemistry. III. The role of exact exchange. *J. Chem. Phys.* **1993**, *98*, 5648–5652, DOI: 10.1063/1.464913.

(S21) Weigend, F.; Ahlrichs, R. Balanced basis sets of split valence, triple zeta valence and quadruple zeta valence quality for H to Rn: Design and assessment of accuracy. *Phys. Chem. Chem. Phys.* **2005**, *7*, 3297, DOI: 10.1039/b508541a.

(S22) Titov, E. On the Low-Lying Electronically Excited States of Azobenzene Dimers: Transition Density Matrix Analysis. *Molecules* **2021**, *26*, 4245, DOI: 10.3390/molecules26144245.

(S23) Malmqvist, P. Å.; Pierloot, K.; Shahi, A. R. M.; Cramer, C. J.; Gagliardi, L. The restricted active space followed by second-order perturbation theory method: Theory and application to the study of CuO_2 and Cu_2O_2 systems. *J. Chem. Phys.* **2008**, *128*, 204109, DOI: 10.1063/1.2920188.

(S24) Sauri, V.; Serrano-Andrés, L.; Shahi, A. R. M.; Gagliardi, L.; Vancoillie, S.; Pierloot, K. Multiconfigurational Second-Order Perturbation Theory Restricted Active Space (RASPT2) Method for Electronic Excited States: A Benchmark Study. *J. Chem. Theory Comput.* **2011**, *7*, 153–168, DOI: 10.1021/ct100478d.

(S25) Fujii, T.; Kashida, H.; Asanuma, H. Analysis of Coherent Heteroclustering of Different Dyes by Use of Threoninol Nucleotides for Comparison with the Molecular Exciton Theory. *Chem. – Eur. J.* **2009**, *15*, 10092–10102, DOI: 10.1002/chem.200900962.

(S26) Kaiser, A.; Daoud, R. E.; Aquilante, F.; Kühn, O.; De Vico, L.; Bokarev, S. I. A Multiconfigurational Wave Function Implementation of the Frenkel Exciton Model for Molecular Aggregates. *J. Chem. Theory Comput.* **2023**, DOI: 10.1021/acs.jctc.3c00185, In press.

(S27) Tenorio, B. N. C.; Decleva, P.; Coriani, S. Multi-reference approach to the computation of double core-hole spectra. *J. Chem. Phys.* **2021**, *155*, 131101, DOI: 10.1063/5.0062130.

(S28) Tenorio, B. N. C.; Voß, T. A.; Bokarev, S. I.; Decleva, P.; Coriani, S. Multireference Approach to Normal and Resonant Auger Spectra Based on the One-Center Approximation. *J. Chem. Theory Comput.* **2022**, *18*, 4387–4407, DOI: 10.1021/acs.jctc.2c00252.

(S29) Ferté, A.; Palaudoux, J.; Penent, F.; Iwayama, H.; Shigemasa, E.; Hikosaka, Y.; Soejima, K.; Ito, K.; Lablanquie, P.; Taïeb, R.; Carniato, S. Advanced Computation Method for Double Core Hole Spectra: Insight into the Nature of Intense Shake-up Satellites. *J. Phys. Chem. Lett.* **2020**, *11*, 4359–4366, DOI: 10.1021/acs.jpclett.0c01167.

(S30) Eberhardt, W.; Stöhr, J.; Feldhaus, J.; Plummer, E. W.; Sette, F. Correlation between Electron Emission and Fragmentation into Ions following Soft-X-Ray Excitation of the N_2 Molecule. *Phys. Rev. Lett.* **1983**, *51*, 2370–2373, DOI: 10.1103/physrevlett.51.2370.

(S31) Kaliakin, D. S. OpenMolcas–GAMESS/Tinker interface repository. `https://gitlab.com/interface-between/openmolcas-and-gamess-us-with-tinker`.

(S32) Ferré, N.; Ángyán, J. G. Approximate electrostatic interaction operator for QM/MM calculations. *Chem. Phys. Lett.* **2002**, *356*, 331–339, DOI: 10.1016/s0009-2614(02)00343-3.

(S33) Aquilante, F.; Autschbach, J.; Baiardi, A.; Battaglia, S.; Borin, V. A.; Chibotaru, L. F.; Conti, I.; De Vico, L.; Delcey, M.; Fdez. Galván, I.; Ferré, N.; Freitag, L.; Garavelli, M.; Gong, X.; Knecht, S.; Larsson, E. D.; Lindh, R.; Lundberg, M.; Malmqvist, P. Å.; Nenov, A.; Norell, J.; Odelius, M.; Olivucci, M.; Pedersen, T. B.; Pedraza-González, L.; Phung, Q. M.; Pierloot, K.; Reiher, M.; Schapiro, I.; Segarra-Martí, J.; Segatta, F.; Seijo, L.; Sen, S.; Sergentu, D.-C.; Stein, C. J.; Ungur, L.; Vacher, M.; Valentini, A.; Veryazov, V. Modern quantum chemistry with [Open]Molcas. *J. Chem. Phys.* **2020**, *152*, 214117, DOI: 10.1063/5.0004835.

(S34) Huix-Rotllant, M.; Ferré, N. Analytic Energy, Gradient, and Hessian of Electrostatic Embedding QM/MM Based on Electrostatic Potential-Fitted Atomic Charges Scaling Linearly with the MM Subsystem Size. *J. Chem. Theory Comput.* **2020**, *17*, 538–548, DOI: 10.1021/acs.jctc.0c01075.

(S35) Yang, X.; Manathunga, M.; Gozem, S.; Léonard, J.; Andruniów, T.; Olivucci, M. Quantum–classical simulations of rhodopsin reveal excited-state population splitting and its effects on quantum efficiency. *Nat. Chem.* **2022**, *14*, 441–449, DOI: 10.1038/s41557-022-00892-6.

(S36) Pedraza-González, L.; Barneschi, L.; Padula, D.; De Vico, L.; Olivucci, M. Evolution of the Automatic Rhodopsin Modeling (ARM) Protocol. *Top Curr Chem (Z)* **2022**, *380*, DOI: 10.1007/s41061-022-00374-w.

(S37) Pedraza-González, L.; De Vico, L.; del Carmen Marín, M.; Fanelli, F.; Olivucci, M. *a*-ARM: Automatic Rhodopsin Modeling with Chromophore Cavity Generation, Ionization State Selection, and External Counterion Placement. *J. Chem. Theory Comput.* **2019**, *15*, 3134–3152, DOI: 10.1021/acs.jctc.9b00061.

(S38) Melaccio, F.; del Carmen Marín, M.; Valentini, A.; Montisci, F.; Rinaldi, S.; Cherubini, M.; Yang, X.; Kato, Y.; Stenrup, M.; Orozco-Gonzalez, Y.; Ferré, N.; Luk, H. L.; Kandori, H.; Olivucci, M. Toward Automatic Rhodopsin Modeling as a Tool for High-Throughput Computational Photobiology. *J. Chem. Theory Comput.* **2016**, *12*, 6020–6034, DOI: 10.1021/acs.jctc.6b00367.

(S39) Paolino, M.; Giovannini, T.; Manathunga, M.; Latterini, L.; Zampini, G.; Pierron, R.; Léonard, J.; Fusi, S.; Giorgi, G.; Giuliani, G.; Cappelli, A.; Cappelli, C.; Olivucci, M. On the Transition from a Biomimetic Molecular Switch to a Rotary Molecular Motor. *J. Phys. Chem. Lett.* **2021**, *12*, 3875–3884, DOI: 10.1021/acs.jpclett.1c00526.

(S40) Chatterjee, G.; Jha, A.; Blanco-Gonzalez, A.; Tiwari, V.; Manathunga, M.; Duan, H.-G.; Tellkamp, F.; Prokhorenko, V. I.; Ferré, N.; Dasgupta, J.; Olivucci, M.; Miller, R. J. D. Torsionally broken symmetry assists infrared excitation of biomimetic charge-coupled nuclear motions in the electronic ground state. *Chem. Sci.* **2022**, *13*, 9392–9400, DOI: 10.1039/d2sc02133a.

(S41) Filatov, M. Spin-restricted ensemble-referenced Kohn–Sham method: basic principles and application to strongly correlated ground and excited states of molecules. *Wiley Interdiscip. Rev.: Comput. Mol. Sci.* **2015**, *5*, 146–167, DOI: 10.1002/wcms.1209.

(S42) Ferré, N., Filatov, M., Huix-Rotllant, M., Eds. *Density-Functional Methods for Excited States*; Topics in Current Chemistry 368; Springer International Publishing, 2016; DOI: 10.1007/978-3-319-22081-9.

(S43) Helgaker, T.; Klopper, W.; Koch, H.; Noga, J. Basis-set convergence of correlated calculations on water. *J. Chem. Phys.* **1997**, *106*, 9639–9646, DOI: 10.1063/1.473863.

(S44) Halkier, A.; Helgaker, T.; Jørgensen, P.; Klopper, W.; Koch, H.; Olsen, J.; Wilson, A. K. Basis-set convergence in correlated calculations on Ne, N_2, and H_2O. *Chem. Phys. Lett.* **1998**, *286*, 243–252, DOI: 10.1016/s0009-2614(98)00111-0.

(S45) Schwenke, D. W. The extrapolation of one-electron basis sets in electronic structure calculations: How it should work and how it can be made to work. *J. Chem. Phys.* **2005**, *122*, 014107, DOI: 10.1063/1.1824880.

(S46) Neese, F.; Valeev, E. F. Revisiting the Atomic Natural Orbital Approach for Basis Sets: Robust Systematic Basis Sets for Explicitly Correlated and Conventional Correlated *ab initio* Methods? *J. Chem. Theory Comput.* **2011**, *7*, 33–43, DOI: 10.1021/ct100396y.

(S47) Granatier, J. Extrapolation of Atomic Natural Orbitals of basis set to complete basis set limit. *Acta Chim. Slovaca* **2017**, *10*, 159–164, DOI: 10.1515/acs-2017-0026.

(S48) Widmark, P.-O.; Persson, B. J.; Roos, B. O. Density matrix averaged atomic natural orbital (ANO) basis sets for correlated molecular wave functions. *Theor. Chim. Acta* **1991**, *79*, 419–432, DOI: 10.1007/bf01112569.

(S49) Roos, B. O.; Lindh, R.; Malmqvist, P.-Å.; Veryazov, V.; Widmark, P.-O. New Relativistic ANO Basis Sets for Transition Metal Atoms. *J. Phys. Chem. A* **2005**, *109*, 6575–6579, DOI: 10.1021/jp0581126.

(S50) Zobel, J. P.; Widmark, P.-O.; Veryazov, V. The ANO-R Basis Set. *J. Chem. Theory Comput.* **2020**, *16*, 278–294, DOI: 10.1021/acs.jctc.9b00873.

(S51) Kelleher, D. E.; Martin, W. C.; Wiese, W. L.; Sugar, J.; Fuhr, J. R.; Olsen, K.; Musgrove, A.; Mohr, P. J.; Reader, J.; Dalton, G. R. The New NIST Atomic Spectra Database. *Phys. Scr.* **1999**, *T83*, 158, DOI: 10.1238/physica.topical.083a00158.

(S52) Kantorovich, L. N.; Gillan, M. J.; White, J. A. Adsorption of atomic oxygen on the MgO (100) surface. *J. Chem. Soc., Faraday Trans.* **1996**, *92*, 2075, DOI: 10.1039/ft9969202075.

(S53) Larsson, E. D.; Krośnicki, M.; Veryazov, V. A program system for self-consistent embedded potentials for ionic crystals. *Chem. Phys.* **2022**, *562*, 111549, DOI: 10.1016/j.chemphys.2022.111549.
